# NUDT1 Could Be a Prognostic Biomarker and Correlated with Immune Infiltration in Clear Cell Renal Cell Carcinoma

**DOI:** 10.1155/2022/3669296

**Published:** 2022-12-26

**Authors:** Yongshuai Lin, Facai Zhang, Yinling Jin, Quliang Zhong, Weiwei Tan, Jinyang Liu, Zhiping Wu

**Affiliations:** ^1^Guizhou Medical University, Guiyang 550004, China; ^2^Department of Urology, Zhejiang Provincial People's Hospital, Hangzhou 310014, China; ^3^Department of Ophthalmology, The First Affiliated Hospital of Hainan Medical University, Haikou 570102, China; ^4^Department of Urology, The Affiliated Hospital of Guizhou Medical University, Guiyang 550004, China; ^5^Department of Urology, Qiannanzhou People's Hospital, Duyun 558000, China

## Abstract

**Background:**

Clear cell renal cell carcinoma (ccRCC) is a malignant tumor with high morbidity and mortality. As a member of the Nudix hydrolase superfamily, Nudix (nucleoside diphosphate-linked moiety X)-type motif 1 (NUDT1) is closely related to the occurrence and development of cancer. Our study aims to explore the role of NUDT1 in ccRCC and its relationship with immune infiltration.

**Methods:**

The NUDT1 expression matrix and corresponding clinical information were obtained from The Cancer Genome Atlas (TCGA) database. The expression difference of NUDT1 in ccRCC and its relationship with the clinical characteristics were investigated using R software. Kaplan–Meier (K–M) analysis, univariate Cox regression, multivariate Cox regression, receiver operating characteristic (ROC) curve, and nomogram were utilized to evaluate the survival and prognosis of patients. Gene Ontology (GO) and Kyoto Encyclopedia of Genes and Genomes (KEGG) were utilized to explore the function of differential genes in low- or high-expression group of NUDT1. TCGA dataset and Tumor IMmune Estimation Resource (TIMER) database were utilized to explore the relationship between NUDT1 and immune infiltration. Finally, TCGA dataset was utilized for gene set enrichment analysis (GSEA).

**Results:**

NUDT1 was not only overexpressed in ccRCC but also significantly correlated with clinicopathological features (*P* < 0.05). K–M survival analysis showed that upregulated NUDT1 was closely related to the decrease of overall survival (OS) and progression-free survival (PFS) in ccRCC patients. Multivariate Cox regression revealed that NUDT1 was a independent prognostic indicator (HR = 1.437, 95% CI: 1.065–1.939, *P*=0.018). The ROC curve showed that NUDT1 had a certain accuracy in predicting the outcome of ccRCC patiens. Furthermore, a total of 150 coexpressed genes and 1,886 differentially expressed genes (DEGs) were identified. GO/KEGG and GSEA results suggested that NUDT1 and its DEGs were involved in the immune-related pathways. NUDT1 expression was positively correlated with infiltrating levels of regulatory T cells (Tregs), CD8^+^ T cells, follicular helper T cells, and M0 macrophages. In addition, NUDT1 was positively related to immune checkpoints, such as PD-1, LAG3, CTLA4, and CD70, in ccRCC.

**Conclusion:**

NUDT1 plays a key role in the prognosis and immune cell infiltration of ccRCC patients, indicating its potential use as a prognostic biomarker and therapeutic target.

## 1. Introduction

Renal cell carcinoma (RCC) is the third urological cancer, representing 3% of all cancers in women and 5% in men, with around 400,000 cases worldwide [1]. Clear cell renal cell carcinoma (ccRCC) is the most common pathological type of RCC, accounting for more than 70% of adult patients [2, 3]. ccRCC is not susceptible to chemoradiotherapy; the current antitumor treatment schemes mainly include partial or radical nephrectomy, local ablation, targeted therapy, and immunotherapy [4, 5]. Approximately, 25%–30% of ccRCC patients have metastatic disease at initial presentation and between 20% and 40% relapse after nephrectomy for localized disease [6]. Although the mechanism of cancer occurrence and development has been extensively studied, considering the high morbidity and mortality of ccRCC, it is essential to explore the molecular signature with prognostic value in ccRCC patients.

Nudix (nucleoside diphosphate-linked moiety X)-type motif 1 (NUDT1), also known as mutT human homolog 1 (MTH1), is an enzyme that encoded by the NUDT1 gene in humans [7]. It has been reported that NUDT1 is overexpressed in various cancers, including cancers of the bladder, breast, colon, and kidney [8, 9]. Overexpression of NUDT1 may have a series of protective effects on cancer cells by hydrolyzing 8-oxo-dGTP or other oxidized nucleotides produced by endogenous elevated reactive oxygen species (ROS) or by therapy-induced ROS, resulting in the malignant phenotypes, poor prognosis, and drug resistance of cancer patients [10]. A study shown that the overexpression of NUDT1 is closely correlated to the patients' case history and clinicopathological characteristics in oral squamous cell carcinoma [11]. In addition, high expression of NUDT1 in tumor tissues will lead to worse overall survival (OS) and progression-free survival (PFS) of colorectal cancer patients [12].

This study was to investigate the relationship between the expression of NUDT1 and the clinicopathological features and prognosis of RCC. In addition, we also explored the mechanism of NUDT1 in ccRCC and its relationship with immune infiltration.

## 2. Materials and Methods

### 2.1. Data Sources

RNA-seq count data and corresponding clinical feature information of ccRCC samples and adjacent tumor samples were obtained from the official website of The Cancer Genome Atlas (TCGA) (https://tcga-data.nci.nih.gov/tcga/). The inclusion criteria were set as follows: (1) patients were diagnosed as ccRCC; (2) patients had complete mRNA data and clinical information. According to the inclusion criteria, our study excluded any samples that had missing or insufficient data on grade, TNM stage, distant metastasis, and lymph node metastasis. RNA sequencing data were collated and converted into “symbol.txt” data for subsequent analysis. Since all of the data in this study were publicly available, there is no need for approval by the ethics committee.

### 2.2. NUDT1 Gene Expression Analysis

Tumor IMmune Estimation Resource (TIMER) (https://cistrome.shinyapps.io/timer/) is a comprehensive resource for systematical analysis of immune infiltrates across diverse cancer types, which included 10,897 samples of 32 cancer types from TCGA database [13]. We first analyzed NUDT1 expression level in Pan-cancer via gene modules. Subsequently, the TCGA-Kidney Clear Cell Carcinoma (KIRC) cohort was analyzed by using the R software limma package to explore the expression difference of NUDT1 between ccRCC group and control group, as well as the expression difference between ccRCC samples and adjacent tissues of its paired samples. In addition, we also evaluated NUDT1 expression on the basis of multiple clinicopathological features in ccRCC samples from TCGA. Boxplots using disease state as variable were graphed to calculate differential expression of NUDT1, Finally, the R software ggpubr package is used to visualize the results.

## 3. RNA Extraction and Quantitative Real-Time PCR

The resected tissues were immediately stored in liquid nitrogen. Total RNA was extracted using TRIzol reagent (Thermo Fisher Scientific, USA) from a total of 10 paired tumor and paracarcinoma normal samples. cDNA library was obtained using Servicebio® RT First Strand cDNA Synthesis Kit (Servicebio, Wuhan, China). qRT-PCR was performed using SYBR Green qPCR Master Mix (Servicebio, Wuhan, China). Glyceraldehyde-3-phosphate dehydrogenase (GAPDH) was set as internal control for gene quantification. The expression level of each gene was detected at least three times. The following primers were used: GAPDH forward 5′-GGAAGCTTGTCATCAATGGAAATC-3′ and reverse 5′-TGATGACCCTTTTGGCTCCC-3′; NUDT1 forward 5′-CAGATCGTGTTTGAGTTCGTGG-3′ and reverse 5′-AAGCAGGAGTGGAAACCAGTAG-3′. Relative quantification was calculated as 2^−ΔΔCt^.

### 3.1. Prognosis Analysis

Kaplan–Meier (K–M) survival analysis was performed to analyze the relationship between the expression of NUDT1 survival days of ccRCC patients. The expression level of NUDT1 mRNA in ccRCC was classified as low- or high-expression groups according to the median value. We used survminer R package to analyze the OS and PFS of low- or high-expression groups in ccRCC. Receiver operating characteristic (ROC) curve was used to evaluate the accuracy of the K–M survival analysis. Univariate Cox regression and multivariate Cox regression were used to evaluate the independent prognostic factors of patients with ccRCC. Nomogram was used to predict the survival of the patients. Finally, we used calibration curves to evaluate the predictive ability of nomograms.

### 3.2. Gene Coexpressed and Differentially Expressed Genes Analysis

R software was used to screen the coexpressed genes of NUDT1 by set the square of correlation coefficient |*R*^2^| > 0.6, *P* < 0.05 as the screening condition. The top-ranked genes were visualized by R software circlize package. Differentially expressed genes (DEGs) were divided into low- and high-expression groups and according to the expression of NUDT1 and |logFC| > 1, false discovery rate (FDR) <0.05 was considered to be significant DEG. The top 100 upregulated or downregulated DEGs were visualized by the pheatmap R package of R software. Gene Ontology (GO) function and Kyoto Encyclopedia of Genes and Genomes (KEGG) pathway enrichment analysis of DEGs were performed by R software clusterProfiler package, and visual analysis of data was performed by ggplot2 software package.

### 3.3. Immune Correlation Analysis

Based on TCGA-KIRC cohort, we explored the relationship between NUDT1 and cancer immune infiltrates at the mRNA level. CIBERSORT, an analytical tool developed by Newman et al. [14], can quantify the infiltrating immune cell fractions based on normalized gene expression profiles. We calculated the immune infiltration scores of 22 immune cell subtypes by CIBERSORT algorithm to explore the correlation between NUDT1 and immune cell infiltration. We then analyzed the correlation between NUDT1 and immune checkpoint molecules in ccRCC. Finally, we validated our analysis through the “correlation” module of the TIMER database.

### 3.4. Gene Set Enrichment Analysis

GSEA is a computational method that determines whether a previously defined set of genes have concordant and significant statistical differences in two biological states [15]. To gain more insight into the function of NUDT1, we performed GSEA on the high- and low-expression datasets of NUDT1 by using the R package “clusterProfiler.” Using GSEA, we analyzed GO terms and the KEGG pathways to investigate possible biological functions of NUDT1. Relevant gene pathways were selected according to the truncation criteria FDR < 0.25 and *P* < 0.05, and five related functional genomes were visualized.

### 3.5. Statistical Analysis

R (v.4.2.0) was used to perform statistical analysis. The impact of NUDT1 on the prognosis of ccRCC was evaluated using K–M and Cox regression analyses. ROC curve was utilized to evaluate the accuracy of K–M survival analysis. Spearman's analysis was used to explore the correlation between NUDT1 expression and immune cell infiltration level. All *P*-values were two-tailed, and *P* < 0.05 was deemed to be statistically significant. The intensity of *P*-value defined as follows:  ^*∗*^*P* < 0.05,  ^*∗∗*^*P* < 0.01, and  ^*∗∗∗*^*P* < 0.001.

## 4. Results

### 4.1. The Expression of NUDT1 Was Upregulated in Tumor Samples

**Figure 1:**
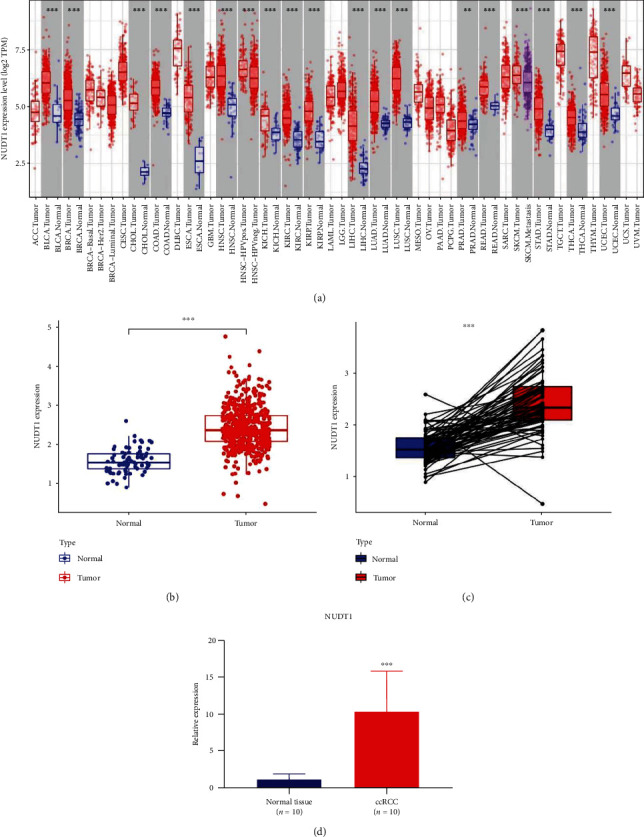
Differential expression analysis of NUDT1 in different samples: (a) NUDT1 expression in Pan-cancer data from TIMER database; (b) expression of NUDT1 in ccRCC and normal samples; (c) expression of NUDT1 in ccRCC and its paired adjacent normal tissues; (d) the qRT-PCR validation results of 10 ccRCC tissue and adjacent normal tissue samples.  ^*∗*^*P* < 0.05,  ^*∗∗*^*P* < 0.01,  ^*∗∗∗*^*P* < 0.001.

We first assessed NUDT1 expression in Pan-cancer data from TIMER database; the results showed that the expression of NUDT1 was upregulated in 18 tumors, including BCLA, BRCA, CHOL, COAD, ESCA, HNSC, KICH, KIRC, KIRP, LIHC, LUAD, LUSC, PRAD, READ, SKCM, STAD, THCA, and UCEC (Figure 1(a)). In addition, the analysis of TCGA-KIRC cohort showed that NUDT1 was highly expressed in ccRCC samples compared with normal samples (*P* < 0.001, Figure 1(b)). In paired specimens, the expression of NUDT1 in the ccRCC group was significantly higher than that found in the adjacent normal tissue (*P* < 0.001, Figure 1(c)). Finally, the difference in NUDT1 expression between ccRCC tissue and its adjacent normal tissue was validated by qRT-PCR (*P* < 0.001, Figure 1(d)).

### 4.2. NUDT1 Is Closely Related to the Clinical Features of ccRCC

**Figure 2:**
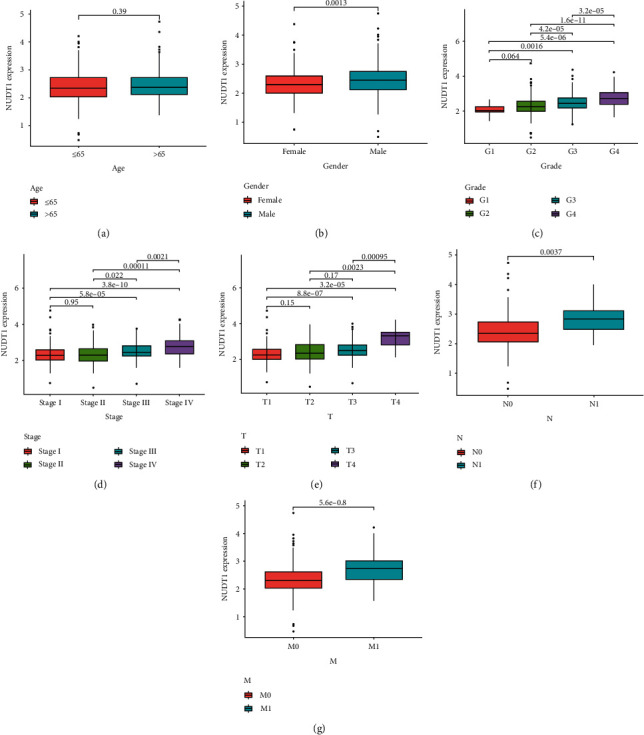
Evaluation and analysis of the clinicopathological features based on NUDT1 expression. The NUDT1 mRNA expression level was expressed for the patient clinicopathological features of (a) age, (b) gender, (c) grade, (d) stage, (e) T stage, (f) N stage, and (g) M stage.

In order to further explore the correlation between the expression of NUDT1 and the clinicopathological features of ccRCC, we extracted and collated the clinicopathological features of patients from TCGA-KIRC cohort (Table 1); the gender, age, grade, clinical stage, and T stage, N stage, and M stage were included. The results showed that in addition to age (Figure 2(a)), upregulated NUDT1 expression was significantly associated with gender (Figure 2(b)), grade (Figure 2(c)), clinical stage (Figure 2(d)), and T stage (Figure 2(e)), N stage (Figure 2(f)), and M stage (Figure 2(g)).

### 4.3. High NUDT1 Expression Is Associated with Adverse Outcomes in ccRCC

**Figure 3:**
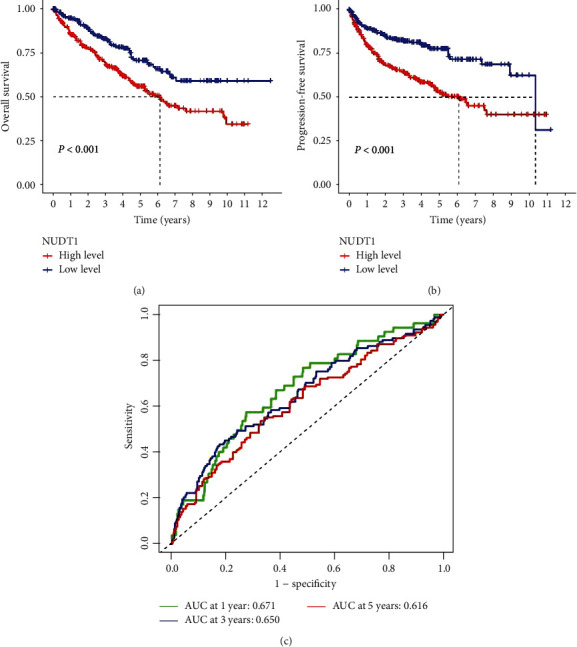
Evaluation of NUDT1 expression on survival of patients with ccRCC: (a) Kaplan–Meier analysis of overall survival based on NUDT1 expression; (b) Kaplan–Meier analysis of progression-free survival based on NUDT1 expression; (c) the areas under the ROC curve about 1, 3, and 5 years.

We further evaluated the value of NUDT1 in the prognosis of patients with ccRCC; K–M survival analysis showed that the OS in the high NUDT1 expression group was shorter than that in the low NUDT1 expression group (Figure 3(a)). Meanwhile, the expression of NUDT1 was negatively correlated with PFS in patients with ccRCC (Figure 3(b)). As shown in Figure 3(c), the ROC curve shows that the area under curve (AUC) corresponding to 1, 3, and 5 years was 0.671, 0.650, and 0.616, respectively. Generally, an AUC from 0.6 to 0.9 is deemed reliable.

**Figure 4:**
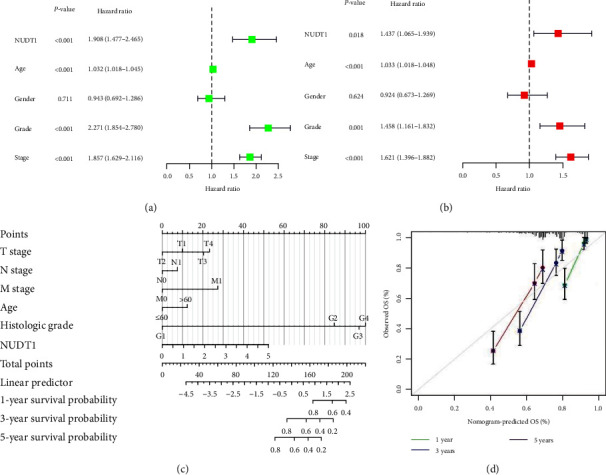
Prognosis evaluation of patients with ccRCC based on NUDT1: (a) the forest plots of univariate Cox regression analysis; (b) the forest plots of multivariate Cox regression analysis; (c) the nomogram based on NUDT1 and clinical characteristics; (d) the calibration curve of the nomogram.

We then used Cox regression analysis to evaluate the independent prognostic factors for ccRCC. Univariate analysis of correlation of using Cox regression revealed that NUDT1 is significantly associated with prognosis of patients with ccRCC (HR = 1.908, 95% CI: 1.477–2.465, *P* < 0.001, Figure 4(a), *Supplementary *1). In addition, the age, grade, and stage also significantly affect the prognosis of patients. We incorporated significant factors in univariate Cox regression analysis into multivariate Cox regression analysis; the results show that NUDT1 remained an independent prognostic factor (HR = 1.437, 95% CI: 1.065–1.939, *P*=0.018, Figure 4(b), Table 2). Finally, we constructed the nomogram containing NUDT1 and clinical characteristics to predict the survival of patients with ccRCC (Figure 4(c)). In the nomogram, we can calculate the total score according to the score of each clinical characteristics, and a higher total score was considered a worse the prognosis of the patient. The OS calibration chart of 1, 3, and 5 years indicates that the prediction effect of nomogram is satisfactory (Figure 4(d)).

### 4.4. Analysis of Gene Coexpressed and DEGs with NUDT1 in ccRCC

**Figure 5:**
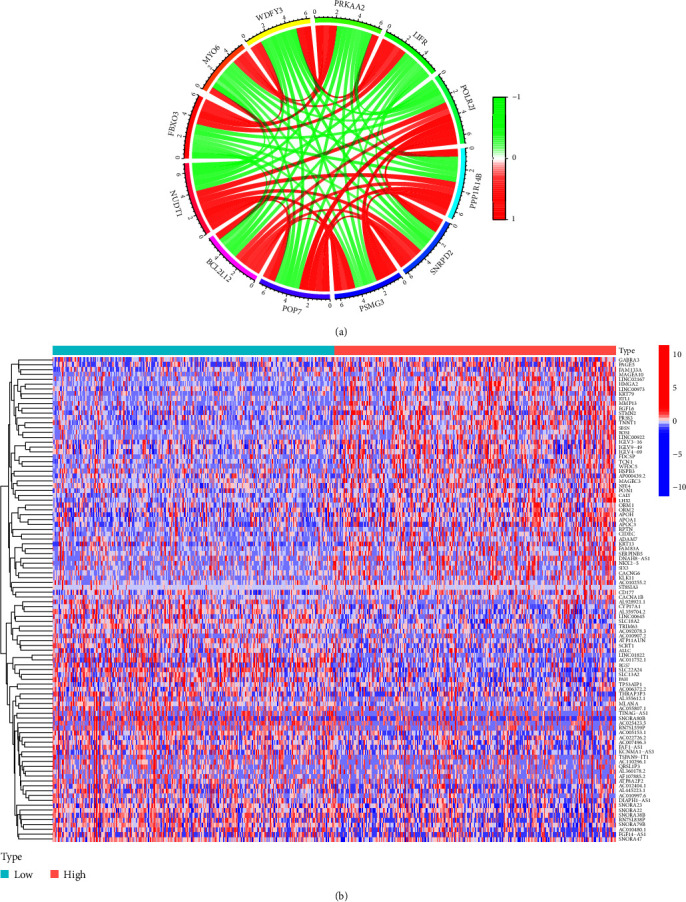
Analysis of coexpressed and differential expression of NUDT1: (a) the top 11 coexpressed genes related to NUDT1; (b) top 100 DEGs for NUDT1.

We analyzed the expression data files of NUDT1 mRNA to explore the coexpressed genes with NUDT1. According to our preset criteria, we found 150 coexpressed genes with NUDT1 (*Supplementary *2). As shown in the circos plot (Figure 5(a)), NUDT1 expression level was positively correlated with the expression of BCL2L12, POLR2J, PPP1R14B, SNRPD2, PSMG3, and POP7. On the contrary, NUDT1 expression level was negatively correlated with the expression of LIFR, PRKAA2, WDFY3, MYO6, and FBXO3. We then performed differential expression analysis of NUDT1 to find the DEGs in the high- and low-expression groups of NUDT1. A total of 1,886 DEGs were obtained, and we used heatmap to visualize the top 100 genes that were significantly upregulated or downregulated (*Supplementary *3, Figure 5(b)). According to the expression heatmap, we visually observed that the upper part of DEGs was overexpressed in the high-expression group and the lower part of DEGs was overexpressed in the low-expression group.

**Figure 6:**
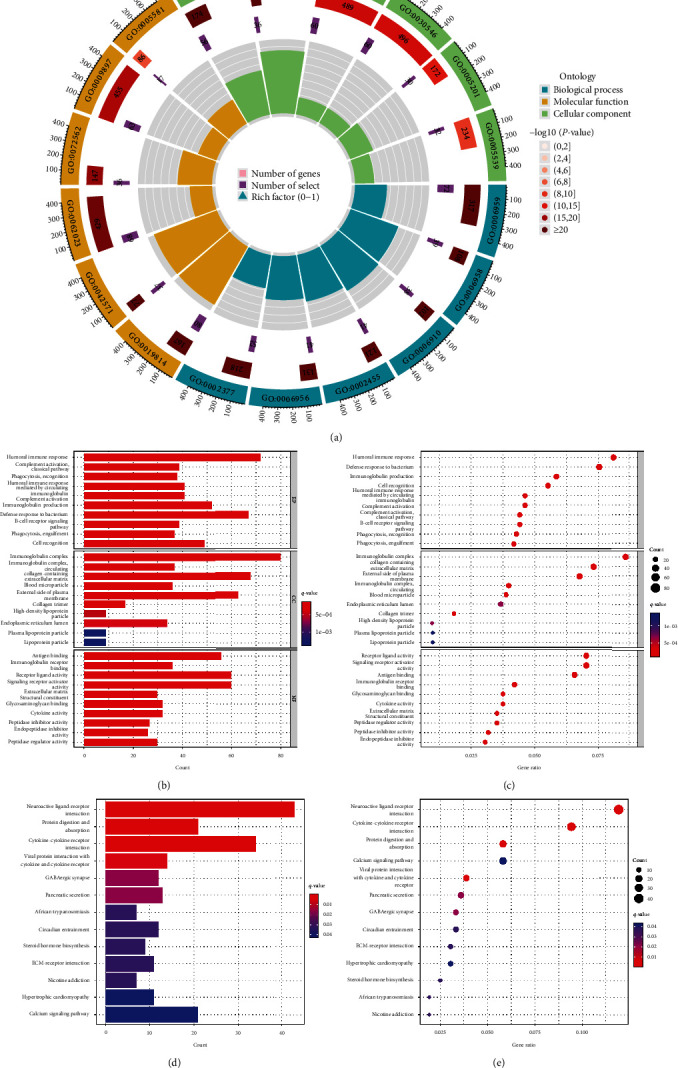
GO/KEGG enrichment analysis of DEGs between high- and low-NUDT1 expression: (a–c) GO enrichment analysis; (d, e) KEGG enrichment analysis.

Subsequently, we performed GO function and KEGG pathway enrichment analysis for these DEGs. Based on the screening criteria, there are 171 biological process (GO-BP), 29 cell component (GO-CC), 65 molecular function (GO-MF), and 13 KEGG were involved (Figure 6(a), *Supplementary *4 and 5). GO functional annotations showed that the DEGs were mainly enriched in humoral immune response, immunoglobulin complex, and receptor ligand activity (Figures 6(b) and 6(c)). KEGG pathway analysis showed that these genes were enriched in neuroactive ligand-receptor interaction, cytokine–cytokine receptor interaction, and protein digestion and absorption pathway (Figures 6(d) and 6(e)).

### 4.5. NUDT1 Expression Is Associated with the Immune Infiltration and Immune Checkpoints

**Figure 7:**
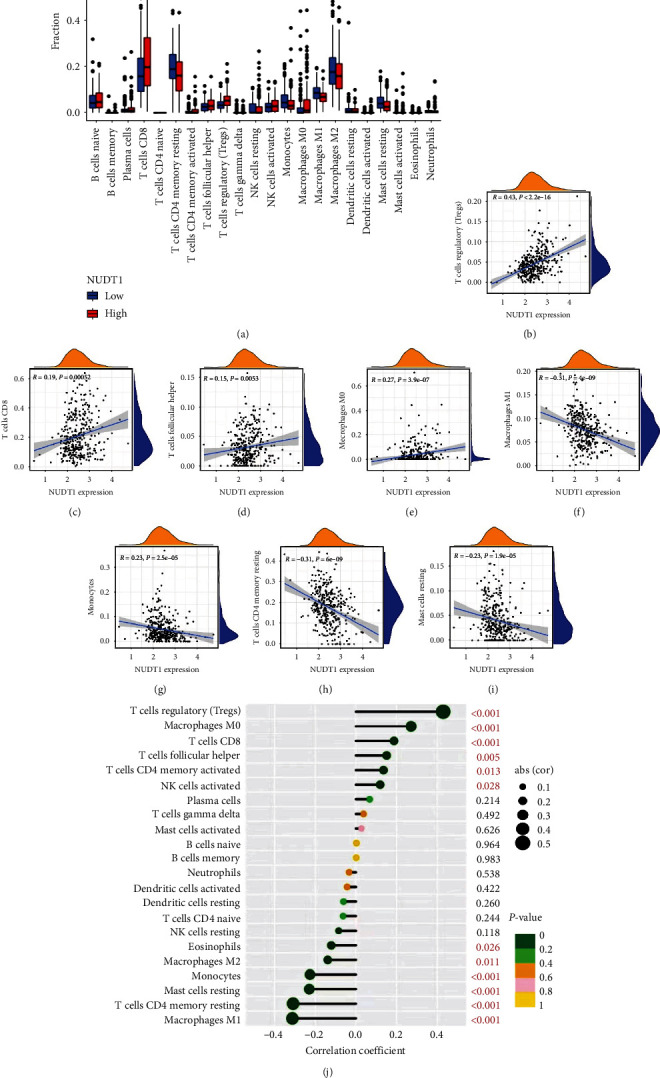
Correlation analysis between NUDT1 and immune infiltration: (a) the infiltration levels of 22 immune cell subtypes in the high- and low-NUDT1 expression group; (b–j) the correlation between tumor immune infiltrated cell levels and NUDT1 expression in ccRCC.  ^*∗*^*P* < 0.05,  ^*∗∗*^*P* < 0.01,  ^*∗∗∗*^*P* < 0.001.

Related studies have proved that tumor-infiltrating lymphocytes are independent predictors of the OS and sentinel lymph node status among cancer patients [16, 17]. Therefore, we tried to explore whether the expression of NUDT1 is related to immune cell infiltration in ccRCC. We analyzed the expression differences of 22 immune cell subtypes in high- and low-expression group of NUDT1 (Figure 7(a), *Supplementary *6). Notably, NUDT1 expression was positively and remarkably linked with the infiltrating levels of regulatory T cells (Tregs), CD8^+^ T cells, follicular helper T cells, and M0 macrophages, but negatively linked with the infiltrating levels of M1 macrophages, M2 macrophages, resting mast cells, resting memory CD4^+^ T cells, and monocytes in ccRCC (Figure 7(b)–7(j)).

**Figure 8:**
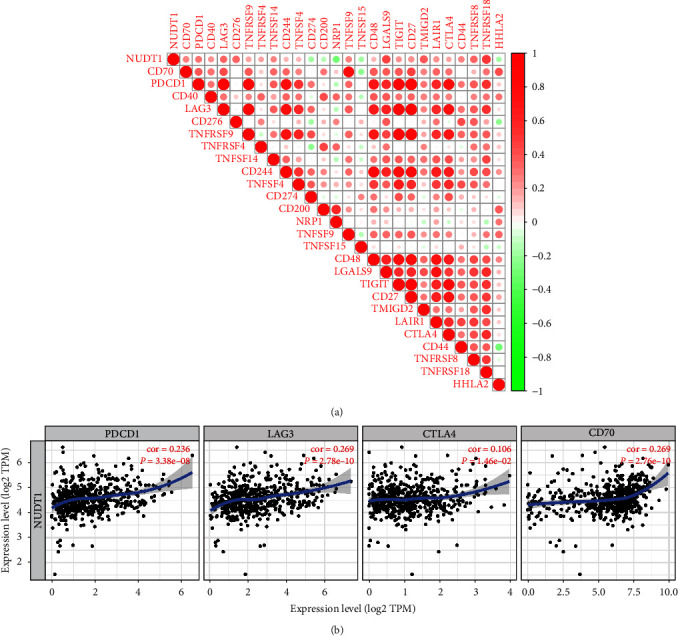
Correlation analysis between NUDT1 and immune checkpoints: (a) the correlation analysis of immune checkpoints based on TCGA-KIRC datasets; (b) the correlation analysis of immune checkpoints by using TIMER database.

We next explore the correlation between NUDT1 expression level and immune checkpoints through TCGA-KIRC cohort. The results are shown in Figure 8(a), (*Supplementary *7). NUDT1 expression level is positively correlated with immune checkpoints such as PD-1, LAG3, CTLA4, CD70, LGALS9, TMIGD2, and CD276. Up to now, PD-1, LAG3, CTLA4, and CD70 have been considered immune checkpoints strongly related to immunotherapy, so we verified the correlation between the expression level of NUDT1 and these four immune checkpoints again through the TIMER database. The results showed that NUDT1 was still positively correlated with PD-1 (cor = 0.236, *P*=3.38e − 08), LAG3 (cor = 0.269, *P*=2.78e − 10), CTLA4 (cor = 0.106, *P*=1.46e − 02), and CD70 (cor = 0.269, *P*=2.76e − 10) (Figure 8(b)). Our results suggest that the treatment of relevant immune checkpoint inhibitors may be helpful to improve the prognosis of patients with high NUDT1 expression group.

### 4.6. Gene Set Enrichment Analysis

**Figure 9:**
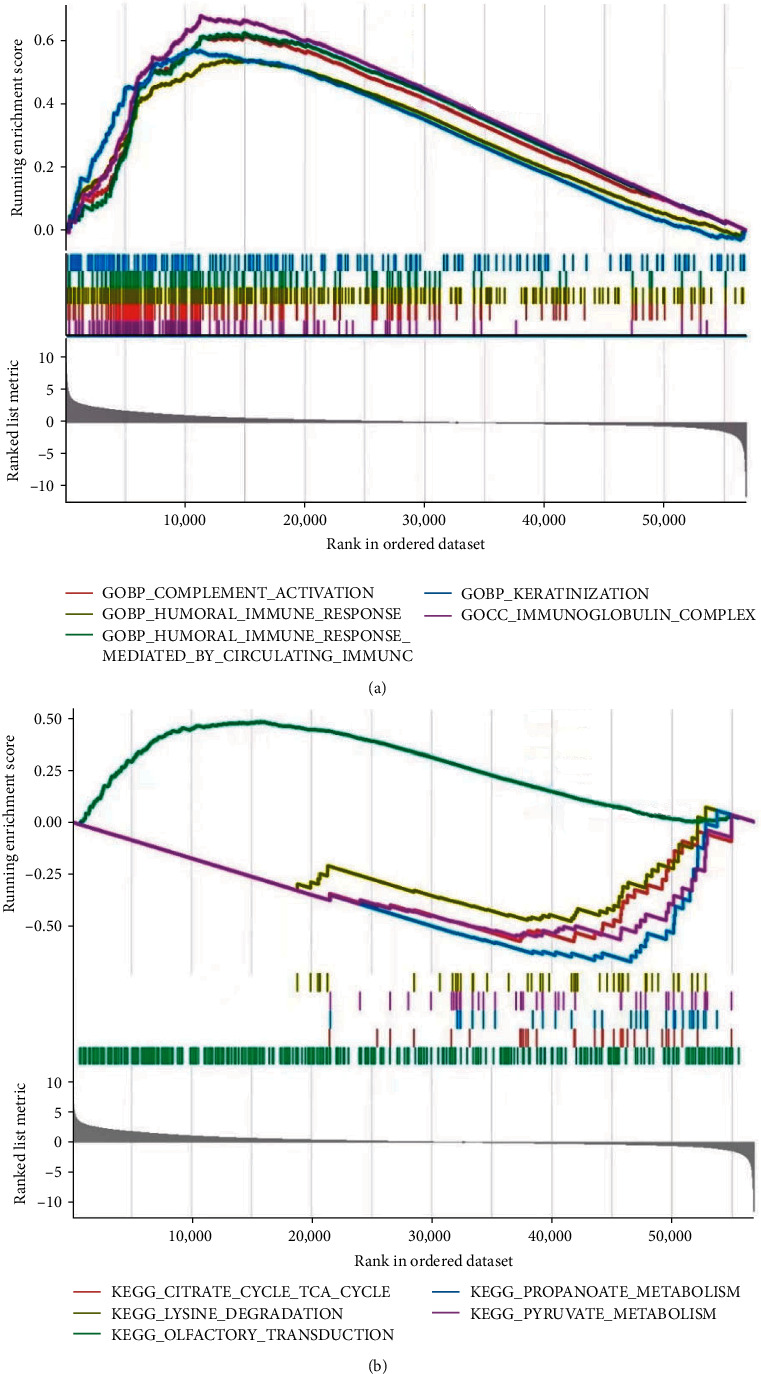
The results of functional analysis based on NUDT1: (a) GO enrichment analysis; (b) KEGG enrichment analysis.

In order to further recognize the potential function of NUDT1, we used GSEA to investigate the potential signaling pathways through which NUDT1 might affect ccRCC progression. In GO analysis, we found that some immune-related biological processes and cellular components were mainly enriched in the high-expression group of NUDT1 (Figure 9(a)). Complement activation, humoral immune response, or humoral immune response mediated by circulating immunoglobin, keratinization, and immunoglobulin complex were included. KEGG enrichment analysis showed that some metabolic-related pathways were significantly enriched in the low-expression group, such as propanoate metabolism, pyruvate metabolism, citrate cycle (tricarboxylic acid (TCA) cycle), and lysine degradation (Figure 9(b), Table 3). Interestingly, we also found that the pathways related to olfactory transduction were significantly enriched in the high-expression group.

## 5. Discussion

RCC is one of the most frequently occurring types of urological cancer and in recent years, its prevalence has continued to increase [18]. The most common histological type of RCC is clear cell type (70%–90%), followed by papillary (10%–15%) and chromophobe RCCs (3%–5%) [2]. At present, partial or radical nephrectomy is still the main treatment for early ccRCC [19]. With the deepening of cancer research, targeted therapy and immunotherapy stand out in the treatment of ccRCC, especially as a current and emerging first-line treatment for metastatic ccRCC [20]. The basis for the application of immunotherapy in ccRCC is that the tumor microenvironment (TME) of ccRCC has the characteristics of extensive immune infiltration, highly vascularized and fibrosis compared with other solid tumors [21]. Although the diagnosis and therapeutic strategies of ccRCC have improved significantly over the past decades, in view of the fact that the clinical symptoms of early ccRCC are not significant, while advanced ccRCC is often accompanied by distant metastasis, and the prognosis of patients with ccRCC is still poor [22, 23]. Abnormal gene expression may be involved in tumorigenesis and associated with the prognosis of patients [24]. Therefore, the exploration of new biomarkers is conducive to the early screening, diagnosis, and treatment of ccRCC.

As a member of the Nudix hydrolase superfamily, NUDT1 is 8,924 bp long and is located on chromosome 7 (2,242,222–2,251,145, in the GRCh38.p7-build of the human genome) [25]. The discovery of NUDT1 first aroused the interest of carcinogenesis investigators owing to its role in maintaining genomic stability, which is often compromised during cancer development. NUDT1 is capable of hydrolyzing the oxidized dNTPs and NTP, such as 8-oxo-dGTP and 2-OH-dATP, to their monophosphate form and prevent their incorporation into the nucleus and mitochondrial DNA, thereby limiting the ROS-induced cell damage [26, 27]. It indicates that NUDT1 plays an indispensable role in surviving the oxidative stress in cancer cells. However, NUDT1 may not be always indispensable for cancer cell survival under oxidative conditions. Several researchers have found that NUDT1 deficiency in certain cancer cell lines caused by small RNA interference or genome editing does not result in any adverse effect on these cells [28, 29]. Therefore, more researches were necessary to explore how NUDT1 affects the occurrence and development in oncogenesis.

In our current research, we explored the expression of NUDT1 as a prognostic biomarker for ccRCC. We systematically analyzed the prognostic significance of NUDT1 in ccRCC patients. A previous study showed that NUDT1 expression was correlated with the clinicopathological features, degree of vascular invasion, OS, and disease-free survival (DFS) in hepatocellular carcinoma (HCC) patients [30]. We used data on ccRCC patients obtained from TCGA to assess the prognostic value of NUDT1. Our study shows that the expression of NUDT1 is related to the prognosis of ccRCC. The expression of NUDT1 was significantly upregulated in ccRCC and correlated with adverse clinicopathological features such as gender, T stage, N stage, M stage, clinical stage, and pathological grade. The K–M curves reveal higher NUDT1 expression levels correlated with short OS and PFS in ccRCC patients. Cox proportional hazards regression model indicates that NUDT1 expression in tumor cells is an independent prognostic indicator of ccRCC. In the coexpression analysis, we found some genes that were positively correlated with NUDT1 expression, such as BCL2L12, POLR2J, PPP1R14B, SNRPD2, PSMG3, and POP7. BCL2L12 is an antiapoptosis factor, and it was discovered and cloned as a member of BCL2 family in 2001. The prognostic significance of BCL2L12 mRNA expression has already been assessed in several cancer types [31, 32]. BCL2L12 plays an important role in carcinogenesis by neutralizing effector caspase activity downstream of mitochondrial dysfunction and apoptosome activity to inhibit apoptosis [33]. POLR2J is a subunit of human nuclear RNA polymerase II [34]. By bioinformatics analysis, Wang et al. found that POLR2J, as a DNA repair gene, may be associated with poor prognosis of uveal melanoma (UM) patients [35]. A study shows that PPP1R14B is highly expressed in glioma and leads to bad outcome for patients [36]. It has been reported that SNRPD2 was overexpressed in HCC compared with adjacent normal tissues, and the expression level of SNRPD2 was significantly correlated with the pathologic stage of HCC [37]. As a chaperone protein, PSGM3 can affect the stability of the protein by assisting the assembly of proteasome. Ma et al.'s findings suggest that the interaction between *Anaplasma phagocytophilum* APTA and PSMG3 affects proteasome activity and ubiquitination process, activates the ubiquitin–proteasome system (UPS) pathway, and then couples with autophagy pathway, resulting in the antiapoptotic effect of APTA [38]. A recent study showed that POP7 can promote the progression of breast cancer by regulating the stability and expression of ILF3 mRNA [39]. In addition, the GO and KEGG function enrichment analysis of 1,886 DEGs significantly correlated with NUDT1 demonstrated that the DEGs were mainly related to humoral immune response, immunoglobulin complex, and receptor ligand activity. KEGG pathway analysis showed that the DEGs were primarily associated to neuroactive ligand-receptor interaction, cytokine–cytokine receptor interaction, and protein digestion and absorption pathway.

Another important aspect of this study is that NUDT1 expression is correlated with diverse immune infiltration levels in ccRCC. Our results demonstrate that there is a significantly positive correlations between NUDT1 expression level and infiltration level of Tregs cell, CD8^+^ T cells, follicular helper T cells, and M0 macrophages. At the same time, the expression level of NUDT1 is negatively linked with the infiltrating levels of M1 macrophages, M2 macrophages, resting mast cells, resting memory CD4^+^ T cells, and monocytes in ccRCC. Previous studies have shown that Treg cells are immune suppressor T cells, which can inhibit antitumor immune response by inhibiting CD8^+^ T cells, natural killer cells (NK cells), B cells, and antigen-presenting cells (APC) [40]. In a variety of cancers including melanoma, high-level Treg cells infiltration is related to tumor recurrence, progression, and metastasis [41]. According to our analysis, the upregulation of Treg cells in NUDT1 high-expression group may be an important factor leading to adverse OS in patients with ccRCC. In addition, some studies have shown that the reduction of immune infiltration in TME, especially CD4^+^ T cells and M1 macrophages, may lead to poor prognosis of cancer patients [42, 43]. Based on the analysis of immune infiltration, we infer that the high-expression group of NUDT1 is related to the decreased infiltration of monocytes and M1 macrophages, which leads to adverse outcomes in ccRCC patients. In addition, the correlation between NUDT1 expression and immune checkpoint marker implicates the role of NUDT1 in regulating tumor immunology in ccRCC. These novel findings have made substantial progress in identifying the important role of ccRCC in immune infiltration. Collectively, in the present study, we observed that the overexpression of NUDT1 was obviously linked to the poor prognosis in ccRCC patients. Furthermore, we found that NUDT1 expression had a positive association with immune infiltrates and immune checkpoints. These results suggest that NUDT1 plays a vital role in immune infiltration and immune escape in ccRCC, which has not been reported in previous studies.

## 6. Conclusion

NUDT1 plays a key role in the prognosis and immune cell infiltration of ccRCC patients, indicating its potential use as a prognostic biomarker and therapeutic target.

## Figures and Tables

**Table 1:** Clinicopathological features of patients with ccRCC.

Clinical characteristics	No. of cases	Percentage (%)
Gender		
Female	191	35.57
Male	346	64.43
Age (years)		
≤65	352	65.55
>65	185	34.45
Grade		
G1	14	2.65
G2	230	43.48
G3	207	39.13
G4	78	14.74
Clinical stage		
Ⅰ	269	50.37
Ⅱ	57	10.68
Ⅲ	125	23.41
Ⅳ	83	15.54
T stage		
T1	275	51.21
T2	69	12.85
T3	182	33.89
T4	11	2.05
N stage		
N0	240	93.39
N1	17	6.61
M stage		
M0	426	84.36
M1	79	15.64

**Table 2:** Multivariate Cox regression analysis of NUDT1 and clinical characteristics on prognosis of patients.

Variable	HR	95% CI	*P*-value
NUDT1	1.437	1.065–1.939	0.018
Age	1.033	1.018–1.048	<0.001
Gender	0.924	0.673–1.269	0.624
Grade	1.458	1.161–1.832	0.001
Stage	1.621	1.396–1.882	<0.001

**Table 3:** Gene sets enriched in the NUDT1 expression.

Name	NES	*P*-value	FDR
KEGG_OLFACTORY_TRANSDUCTION	1.494	0.001	0.176
KEGG_CITRATE_CYCLE_TCA_CYCLE	−1.916	0.007	0.228
KEGG_PROPANOATE_METABOLISM	−2.247	0.007	0.228
KEGG_PYRUVATE_METABOLISM	−1.945	0.009	0.228
KEGG_LYSINE_DEGRADATION	−1.662	0.009	0.228
KEGG_VALINE_LEUCINE_AND_ISOLEUCINE_DEGRADATION	−2.091	0.009	0.228
KEGG_INOSITOL_PHOSPHATE_METABOLISM	−1.645	0.010	0.228
KEGG_ENDOMETRIAL_CANCER	−1.697	0.011	0.228
KEGG_RIBOSOME	1.429	0.013	0.228
KEGG_ADIPOCYTOKINE_SIGNALING_PATHWAY	−1.830	0.013	0.228
KEGG_BUTANOATE_METABOLISM	−1.655	0.014	0.230
KEGG_DRUG_METABOLISM_CYTOCHROME_P450	1.421	0.016	0.236

Note: Gene sets with *P*-value < 0.05 and FDR < 0.25 were considered significant. NES, normalized enrichment score; FDR, false discovery rate.

## Data Availability

The datasets supporting the conclusion of this article are included within the article.

## Abbreviations

RCC: Renal cell carcinoma
ccRCC: Clear cell renal cell carcinoma
NUDT1: Nudix (nucleoside diphosphate-linked moiety X)-type motif 1
OS: Overall survival
PFS: Progression-free survival
DEGs: Differentially expressed genes
ROC curve: Receiver operating characteristic curve
AUC: The area under curve
GO: Gene Ontology
KEGG: Kyoto Encyclopedia of Genes and Genomes
GSEA: Gene set enrichment analysis
qRT-PCR: Quantitative real-time PCR.

## Supplementary Materials

Supplementary 1 Univariate multivariate Cox regression analysis of NUDT1 and clinical characteristics on prognosis of patients.

Supplementary 2 List of coexpression genes with NUDT1.

Supplementary 3 List of differentially expressed genes associated with NUDT1.

Supplementary 4 GO enrichment analysis of DEGs.

Supplementary 5 KEGG enrichment analysis of DEGs.

Supplementary 6 The correlation between 22 immune cell subtypes and NUDT1 expression level.

Supplementary 7 The correlation between immune checkpoints and NUDT1 expression level.
