# Supplementary material for: NUDT1 Could Be a Prognostic Biomarker and Correlated with Immune Infiltration in Clear Cell Renal Cell Carcinoma
Source: Appl Bionics Biomech. 2022 Dec 26;2022:3669296. doi: 10.1155/2022/3669296 (PMC9808898; doi:10.1155/2022/3669296)
Supplement: Supplementary 1 — Univariate multivariate Cox regression analysis of NUDT1 and clinical characteristics on prognosis of patients. [file 3669296.f1.docx]

Table. Univariate Multivariate Cox regression analysis of NUDT1 and clinical characteristics on prognosis of patients

|  | HR | 95%CI | pvalue |
| --- | --- | --- | --- |
| NUDT1 | 1.908 | 1.477-2.465 | ＜0.001 |
| Age | 1.032 | 1.018-1.045 | ＜0.001 |
| Gender | 0.943 | 0.692-1.286 | 0.711 |
| Grade | 2.271 | 1.854-2.780 | ＜0.001 |
| Stage | 1.857 | 1.629-2.116 | ＜0.001 |
