# Supplementary material for: NUDT1 Could Be a Prognostic Biomarker and Correlated with Immune Infiltration in Clear Cell Renal Cell Carcinoma
Source: Appl Bionics Biomech. 2022 Dec 26;2022:3669296. doi: 10.1155/2022/3669296 (PMC9808898; doi:10.1155/2022/3669296)
Supplement: Supplementary 2 — List of coexpression genes with NUDT1. [file 3669296.f2.docx]

List of co-expression genes with NUDT1

|  | Gene | cor | | pvalue |
| --- | --- | --- | --- | --- |
| NUDT1 | C9orf16 | 0.632273 | 9.79E-62 | |
| NUDT1 | RPS19 | 0.673998 | 6.63E-73 | |
| NUDT1 | RPUSD1 | 0.603698 | 5.04E-55 | |
| NUDT1 | METTL14 | -0.62119 | 4.75E-59 | |
| NUDT1 | BRAT1 | 0.611519 | 8.58E-57 | |
| NUDT1 | PPP1R14B | 0.718779 | 3.88E-87 | |
| NUDT1 | LAGE3 | 0.706626 | 5.22E-83 | |
| NUDT1 | PRKAA2 | -0.66032 | 4.79E-69 | |
| NUDT1 | UBE2C | 0.677015 | 8.75E-74 | |
| NUDT1 | RPL28 | 0.703268 | 6.62E-82 | |
| NUDT1 | COPS6 | 0.626206 | 2.98E-60 | |
| NUDT1 | UBA52 | 0.609183 | 2.93E-56 | |
| NUDT1 | ROMO1 | 0.659566 | 7.71E-69 | |
| NUDT1 | MAP2K2 | 0.613852 | 2.49E-57 | |
| NUDT1 | RPS15 | 0.658927 | 1.15E-68 | |
| NUDT1 | SBF2 | -0.60013 | 3.11E-54 | |
| NUDT1 | PSMD13 | 0.626354 | 2.74E-60 | |
| NUDT1 | PRPF31 | 0.616949 | 4.74E-58 | |
| NUDT1 | MYDGF | 0.60253 | 9.17E-55 | |
| NUDT1 | ADRM1 | 0.612976 | 3.96E-57 | |
| NUDT1 | ARPC4 | 0.612363 | 5.49E-57 | |
| NUDT1 | H2AX | 0.66546 | 1.79E-70 | |
| NUDT1 | CFL1 | 0.632726 | 7.56E-62 | |
| NUDT1 | EIF5A | 0.621869 | 3.27E-59 | |
| NUDT1 | TSPO | 0.640861 | 6.81E-64 | |
| NUDT1 | ZNHIT1 | 0.688013 | 4.42E-77 | |
| NUDT1 | SLK | -0.61422 | 2.04E-57 | |
| NUDT1 | SECISBP2L | -0.64727 | 1.50E-65 | |
| NUDT1 | ANAPC11 | 0.66018 | 5.23E-69 | |
| NUDT1 | GADD45GIP1 | 0.630729 | 2.35E-61 | |
| NUDT1 | ITPA | 0.637771 | 4.14E-63 | |
| NUDT1 | CENPM | 0.658928 | 1.15E-68 | |
| NUDT1 | TBCB | 0.640808 | 7.03E-64 | |
| NUDT1 | SH3BGRL3 | 0.607378 | 7.52E-56 | |
| NUDT1 | GPX1 | 0.607294 | 7.85E-56 | |
| NUDT1 | APOOL | -0.61106 | 1.09E-56 | |
| NUDT1 | USP12 | -0.61925 | 1.37E-58 | |
| NUDT1 | LGALS1 | 0.643846 | 1.17E-64 | |
| NUDT1 | JPT1 | 0.661565 | 2.17E-69 | |
| NUDT1 | RPL27A | 0.641988 | 3.51E-64 | |
| NUDT1 | AP1G1 | -0.60779 | 6.06E-56 | |
| NUDT1 | ENPP4 | -0.63303 | 6.37E-62 | |
| NUDT1 | ATP5MC2 | 0.601946 | 1.24E-54 | |
| NUDT1 | SNRPF | 0.602931 | 7.47E-55 | |
| NUDT1 | RUVBL2 | 0.647681 | 1.17E-65 | |
| NUDT1 | SNRPA | 0.645114 | 5.48E-65 | |
| NUDT1 | SEM1 | 0.67239 | 1.93E-72 | |
| NUDT1 | RPL13A | 0.601889 | 1.27E-54 | |
| NUDT1 | RAB11FIP2 | -0.61627 | 6.81E-58 | |
| NUDT1 | PTTG1 | 0.687716 | 5.45E-77 | |
| NUDT1 | FAM174C | 0.629099 | 5.90E-61 | |
| NUDT1 | RPL13 | 0.61535 | 1.12E-57 | |
| NUDT1 | CLTB | 0.637779 | 4.12E-63 | |
| NUDT1 | JOSD2 | 0.676789 | 1.02E-73 | |
| NUDT1 | SLC52A2 | 0.661739 | 1.95E-69 | |
| NUDT1 | BRMS1 | 0.612338 | 5.56E-57 | |
| NUDT1 | ZMYND11 | -0.61966 | 1.09E-58 | |
| NUDT1 | STX10 | 0.638548 | 2.64E-63 | |
| NUDT1 | ZNF787 | 0.605828 | 1.68E-55 | |
| NUDT1 | BANF1 | 0.623417 | 1.40E-59 | |
| NUDT1 | NSUN5 | 0.682132 | 2.67E-75 | |
| NUDT1 | MEGF9 | -0.60165 | 1.44E-54 | |
| NUDT1 | C7orf50 | 0.637096 | 6.13E-63 | |
| NUDT1 | RALY | 0.702925 | 8.57E-82 | |
| NUDT1 | POP7 | 0.731531 | 1.04E-91 | |
| NUDT1 | FNBP1L | -0.62744 | 1.50E-60 | |
| NUDT1 | SRM | 0.619734 | 1.05E-58 | |
| NUDT1 | RNF38 | -0.61464 | 1.64E-57 | |
| NUDT1 | ZBTB44 | -0.60339 | 5.91E-55 | |
| NUDT1 | PKN2 | -0.61757 | 3.39E-58 | |
| NUDT1 | TMUB1 | 0.656834 | 4.28E-68 | |
| NUDT1 | SNRPD2 | 0.720895 | 7.04E-88 | |
| NUDT1 | SNRPB | 0.665184 | 2.14E-70 | |
| NUDT1 | MRPS34 | 0.603964 | 4.40E-55 | |
| NUDT1 | SESTD1 | -0.6396 | 1.43E-63 | |
| NUDT1 | RPL38 | 0.608262 | 4.74E-56 | |
| NUDT1 | RPLP2 | 0.680102 | 1.07E-74 | |
| NUDT1 | LAMTOR4 | 0.646569 | 2.29E-65 | |
| NUDT1 | DGUOK | 0.677934 | 4.70E-74 | |
| NUDT1 | NOC4L | 0.616849 | 5.00E-58 | |
| NUDT1 | POLR2J | 0.71813 | 6.53E-87 | |
| NUDT1 | YIF1B | 0.652521 | 6.18E-67 | |
| NUDT1 | SAMD1 | 0.604856 | 2.78E-55 | |
| NUDT1 | RPS2 | 0.651137 | 1.44E-66 | |
| NUDT1 | C19orf53 | 0.651833 | 9.43E-67 | |
| NUDT1 | RPLP1 | 0.624144 | 9.35E-60 | |
| NUDT1 | ARHGEF12 | -0.624 | 1.01E-59 | |
| NUDT1 | SPOPL | -0.62058 | 6.63E-59 | |
| NUDT1 | PSMG3 | 0.729476 | 5.88E-91 | |
| NUDT1 | MYO6 | -0.666 | 1.27E-70 | |
| NUDT1 | DNAJC16 | -0.60055 | 2.52E-54 | |
| NUDT1 | BCL2L12 | 0.735761 | 2.75E-93 | |
| NUDT1 | BOP1 | 0.656869 | 4.19E-68 | |
| NUDT1 | MYBL2 | 0.622217 | 2.70E-59 | |
| NUDT1 | TFPT | 0.602929 | 7.47E-55 | |
| NUDT1 | PIK3C2A | -0.636 | 1.16E-62 | |
| NUDT1 | UBE2S | 0.717454 | 1.12E-86 | |
| NUDT1 | WDFY3 | -0.66429 | 3.81E-70 | |
| NUDT1 | PCCA | -0.61218 | 6.06E-57 | |
| NUDT1 | NME1 | 0.616396 | 6.38E-58 | |
| NUDT1 | MYO9A | -0.60628 | 1.33E-55 | |
| NUDT1 | PPM1A | -0.6138 | 2.56E-57 | |
| NUDT1 | NPM3 | 0.60918 | 2.93E-56 | |
| NUDT1 | CDC20 | 0.629409 | 4.95E-61 | |
| NUDT1 | RPL36 | 0.609781 | 2.14E-56 | |
| NUDT1 | LIFR | -0.6554 | 1.04E-67 | |
| NUDT1 | ELOB | 0.6238 | 1.13E-59 | |
| NUDT1 | ZC3H12C | -0.60773 | 6.27E-56 | |
| NUDT1 | RAPGEF2 | -0.61548 | 1.04E-57 | |
| NUDT1 | CCDC186 | -0.64932 | 4.36E-66 | |
| NUDT1 | RPL29 | 0.602279 | 1.04E-54 | |
| NUDT1 | AURKB | 0.621181 | 4.77E-59 | |
| NUDT1 | PDAP1 | 0.608137 | 5.06E-56 | |
| NUDT1 | CDK5 | 0.604018 | 4.27E-55 | |
| NUDT1 | BIRC5 | 0.63716 | 5.91E-63 | |
| NUDT1 | PTAR1 | -0.63665 | 7.94E-63 | |
| NUDT1 | ELOF1 | 0.614323 | 1.94E-57 | |
| NUDT1 | PYCARD | 0.621908 | 3.20E-59 | |
| NUDT1 | PSMB3 | 0.658888 | 1.18E-68 | |
| NUDT1 | POC1A | 0.602373 | 9.94E-55 | |
| NUDT1 | CCM2 | 0.712461 | 5.78E-85 | |
| NUDT1 | EPB41L5 | -0.6142 | 2.06E-57 | |
| NUDT1 | DTX2 | 0.613362 | 3.23E-57 | |
| NUDT1 | PGLS | 0.686456 | 1.32E-76 | |
| NUDT1 | SPC24 | 0.656193 | 6.39E-68 | |
| NUDT1 | SAC3D1 | 0.670316 | 7.59E-72 | |
| NUDT1 | DNTTIP1 | 0.601978 | 1.22E-54 | |
| NUDT1 | FBXO3 | -0.67709 | 8.29E-74 | |
| NUDT1 | ACADSB | -0.63819 | 3.25E-63 | |
| NUDT1 | GFUS | 0.602318 | 1.02E-54 | |
| NUDT1 | HSPB1 | 0.607864 | 5.84E-56 | |
| NUDT1 | LAMTOR2 | 0.61188 | 7.09E-57 | |
| NUDT1 | DNAJC27 | -0.60795 | 5.57E-56 | |
| NUDT1 | SEC24B | -0.63163 | 1.41E-61 | |
| NUDT1 | PAFAH1B3 | 0.626313 | 2.81E-60 | |
| NUDT1 | VWA8 | -0.61609 | 7.52E-58 | |
| NUDT1 | RPL37 | 0.605285 | 2.23E-55 | |
| NUDT1 | LSM7 | 0.669851 | 1.03E-71 | |
| NUDT1 | DRAP1 | 0.68456 | 4.96E-76 | |
| NUDT1 | MAPK8 | -0.61203 | 6.53E-57 | |
| NUDT1 | RPS20 | 0.628459 | 8.45E-61 | |
| NUDT1 | MIIP | 0.633106 | 6.09E-62 | |
| NUDT1 | TWF2 | 0.672426 | 1.88E-72 | |
| NUDT1 | PPP1R14BP3 | 0.68129 | 4.76E-75 | |
| NUDT1 | TMSB10 | 0.642536 | 2.54E-64 | |
| NUDT1 | HSPA4L | -0.64311 | 1.81E-64 | |
| NUDT1 | CHIC1 | -0.62821 | 9.75E-61 | |
| NUDT1 | AP2S1 | 0.685124 | 3.35E-76 | |
| NUDT1 | DTYMK | 0.629294 | 5.29E-61 | |
| NUDT1 | CENPW | 0.704965 | 1.84E-82 | |
