# Supplementary material for: NUDT1 Could Be a Prognostic Biomarker and Correlated with Immune Infiltration in Clear Cell Renal Cell Carcinoma
Source: Appl Bionics Biomech. 2022 Dec 26;2022:3669296. doi: 10.1155/2022/3669296 (PMC9808898; doi:10.1155/2022/3669296)
Supplement: Supplementary 3 — List of differentially expressed genes associated with NUDT1. [file 3669296.f3.docx]

List of differentially expressed genes associated with NUDT1

| gene | lowMean | highMean | logFC | pValue | fdr |
| --- | --- | --- | --- | --- | --- |
| MAGEL2 | 0.048932 | 0.127766 | 1.384662 | 2.45E-10 | 1.32E-09 |
| AC110285.3 | 0.029178 | 0.103953 | 1.832994 | 0.000449 | 0.000956 |
| SNORA22 | 1.37701 | 0.399218 | -1.78629 | 0.032525 | 0.047198 |
| SLC25A24P1 | 0.016669 | 0.036352 | 1.124865 | 0.000824 | 0.001665 |
| AC093895.1 | 0.053377 | 0.120262 | 1.171881 | 5.91E-08 | 2.31E-07 |
| AC108752.1 | 0.574366 | 1.523094 | 1.406962 | 0.029568 | 0.043358 |
| SPACA3 | 0.016288 | 0.10201 | 2.646834 | 1.42E-07 | 5.26E-07 |
| SYT5 | 0.03059 | 0.127771 | 2.062439 | 3.05E-06 | 9.36E-06 |
| TMEM119 | 3.320406 | 6.926335 | 1.060732 | 2.38E-08 | 9.75E-08 |
| PPIAL4C | 0.019976 | 0.040852 | 1.032153 | 8.86E-06 | 2.53E-05 |
| AC096734.1 | 0.020869 | 0.064628 | 1.630825 | 0.000302 | 0.000664 |
| GRIN1 | 0.008926 | 0.03141 | 1.815158 | 0.001017 | 0.002018 |
| CLDN6 | 0.042057 | 0.224364 | 2.415428 | 0.000225 | 0.000506 |
| IRF6 | 7.269916 | 3.550583 | -1.03388 | 5.74E-20 | 1.06E-18 |
| HAS1 | 0.092406 | 0.213732 | 1.209743 | 6.56E-07 | 2.22E-06 |
| FYTTD1P1 | 0.055741 | 0.019539 | -1.51239 | 2.25E-06 | 7.04E-06 |
| PODNL1 | 0.399536 | 1.196679 | 1.58264 | 6.49E-15 | 6.09E-14 |
| CPNE7 | 0.697057 | 1.920896 | 1.462431 | 4.08E-14 | 3.47E-13 |
| IGFBP1 | 14.4109 | 30.16799 | 1.065858 | 2.94E-05 | 7.69E-05 |
| RNASE2 | 1.484357 | 3.049483 | 1.038727 | 8.70E-16 | 9.16E-15 |
| LINC02240 | 0.0063 | 0.014756 | 1.227857 | 0.005066 | 0.00877 |
| PLPPR1 | 0.605554 | 0.264716 | -1.19381 | 0.000757 | 0.001539 |
| MLXP1 | 0.108588 | 0.485712 | 2.161229 | 4.53E-09 | 2.05E-08 |
| LINC02265 | 0.016949 | 0.067932 | 2.002909 | 0.000715 | 0.001462 |
| CKM | 0.075849 | 0.214728 | 1.501299 | 3.68E-09 | 1.68E-08 |
| FSD1 | 0.084069 | 0.202995 | 1.271791 | 5.37E-12 | 3.52E-11 |
| OR2T11 | 0.060295 | 0.023423 | -1.36411 | 6.97E-06 | 2.02E-05 |
| FST | 1.101084 | 2.494254 | 1.179684 | 1.46E-06 | 4.71E-06 |
| CA4 | 6.083097 | 2.521303 | -1.27064 | 9.72E-17 | 1.16E-15 |
| LHX2 | 0.040305 | 0.428876 | 3.411527 | 1.34E-10 | 7.40E-10 |
| IFNG | 0.489777 | 1.143605 | 1.223392 | 7.91E-07 | 2.65E-06 |
| ETV3L | 0.015575 | 0.040344 | 1.373133 | 0.00056 | 0.001173 |
| AC122134.1 | 0.380388 | 0.816124 | 1.101318 | 1.14E-06 | 3.71E-06 |
| AC116456.2 | 0.015622 | 0.007674 | -1.02547 | 0.000153 | 0.000355 |
| AC104779.1 | 0.496771 | 0.226016 | -1.13616 | 3.27E-16 | 3.64E-15 |
| SLC35G5 | 0.049764 | 0.10581 | 1.08829 | 0.003411 | 0.006117 |
| IGKV2-24 | 6.350302 | 26.83607 | 2.079276 | 0.000383 | 0.000827 |
| AC107208.1 | 0.024781 | 0.058998 | 1.251399 | 6.09E-08 | 2.37E-07 |
| AHSP | 0.292072 | 1.367051 | 2.226672 | 0.000725 | 0.00148 |
| AL079338.1 | 1.417671 | 3.133208 | 1.144118 | 0.000219 | 0.000493 |
| CDK6-AS1 | 0.095365 | 0.27889 | 1.548171 | 1.08E-06 | 3.55E-06 |
| COPDA1 | 0.054889 | 0.4076 | 2.892571 | 3.52E-05 | 9.10E-05 |
| TRPV3 | 0.068692 | 0.14262 | 1.053949 | 3.12E-06 | 9.59E-06 |
| CRABP1 | 0.726863 | 3.915757 | 2.429535 | 3.36E-06 | 1.03E-05 |
| PDCD1 | 1.834825 | 3.866586 | 1.075418 | 2.98E-09 | 1.38E-08 |
| ILDR2 | 1.196383 | 0.597343 | -1.00205 | 4.74E-13 | 3.55E-12 |
| ZDHHC19 | 0.065999 | 0.136991 | 1.053562 | 6.60E-09 | 2.91E-08 |
| RGS20 | 0.164952 | 0.386208 | 1.227334 | 8.38E-14 | 6.88E-13 |
| AC097493.3 | 0.398901 | 0.838168 | 1.071209 | 5.62E-11 | 3.26E-10 |
| LINC01971 | 0.010655 | 0.025718 | 1.2713 | 3.29E-06 | 1.00E-05 |
| Z93403.1 | 0.104401 | 0.041655 | -1.32557 | 0.002672 | 0.00488 |
| SNORA23 | 6.19154 | 0.271864 | -4.50934 | 0.019 | 0.029068 |
| GPR84 | 0.429437 | 0.930333 | 1.115301 | 2.21E-14 | 1.94E-13 |
| MELTF | 0.704756 | 1.641179 | 1.219536 | 5.23E-23 | 1.48E-21 |
| EN2 | 0.090984 | 0.246162 | 1.435928 | 5.52E-07 | 1.89E-06 |
| LINC02043 | 0.022799 | 0.062496 | 1.454818 | 0.006864 | 0.011553 |
| B3GALT5 | 0.260517 | 0.711182 | 1.448839 | 7.10E-11 | 4.06E-10 |
| CIR1P2 | 0.051912 | 0.021145 | -1.29575 | 2.27E-07 | 8.18E-07 |
| AC021127.1 | 0.065987 | 0.134692 | 1.029403 | 0.001153 | 0.002263 |
| AL008638.6 | 0.00612 | 0.013742 | 1.166929 | 0.00917 | 0.015062 |
| AP000251.1 | 0.030913 | 0.068729 | 1.152695 | 4.69E-05 | 0.000119 |
| PRLR | 2.069487 | 0.907956 | -1.18858 | 7.68E-09 | 3.36E-08 |
| MDK | 15.09936 | 36.46606 | 1.272067 | 2.61E-18 | 3.85E-17 |
| ARHGEF26-AS1 | 0.010312 | 0.023453 | 1.185406 | 0.007402 | 0.012374 |
| RN7SL338P | 0.177854 | 0.074824 | -1.24912 | 0.00063 | 0.001306 |
| TDO2 | 0.419592 | 0.910931 | 1.118356 | 0.002577 | 0.00472 |
| SCGB3A2 | 0.472958 | 1.011731 | 1.097041 | 2.28E-06 | 7.13E-06 |
| ERFE | 0.29117 | 1.123184 | 1.947661 | 1.47E-07 | 5.43E-07 |
| IL1R2 | 3.560999 | 14.63406 | 2.038976 | 2.72E-11 | 1.64E-10 |
| ITM2BP1 | 0.338336 | 0.160557 | -1.07537 | 4.82E-15 | 4.60E-14 |
| SFRP2 | 18.38544 | 38.51478 | 1.066848 | 1.76E-07 | 6.44E-07 |
| ROS1 | 0.011074 | 0.151666 | 3.775591 | 2.82E-06 | 8.69E-06 |
| FCRL5 | 0.161345 | 0.445189 | 1.46427 | 1.45E-05 | 3.99E-05 |
| TRBC2 | 25.89841 | 53.95666 | 1.058937 | 4.64E-14 | 3.91E-13 |
| AC005262.1 | 0.109335 | 0.441095 | 2.012327 | 0.022425 | 0.033727 |
| AL450345.2 | 0.037485 | 0.07985 | 1.090978 | 2.68E-05 | 7.06E-05 |
| KRT39 | 0.017686 | 0.038897 | 1.137032 | 5.67E-05 | 0.000141 |
| AC007040.2 | 0.030414 | 0.069188 | 1.185787 | 0.002623 | 0.004799 |
| AC008379.1 | 0.091971 | 0.040824 | -1.17176 | 0.00284 | 0.005164 |
| MIXL1 | 0.076033 | 0.178914 | 1.23457 | 2.71E-09 | 1.26E-08 |
| IGKV2-29 | 1.153068 | 5.66632 | 2.296934 | 0.008571 | 0.014154 |
| KCTD8 | 0.045509 | 0.018754 | -1.27895 | 0.000325 | 0.00071 |
| UBE2C | 2.62969 | 10.34262 | 1.975637 | 3.45E-39 | 3.90E-36 |
| CACNG7 | 0.011518 | 0.031343 | 1.444257 | 0.01481 | 0.023221 |
| DIAPH1-AS1 | 0.086755 | 0.024567 | -1.82025 | 0.007292 | 0.012212 |
| AC012378.2 | 0.047728 | 0.104227 | 1.12683 | 7.60E-05 | 0.000185 |
| WNT7B | 0.061765 | 0.461726 | 2.902173 | 2.61E-09 | 1.22E-08 |
| RPS8P4 | 0.07176 | 0.163415 | 1.187282 | 0.000997 | 0.001982 |
| AC104964.2 | 0.096565 | 0.04534 | -1.09071 | 1.14E-16 | 1.34E-15 |
| C1orf87 | 0.00773 | 0.019892 | 1.363685 | 0.001695 | 0.003219 |
| PRTN3 | 0.165319 | 0.409358 | 1.308115 | 0.008164 | 0.013538 |
| AC023043.1 | 0.282617 | 0.590454 | 1.062976 | 1.04E-17 | 1.42E-16 |
| RPS3AP25 | 0.168191 | 0.341361 | 1.021195 | 3.29E-05 | 8.53E-05 |
| AC093520.1 | 0.034688 | 0.075045 | 1.113313 | 3.35E-07 | 1.18E-06 |
| IGHV4-59 | 14.45306 | 54.24181 | 1.90803 | 2.72E-05 | 7.14E-05 |
| EPHA8 | 0.011561 | 0.035426 | 1.61556 | 4.54E-05 | 0.000115 |
| AL158828.1 | 0.007795 | 0.017652 | 1.179257 | 1.57E-06 | 5.04E-06 |
| MTRNR2L3 | 0.115072 | 0.295267 | 1.359482 | 0.002293 | 0.004241 |
| SLC22A13 | 0.856518 | 0.369361 | -1.21345 | 2.87E-13 | 2.21E-12 |
| CDKN2A | 1.236303 | 2.537522 | 1.037388 | 3.85E-12 | 2.56E-11 |
| LKAAEAR1 | 0.032463 | 0.07877 | 1.278847 | 0.000695 | 0.001426 |
| IGLV2-23 | 52.61039 | 117.6666 | 1.161285 | 1.27E-05 | 3.53E-05 |
| CGA | 0.222078 | 0.697153 | 1.650405 | 0.011495 | 0.018472 |
| FNDC4 | 1.936933 | 4.018438 | 1.052861 | 3.52E-11 | 2.10E-10 |
| TSPAN9-IT1 | 0.090152 | 0.024908 | -1.85574 | 0.003044 | 0.005501 |
| AC084026.2 | 0.103104 | 0.274711 | 1.413812 | 6.87E-11 | 3.94E-10 |
| IGHV3-69-1 | 0.890873 | 2.400106 | 1.429807 | 0.000202 | 0.000457 |
| KIF18B | 0.423554 | 1.077642 | 1.347262 | 1.53E-17 | 2.03E-16 |
| IL31RA | 0.040607 | 0.119474 | 1.55691 | 1.97E-07 | 7.16E-07 |
| LINC01276 | 0.009045 | 0.018788 | 1.05452 | 0.001328 | 0.002579 |
| OR52B3P | 0.049208 | 0.109683 | 1.156386 | 0.000124 | 0.00029 |
| IGLV1-36 | 2.630624 | 6.10949 | 1.215647 | 1.47E-06 | 4.74E-06 |
| OR2AT4 | 0.012297 | 0.036909 | 1.585705 | 0.000367 | 0.000794 |
| AL356417.2 | 0.047829 | 0.13595 | 1.507119 | 2.24E-11 | 1.37E-10 |
| ATP5F1EP2 | 0.784886 | 1.571014 | 1.001141 | 6.93E-15 | 6.47E-14 |
| EPYC | 0.058607 | 0.4083 | 2.800483 | 8.03E-06 | 2.31E-05 |
| UCA1 | 0.01666 | 0.052122 | 1.64548 | 0.002449 | 0.004505 |
| CHGB | 0.314314 | 0.871594 | 1.471448 | 1.73E-05 | 4.69E-05 |
| HEPACAM | 0.012141 | 0.038473 | 1.663921 | 0.027954 | 0.041171 |
| LINC02511 | 0.247709 | 0.559162 | 1.17462 | 0.001569 | 0.002999 |
| GALR1 | 0.20285 | 0.088362 | -1.19891 | 5.48E-09 | 2.45E-08 |
| DPYSL5 | 0.024764 | 0.126434 | 2.352079 | 3.81E-05 | 9.78E-05 |
| RBFOX3 | 0.016446 | 0.057047 | 1.794452 | 0.009933 | 0.016195 |
| MOCOS | 0.890163 | 1.892905 | 1.08846 | 1.29E-14 | 1.16E-13 |
| JAKMIP2 | 0.072436 | 0.160803 | 1.150525 | 5.85E-10 | 3.00E-09 |
| ADAMTS14 | 0.430196 | 0.946532 | 1.137659 | 8.56E-14 | 7.02E-13 |
| LINC01812 | 0.01966 | 0.069325 | 1.81813 | 9.49E-05 | 0.000228 |
| AC138904.1 | 0.019529 | 0.047883 | 1.293881 | 0.000228 | 0.000513 |
| AL121899.1 | 0.152917 | 0.353409 | 1.208595 | 6.71E-14 | 5.57E-13 |
| AC089983.1 | 0.030478 | 0.105182 | 1.787073 | 2.80E-07 | 9.96E-07 |
| AC092490.1 | 0.111889 | 0.24145 | 1.109656 | 0.014949 | 0.023416 |
| RPL5P14 | 0.07142 | 0.033541 | -1.09043 | 1.07E-08 | 4.58E-08 |
| C6orf141 | 0.282201 | 0.772318 | 1.452472 | 8.18E-09 | 3.56E-08 |
| CYP4F23P | 0.020619 | 0.123903 | 2.587179 | 1.54E-05 | 4.21E-05 |
| AC022167.3 | 0.071494 | 0.185496 | 1.375489 | 0.000833 | 0.001681 |
| AC104024.1 | 0.01353 | 0.02905 | 1.10241 | 0.000156 | 0.000361 |
| LINC00475 | 0.564107 | 1.189559 | 1.076387 | 1.31E-09 | 6.37E-09 |
| ATP5MFP5 | 0.096211 | 0.532952 | 2.469736 | 0.025862 | 0.038371 |
| AC007608.4 | 0.019099 | 0.091414 | 2.258909 | 0.016675 | 0.025832 |
| AC105129.3 | 0.096038 | 0.042708 | -1.16911 | 1.48E-06 | 4.77E-06 |
| AC023043.4 | 0.426696 | 1.100817 | 1.367294 | 8.64E-16 | 9.10E-15 |
| AC007686.1 | 0.049013 | 0.260588 | 2.410537 | 4.99E-10 | 2.58E-09 |
| PRRX1 | 1.371447 | 2.770017 | 1.014196 | 8.59E-08 | 3.28E-07 |
| CDK5R2 | 0.036541 | 0.198761 | 2.443452 | 2.15E-12 | 1.48E-11 |
| SLC30A3 | 0.041629 | 0.111322 | 1.419082 | 5.84E-13 | 4.31E-12 |
| RARRES1 | 4.595903 | 10.02685 | 1.125449 | 1.62E-14 | 1.45E-13 |
| UNC13A | 0.108476 | 0.224249 | 1.04773 | 0.001651 | 0.003141 |
| PPIAL4H | 0.022561 | 0.053213 | 1.237948 | 0.000191 | 0.000433 |
| AC026369.1 | 0.547469 | 0.247884 | -1.14311 | 1.46E-11 | 9.07E-11 |
| AC133644.1 | 0.827874 | 2.508996 | 1.599628 | 1.98E-13 | 1.55E-12 |
| SLC38A5 | 1.270387 | 6.796022 | 2.419423 | 1.62E-20 | 3.25E-19 |
| Z94160.1 | 0.010124 | 0.040126 | 1.986743 | 3.74E-07 | 1.31E-06 |
| LINC00466 | 0.002781 | 0.014698 | 2.401879 | 0.000862 | 0.001734 |
| AC087392.1 | 0.074149 | 0.243565 | 1.715803 | 0.0009 | 0.001805 |
| HPX | 0.31173 | 1.348599 | 2.113094 | 0.001156 | 0.002269 |
| CTXN3 | 1.895739 | 0.848244 | -1.16021 | 1.92E-06 | 6.08E-06 |
| PIANP | 0.196199 | 0.455744 | 1.215901 | 1.25E-05 | 3.48E-05 |
| AC083967.1 | 0.177305 | 0.646779 | 1.867043 | 0.000724 | 0.001479 |
| APLP1 | 0.610493 | 2.143965 | 1.812235 | 9.02E-11 | 5.09E-10 |
| MTRNR2L6 | 0.08243 | 0.282342 | 1.776203 | 1.52E-05 | 4.18E-05 |
| RPL23P5 | 0.034446 | 0.08209 | 1.252864 | 0.017083 | 0.026408 |
| AC004817.3 | 0.098117 | 0.373649 | 1.929102 | 1.06E-05 | 2.98E-05 |
| LINC01151 | 0.032755 | 0.097879 | 1.57928 | 2.21E-05 | 5.89E-05 |
| MIR587 | 1.563572 | 0.772903 | -1.01649 | 1.30E-07 | 4.82E-07 |
| RNU7-84P | 1.094944 | 0.399227 | -1.45558 | 7.57E-07 | 2.54E-06 |
| AC133565.1 | 0.076903 | 0.264161 | 1.780314 | 0.001227 | 0.002395 |
| DCAF12L2 | 0.067615 | 0.139465 | 1.044495 | 0.001407 | 0.002717 |
| AC022726.1 | 0.153609 | 0.058882 | -1.38338 | 0.007235 | 0.012128 |
| IGLV2-33 | 0.09358 | 0.205513 | 1.134953 | 0.000467 | 0.00099 |
| PRRX2 | 0.665074 | 1.544176 | 1.215251 | 2.26E-15 | 2.25E-14 |
| AL365229.1 | 0.051435 | 0.022014 | -1.22436 | 5.72E-05 | 0.000142 |
| AC244035.3 | 2.074292 | 0.952021 | -1.12355 | 0.027028 | 0.039933 |
| CCKBR | 0.028252 | 0.057464 | 1.024287 | 0.002125 | 0.003957 |
| LY6D | 0.015887 | 0.061881 | 1.961697 | 4.53E-05 | 0.000115 |
| AL450338.2 | 0.037018 | 0.075025 | 1.019149 | 0.027088 | 0.040009 |
| FIRRE | 0.065266 | 0.159947 | 1.293187 | 1.16E-05 | 3.24E-05 |
| BHMG1 | 0.011506 | 0.05348 | 2.21666 | 0.000958 | 0.00191 |
| AC114930.1 | 0.021014 | 0.056224 | 1.419827 | 0.000118 | 0.000278 |
| Z97200.1 | 0.070866 | 0.28462 | 2.005869 | 5.69E-07 | 1.94E-06 |
| KIF2C | 0.917127 | 1.917183 | 1.063795 | 2.94E-20 | 5.70E-19 |
| ACTBL2 | 0.038249 | 0.088343 | 1.207702 | 1.27E-06 | 4.14E-06 |
| NPBWR1 | 0.040275 | 0.111279 | 1.466235 | 4.36E-10 | 2.27E-09 |
| NUDT11 | 0.169376 | 0.475125 | 1.488081 | 6.73E-10 | 3.42E-09 |
| AL596087.2 | 0.053275 | 0.18372 | 1.785969 | 6.38E-16 | 6.81E-15 |
| P2RX6 | 0.060287 | 0.131863 | 1.129107 | 6.84E-05 | 0.000168 |
| AL928921.1 | 0.441397 | 0.0475 | -3.21607 | 0.00021 | 0.000473 |
| CRABP2 | 2.805591 | 11.65006 | 2.05396 | 7.25E-07 | 2.44E-06 |
| AC126773.3 | 0.13222 | 0.361562 | 1.451305 | 0.00172 | 0.003262 |
| CILP2 | 0.17018 | 0.671736 | 1.980833 | 6.40E-18 | 9.01E-17 |
| LINC00705 | 0.033023 | 0.096748 | 1.550763 | 2.94E-05 | 7.69E-05 |
| ENTHD1 | 0.020305 | 0.055424 | 1.448685 | 7.01E-07 | 2.36E-06 |
| IGLV1-41 | 0.486707 | 2.413169 | 2.309802 | 0.003132 | 0.005651 |
| ZIC2 | 0.033962 | 0.219935 | 2.695069 | 5.09E-14 | 4.27E-13 |
| THBS2 | 6.315285 | 12.92177 | 1.032884 | 1.36E-05 | 3.75E-05 |
| PYCR1 | 1.308494 | 3.664794 | 1.485824 | 1.32E-21 | 3.05E-20 |
| MPO | 0.088358 | 0.195883 | 1.148565 | 0.032936 | 0.047734 |
| SRPX | 2.338432 | 5.700875 | 1.285642 | 1.53E-08 | 6.43E-08 |
| TUBA3D | 5.019208 | 13.11177 | 1.385331 | 7.87E-06 | 2.26E-05 |
| LINC00634 | 0.026889 | 0.091576 | 1.767936 | 1.45E-11 | 9.01E-11 |
| AC055807.1 | 0.100598 | 0.029418 | -1.77381 | 0.000469 | 0.000995 |
| AC009948.3 | 0.019904 | 0.008882 | -1.16402 | 0.01915 | 0.02927 |
| IGLV3-9 | 3.28595 | 14.26352 | 2.117947 | 1.61E-06 | 5.16E-06 |
| TRABD2A | 0.259566 | 0.569556 | 1.133736 | 1.28E-11 | 8.04E-11 |
| DLL3 | 0.027305 | 0.072465 | 1.408119 | 6.21E-08 | 2.41E-07 |
| AC015977.1 | 1.142756 | 0.563909 | -1.01898 | 1.59E-09 | 7.65E-09 |
| DPP6 | 0.197543 | 0.081392 | -1.27921 | 9.07E-05 | 0.000218 |
| GJB6 | 0.095914 | 0.273863 | 1.513646 | 6.59E-06 | 1.92E-05 |
| ZNF728 | 0.099692 | 0.048601 | -1.03649 | 0.000365 | 0.00079 |
| IGLV9-49 | 5.449409 | 46.63843 | 3.097348 | 3.79E-05 | 9.72E-05 |
| AL353807.2 | 0.097422 | 0.194941 | 1.00072 | 0.014638 | 0.022985 |
| ADAM33 | 0.151451 | 0.53438 | 1.819016 | 0.001299 | 0.002527 |
| TUBA3C | 0.081892 | 0.275459 | 1.750039 | 7.98E-05 | 0.000194 |
| EMILIN1 | 7.12336 | 15.63354 | 1.134014 | 1.46E-12 | 1.03E-11 |
| KCTD9P1 | 0.037839 | 0.015933 | -1.24786 | 0.002199 | 0.004083 |
| OTP | 0.008821 | 0.024817 | 1.492342 | 8.71E-05 | 0.00021 |
| WT1-AS | 0.028673 | 0.0646 | 1.171866 | 0.005265 | 0.00908 |
| AC091196.1 | 0.037416 | 0.015809 | -1.24295 | 0.004297 | 0.007544 |
| AP004609.1 | 0.033554 | 0.071845 | 1.09841 | 3.62E-05 | 9.32E-05 |
| ANKFN1 | 0.018439 | 0.066211 | 1.844297 | 9.94E-08 | 3.76E-07 |
| RUNDC3A | 0.23003 | 0.551358 | 1.261166 | 9.11E-13 | 6.56E-12 |
| SRPX2 | 0.639364 | 2.342127 | 1.873109 | 9.31E-16 | 9.77E-15 |
| AC012404.1 | 0.369417 | 0.107558 | -1.78014 | 0.001476 | 0.002836 |
| FSIP2 | 0.192598 | 0.089728 | -1.10196 | 0.000908 | 0.001819 |
| WNT2 | 0.094451 | 0.228435 | 1.274155 | 7.30E-06 | 2.11E-05 |
| ARID3C | 0.10642 | 0.2557 | 1.264687 | 1.86E-10 | 1.01E-09 |
| LCT-AS1 | 0.011011 | 0.022526 | 1.032655 | 2.29E-08 | 9.42E-08 |
| AC126468.1 | 0.032457 | 0.068348 | 1.074385 | 7.29E-05 | 0.000178 |
| AC239859.5 | 0.98323 | 2.221079 | 1.17566 | 6.16E-17 | 7.57E-16 |
| CACNG4 | 0.137532 | 0.350341 | 1.348992 | 0.024311 | 0.036296 |
| TUBBP6 | 0.073395 | 0.172136 | 1.229802 | 0.000423 | 0.000906 |
| ATP5MC1P4 | 0.204157 | 0.51849 | 1.344637 | 3.95E-16 | 4.33E-15 |
| UGT1A10 | 0.152399 | 0.683283 | 2.164632 | 0.001333 | 0.002588 |
| AC100849.2 | 0.013209 | 0.029413 | 1.154917 | 0.011919 | 0.019098 |
| NANOGP1 | 0.018157 | 0.051072 | 1.49201 | 1.86E-07 | 6.78E-07 |
| AC126696.3 | 0.018425 | 0.04367 | 1.245022 | 1.41E-06 | 4.55E-06 |
| TAS2R3 | 0.107531 | 0.047128 | -1.19011 | 1.85E-08 | 7.71E-08 |
| IGHV3-41 | 0.174867 | 0.380402 | 1.12127 | 0.000132 | 0.000309 |
| AP003043.1 | 0.162019 | 0.073839 | -1.1337 | 2.14E-12 | 1.47E-11 |
| GDF5 | 0.047586 | 0.107594 | 1.176986 | 7.50E-08 | 2.88E-07 |
| PRELID2P1 | 0.06265 | 0.149539 | 1.255136 | 6.27E-06 | 1.83E-05 |
| AL121989.1 | 0.054373 | 0.119642 | 1.137756 | 5.88E-05 | 0.000146 |
| AL713868.1 | 0.01867 | 0.038385 | 1.039859 | 0.000251 | 0.000559 |
| TRAV38-2DV8 | 0.565764 | 1.168621 | 1.046534 | 0.000652 | 0.001346 |
| ABCA13 | 0.013156 | 0.028665 | 1.123616 | 0.008073 | 0.013402 |
| LINC01563 | 0.011429 | 0.048608 | 2.08844 | 0.000735 | 0.001498 |
| DMRTA2 | 0.006219 | 0.020133 | 1.694717 | 0.018049 | 0.02775 |
| PPIAP45 | 0.323774 | 0.657102 | 1.021133 | 2.50E-05 | 6.59E-05 |
| DKFZP434A062 | 0.010223 | 0.025753 | 1.33292 | 0.013403 | 0.021231 |
| AC090403.1 | 0.021912 | 0.076153 | 1.797195 | 0.022104 | 0.033298 |
| MAST1 | 0.060344 | 0.148085 | 1.295137 | 1.65E-08 | 6.92E-08 |
| LINC02223 | 0.005273 | 0.027342 | 2.374378 | 0.006496 | 0.010991 |
| RPL29P30 | 0.021654 | 0.050003 | 1.207356 | 3.86E-05 | 9.91E-05 |
| AC011755.1 | 0.032637 | 0.011031 | -1.56497 | 0.034241 | 0.049422 |
| IGLC2 | 119.0838 | 391.6245 | 1.717494 | 7.54E-13 | 5.47E-12 |
| RPS19P7 | 0.101574 | 0.327554 | 1.689203 | 4.52E-08 | 1.79E-07 |
| REG1A | 25.01233 | 53.91482 | 1.108042 | 0.001468 | 0.002823 |
| AP000289.1 | 0.032951 | 0.071135 | 1.110252 | 7.00E-05 | 0.000172 |
| HCRT | 0.020956 | 0.099978 | 2.254284 | 8.48E-05 | 0.000205 |
| F3 | 2.536273 | 5.919285 | 1.222713 | 0.000366 | 0.000792 |
| AADAC | 0.069817 | 0.318037 | 2.187554 | 0.011874 | 0.019029 |
| AC104078.1 | 0.015127 | 0.044792 | 1.566166 | 0.001972 | 0.003699 |
| LINC01910 | 0.010194 | 0.037446 | 1.877032 | 0.003838 | 0.006812 |
| SCN4A | 1.437171 | 0.578446 | -1.31298 | 8.77E-20 | 1.57E-18 |
| LAG3 | 2.438654 | 5.626198 | 1.206075 | 6.00E-11 | 3.47E-10 |
| MFSD2A | 1.021747 | 2.346781 | 1.199645 | 8.71E-13 | 6.28E-12 |
| LTB | 4.210894 | 9.350427 | 1.150906 | 1.75E-19 | 2.99E-18 |
| KRT13 | 0.083962 | 0.783008 | 3.221211 | 0.000188 | 0.000427 |
| C1R | 33.43878 | 76.97041 | 1.202782 | 3.08E-25 | 1.26E-23 |
| CCSER1 | 0.282601 | 0.136671 | -1.04805 | 1.82E-24 | 6.57E-23 |
| CLCA2 | 0.015759 | 0.033161 | 1.073295 | 7.52E-06 | 2.17E-05 |
| AL035427.2 | 0.040491 | 0.0164 | -1.30387 | 6.37E-05 | 0.000158 |
| CSTF3-DT | 0.011567 | 0.033102 | 1.516941 | 0.002962 | 0.005365 |
| RPL13AP2 | 0.122938 | 0.268883 | 1.129048 | 0.007083 | 0.01189 |
| AL117329.1 | 0.002802 | 0.012996 | 2.213457 | 0.030804 | 0.044983 |
| POTEM | 0.002944 | 0.008777 | 1.575785 | 0.004472 | 0.007823 |
| CILP | 0.236882 | 0.685262 | 1.532487 | 0.01199 | 0.019199 |
| AP001094.3 | 0.134854 | 0.064458 | -1.06496 | 0.000207 | 0.000468 |
| ESRRG | 1.627418 | 0.768471 | -1.08252 | 5.25E-16 | 5.66E-15 |
| Z98886.1 | 0.019952 | 0.009617 | -1.0528 | 6.19E-06 | 1.81E-05 |
| IGHV5-10-1 | 7.425134 | 19.74688 | 1.411136 | 0.018544 | 0.028437 |
| KREMEN2 | 0.077714 | 0.263106 | 1.759388 | 1.82E-16 | 2.08E-15 |
| AC092844.1 | 0.022995 | 0.094109 | 2.032992 | 0.010491 | 0.017007 |
| LINC01841 | 0.085146 | 0.19147 | 1.169103 | 4.16E-05 | 0.000106 |
| COCH | 0.195172 | 0.451947 | 1.211409 | 2.78E-09 | 1.29E-08 |
| DGKI | 1.32046 | 0.480011 | -1.4599 | 5.64E-15 | 5.34E-14 |
| AKNAD1 | 0.01921 | 0.049504 | 1.365683 | 0.000503 | 0.001063 |
| AL159158.1 | 0.042319 | 0.086634 | 1.033634 | 0.012353 | 0.01973 |
| NPSR1-AS1 | 0.008969 | 0.043194 | 2.267884 | 9.80E-06 | 2.78E-05 |
| AC104435.2 | 0.025816 | 0.065587 | 1.345146 | 0.005353 | 0.009218 |
| AL512378.1 | 0.021616 | 0.06917 | 1.678047 | 7.54E-05 | 0.000184 |
| AC142381.1 | 0.285885 | 0.617323 | 1.11059 | 0.005901 | 0.010067 |
| AL022170.1 | 0.230715 | 0.100564 | -1.198 | 1.28E-11 | 8.01E-11 |
| PRR20G | 0.01325 | 0.065281 | 2.300675 | 2.71E-07 | 9.64E-07 |
| RPS26P35 | 0.042094 | 0.090413 | 1.102932 | 0.032035 | 0.046569 |
| AL359092.1 | 0.129003 | 0.352474 | 1.450116 | 0.002482 | 0.004561 |
| MYO7B | 3.735133 | 1.805836 | -1.04849 | 7.33E-08 | 2.83E-07 |
| AC020928.1 | 0.03319 | 0.068974 | 1.055328 | 7.74E-05 | 0.000188 |
| ST6GALNAC5 | 0.069024 | 0.284513 | 2.043331 | 7.75E-06 | 2.23E-05 |
| PPARGC1A | 5.841546 | 2.767538 | -1.07775 | 2.46E-20 | 4.82E-19 |
| IGLV4-60 | 2.443067 | 12.64108 | 2.371355 | 0.002512 | 0.004613 |
| IGHV3-33 | 9.725995 | 32.45029 | 1.738314 | 6.76E-05 | 0.000166 |
| PTPRQ | 0.007432 | 0.030673 | 2.045208 | 0.005615 | 0.009633 |
| CRYGN | 0.045747 | 0.112089 | 1.29288 | 5.88E-05 | 0.000146 |
| CFHR3 | 0.063493 | 0.146055 | 1.201842 | 1.24E-08 | 5.29E-08 |
| SNORA58 | 0.236813 | 0.09555 | -1.30942 | 1.56E-05 | 4.27E-05 |
| VPREB3 | 0.624821 | 1.304871 | 1.062392 | 2.03E-12 | 1.40E-11 |
| LRRTM2 | 0.170644 | 0.081312 | -1.06945 | 1.88E-08 | 7.82E-08 |
| FOXB1 | 0.02839 | 0.066734 | 1.233067 | 1.09E-06 | 3.58E-06 |
| RNY3P16 | 0.960578 | 1.964495 | 1.032184 | 2.24E-08 | 9.22E-08 |
| TPBGL | 0.312035 | 0.878434 | 1.493228 | 7.42E-16 | 7.88E-15 |
| AC005153.1 | 0.096464 | 0.021958 | -2.13526 | 3.48E-08 | 1.40E-07 |
| CERS1 | 0.079499 | 0.195782 | 1.300231 | 0.005443 | 0.009365 |
| LINC02199 | 0.002326 | 0.010874 | 2.22485 | 0.000232 | 0.00052 |
| LINC01655 | 0.048315 | 0.281555 | 2.542885 | 1.18E-05 | 3.29E-05 |
| AC112721.2 | 0.046158 | 0.281001 | 2.605919 | 1.03E-06 | 3.39E-06 |
| PON1 | 0.040548 | 0.509424 | 3.651157 | 0.00321 | 0.005782 |
| VPS33B-DT | 0.04578 | 0.109031 | 1.251958 | 4.04E-12 | 2.69E-11 |
| DYDC2 | 0.051099 | 0.102882 | 1.009628 | 7.83E-07 | 2.62E-06 |
| MT-TY | 10.32701 | 23.14958 | 1.164564 | 0.001279 | 0.00249 |
| CDKN3 | 1.027536 | 2.19642 | 1.095965 | 8.49E-26 | 3.88E-24 |
| CFHR1 | 0.154821 | 0.529302 | 1.773492 | 6.78E-06 | 1.97E-05 |
| CD19 | 0.174963 | 0.631686 | 1.852163 | 3.40E-11 | 2.03E-10 |
| AC139491.7 | 0.230517 | 0.10059 | -1.19638 | 0.00423 | 0.00744 |
| MYBPC1 | 0.005737 | 0.015815 | 1.462874 | 0.002255 | 0.004178 |
| LINC02323 | 0.018193 | 0.07796 | 2.099308 | 7.71E-07 | 2.58E-06 |
| CELA1 | 0.034299 | 0.076706 | 1.161189 | 4.58E-10 | 2.38E-09 |
| AC012613.2 | 0.0698 | 0.033137 | -1.07479 | 8.50E-06 | 2.43E-05 |
| HNRNPA1P17 | 0.120147 | 0.050417 | -1.25282 | 5.17E-07 | 1.78E-06 |
| PSMB3P1 | 0.138758 | 0.295641 | 1.091271 | 0.000785 | 0.001593 |
| REEP2 | 0.245512 | 0.721155 | 1.554516 | 6.42E-14 | 5.34E-13 |
| NBEAL1 | 1.613651 | 0.799597 | -1.01298 | 5.72E-31 | 7.12E-29 |
| AC108479.2 | 0.040278 | 0.094236 | 1.226299 | 0.014155 | 0.02229 |
| AC124854.1 | 8.131756 | 3.798622 | -1.09809 | 1.15E-15 | 1.20E-14 |
| COL22A1 | 0.51739 | 1.130291 | 1.127371 | 1.19E-12 | 8.47E-12 |
| RPS15AP29 | 0.097224 | 0.039704 | -1.29203 | 0.016871 | 0.026102 |
| AC007405.2 | 0.042902 | 0.104176 | 1.279899 | 1.55E-08 | 6.50E-08 |
| AL590483.4 | 0.007985 | 0.016796 | 1.072803 | 0.005573 | 0.009566 |
| AL139220.2 | 0.022192 | 0.067921 | 1.613841 | 2.43E-17 | 3.15E-16 |
| AC062037.3 | 0.022802 | 0.053667 | 1.234866 | 1.08E-05 | 3.03E-05 |
| AC105760.1 | 0.017058 | 0.007026 | -1.27957 | 0.014734 | 0.023121 |
| ZFHX4 | 0.169228 | 0.420109 | 1.311797 | 4.12E-08 | 1.64E-07 |
| AC092810.3 | 0.017963 | 0.051523 | 1.520184 | 0.002105 | 0.003926 |
| IGLC3 | 57.67464 | 308.0007 | 2.416925 | 1.14E-11 | 7.23E-11 |
| IGLV2-34 | 0.284706 | 0.71906 | 1.336638 | 0.000435 | 0.00093 |
| CXCL5 | 2.214453 | 6.865573 | 1.63243 | 6.11E-06 | 1.79E-05 |
| TOX3 | 1.583481 | 0.692674 | -1.19285 | 1.09E-09 | 5.40E-09 |
| CYP4A11 | 32.77003 | 11.71833 | -1.48361 | 7.33E-14 | 6.05E-13 |
| ACOD1 | 0.018558 | 0.059336 | 1.676871 | 0.014661 | 0.023015 |
| TLCD3B | 0.058479 | 0.350448 | 2.583213 | 2.65E-09 | 1.24E-08 |
| ALX1 | 0.12218 | 0.318674 | 1.383074 | 0.000131 | 0.000307 |
| MTATP8P1 | 1.324881 | 6.892775 | 2.379222 | 0.025739 | 0.038202 |
| SCRG1 | 0.143464 | 0.304197 | 1.084314 | 1.85E-05 | 4.98E-05 |
| SKA1 | 0.397201 | 0.856889 | 1.10924 | 7.39E-21 | 1.54E-19 |
| IL2RG | 2.702017 | 5.805979 | 1.103503 | 5.26E-17 | 6.51E-16 |
| IGHV3-35 | 0.295108 | 0.615989 | 1.061664 | 0.021861 | 0.032971 |
| AC008013.1 | 0.133465 | 0.295659 | 1.147473 | 4.83E-08 | 1.90E-07 |
| IGHV3-52 | 0.139363 | 0.430728 | 1.627925 | 8.53E-05 | 0.000206 |
| IL11 | 0.047547 | 0.241784 | 2.346294 | 1.66E-09 | 7.96E-09 |
| CACNG6 | 0.015725 | 0.177481 | 3.49656 | 0.000658 | 0.001358 |
| TROAP | 0.475144 | 1.234387 | 1.377359 | 1.28E-19 | 2.23E-18 |
| AC020912.1 | 0.14503 | 0.299267 | 1.045077 | 1.49E-12 | 1.05E-11 |
| B3GALT5-AS1 | 0.032685 | 0.068613 | 1.069861 | 1.80E-06 | 5.72E-06 |
| JSRP1 | 0.381713 | 1.0824 | 1.503673 | 9.78E-15 | 8.99E-14 |
| KCNQ2 | 0.002771 | 0.007347 | 1.406809 | 0.022162 | 0.03337 |
| LINC01191 | 0.051949 | 0.111318 | 1.099527 | 5.86E-06 | 1.72E-05 |
| GSG1 | 0.107033 | 0.428284 | 2.000518 | 3.76E-13 | 2.86E-12 |
| AC010198.2 | 0.027451 | 0.079165 | 1.528005 | 7.87E-09 | 3.43E-08 |
| AC023669.1 | 2.464707 | 1.113674 | -1.14609 | 0.006962 | 0.0117 |
| GRIN2D | 0.238602 | 0.584743 | 1.293195 | 4.13E-25 | 1.65E-23 |
| OR2AT1P | 0.086117 | 0.455392 | 2.402744 | 7.16E-09 | 3.14E-08 |
| AL117328.2 | 0.010734 | 0.022766 | 1.08465 | 0.012875 | 0.020475 |
| SLC27A2 | 21.10487 | 10.49227 | -1.00825 | 1.18E-20 | 2.39E-19 |
| SPEG | 0.12928 | 0.308513 | 1.254833 | 7.55E-10 | 3.80E-09 |
| AC126773.4 | 0.034243 | 0.105393 | 1.621894 | 4.95E-10 | 2.56E-09 |
| SMIM22 | 0.209586 | 0.557887 | 1.41243 | 0.002338 | 0.004318 |
| TRIML2 | 0.023213 | 0.10303 | 2.150041 | 0.000462 | 0.000982 |
| IGKV1-33 | 0.385741 | 1.106755 | 1.520631 | 7.09E-05 | 0.000174 |
| AC034213.1 | 0.106618 | 0.263795 | 1.306962 | 6.18E-08 | 2.40E-07 |
| PRSS3 | 0.086598 | 0.825274 | 3.252459 | 2.28E-10 | 1.23E-09 |
| ELOBP1 | 0.031227 | 0.064622 | 1.049241 | 6.89E-05 | 0.000169 |
| IL21-AS1 | 0.055664 | 0.177707 | 1.674681 | 1.14E-11 | 7.21E-11 |
| LINC02154 | 0.150542 | 0.571911 | 1.925623 | 0.005964 | 0.010165 |
| CHAT | 0.097848 | 0.418352 | 2.096101 | 3.24E-05 | 8.42E-05 |
| RPL12P21 | 0.066088 | 0.240827 | 1.86554 | 0.002485 | 0.004566 |
| SEMA3A | 0.246879 | 0.647968 | 1.39212 | 0.000704 | 0.001443 |
| TF | 0.552301 | 2.229736 | 2.013346 | 0.002385 | 0.004398 |
| NLRP7 | 0.033805 | 0.088878 | 1.394587 | 5.51E-06 | 1.63E-05 |
| PLK1 | 0.665234 | 1.492177 | 1.165484 | 2.41E-20 | 4.72E-19 |
| GAPDHP66 | 0.023044 | 0.079842 | 1.792776 | 0.005937 | 0.010125 |
| IGKV2-26 | 0.100651 | 0.371149 | 1.882641 | 0.004193 | 0.007381 |
| IGHJ5 | 1.364527 | 3.884048 | 1.50916 | 0.001411 | 0.002724 |
| AL137246.2 | 0.040338 | 0.094557 | 1.229052 | 0.000363 | 0.000787 |
| SP7 | 0.010936 | 0.023383 | 1.096342 | 0.000665 | 0.001371 |
| PANK1 | 4.078728 | 1.962301 | -1.05557 | 1.94E-30 | 2.14E-28 |
| ECM1 | 5.666283 | 11.37844 | 1.005828 | 4.74E-18 | 6.77E-17 |
| AC079210.1 | 0.182412 | 0.377758 | 1.050266 | 6.17E-11 | 3.56E-10 |
| SCG2 | 1.866697 | 5.755927 | 1.624561 | 1.16E-09 | 5.69E-09 |
| AK3P5 | 0.168847 | 0.074565 | -1.17914 | 0.000197 | 0.000448 |
| SOX2 | 0.018083 | 0.124181 | 2.779745 | 0.000398 | 0.000856 |
| AL023803.2 | 0.101599 | 0.265656 | 1.386669 | 2.31E-11 | 1.40E-10 |
| IGKC | 1444.189 | 4382.777 | 1.601586 | 2.11E-10 | 1.14E-09 |
| HAS2-AS1 | 0.010418 | 0.047642 | 2.193089 | 1.39E-08 | 5.87E-08 |
| CRNN | 0.023021 | 0.058684 | 1.350021 | 1.22E-08 | 5.20E-08 |
| HBA2 | 14.91222 | 32.87622 | 1.14055 | 0.006556 | 0.011081 |
| IL20RB-AS1 | 0.053714 | 0.22982 | 2.097139 | 2.13E-09 | 1.01E-08 |
| LPO | 0.00848 | 0.022221 | 1.389722 | 0.029937 | 0.043848 |
| MNX1-AS2 | 0.071601 | 0.149333 | 1.06047 | 0.000541 | 0.001135 |
| ATP5MFP4 | 0.21425 | 0.452873 | 1.079809 | 0.016708 | 0.025876 |
| MIR508 | 0.245195 | 0.110971 | -1.14374 | 4.72E-06 | 1.41E-05 |
| COLGALT2 | 0.215247 | 0.541612 | 1.331262 | 3.12E-06 | 9.59E-06 |
| IBSP | 0.529639 | 1.211058 | 1.193187 | 7.59E-11 | 4.32E-10 |
| AP003469.3 | 0.049598 | 0.12328 | 1.313596 | 9.60E-05 | 0.00023 |
| AC026956.2 | 0.009734 | 0.027666 | 1.507042 | 0.016677 | 0.025835 |
| OR2A3P | 0.018677 | 0.007304 | -1.3545 | 1.59E-05 | 4.35E-05 |
| LINC02345 | 0.074824 | 0.164442 | 1.135997 | 0.007209 | 0.012087 |
| AC022079.1 | 0.281279 | 0.920325 | 1.710143 | 3.89E-07 | 1.36E-06 |
| TRBV6-5 | 1.042349 | 2.40124 | 1.203941 | 1.37E-06 | 4.42E-06 |
| EFNA2 | 0.104739 | 0.273592 | 1.385235 | 5.63E-08 | 2.20E-07 |
| IGKV1-9 | 17.5488 | 61.60172 | 1.811598 | 9.36E-07 | 3.10E-06 |
| AL078602.1 | 0.004164 | 0.019599 | 2.234614 | 0.023427 | 0.035094 |
| AC023043.3 | 0.048234 | 0.112446 | 1.221109 | 1.28E-07 | 4.78E-07 |
| AC022167.4 | 0.044388 | 0.091152 | 1.038112 | 2.92E-09 | 1.35E-08 |
| CYP4F26P | 0.011118 | 0.028653 | 1.365786 | 0.000239 | 0.000534 |
| TLX1 | 0.009184 | 0.028088 | 1.612685 | 0.000648 | 0.001338 |
| AP000439.2 | 0.017597 | 0.154462 | 3.133881 | 0.009135 | 0.015009 |
| ARHGEF7-IT1 | 0.038339 | 0.018421 | -1.05741 | 3.23E-09 | 1.49E-08 |
| PTX3 | 0.627851 | 1.784411 | 1.506953 | 5.72E-07 | 1.95E-06 |
| F2 | 1.279026 | 4.068763 | 1.669545 | 4.80E-10 | 2.48E-09 |
| AC078820.2 | 0.044003 | 0.09637 | 1.130997 | 1.82E-08 | 7.57E-08 |
| CCIN | 0.065795 | 0.134408 | 1.030575 | 4.53E-08 | 1.79E-07 |
| AL451081.2 | 0.077145 | 0.158967 | 1.043071 | 1.09E-06 | 3.57E-06 |
| TLL2 | 0.057679 | 0.131537 | 1.189358 | 9.36E-13 | 6.72E-12 |
| SLC13A2 | 1.476712 | 0.1844 | -3.00148 | 1.14E-09 | 5.58E-09 |
| IL1RL1 | 3.200998 | 1.194839 | -1.42171 | 2.72E-11 | 1.64E-10 |
| LINC02570 | 0.086465 | 0.24581 | 1.50736 | 2.45E-05 | 6.49E-05 |
| IGHV1-69 | 4.313099 | 14.86853 | 1.785465 | 0.001595 | 0.003042 |
| SERPINE1 | 84.75975 | 179.2357 | 1.080407 | 3.64E-06 | 1.10E-05 |
| CCL25 | 0.049487 | 0.159208 | 1.68578 | 2.91E-09 | 1.35E-08 |
| PLEKHG4B | 0.111088 | 0.278227 | 1.324556 | 5.90E-07 | 2.01E-06 |
| Z98745.1 | 0.042821 | 0.106517 | 1.314688 | 0.026239 | 0.038878 |
| DLGAP1-AS3 | 0.022758 | 0.054398 | 1.25722 | 1.79E-05 | 4.85E-05 |
| AC010677.1 | 0.139758 | 0.290618 | 1.056194 | 7.63E-09 | 3.34E-08 |
| SEMA3D | 1.745841 | 0.761417 | -1.19716 | 7.18E-09 | 3.15E-08 |
| AC105020.2 | 0.053852 | 0.178268 | 1.726983 | 0.024795 | 0.036934 |
| TRPM3 | 1.482089 | 0.592371 | -1.32306 | 1.16E-27 | 7.29E-26 |
| DAND5 | 0.131993 | 0.277496 | 1.072004 | 8.72E-05 | 0.00021 |
| ONECUT1 | 0.02227 | 0.051316 | 1.204302 | 0.006452 | 0.010923 |
| SEMA5A-AS1 | 0.028423 | 0.009398 | -1.59655 | 0.008862 | 0.01459 |
| RFLNA | 0.48465 | 2.309125 | 2.252332 | 6.37E-13 | 4.67E-12 |
| KRT18P40 | 0.009686 | 0.034891 | 1.848958 | 2.40E-05 | 6.35E-05 |
| PI3 | 1.028812 | 8.079139 | 2.973222 | 1.27E-21 | 2.94E-20 |
| KLF1 | 0.041863 | 0.156218 | 1.899812 | 2.34E-09 | 1.10E-08 |
| PDK4 | 237.7284 | 117.2141 | -1.02017 | 2.07E-14 | 1.82E-13 |
| CT75 | 0.044611 | 0.10283 | 1.204793 | 9.68E-07 | 3.20E-06 |
| MYBPC2 | 0.178575 | 0.398286 | 1.157271 | 3.05E-05 | 7.96E-05 |
| PRSS2 | 0.220958 | 0.58204 | 1.397344 | 1.06E-05 | 2.99E-05 |
| AC012236.1 | 0.231977 | 0.69353 | 1.579974 | 9.97E-10 | 4.94E-09 |
| AL669983.1 | 0.118994 | 0.541446 | 2.185932 | 0.003051 | 0.005514 |
| FOXG1 | 0.015571 | 0.091811 | 2.55984 | 0.020851 | 0.0316 |
| AL136380.1 | 0.442396 | 1.258432 | 1.508217 | 1.23E-06 | 4.00E-06 |
| AC093817.2 | 0.091491 | 0.041201 | -1.15095 | 4.05E-05 | 0.000104 |
| APOL1 | 73.30645 | 157.8734 | 1.106756 | 2.71E-10 | 1.44E-09 |
| AL513164.1 | 0.277669 | 0.597004 | 1.104375 | 0.008871 | 0.014604 |
| RPS26P31 | 0.283196 | 0.64206 | 1.180906 | 3.42E-05 | 8.85E-05 |
| AF038458.2 | 0.036409 | 0.088429 | 1.280244 | 0.000478 | 0.001013 |
| PLAC1 | 0.01964 | 0.064113 | 1.706805 | 4.90E-13 | 3.67E-12 |
| AL353583.1 | 0.089365 | 0.03392 | -1.39758 | 0.002167 | 0.004029 |
| AL136164.1 | 0.104718 | 0.047122 | -1.15203 | 0.015963 | 0.024827 |
| ZNF670-ZNF695 | 0.006494 | 0.018879 | 1.539505 | 0.02349 | 0.035179 |
| AL162151.3 | 0.011105 | 0.024494 | 1.141162 | 0.002818 | 0.005127 |
| AC023034.1 | 0.016269 | 0.040243 | 1.306583 | 0.029176 | 0.042836 |
| CENPM | 1.228229 | 2.601568 | 1.082802 | 2.53E-30 | 2.72E-28 |
| AC016549.1 | 0.027001 | 0.011068 | -1.28668 | 0.028733 | 0.042236 |
| HHIPL2 | 0.038564 | 0.081855 | 1.08582 | 0.001755 | 0.003322 |
| HCST | 1.23107 | 2.835824 | 1.203855 | 2.54E-21 | 5.63E-20 |
| AL031651.1 | 0.023908 | 0.05413 | 1.178899 | 0.015589 | 0.024309 |
| AL359704.2 | 10.05301 | 1.455662 | -2.78788 | 8.38E-14 | 6.89E-13 |
| IL6-AS1 | 0.086785 | 0.183367 | 1.079215 | 6.05E-09 | 2.68E-08 |
| KPNA7 | 0.071186 | 0.149552 | 1.070985 | 0.021789 | 0.03287 |
| SPINK13 | 2.591727 | 6.661982 | 1.362038 | 1.23E-09 | 6.04E-09 |
| EEF1DP4 | 0.194593 | 0.473736 | 1.283625 | 6.10E-05 | 0.000151 |
| LINC01615 | 0.244256 | 0.817961 | 1.743639 | 5.13E-16 | 5.53E-15 |
| MRC2 | 6.137527 | 12.75083 | 1.054861 | 1.91E-14 | 1.69E-13 |
| RN7SL838P | 0.491398 | 0.152248 | -1.69047 | 1.59E-05 | 4.34E-05 |
| MTATP6P2 | 0.203439 | 0.448498 | 1.140507 | 0.020905 | 0.031677 |
| AC125611.1 | 0.237618 | 0.737124 | 1.633265 | 3.62E-09 | 1.66E-08 |
| SLC5A5 | 0.036113 | 0.116548 | 1.690345 | 0.000428 | 0.000915 |
| CASR | 0.407485 | 0.198474 | -1.0378 | 0.001719 | 0.003261 |
| AC104211.1 | 1.994793 | 0.939962 | -1.08556 | 1.56E-16 | 1.81E-15 |
| IGKV2-28 | 1.07141 | 4.066159 | 1.924156 | 0.000226 | 0.000508 |
| TAS2R43 | 0.064584 | 0.031339 | -1.04322 | 0.000708 | 0.001449 |
| RSPO4 | 0.149389 | 0.572025 | 1.937 | 2.09E-06 | 6.57E-06 |
| IGFL2 | 0.09992 | 0.307057 | 1.619662 | 2.65E-09 | 1.24E-08 |
| LINC02513 | 0.038305 | 0.155913 | 2.025147 | 2.13E-08 | 8.79E-08 |
| RNU6-549P | 0.189261 | 0.583366 | 1.624024 | 9.57E-05 | 0.000229 |
| CCDC33 | 0.009118 | 0.034868 | 1.935164 | 0.01446 | 0.022729 |
| TMED11P | 0.087563 | 0.032638 | -1.42379 | 0.020703 | 0.03141 |
| AC134879.2 | 0.24248 | 0.569245 | 1.231187 | 1.65E-06 | 5.28E-06 |
| FCRLA | 0.173396 | 0.399959 | 1.205781 | 1.06E-07 | 4.00E-07 |
| ADH1C | 0.543025 | 2.51017 | 2.208694 | 0.000143 | 0.000333 |
| TNFRSF17 | 0.498261 | 1.093692 | 1.134234 | 3.88E-07 | 1.36E-06 |
| SLC7A4 | 0.035062 | 0.135021 | 1.94521 | 1.23E-05 | 3.44E-05 |
| POU2AF1 | 0.505521 | 1.29385 | 1.355826 | 7.52E-07 | 2.53E-06 |
| WIF1 | 0.033185 | 0.075465 | 1.185273 | 0.000656 | 0.001352 |
| AC022726.2 | 0.229389 | 0.06646 | -1.78724 | 0.017561 | 0.027077 |
| ETV4 | 0.252119 | 0.606552 | 1.266523 | 6.41E-06 | 1.87E-05 |
| APOC1P1 | 0.192758 | 0.454451 | 1.237332 | 5.54E-17 | 6.85E-16 |
| AL109653.3 | 0.719333 | 1.49593 | 1.056311 | 3.57E-07 | 1.25E-06 |
| AC011352.1 | 1.104296 | 2.989945 | 1.436992 | 4.45E-09 | 2.01E-08 |
| HMGB3P11 | 0.041053 | 0.019862 | -1.04748 | 3.27E-10 | 1.73E-09 |
| AC034105.1 | 0.15988 | 0.691167 | 2.11204 | 0.000312 | 0.000683 |
| CST6 | 0.536774 | 1.294579 | 1.270095 | 1.14E-10 | 6.37E-10 |
| AL445223.1 | 0.122153 | 0.028595 | -2.09483 | 0.001499 | 0.002877 |
| INA | 0.02329 | 0.082282 | 1.82087 | 0.005264 | 0.009079 |
| AC067752.1 | 0.039623 | 0.12019 | 1.600889 | 1.01E-10 | 5.68E-10 |
| AL049874.2 | 0.037749 | 0.083594 | 1.146945 | 0.001595 | 0.003042 |
| DNAH8-AS1 | 0.015642 | 0.14396 | 3.202186 | 0.000358 | 0.000777 |
| LINC01788 | 0.615288 | 0.221755 | -1.4723 | 1.13E-08 | 4.83E-08 |
| RPL13P4 | 0.055387 | 0.027151 | -1.02857 | 0.000113 | 0.000266 |
| RASGEF1C | 0.14349 | 0.440347 | 1.617693 | 4.54E-14 | 3.83E-13 |
| PRSS21 | 0.075841 | 0.211449 | 1.479268 | 1.65E-06 | 5.29E-06 |
| ZFHX4-AS1 | 0.004157 | 0.029324 | 2.818483 | 2.77E-06 | 8.57E-06 |
| SNX2P1 | 0.159157 | 0.072793 | -1.12857 | 2.67E-08 | 1.09E-07 |
| IGHV1-12 | 0.4763 | 1.170582 | 1.297283 | 0.002035 | 0.003807 |
| SREK1IP1P1 | 0.045218 | 0.277579 | 2.61794 | 0.002647 | 0.004838 |
| CHGA | 0.052033 | 0.171752 | 1.722814 | 9.20E-05 | 0.000221 |
| CACNA1G | 0.012122 | 0.056807 | 2.228451 | 1.96E-06 | 6.20E-06 |
| NUF2 | 0.510297 | 1.089552 | 1.094325 | 1.69E-15 | 1.72E-14 |
| AC002074.1 | 0.14064 | 0.400424 | 1.509523 | 1.11E-06 | 3.65E-06 |
| AC061992.2 | 0.322465 | 0.653985 | 1.020116 | 3.30E-17 | 4.20E-16 |
| IL19 | 0.009597 | 0.020634 | 1.104282 | 0.00613 | 0.010426 |
| PAGE5 | 0.02672 | 2.689064 | 6.653031 | 7.94E-09 | 3.46E-08 |
| EPS15P1 | 0.021336 | 0.097503 | 2.192186 | 5.96E-07 | 2.03E-06 |
| AC108114.1 | 0.049759 | 0.165398 | 1.732922 | 0.026094 | 0.038687 |
| IGKV1-17 | 10.79087 | 39.358 | 1.866846 | 2.46E-06 | 7.66E-06 |
| LNCOG | 0.031727 | 0.118284 | 1.898494 | 1.11E-12 | 7.92E-12 |
| CPZ | 0.011262 | 0.02368 | 1.072294 | 9.58E-07 | 3.17E-06 |
| LINC02324 | 0.005956 | 0.016977 | 1.511289 | 2.97E-07 | 1.06E-06 |
| TMEM158 | 0.518625 | 2.408641 | 2.215456 | 3.23E-22 | 8.18E-21 |
| AP002993.1 | 0.109326 | 0.314489 | 1.524376 | 0.001552 | 0.00297 |
| MIR1-1HG-AS1 | 0.021491 | 0.052862 | 1.298536 | 9.95E-08 | 3.76E-07 |
| AC126120.1 | 0.028322 | 0.119626 | 2.078524 | 0.010623 | 0.017203 |
| MECOM-AS1 | 0.138627 | 0.062284 | -1.15427 | 0.00104 | 0.002059 |
| OLA1P3 | 0.008825 | 0.020206 | 1.195168 | 0.027115 | 0.040041 |
| KRT5 | 0.204246 | 0.622227 | 1.607136 | 5.22E-08 | 2.05E-07 |
| SSC4D | 0.412518 | 0.859932 | 1.059765 | 3.46E-26 | 1.70E-24 |
| KRT4 | 0.031851 | 0.124272 | 1.964081 | 0.000141 | 0.000328 |
| STRA6 | 0.083012 | 0.244696 | 1.559596 | 3.81E-12 | 2.54E-11 |
| PTPRH | 0.378079 | 1.308858 | 1.791549 | 6.09E-17 | 7.50E-16 |
| TMEM145 | 0.45021 | 1.059679 | 1.234959 | 2.54E-05 | 6.69E-05 |
| AC244093.1 | 0.027574 | 0.068791 | 1.318919 | 1.38E-05 | 3.81E-05 |
| AL161431.1 | 0.109835 | 0.544617 | 2.309901 | 3.85E-08 | 1.54E-07 |
| AC006062.1 | 0.031629 | 0.079484 | 1.329413 | 7.37E-05 | 0.00018 |
| ATP6V0E1P2 | 0.091784 | 0.259053 | 1.496926 | 0.020725 | 0.031433 |
| EPHA10 | 0.136969 | 0.27958 | 1.029417 | 2.58E-12 | 1.76E-11 |
| BMP5 | 0.49619 | 0.236679 | -1.06796 | 4.86E-08 | 1.92E-07 |
| TRNP1 | 1.612117 | 3.846279 | 1.254507 | 1.02E-14 | 9.31E-14 |
| IGKV2D-24 | 0.391571 | 0.936965 | 1.258721 | 0.001028 | 0.002038 |
| CPA2 | 0.027576 | 0.072486 | 1.394296 | 5.84E-11 | 3.38E-10 |
| AP001267.2 | 0.02655 | 0.060686 | 1.192656 | 0.024283 | 0.036255 |
| ITGA2B | 0.179901 | 0.698197 | 1.956429 | 6.82E-07 | 2.30E-06 |
| UBE2SP1 | 0.204638 | 0.427898 | 1.064194 | 2.44E-17 | 3.16E-16 |
| C10orf90 | 0.008329 | 0.039935 | 2.261536 | 1.04E-11 | 6.64E-11 |
| AC046158.3 | 0.071564 | 0.034438 | -1.05526 | 0.000557 | 0.001165 |
| SNORD14E | 2.034094 | 5.113423 | 1.329903 | 5.12E-05 | 0.000128 |
| AC116609.3 | 0.1185 | 0.280227 | 1.241704 | 0.005425 | 0.009334 |
| KHDRBS2 | 0.034327 | 0.086484 | 1.333105 | 0.001623 | 0.003091 |
| MAGED4B | 0.003276 | 0.010772 | 1.717219 | 3.30E-07 | 1.16E-06 |
| NEB | 0.420151 | 0.873339 | 1.055633 | 0.001006 | 0.001998 |
| GREM1 | 0.204651 | 0.547281 | 1.419119 | 5.38E-08 | 2.11E-07 |
| LGALS1 | 185.5653 | 372.3993 | 1.004924 | 5.92E-32 | 9.35E-30 |
| AKR1D1 | 0.029581 | 0.201578 | 2.768618 | 7.74E-05 | 0.000188 |
| AC011586.2 | 0.022714 | 0.052744 | 1.215388 | 0.020187 | 0.030699 |
| ALDOB | 56.2825 | 27.97604 | -1.00849 | 9.58E-08 | 3.63E-07 |
| MNX1 | 0.087248 | 0.220065 | 1.334741 | 2.11E-09 | 9.98E-09 |
| IFNL3P1 | 0.035753 | 0.087504 | 1.291285 | 0.012388 | 0.019782 |
| IVL | 0.023648 | 0.13248 | 2.485993 | 3.07E-11 | 1.84E-10 |
| WFDC10B | 0.082503 | 0.367838 | 2.156559 | 6.36E-18 | 8.96E-17 |
| IGHV3-21 | 25.36047 | 76.16914 | 1.586625 | 0.000462 | 0.000981 |
| KRT18P55 | 0.016326 | 0.04113 | 1.333002 | 7.51E-06 | 2.16E-05 |
| PCBP3 | 0.157734 | 0.612514 | 1.957248 | 6.64E-11 | 3.82E-10 |
| FCRL2 | 0.051124 | 0.26136 | 2.353956 | 0.000572 | 0.001194 |
| IGHV3-73 | 5.666506 | 38.00202 | 2.745545 | 0.002625 | 0.004801 |
| CHRDL2 | 0.501951 | 1.604876 | 1.676844 | 3.85E-14 | 3.29E-13 |
| AC015909.1 | 0.018438 | 0.046788 | 1.343435 | 2.24E-07 | 8.07E-07 |
| AC023043.2 | 0.027602 | 0.069492 | 1.332099 | 2.99E-05 | 7.79E-05 |
| AL162725.2 | 0.009354 | 0.020793 | 1.152412 | 0.034241 | 0.049422 |
| LINC01583 | 0.014043 | 0.106898 | 2.928349 | 6.99E-06 | 2.02E-05 |
| AC005330.1 | 0.040904 | 0.100953 | 1.303375 | 0.00111 | 0.002184 |
| SFRP5 | 0.031343 | 0.155748 | 2.313021 | 0.000147 | 0.000342 |
| IGHV1-46 | 8.851793 | 22.57438 | 1.350645 | 0.000122 | 0.000287 |
| FGF14-AS1 | 0.148592 | 0.036296 | -2.03347 | 3.46E-05 | 8.94E-05 |
| ARPP21 | 0.255025 | 0.103033 | -1.30753 | 3.48E-07 | 1.22E-06 |
| SLC6A19 | 15.75914 | 6.451171 | -1.28856 | 7.02E-15 | 6.56E-14 |
| LINC00868 | 0.015357 | 0.075614 | 2.299794 | 0.013419 | 0.021253 |
| LINC01460 | 0.022582 | 0.067826 | 1.586679 | 0.000105 | 0.00025 |
| PLTP | 31.23859 | 65.27712 | 1.063248 | 8.47E-17 | 1.02E-15 |
| RPS26P45 | 0.074143 | 0.233051 | 1.652262 | 0.000574 | 0.001199 |
| KRT79 | 0.007269 | 0.063049 | 3.116691 | 4.30E-07 | 1.49E-06 |
| MAPK8IP2 | 0.278352 | 0.601359 | 1.111318 | 0.0009 | 0.001806 |
| AC246787.1 | 2.303658 | 4.786373 | 1.055006 | 6.93E-16 | 7.37E-15 |
| SMIM23 | 0.036508 | 0.078893 | 1.111676 | 0.001931 | 0.003629 |
| Z98885.1 | 0.129189 | 0.054227 | -1.25239 | 2.25E-08 | 9.24E-08 |
| RCOR2 | 0.178124 | 0.394519 | 1.147216 | 0.010229 | 0.016626 |
| SLC7A13 | 0.421518 | 0.165326 | -1.35029 | 0.01391 | 0.021945 |
| MPHOSPH6P1 | 0.144116 | 0.062367 | -1.20837 | 0.008543 | 0.014113 |
| LYPD1 | 0.106696 | 0.260549 | 1.288044 | 5.46E-16 | 5.87E-15 |
| IGHV6-1 | 8.12292 | 27.36897 | 1.752471 | 2.69E-05 | 7.08E-05 |
| BNIP3P30 | 0.16889 | 0.082597 | -1.03193 | 1.38E-05 | 3.81E-05 |
| SPAG5-AS1 | 0.496641 | 0.208447 | -1.25252 | 1.92E-15 | 1.93E-14 |
| AP003717.4 | 0.009703 | 0.020635 | 1.088557 | 4.58E-06 | 1.37E-05 |
| AC092839.1 | 0.161526 | 0.063349 | -1.35037 | 0.012409 | 0.019809 |
| CADM3 | 1.442815 | 4.280569 | 1.568916 | 1.73E-09 | 8.28E-09 |
| IGHV1-69D | 9.573635 | 25.99465 | 1.441076 | 5.68E-05 | 0.000142 |
| AC079922.1 | 2.096385 | 4.488183 | 1.098228 | 1.25E-18 | 1.94E-17 |
| IGSF23 | 0.185692 | 0.708751 | 1.932366 | 1.20E-12 | 8.54E-12 |
| KIRREL2 | 0.035039 | 0.099488 | 1.505562 | 1.13E-09 | 5.54E-09 |
| AL355336.1 | 0.083062 | 0.035554 | -1.22419 | 3.85E-07 | 1.35E-06 |
| LINC00928 | 0.018494 | 0.052393 | 1.502275 | 9.84E-05 | 0.000235 |
| AL139351.3 | 0.375706 | 0.770315 | 1.035844 | 2.05E-11 | 1.26E-10 |
| LINC01842 | 0.1335 | 0.277029 | 1.053193 | 9.18E-06 | 2.61E-05 |
| C2orf91 | 0.022724 | 0.053592 | 1.23779 | 4.41E-07 | 1.53E-06 |
| FREM2 | 2.424269 | 0.997095 | -1.28175 | 5.33E-29 | 4.27E-27 |
| AC099789.1 | 0.119271 | 0.316284 | 1.406976 | 0.001122 | 0.002207 |
| DUX4L37 | 0.044956 | 0.127347 | 1.502179 | 1.33E-08 | 5.63E-08 |
| LINC01698 | 0.007122 | 0.024515 | 1.783403 | 2.69E-06 | 8.31E-06 |
| AL133444.1 | 0.191475 | 0.08546 | -1.16384 | 2.39E-06 | 7.46E-06 |
| AL354743.2 | 0.087045 | 0.18404 | 1.08018 | 1.82E-10 | 9.90E-10 |
| AL606753.2 | 0.008125 | 0.02663 | 1.712541 | 4.00E-09 | 1.82E-08 |
| AL049767.1 | 0.130983 | 0.545193 | 2.057391 | 1.51E-10 | 8.30E-10 |
| MIR200A | 0.665717 | 0.328064 | -1.02093 | 0.015275 | 0.023871 |
| DERL3 | 0.409444 | 0.864498 | 1.078196 | 2.62E-10 | 1.40E-09 |
| IGHA2 | 21.86223 | 69.32438 | 1.664922 | 1.44E-07 | 5.32E-07 |
| AJ239328.1 | 0.016966 | 0.041034 | 1.274177 | 0.000403 | 0.000866 |
| LINC01554 | 2.309419 | 6.117954 | 1.405519 | 0.007551 | 0.012604 |
| AL358216.1 | 0.646284 | 0.275558 | -1.22981 | 0.002534 | 0.004651 |
| GABRG1 | 0.108177 | 0.04597 | -1.23462 | 5.70E-11 | 3.30E-10 |
| SMIM33 | 0.018933 | 0.057673 | 1.606961 | 0.002108 | 0.00393 |
| IGLV10-54 | 2.811147 | 6.872447 | 1.289665 | 0.009821 | 0.016023 |
| PKMYT1 | 0.312758 | 0.641445 | 1.036281 | 1.28E-19 | 2.23E-18 |
| C1orf210 | 9.745808 | 4.691554 | -1.05472 | 3.96E-23 | 1.14E-21 |
| AC002057.2 | 0.236544 | 0.107603 | -1.13639 | 0.000597 | 0.001242 |
| AC098847.1 | 0.030535 | 0.073738 | 1.271938 | 8.35E-06 | 2.39E-05 |
| ERN2 | 0.020789 | 0.064611 | 1.63595 | 7.95E-06 | 2.28E-05 |
| PLA2G1B | 0.449656 | 0.954356 | 1.085705 | 2.16E-11 | 1.32E-10 |
| IGLV1-44 | 28.67394 | 77.11385 | 1.42725 | 6.27E-07 | 2.13E-06 |
| USP12-AS2 | 0.216754 | 0.507479 | 1.227288 | 0.01227 | 0.019612 |
| RORB | 0.038907 | 0.219396 | 2.49542 | 5.60E-06 | 1.65E-05 |
| LINC02268 | 0.043995 | 0.214682 | 2.286775 | 8.97E-05 | 0.000216 |
| IGHA1 | 167.3777 | 476.5201 | 1.509429 | 1.09E-07 | 4.08E-07 |
| AC112493.1 | 0.024982 | 0.05756 | 1.204167 | 0.011221 | 0.018075 |
| CFL1P4 | 0.033402 | 0.075298 | 1.172686 | 0.000862 | 0.001734 |
| DMRT3 | 0.037845 | 0.105157 | 1.474384 | 1.84E-12 | 1.27E-11 |
| AC090505.1 | 0.011698 | 0.02532 | 1.113973 | 0.000283 | 0.000626 |
| AC015959.1 | 0.035377 | 0.015978 | -1.14675 | 0.00022 | 0.000496 |
| FREM1 | 0.822203 | 0.351912 | -1.22428 | 6.16E-12 | 4.01E-11 |
| AL512328.1 | 0.01052 | 0.023997 | 1.189652 | 0.001795 | 0.003393 |
| AC107884.2 | 0.08407 | 0.027315 | -1.62187 | 7.06E-09 | 3.10E-08 |
| HAMP | 0.322809 | 0.940069 | 1.542086 | 2.14E-21 | 4.80E-20 |
| IGHV3-64D | 4.110258 | 16.70333 | 2.022835 | 0.000796 | 0.001612 |
| AC093225.1 | 0.028943 | 0.011063 | -1.38749 | 0.000241 | 0.000539 |
| Z93241.1 | 0.127836 | 0.376897 | 1.559881 | 5.33E-08 | 2.09E-07 |
| AL138826.1 | 2.85109 | 1.34055 | -1.08869 | 1.92E-08 | 7.97E-08 |
| LINC01133 | 0.028164 | 0.067524 | 1.261546 | 2.94E-10 | 1.56E-09 |
| TMPRSS11D | 0.072823 | 0.032111 | -1.18135 | 0.015523 | 0.024215 |
| AL049836.1 | 0.042761 | 0.119412 | 1.481592 | 2.34E-09 | 1.10E-08 |
| AC008747.1 | 0.067391 | 0.173706 | 1.366019 | 0.001288 | 0.002507 |
| AC100812.1 | 0.170551 | 0.386449 | 1.180072 | 3.65E-10 | 1.92E-09 |
| GRK6P1 | 0.045725 | 0.106502 | 1.219822 | 0.024669 | 0.036762 |
| KIRREL3 | 0.075609 | 0.171509 | 1.181657 | 7.63E-06 | 2.20E-05 |
| LINC02450 | 0.064658 | 0.176538 | 1.449074 | 1.00E-06 | 3.30E-06 |
| AC119424.1 | 0.219816 | 0.109079 | -1.01093 | 2.21E-09 | 1.04E-08 |
| PRDM12 | 0.016564 | 0.033255 | 1.005529 | 1.24E-07 | 4.63E-07 |
| NMU | 0.651116 | 1.487145 | 1.19156 | 4.02E-16 | 4.40E-15 |
| RYR2 | 0.15503 | 0.33897 | 1.128606 | 1.05E-11 | 6.70E-11 |
| AC005912.1 | 13.58443 | 28.69868 | 1.07903 | 6.48E-19 | 1.04E-17 |
| CPNE6 | 0.031091 | 0.014266 | -1.12392 | 4.92E-05 | 0.000124 |
| MT-TC | 8.616548 | 19.48953 | 1.177517 | 6.91E-05 | 0.00017 |
| AC118755.1 | 0.150769 | 0.572023 | 1.923732 | 0.027152 | 0.040089 |
| AC087749.1 | 0.122227 | 0.054008 | -1.17834 | 0.000157 | 0.000362 |
| KRT18P8 | 0.034454 | 0.122418 | 1.829091 | 4.47E-09 | 2.02E-08 |
| POPDC3 | 0.077833 | 0.201208 | 1.370239 | 0.000236 | 0.000529 |
| G6PC | 3.231963 | 1.473697 | -1.13297 | 3.69E-17 | 4.66E-16 |
| AL096855.1 | 0.259229 | 0.842868 | 1.701082 | 8.92E-06 | 2.54E-05 |
| LINC01973 | 0.009715 | 0.03116 | 1.681467 | 0.000217 | 0.000489 |
| TNFRSF13B | 0.061487 | 0.138234 | 1.168752 | 2.33E-08 | 9.54E-08 |
| AC108693.1 | 0.143175 | 0.045128 | -1.66569 | 1.81E-10 | 9.87E-10 |
| LINC02870 | 0.014719 | 0.076966 | 2.38657 | 1.18E-08 | 5.05E-08 |
| AL096712.2 | 0.134077 | 0.061468 | -1.12516 | 9.40E-15 | 8.66E-14 |
| SLN | 1.030818 | 2.934919 | 1.509531 | 3.24E-15 | 3.17E-14 |
| AC020907.1 | 0.024111 | 0.101118 | 2.068275 | 0.000375 | 0.000811 |
| CAV3 | 0.008524 | 0.026459 | 1.634158 | 0.000376 | 0.000812 |
| CYP1D1P | 0.123828 | 0.057951 | -1.09543 | 1.69E-14 | 1.50E-13 |
| LINC01016 | 0.011909 | 0.037323 | 1.648045 | 0.000651 | 0.001344 |
| MFAP2 | 0.487568 | 1.720323 | 1.819004 | 2.06E-10 | 1.11E-09 |
| AL356776.1 | 0.065554 | 0.030791 | -1.0902 | 0.00779 | 0.012969 |
| RN7SL180P | 0.176067 | 0.614715 | 1.803788 | 0.014093 | 0.022201 |
| RPL6P4 | 0.022214 | 0.044553 | 1.00406 | 0.000192 | 0.000437 |
| ATP8A2P2 | 0.04043 | 0.012441 | -1.70037 | 0.00123 | 0.002401 |
| RN7SL559P | 0.204817 | 0.061724 | -1.73044 | 0.000714 | 0.00146 |
| FCHO1 | 0.79368 | 1.620266 | 1.029602 | 1.33E-16 | 1.56E-15 |
| AZU1 | 0.070627 | 0.207709 | 1.556266 | 0.001461 | 0.002811 |
| AC068831.5 | 0.043929 | 0.09153 | 1.059086 | 0.007306 | 0.012232 |
| PTTG1 | 2.268813 | 5.914154 | 1.382234 | 1.77E-39 | 2.28E-36 |
| LINC01857 | 0.869987 | 1.767869 | 1.022946 | 3.92E-08 | 1.57E-07 |
| BX679664.1 | 0.20399 | 0.454847 | 1.156882 | 1.24E-05 | 3.45E-05 |
| AC131971.1 | 0.180968 | 0.073338 | -1.3031 | 0.00139 | 0.002688 |
| AC008269.2 | 0.033071 | 0.013826 | -1.25823 | 0.03363 | 0.048637 |
| OPRD1 | 0.045845 | 0.100443 | 1.131533 | 2.50E-15 | 2.48E-14 |
| AC024619.4 | 0.109711 | 0.255323 | 1.21861 | 0.001567 | 0.002995 |
| LOXL1 | 2.257059 | 5.439415 | 1.269007 | 6.54E-19 | 1.05E-17 |
| MDFI | 1.05077 | 2.394769 | 1.188439 | 6.41E-09 | 2.84E-08 |
| IGLV3-10 | 21.34095 | 56.77695 | 1.411681 | 9.54E-08 | 3.62E-07 |
| MTRNR2L1 | 0.912233 | 1.843016 | 1.014594 | 0.009372 | 0.015355 |
| PTPRZ1 | 0.018242 | 0.109189 | 2.581465 | 2.61E-06 | 8.10E-06 |
| CNFN | 0.994159 | 2.997182 | 1.592058 | 4.36E-19 | 7.09E-18 |
| TMEM270 | 0.071524 | 0.181951 | 1.347042 | 0.023441 | 0.035111 |
| STEAP1B | 0.079086 | 0.159774 | 1.01454 | 9.52E-07 | 3.15E-06 |
| LIN7A | 11.05247 | 4.928874 | -1.16504 | 1.57E-25 | 6.79E-24 |
| ANXA8 | 0.032336 | 0.095239 | 1.558403 | 2.38E-11 | 1.45E-10 |
| GPR78 | 0.005001 | 0.014793 | 1.564594 | 0.001014 | 0.002012 |
| COL18A1-AS2 | 0.310196 | 0.114232 | -1.44122 | 0.000234 | 0.000525 |
| MYMX | 0.118167 | 0.857364 | 2.859085 | 0.000154 | 0.000356 |
| PCK1 | 34.35916 | 13.06585 | -1.39489 | 3.66E-14 | 3.13E-13 |
| IGLV2-28 | 2.733877 | 7.497669 | 1.455494 | 0.000254 | 0.000566 |
| CACNA1B | 0.008831 | 0.14232 | 4.010339 | 0.011953 | 0.019146 |
| UNC5A | 0.377067 | 0.891975 | 1.242182 | 8.39E-11 | 4.75E-10 |
| MYL2 | 0.015141 | 0.045189 | 1.577504 | 2.06E-10 | 1.11E-09 |
| SLC2A3P2 | 0.17746 | 0.088616 | -1.00186 | 1.48E-11 | 9.22E-11 |
| GTSF1 | 0.158329 | 0.349621 | 1.142867 | 1.15E-08 | 4.91E-08 |
| AL160286.2 | 0.04099 | 0.094029 | 1.197815 | 6.34E-07 | 2.15E-06 |
| EFNA5 | 3.542524 | 8.450001 | 1.254174 | 2.72E-16 | 3.05E-15 |
| IGHJ1 | 0.601853 | 1.762228 | 1.549916 | 2.32E-05 | 6.16E-05 |
| C5orf38 | 0.258392 | 0.827252 | 1.678764 | 9.44E-07 | 3.12E-06 |
| IL22RA2 | 0.011784 | 0.031974 | 1.440078 | 0.000116 | 0.000274 |
| IL20RB | 3.928811 | 16.80441 | 2.096675 | 2.28E-17 | 2.97E-16 |
| GABRB2 | 0.139204 | 0.046714 | -1.57527 | 7.41E-06 | 2.14E-05 |
| IGHG2 | 50.43214 | 165.4873 | 1.714305 | 1.60E-07 | 5.86E-07 |
| LINC02544 | 0.199782 | 0.431615 | 1.111316 | 8.04E-10 | 4.03E-09 |
| RBPJL | 0.018478 | 0.072936 | 1.980809 | 0.013278 | 0.021055 |
| SULT2B1 | 0.142343 | 0.32202 | 1.177779 | 7.60E-14 | 6.26E-13 |
| FOXE1 | 0.035314 | 0.130203 | 1.882457 | 5.22E-06 | 1.55E-05 |
| CIB3 | 0.024769 | 0.079629 | 1.684776 | 0.002347 | 0.004333 |
| EML6 | 0.509507 | 0.186188 | -1.45234 | 1.40E-06 | 4.54E-06 |
| ORM1 | 0.467656 | 8.009145 | 4.09813 | 0.009338 | 0.015309 |
| AL158800.1 | 0.013661 | 0.038545 | 1.496496 | 4.34E-08 | 1.72E-07 |
| AL161782.1 | 0.700775 | 0.302758 | -1.21079 | 8.46E-28 | 5.42E-26 |
| AC090983.1 | 0.005968 | 0.021442 | 1.845106 | 0.030549 | 0.044655 |
| AC013391.3 | 0.038682 | 0.098961 | 1.35521 | 4.62E-06 | 1.38E-05 |
| AC234778.1 | 0.069508 | 0.180084 | 1.373426 | 0.004202 | 0.007396 |
| TGFBI | 127.3694 | 345.0475 | 1.437776 | 7.88E-15 | 7.32E-14 |
| ITIH3 | 0.766268 | 2.601282 | 1.763302 | 4.71E-10 | 2.44E-09 |
| GOLGA6L2 | 0.060908 | 0.200327 | 1.717659 | 5.74E-10 | 2.95E-09 |
| AC015911.1 | 0.153173 | 0.311447 | 1.023822 | 0.001016 | 0.002017 |
| C5orf46 | 6.475905 | 23.44759 | 1.856286 | 3.38E-12 | 2.27E-11 |
| AC017104.2 | 0.061338 | 0.132093 | 1.106709 | 0.020684 | 0.031389 |
| AL031280.1 | 0.036494 | 0.090012 | 1.302439 | 0.016796 | 0.025997 |
| LINC01638 | 0.566088 | 1.322942 | 1.224652 | 2.94E-11 | 1.77E-10 |
| ISL2 | 0.019402 | 0.080419 | 2.051324 | 0.000133 | 0.000312 |
| NPY4R | 0.020985 | 0.051196 | 1.286665 | 0.002639 | 0.004824 |
| LINC01517 | 0.305156 | 0.140828 | -1.11561 | 4.19E-15 | 4.03E-14 |
| AL136456.1 | 0.028314 | 0.071408 | 1.334558 | 0.003296 | 0.005925 |
| AC027601.4 | 0.185883 | 0.083253 | -1.15882 | 0.003362 | 0.006033 |
| PCCA-AS1 | 0.156606 | 0.071077 | -1.13969 | 1.82E-06 | 5.77E-06 |
| FAF1-AS1 | 0.145147 | 0.045592 | -1.67068 | 5.35E-09 | 2.39E-08 |
| CEP55 | 1.206578 | 2.514272 | 1.05922 | 2.07E-16 | 2.36E-15 |
| AL353152.1 | 0.245653 | 0.109032 | -1.17187 | 0.000825 | 0.001666 |
| IGHV3OR16-10 | 0.092247 | 0.313719 | 1.76589 | 0.000536 | 0.001125 |
| TAS2R46 | 0.052455 | 0.020581 | -1.34976 | 0.003446 | 0.006175 |
| LINC00337 | 0.050848 | 0.139559 | 1.456604 | 2.61E-08 | 1.06E-07 |
| RPL29P19 | 0.338443 | 0.952848 | 1.493335 | 2.57E-11 | 1.55E-10 |
| IGHG1 | 347.7234 | 1086.085 | 1.643125 | 3.68E-09 | 1.68E-08 |
| LINC02539 | 0.021763 | 0.052938 | 1.282425 | 9.86E-05 | 0.000236 |
| CLLU1-AS1 | 0.063749 | 0.176138 | 1.466221 | 0.002276 | 0.004213 |
| PLEKHS1 | 0.13071 | 0.311962 | 1.254997 | 6.06E-06 | 1.78E-05 |
| RPL23AP12 | 0.03136 | 0.074097 | 1.240487 | 0.014421 | 0.022676 |
| PLAAT1 | 0.473542 | 0.968366 | 1.032061 | 3.47E-07 | 1.22E-06 |
| PKP3 | 0.587391 | 2.032355 | 1.790759 | 3.59E-18 | 5.19E-17 |
| AC005261.5 | 0.248748 | 0.117284 | -1.08468 | 6.76E-09 | 2.98E-08 |
| CDHR4 | 0.034436 | 0.126816 | 1.880766 | 1.26E-06 | 4.11E-06 |
| NUDT1 | 3.12547 | 6.352118 | 1.023165 | 1.33E-88 | 3.76E-84 |
| FAM178B | 0.039289 | 0.102089 | 1.377623 | 1.35E-12 | 9.49E-12 |
| TUBAL3 | 0.114285 | 0.234668 | 1.037991 | 0.004316 | 0.007576 |
| BARX1 | 0.089675 | 0.366313 | 2.030303 | 3.01E-05 | 7.85E-05 |
| LINC02061 | 1.075768 | 0.420314 | -1.35583 | 5.40E-13 | 4.02E-12 |
| RPTN | 0.017972 | 0.227394 | 3.661404 | 0.019328 | 0.029509 |
| AC023669.2 | 2.704901 | 1.211963 | -1.15823 | 0.014661 | 0.023015 |
| CCL21 | 7.144523 | 21.80935 | 1.610037 | 4.65E-08 | 1.84E-07 |
| AC104806.1 | 0.168051 | 0.071912 | -1.2246 | 1.44E-06 | 4.66E-06 |
| AC114812.3 | 0.143135 | 0.060956 | -1.23154 | 0.000863 | 0.001736 |
| AL355810.1 | 0.040171 | 0.111233 | 1.469351 | 0.000423 | 0.000906 |
| CNTN2 | 0.016872 | 0.042419 | 1.330124 | 0.00013 | 0.000304 |
| ALDH3B2 | 0.030843 | 0.069047 | 1.162648 | 0.000427 | 0.000913 |
| MAGEA10 | 0.008345 | 0.180626 | 4.435911 | 0.019458 | 0.029676 |
| KRT19 | 57.12961 | 126.4122 | 1.145826 | 1.02E-15 | 1.07E-14 |
| ITIH1 | 0.324527 | 1.465228 | 2.174716 | 0.001103 | 0.002172 |
| AF233439.2 | 0.010965 | 0.030869 | 1.493231 | 2.54E-08 | 1.04E-07 |
| IGKV1-6 | 9.550121 | 19.93747 | 1.061892 | 1.57E-05 | 4.30E-05 |
| AL136981.3 | 0.031538 | 0.066633 | 1.079161 | 0.005811 | 0.00993 |
| AC111000.1 | 0.037215 | 0.017505 | -1.0881 | 9.75E-09 | 4.20E-08 |
| GTSE1 | 0.643983 | 1.317379 | 1.032576 | 4.13E-19 | 6.75E-18 |
| SLAMF9 | 0.058706 | 0.20945 | 1.835036 | 3.05E-06 | 9.37E-06 |
| LINC02156 | 0.016182 | 0.038299 | 1.242894 | 1.34E-05 | 3.71E-05 |
| AC006557.6 | 0.160471 | 0.420915 | 1.391215 | 3.17E-06 | 9.72E-06 |
| IGHV3-49 | 13.19385 | 26.69476 | 1.016691 | 0.000597 | 0.001242 |
| CNIH3-AS2 | 0.005408 | 0.023167 | 2.098793 | 6.53E-09 | 2.88E-08 |
| MSX2 | 0.208682 | 0.544983 | 1.384904 | 1.97E-20 | 3.89E-19 |
| RPL6P6 | 0.192198 | 0.082499 | -1.22014 | 3.30E-06 | 1.01E-05 |
| AC092620.1 | 0.038235 | 0.087756 | 1.198599 | 0.002693 | 0.004915 |
| SOSTDC1 | 3.39694 | 1.313649 | -1.37066 | 2.62E-06 | 8.12E-06 |
| MOBP | 0.006513 | 0.014012 | 1.105157 | 0.002839 | 0.005161 |
| KRT15 | 0.078049 | 0.259423 | 1.732855 | 3.26E-15 | 3.18E-14 |
| SIX2 | 0.27814 | 0.611064 | 1.13551 | 1.24E-09 | 6.05E-09 |
| AL138720.1 | 0.028259 | 0.067768 | 1.26187 | 0.000949 | 0.001894 |
| TFF2 | 0.290646 | 0.725049 | 1.318818 | 2.13E-07 | 7.69E-07 |
| FOXA1 | 0.109989 | 0.429252 | 1.964467 | 5.08E-06 | 1.51E-05 |
| RPS19P6 | 0.073601 | 0.029639 | -1.31223 | 3.62E-06 | 1.10E-05 |
| AC007342.8 | 0.133824 | 0.061693 | -1.11716 | 0.009335 | 0.015306 |
| LINC02167 | 0.034679 | 0.6794 | 4.292143 | 1.94E-12 | 1.34E-11 |
| RPL10P3 | 0.723987 | 1.453698 | 1.005692 | 1.86E-09 | 8.88E-09 |
| DTNB-AS1 | 0.10767 | 0.237614 | 1.142008 | 1.40E-06 | 4.54E-06 |
| AC016745.2 | 0.003929 | 0.013474 | 1.777792 | 4.60E-05 | 0.000116 |
| PTPN2P2 | 0.049677 | 0.023452 | -1.08288 | 0.019161 | 0.029286 |
| IGKV3D-15 | 1.628636 | 3.67515 | 1.174139 | 6.17E-05 | 0.000153 |
| VSTM2L | 0.429027 | 2.175128 | 2.341959 | 1.19E-12 | 8.45E-12 |
| AC093577.1 | 0.02118 | 0.056471 | 1.414811 | 0.003031 | 0.005479 |
| SCAT8 | 0.073358 | 0.170552 | 1.217178 | 1.80E-05 | 4.87E-05 |
| GOLGA6L7 | 0.122676 | 0.477965 | 1.96205 | 0.006278 | 0.010651 |
| CCNO | 0.386805 | 0.862886 | 1.157562 | 3.21E-10 | 1.70E-09 |
| GRAMD4P2 | 0.014418 | 0.049901 | 1.791234 | 0.00042 | 0.0009 |
| IFITM9P | 0.238714 | 0.574235 | 1.266359 | 0.011753 | 0.01885 |
| COL25A1 | 1.340696 | 0.567029 | -1.24149 | 4.16E-20 | 7.84E-19 |
| RN7SKP197 | 0.201594 | 0.0842 | -1.25957 | 2.26E-11 | 1.37E-10 |
| CEACAM5 | 0.021487 | 0.062829 | 1.54798 | 0.026432 | 0.039141 |
| AL731556.1 | 0.050397 | 0.167186 | 1.730052 | 0.000196 | 0.000445 |
| FSTL4 | 0.294109 | 0.107281 | -1.45495 | 2.27E-07 | 8.17E-07 |
| SNTG2-AS1 | 0.580691 | 2.235072 | 1.944479 | 7.30E-14 | 6.03E-13 |
| DNMT3L | 0.014446 | 0.030705 | 1.087769 | 0.000287 | 0.000632 |
| AC092422.1 | 0.198549 | 0.091155 | -1.12311 | 4.64E-09 | 2.09E-08 |
| KCNJ6 | 0.006361 | 0.02163 | 1.765622 | 0.000487 | 0.00103 |
| AC021678.2 | 0.125421 | 0.293686 | 1.227499 | 2.66E-07 | 9.48E-07 |
| XKR7 | 0.015905 | 0.120286 | 2.918966 | 0.000464 | 0.000985 |
| MANCR | 0.080697 | 0.243312 | 1.592224 | 3.77E-07 | 1.32E-06 |
| ATP1A3 | 0.037238 | 0.108694 | 1.545406 | 1.96E-12 | 1.36E-11 |
| TPT1P5 | 0.475539 | 1.213497 | 1.351536 | 2.60E-12 | 1.77E-11 |
| EIF5AP2 | 0.062408 | 0.141386 | 1.17983 | 1.10E-06 | 3.59E-06 |
| IGHV1OR15-9 | 0.32792 | 1.354448 | 2.04629 | 0.000299 | 0.000657 |
| CHRNA1 | 0.166545 | 0.34391 | 1.046123 | 4.55E-05 | 0.000115 |
| CHRM3 | 0.774738 | 0.317837 | -1.28542 | 4.23E-17 | 5.30E-16 |
| AC104971.3 | 0.111881 | 0.236764 | 1.081476 | 7.62E-07 | 2.55E-06 |
| AC082651.1 | 0.019082 | 0.062488 | 1.71138 | 6.79E-06 | 1.97E-05 |
| AP000897.2 | 0.616696 | 0.303997 | -1.0205 | 9.53E-12 | 6.09E-11 |
| AC026100.1 | 0.022922 | 0.061804 | 1.430986 | 0.005977 | 0.010185 |
| P4HA3 | 1.280178 | 3.091456 | 1.271942 | 7.57E-10 | 3.81E-09 |
| AC112721.1 | 0.027475 | 0.168057 | 2.61278 | 0.001631 | 0.003106 |
| MATN4 | 0.043376 | 0.179935 | 2.052493 | 4.56E-06 | 1.36E-05 |
| AC106801.1 | 0.025664 | 0.059266 | 1.20743 | 5.06E-08 | 1.99E-07 |
| PGBD4P3 | 0.033783 | 0.016845 | -1.00398 | 5.45E-09 | 2.44E-08 |
| HSPE1P11 | 0.265439 | 0.092698 | -1.51776 | 0.000411 | 0.000882 |
| SHOX2 | 0.054175 | 0.242114 | 2.15999 | 4.13E-09 | 1.87E-08 |
| LIF-AS1 | 0.057533 | 0.197747 | 1.781209 | 7.92E-08 | 3.04E-07 |
| AL360178.2 | 0.025915 | 0.004888 | -2.4065 | 0.030414 | 0.044477 |
| AC011294.1 | 0.032993 | 0.084065 | 1.349338 | 7.73E-06 | 2.22E-05 |
| AC026396.1 | 0.223578 | 0.097218 | -1.20148 | 2.72E-11 | 1.64E-10 |
| SLC22A8 | 0.829779 | 0.37154 | -1.15921 | 5.03E-08 | 1.98E-07 |
| RPL10P2 | 0.058672 | 0.329826 | 2.49096 | 0.005996 | 0.010217 |
| AC024940.1 | 0.014915 | 0.037002 | 1.310816 | 0.001211 | 0.002368 |
| AC011506.1 | 0.095713 | 0.193211 | 1.013382 | 2.80E-08 | 1.13E-07 |
| IGKJ3 | 1.638664 | 4.516751 | 1.462765 | 0.000233 | 0.000524 |
| AC006116.5 | 0.116386 | 0.056979 | -1.03041 | 0.006242 | 0.010598 |
| TINAG-AS1 | 0.081881 | 0.025726 | -1.67029 | 2.17E-06 | 6.80E-06 |
| PRAME | 2.551573 | 6.035096 | 1.24199 | 5.25E-16 | 5.66E-15 |
| TUBB3 | 0.036189 | 0.097959 | 1.436625 | 1.29E-11 | 8.11E-11 |
| TEX46 | 0.038194 | 0.08928 | 1.225001 | 0.003163 | 0.005705 |
| AC093849.2 | 0.12602 | 0.377791 | 1.583934 | 1.58E-07 | 5.83E-07 |
| AC087289.3 | 0.003837 | 0.009687 | 1.335871 | 0.003346 | 0.006007 |
| AL356108.1 | 0.023515 | 0.060141 | 1.354758 | 0.003112 | 0.005617 |
| GFRA3 | 0.057547 | 0.148279 | 1.365508 | 7.33E-05 | 0.000179 |
| NPR3 | 37.89745 | 17.05537 | -1.15187 | 6.92E-20 | 1.26E-18 |
| SPIB | 0.150523 | 0.331407 | 1.13862 | 1.41E-08 | 5.95E-08 |
| AC017033.1 | 0.021451 | 0.099916 | 2.219681 | 1.77E-12 | 1.23E-11 |
| AC091182.2 | 0.061225 | 0.178284 | 1.54198 | 0.000245 | 0.000548 |
| AL137786.1 | 0.010046 | 0.026819 | 1.416636 | 0.00092 | 0.001842 |
| COL18A1-AS1 | 0.248241 | 0.0956 | -1.37666 | 5.25E-11 | 3.06E-10 |
| AL391560.2 | 0.008075 | 0.029976 | 1.892255 | 5.89E-05 | 0.000146 |
| NPFFR2 | 0.021406 | 0.072126 | 1.752509 | 3.07E-05 | 8.01E-05 |
| KRT6A | 0.082395 | 0.37937 | 2.202979 | 1.14E-05 | 3.18E-05 |
| AC018695.2 | 0.045782 | 0.137102 | 1.582378 | 0.000718 | 0.001468 |
| AC012150.2 | 0.109665 | 0.050697 | -1.11315 | 0.001232 | 0.002404 |
| SNORA80E | 0.703944 | 0.231675 | -1.60336 | 0.00013 | 0.000303 |
| AC148477.3 | 0.100205 | 0.044819 | -1.16078 | 3.72E-07 | 1.30E-06 |
| Z98749.1 | 0.072154 | 0.030644 | -1.23545 | 0.000515 | 0.001084 |
| FOXA2 | 0.220966 | 0.445498 | 1.011594 | 0.000744 | 0.001515 |
| KCNMA1-AS3 | 0.08213 | 0.017592 | -2.22295 | 0.022681 | 0.034076 |
| PDGFRA | 0.956592 | 1.954231 | 1.030626 | 1.87E-05 | 5.05E-05 |
| LEFTY1 | 0.573703 | 1.210282 | 1.076966 | 0.000213 | 0.00048 |
| AC011352.3 | 2.492457 | 5.46854 | 1.133587 | 1.57E-07 | 5.77E-07 |
| EML5 | 0.085603 | 0.03672 | -1.2211 | 1.25E-18 | 1.94E-17 |
| PPP1R27 | 0.026236 | 0.072174 | 1.459903 | 0.000622 | 0.001289 |
| VWA3B | 0.012135 | 0.028655 | 1.239615 | 8.61E-06 | 2.46E-05 |
| CXCL13 | 2.63934 | 8.168337 | 1.629865 | 1.50E-10 | 8.23E-10 |
| AMH | 0.239578 | 0.516022 | 1.106935 | 0.010626 | 0.017206 |
| UGT3A2 | 0.223897 | 0.727363 | 1.699838 | 0.020005 | 0.030443 |
| DAW1 | 0.070132 | 0.264989 | 1.917783 | 1.00E-06 | 3.31E-06 |
| AP000851.2 | 0.086387 | 0.306458 | 1.826805 | 3.28E-06 | 1.00E-05 |
| AWAT2 | 0.008705 | 0.022636 | 1.37873 | 0.021249 | 0.032138 |
| ADCYAP1 | 0.854681 | 0.387464 | -1.14132 | 4.11E-05 | 0.000105 |
| SLC10A2 | 9.314441 | 4.051182 | -1.20113 | 1.22E-10 | 6.78E-10 |
| CDCA3 | 0.484189 | 1.178155 | 1.282888 | 9.77E-28 | 6.21E-26 |
| C7orf61 | 0.106343 | 0.242781 | 1.190929 | 7.60E-11 | 4.33E-10 |
| CYP19A1 | 0.025324 | 0.051748 | 1.031019 | 8.71E-11 | 4.92E-10 |
| MT2P1 | 0.909448 | 2.382742 | 1.38956 | 7.48E-16 | 7.93E-15 |
| MEG8 | 0.005512 | 0.013398 | 1.281302 | 0.027185 | 0.040137 |
| ADAM12 | 0.924403 | 2.697845 | 1.545214 | 4.43E-16 | 4.82E-15 |
| GAPDHP45 | 0.046432 | 0.155232 | 1.741227 | 0.011042 | 0.017822 |
| LINC01111 | 0.389255 | 0.834971 | 1.101011 | 0.000385 | 0.00083 |
| ASGR1 | 0.359462 | 0.760053 | 1.080261 | 1.28E-18 | 1.98E-17 |
| AC076968.2 | 0.010764 | 0.030886 | 1.520788 | 0.000299 | 0.000657 |
| LMO7DN | 0.020459 | 0.056095 | 1.455169 | 0.017473 | 0.026955 |
| LRTM2 | 0.003127 | 0.008689 | 1.474223 | 8.43E-05 | 0.000204 |
| AC104248.1 | 0.02582 | 0.059691 | 1.209029 | 0.000818 | 0.001654 |
| WNT10A | 0.381753 | 0.915491 | 1.261905 | 9.14E-11 | 5.15E-10 |
| TSKS | 0.0677 | 0.144821 | 1.097048 | 4.75E-06 | 1.42E-05 |
| AP001972.4 | 0.061667 | 0.238845 | 1.953514 | 1.12E-05 | 3.14E-05 |
| AC013270.1 | 0.055118 | 0.142018 | 1.365469 | 2.52E-06 | 7.84E-06 |
| LINC02446 | 0.298776 | 0.723483 | 1.275895 | 5.43E-06 | 1.60E-05 |
| RPL27AP | 0.055854 | 0.119562 | 1.09802 | 0.000377 | 0.000813 |
| PCSK1 | 0.185719 | 0.603875 | 1.701128 | 0.005406 | 0.009305 |
| AL132655.2 | 0.063489 | 0.189294 | 1.576056 | 4.82E-13 | 3.60E-12 |
| DSG3 | 0.008121 | 0.030075 | 1.888857 | 0.016494 | 0.025583 |
| CCL7 | 0.102819 | 0.239384 | 1.219223 | 4.65E-05 | 0.000118 |
| AL591002.1 | 0.284897 | 0.109884 | -1.37446 | 2.12E-10 | 1.14E-09 |
| AL713998.4 | 0.098926 | 0.041983 | -1.23653 | 0.03125 | 0.045551 |
| RPL6P2 | 0.059356 | 0.02581 | -1.20148 | 0.006122 | 0.010415 |
| PLAU | 8.391911 | 17.22451 | 1.037391 | 5.60E-13 | 4.15E-12 |
| MT1XP1 | 0.517255 | 1.03844 | 1.005471 | 1.86E-07 | 6.78E-07 |
| AC105046.2 | 0.007947 | 0.033899 | 2.092738 | 0.015843 | 0.024664 |
| INSL3 | 0.277608 | 0.676888 | 1.285866 | 1.38E-18 | 2.13E-17 |
| IGLV5-37 | 0.532426 | 1.261542 | 1.244536 | 0.007849 | 0.013059 |
| PLXNB3 | 0.289928 | 0.664547 | 1.196676 | 0.000419 | 0.000898 |
| SLC2A12 | 0.351508 | 0.175163 | -1.00486 | 0.006169 | 0.010488 |
| MYH7 | 0.005599 | 0.014318 | 1.354459 | 0.006658 | 0.011238 |
| IGLV7-46 | 8.475724 | 57.554 | 2.763508 | 2.10E-05 | 5.61E-05 |
| AC083973.1 | 0.013637 | 0.028705 | 1.073715 | 1.33E-06 | 4.32E-06 |
| IGHV1OR21-1 | 0.181673 | 0.509846 | 1.48872 | 0.000834 | 0.001683 |
| COL10A1 | 0.320124 | 1.11583 | 1.801414 | 2.33E-08 | 9.55E-08 |
| INHBE | 0.481205 | 1.34819 | 1.4863 | 7.17E-05 | 0.000176 |
| AC006372.2 | 0.227748 | 0.0702 | -1.69789 | 0.000118 | 0.000278 |
| C10orf82 | 0.050055 | 0.109197 | 1.125331 | 2.72E-08 | 1.10E-07 |
| MAPK6P3 | 0.054752 | 0.018964 | -1.52964 | 0.011461 | 0.018425 |
| AC087894.3 | 0.010253 | 0.047491 | 2.211565 | 0.013314 | 0.021109 |
| PPP2R2C | 0.226021 | 0.526433 | 1.219795 | 9.78E-11 | 5.49E-10 |
| BLK | 0.098158 | 0.274328 | 1.482724 | 1.83E-07 | 6.66E-07 |
| IGLV7-43 | 3.912525 | 14.40174 | 1.880072 | 0.000128 | 0.000301 |
| AL355796.1 | 0.203023 | 0.562896 | 1.471224 | 6.07E-13 | 4.46E-12 |
| RDM1 | 0.132964 | 0.282591 | 1.087676 | 5.14E-15 | 4.89E-14 |
| AP000844.2 | 0.299776 | 0.965386 | 1.687218 | 6.66E-05 | 0.000164 |
| ORM2 | 0.213659 | 5.932003 | 4.795137 | 0.00425 | 0.007471 |
| APOC4-APOC2 | 0.008664 | 0.018333 | 1.081278 | 0.000869 | 0.001747 |
| IL17RD | 2.926788 | 1.380021 | -1.08463 | 1.87E-33 | 3.97E-31 |
| AL353803.1 | 1.315899 | 0.625638 | -1.07265 | 1.92E-11 | 1.18E-10 |
| FCRL4 | 0.005316 | 0.027057 | 2.347586 | 2.64E-06 | 8.18E-06 |
| AL357514.1 | 0.215505 | 0.07822 | -1.46212 | 1.49E-09 | 7.19E-09 |
| AC136428.1 | 0.270239 | 0.567544 | 1.070496 | 0.000118 | 0.000278 |
| RPS15P5 | 0.240609 | 0.538926 | 1.163394 | 6.50E-12 | 4.23E-11 |
| AC051635.1 | 0.034978 | 0.096724 | 1.467425 | 0.000178 | 0.000408 |
| CARD11 | 2.444569 | 5.786492 | 1.243109 | 2.10E-14 | 1.85E-13 |
| FGF5 | 0.037054 | 0.174241 | 2.233394 | 3.17E-11 | 1.90E-10 |
| APCDD1L-DT | 0.070088 | 0.503933 | 2.845988 | 2.42E-09 | 1.14E-08 |
| TRIM71 | 0.308797 | 0.142088 | -1.11988 | 0.000685 | 0.001409 |
| GCK | 0.042873 | 0.150635 | 1.812904 | 3.64E-11 | 2.16E-10 |
| AL445253.1 | 0.03709 | 0.07796 | 1.071681 | 4.49E-05 | 0.000114 |
| AC093627.6 | 0.028908 | 0.073195 | 1.34029 | 5.85E-06 | 1.72E-05 |
| AIM2 | 0.891875 | 2.127176 | 1.254026 | 1.53E-10 | 8.39E-10 |
| AC004593.1 | 0.051365 | 0.018744 | -1.45433 | 0.023914 | 0.03576 |
| IGKV1D-13 | 0.683795 | 2.540294 | 1.89336 | 0.009653 | 0.015769 |
| AL022718.1 | 0.153026 | 0.33863 | 1.145936 | 5.00E-06 | 1.48E-05 |
| MRPL12 | 2.837768 | 5.701329 | 1.006542 | 3.35E-18 | 4.88E-17 |
| RN7SL502P | 0.15551 | 0.072205 | -1.10685 | 0.001175 | 0.002303 |
| AL365356.1 | 0.075758 | 0.029268 | -1.37208 | 0.003273 | 0.005888 |
| C1orf158 | 0.004802 | 0.024112 | 2.328172 | 0.020844 | 0.031593 |
| AC009145.4 | 0.04888 | 0.101269 | 1.050884 | 0.000371 | 0.000802 |
| TAFA3 | 0.079006 | 0.162192 | 1.037665 | 6.98E-06 | 2.02E-05 |
| PPFIA1P1 | 0.046434 | 0.017351 | -1.42015 | 1.77E-05 | 4.81E-05 |
| RPL10P14 | 0.049161 | 0.108409 | 1.140916 | 7.75E-06 | 2.23E-05 |
| ISLR | 5.549669 | 16.77365 | 1.595723 | 2.30E-10 | 1.23E-09 |
| SERBP1P3 | 0.076309 | 0.16397 | 1.103514 | 1.42E-07 | 5.26E-07 |
| LINC02195 | 0.183163 | 0.457136 | 1.319492 | 7.38E-13 | 5.37E-12 |
| AC124276.1 | 0.023089 | 0.065725 | 1.509231 | 0.000183 | 0.000418 |
| RPLP0P2 | 0.080238 | 0.304063 | 1.922014 | 1.95E-19 | 3.32E-18 |
| SNRPGP4 | 0.993439 | 0.42221 | -1.23447 | 5.66E-11 | 3.28E-10 |
| RPL12P37 | 0.037904 | 0.091168 | 1.266191 | 0.034086 | 0.049222 |
| CCNYL7 | 0.171468 | 0.056951 | -1.59015 | 6.29E-07 | 2.13E-06 |
| AC022440.1 | 0.008679 | 0.027471 | 1.66229 | 0.000966 | 0.001924 |
| C19orf81 | 0.12861 | 0.39957 | 1.635444 | 3.25E-14 | 2.80E-13 |
| C1QTNF1 | 9.589209 | 19.68994 | 1.037975 | 1.43E-19 | 2.47E-18 |
| AC091304.2 | 0.037821 | 0.082686 | 1.128465 | 0.006453 | 0.010923 |
| ZPLD1 | 0.08067 | 0.467633 | 2.535282 | 1.34E-06 | 4.35E-06 |
| AL109840.2 | 0.031166 | 0.068494 | 1.135985 | 0.000304 | 0.000668 |
| LINC01914 | 0.149484 | 0.551854 | 1.884301 | 2.26E-16 | 2.57E-15 |
| IGKV1-13 | 0.121179 | 0.509903 | 2.073077 | 0.001696 | 0.00322 |
| LINC01234 | 0.068788 | 0.386849 | 2.491551 | 1.81E-15 | 1.83E-14 |
| IGDCC4 | 0.547572 | 1.412618 | 1.36725 | 1.60E-12 | 1.12E-11 |
| NFIA-AS2 | 0.028128 | 0.060844 | 1.113098 | 1.52E-10 | 8.32E-10 |
| HMGB3P7 | 0.080407 | 0.306521 | 1.93059 | 7.29E-08 | 2.81E-07 |
| SLC6A18 | 8.286361 | 3.64809 | -1.1836 | 2.16E-09 | 1.02E-08 |
| EREG | 0.141881 | 0.293982 | 1.051044 | 0.001298 | 0.002526 |
| IGHV3-72 | 3.476551 | 8.326526 | 1.260058 | 0.010429 | 0.016919 |
| HOXD12 | 0.011051 | 0.028322 | 1.357801 | 0.000616 | 0.001278 |
| GRIK4 | 0.208887 | 0.422526 | 1.016318 | 4.05E-14 | 3.44E-13 |
| SNORA80B | 0.420903 | 0.132623 | -1.66616 | 0.0113 | 0.018184 |
| PIMREG | 0.512193 | 1.166132 | 1.186972 | 1.65E-25 | 7.09E-24 |
| AC124067.3 | 0.008512 | 0.042969 | 2.335714 | 0.000591 | 0.001231 |
| ETDC | 0.117153 | 0.043588 | -1.42639 | 8.76E-07 | 2.92E-06 |
| RPS26P47 | 0.305967 | 1.065611 | 1.800233 | 0.020163 | 0.030667 |
| DDX59-AS1 | 0.252858 | 0.521385 | 1.044022 | 2.23E-07 | 8.04E-07 |
| AC024475.1 | 0.054985 | 0.027201 | -1.01537 | 0.026243 | 0.038882 |
| XIRP1 | 0.058746 | 0.131964 | 1.167586 | 0.005317 | 0.009162 |
| IGKV2D-28 | 0.488741 | 1.584059 | 1.696485 | 0.000122 | 0.000286 |
| CRP | 1.659907 | 7.115711 | 2.099905 | 2.59E-09 | 1.21E-08 |
| ONECUT2 | 0.051188 | 0.178431 | 1.801482 | 1.25E-08 | 5.33E-08 |
| SCG5 | 1.324111 | 3.046997 | 1.202364 | 7.13E-12 | 4.62E-11 |
| CCL11 | 0.241051 | 0.707827 | 1.554057 | 3.57E-10 | 1.88E-09 |
| NKX6-1 | 0.360454 | 0.752676 | 1.062213 | 0.001868 | 0.00352 |
| AP003175.1 | 0.006812 | 0.019739 | 1.534832 | 0.00331 | 0.005947 |
| ADD2 | 0.244231 | 0.498771 | 1.03013 | 0.000486 | 0.001027 |
| AP004147.1 | 0.005355 | 0.025557 | 2.254794 | 0.018629 | 0.028543 |
| COL19A1 | 0.180827 | 0.078575 | -1.20246 | 4.04E-10 | 2.11E-09 |
| QRSL1P3 | 0.053566 | 0.012841 | -2.06056 | 0.026818 | 0.039652 |
| SLC25A21 | 0.356351 | 0.152106 | -1.22823 | 5.48E-15 | 5.19E-14 |
| TMEM151A | 0.047835 | 0.272429 | 2.509743 | 5.47E-08 | 2.14E-07 |
| AC128709.1 | 0.03452 | 0.072258 | 1.065724 | 0.017111 | 0.026446 |
| RTL3 | 0.020868 | 0.052805 | 1.339373 | 3.05E-05 | 7.94E-05 |
| AC008277.1 | 0.068155 | 0.023283 | -1.54953 | 0.00255 | 0.004677 |
| BRDT | 0.008 | 0.026386 | 1.72164 | 0.02243 | 0.033732 |
| FZD2 | 0.813871 | 1.858853 | 1.191541 | 2.52E-27 | 1.47E-25 |
| ZBED2 | 0.42775 | 0.999432 | 1.22434 | 2.01E-09 | 9.56E-09 |
| SLC6A11 | 0.006521 | 0.02312 | 1.825932 | 7.49E-06 | 2.16E-05 |
| FABP6 | 13.33648 | 31.13697 | 1.223251 | 3.62E-18 | 5.24E-17 |
| AC096637.2 | 0.031622 | 0.121919 | 1.946908 | 0.00167 | 0.003175 |
| RPL29P2 | 0.025089 | 0.069763 | 1.475383 | 0.016177 | 0.025132 |
| S100A7 | 0.043452 | 0.203758 | 2.229359 | 2.46E-06 | 7.66E-06 |
| GACAT2 | 1.199275 | 3.254274 | 1.440173 | 5.11E-08 | 2.01E-07 |
| AC010099.1 | 0.029039 | 0.092189 | 1.666593 | 0.000858 | 0.001727 |
| IGKV3-15 | 20.23475 | 92.33145 | 2.189987 | 1.17E-06 | 3.84E-06 |
| AL591242.1 | 0.041538 | 0.019007 | -1.1279 | 5.92E-06 | 1.74E-05 |
| SNORA47 | 1.664387 | 0.446054 | -1.8997 | 0.002469 | 0.004539 |
| CLMP | 0.495677 | 2.072027 | 2.063571 | 2.30E-08 | 9.46E-08 |
| LINC01447 | 0.008868 | 0.028186 | 1.668335 | 0.029033 | 0.042644 |
| AC009403.2 | 0.010823 | 0.023877 | 1.141524 | 0.026854 | 0.039693 |
| DUTP7 | 0.082042 | 0.030534 | -1.42595 | 3.57E-05 | 9.22E-05 |
| GNAS-AS1 | 0.026205 | 0.063524 | 1.277447 | 1.64E-09 | 7.87E-09 |
| IGHV2-70 | 6.66344 | 21.65573 | 1.70041 | 0.000115 | 0.000272 |
| AC016769.5 | 0.02703 | 0.077527 | 1.520172 | 1.92E-05 | 5.17E-05 |
| RPL37P6 | 0.76626 | 1.78108 | 1.216847 | 1.19E-16 | 1.40E-15 |
| COX6CP2 | 0.104756 | 0.273232 | 1.38309 | 0.002626 | 0.004803 |
| MAB21L4 | 0.554351 | 0.2744 | -1.01452 | 0.019419 | 0.029626 |
| IGHV2-5 | 13.08469 | 26.18413 | 1.000813 | 0.001928 | 0.003624 |
| IGHV3-13 | 3.027023 | 6.879836 | 1.184475 | 0.000691 | 0.001419 |
| KRT25 | 0.116135 | 0.285244 | 1.296389 | 4.72E-06 | 1.41E-05 |
| CYP4A22 | 3.651061 | 1.509558 | -1.27419 | 1.11E-12 | 7.91E-12 |
| AL136301.1 | 0.044773 | 0.096292 | 1.10478 | 0.017474 | 0.026955 |
| SLC8A2 | 0.025267 | 0.074567 | 1.561261 | 9.46E-08 | 3.59E-07 |
| TFF1 | 0.276663 | 1.387501 | 2.326288 | 0.000306 | 0.000672 |
| AP001783.1 | 0.028818 | 0.143026 | 2.311235 | 1.14E-07 | 4.29E-07 |
| AL592114.3 | 0.038678 | 0.016328 | -1.24417 | 3.25E-07 | 1.14E-06 |
| RSPO2 | 0.007718 | 0.01672 | 1.115387 | 0.021129 | 0.031982 |
| SV2C | 0.079574 | 0.038862 | -1.03393 | 6.87E-13 | 5.02E-12 |
| UST-AS2 | 0.219108 | 0.653484 | 1.576509 | 7.18E-15 | 6.70E-14 |
| LINC02538 | 0.212727 | 0.096414 | -1.14169 | 1.49E-08 | 6.26E-08 |
| APOLD1 | 54.69772 | 26.95372 | -1.021 | 1.51E-21 | 3.46E-20 |
| GNB3 | 0.134219 | 0.293518 | 1.128858 | 0.002724 | 0.004967 |
| HBQ1 | 0.034934 | 0.232992 | 2.737569 | 1.50E-05 | 4.12E-05 |
| SMKR1 | 0.449853 | 1.092705 | 1.280378 | 5.26E-24 | 1.76E-22 |
| TRAF6P1 | 0.041009 | 0.019548 | -1.06894 | 2.65E-06 | 8.19E-06 |
| AF127577.6 | 0.083192 | 0.028536 | -1.54367 | 0.011388 | 0.018318 |
| BASP1 | 6.608989 | 15.35301 | 1.21602 | 6.76E-18 | 9.48E-17 |
| FXYD4 | 1.718147 | 3.646499 | 1.085659 | 0.001405 | 0.002714 |
| PTCSC2 | 0.011598 | 0.040429 | 1.801515 | 0.015986 | 0.024861 |
| CEMIP | 0.378949 | 0.983862 | 1.376455 | 0.024031 | 0.035917 |
| IGLV3-19 | 69.62367 | 193.1539 | 1.472101 | 4.81E-06 | 1.43E-05 |
| AC108865.2 | 0.039913 | 0.088108 | 1.142396 | 2.34E-06 | 7.32E-06 |
| MUC12-AS1 | 0.253339 | 0.616113 | 1.282126 | 3.02E-16 | 3.37E-15 |
| DQX1 | 0.04571 | 0.151925 | 1.732773 | 2.25E-05 | 5.98E-05 |
| AL592494.1 | 0.04214 | 0.101704 | 1.271117 | 4.65E-08 | 1.84E-07 |
| DMP1 | 0.07776 | 0.156468 | 1.008778 | 1.65E-05 | 4.51E-05 |
| HAR1B | 0.011041 | 0.026351 | 1.255025 | 1.83E-06 | 5.80E-06 |
| AC093702.1 | 0.068234 | 0.278485 | 2.02903 | 1.67E-13 | 1.33E-12 |
| ASIC1 | 0.23569 | 0.4798 | 1.025545 | 3.24E-12 | 2.18E-11 |
| SKAP1-AS1 | 0.109583 | 0.222739 | 1.023335 | 0.000529 | 0.001112 |
| AP003783.1 | 0.0448 | 0.018339 | -1.28856 | 0.01756 | 0.027076 |
| KRT16 | 0.076581 | 0.266217 | 1.797538 | 6.31E-07 | 2.14E-06 |
| RAB3B | 0.091127 | 0.210937 | 1.210859 | 1.15E-08 | 4.91E-08 |
| ENPP7P8 | 1.73103 | 0.698151 | -1.31002 | 4.45E-15 | 4.26E-14 |
| COL26A1 | 0.111995 | 0.227912 | 1.025044 | 1.59E-09 | 7.67E-09 |
| DKKL1 | 0.113965 | 0.369625 | 1.697469 | 0.001056 | 0.002089 |
| CAMK2N2 | 0.417556 | 1.1032 | 1.401653 | 6.66E-20 | 1.22E-18 |
| AL031733.2 | 0.082471 | 0.191046 | 1.211967 | 5.05E-07 | 1.74E-06 |
| IGHV3-47 | 0.196371 | 0.403751 | 1.039883 | 0.002565 | 0.004703 |
| SLC22A24 | 0.742172 | 0.231743 | -1.67923 | 5.04E-24 | 1.69E-22 |
| IGLV2-14 | 45.07109 | 170.7559 | 1.921661 | 4.62E-05 | 0.000117 |
| EDN3 | 0.036117 | 0.112977 | 1.645266 | 4.05E-08 | 1.61E-07 |
| LAMP5 | 0.535606 | 1.422739 | 1.409428 | 2.50E-10 | 1.34E-09 |
| TBC1D27P | 0.024631 | 0.077941 | 1.661877 | 0.000148 | 0.000343 |
| MNX1-AS1 | 0.030764 | 0.132546 | 2.107163 | 1.43E-11 | 8.92E-11 |
| AL592071.1 | 0.104726 | 0.269211 | 1.362124 | 7.39E-10 | 3.73E-09 |
| AP001324.3 | 0.05674 | 0.152948 | 1.430605 | 7.53E-05 | 0.000184 |
| IGKV3OR2-268 | 0.814618 | 2.328688 | 1.515323 | 6.37E-06 | 1.86E-05 |
| SPON1 | 22.6719 | 11.13915 | -1.02527 | 4.89E-08 | 1.93E-07 |
| AC018797.2 | 0.076856 | 0.032598 | -1.23736 | 0.000184 | 0.00042 |
| NCAM2 | 0.038407 | 0.10661 | 1.472917 | 1.69E-11 | 1.05E-10 |
| WNT4 | 0.115166 | 0.316274 | 1.457465 | 1.34E-05 | 3.70E-05 |
| TFPI2 | 12.63561 | 38.00872 | 1.588835 | 1.94E-07 | 7.05E-07 |
| FAM30A | 0.077463 | 0.270744 | 1.805339 | 1.36E-05 | 3.76E-05 |
| AC104058.1 | 0.129288 | 0.057736 | -1.16304 | 0.003595 | 0.006417 |
| LINC00570 | 0.015283 | 0.037501 | 1.295022 | 0.000265 | 0.000588 |
| AL713998.1 | 0.105772 | 0.329729 | 1.640318 | 1.20E-05 | 3.34E-05 |
| AC073569.1 | 0.128395 | 0.057424 | -1.16088 | 0.001216 | 0.002377 |
| SYCE1L | 1.092981 | 2.61062 | 1.256124 | 3.55E-20 | 6.80E-19 |
| LINC02742 | 0.007507 | 0.022898 | 1.608906 | 0.014988 | 0.02347 |
| LINC00626 | 0.655811 | 2.249323 | 1.77814 | 5.22E-05 | 0.000131 |
| COL5A1 | 7.683818 | 18.07374 | 1.234 | 2.05E-11 | 1.25E-10 |
| MAFA-AS1 | 0.144088 | 0.443894 | 1.623267 | 1.07E-09 | 5.28E-09 |
| TMEM92-AS1 | 0.322801 | 0.721248 | 1.15985 | 1.42E-15 | 1.45E-14 |
| GBX2 | 0.031607 | 0.073639 | 1.220257 | 5.92E-08 | 2.31E-07 |
| SOX6 | 2.086731 | 1.009852 | -1.0471 | 3.56E-30 | 3.71E-28 |
| AC097709.1 | 0.02727 | 0.072138 | 1.403442 | 9.48E-05 | 0.000227 |
| DES | 3.922599 | 8.205411 | 1.064766 | 0.002098 | 0.003913 |
| TRBV4-2 | 0.856817 | 2.110124 | 1.300269 | 8.28E-06 | 2.37E-05 |
| AC009560.4 | 0.076859 | 0.029706 | -1.37145 | 0.003293 | 0.005919 |
| SLC12A8 | 1.031501 | 2.131626 | 1.04721 | 1.84E-11 | 1.13E-10 |
| STUM | 2.256806 | 0.930915 | -1.27756 | 1.40E-14 | 1.26E-13 |
| STARD13-AS | 0.281775 | 0.121729 | -1.21088 | 7.34E-11 | 4.19E-10 |
| AC132825.1 | 0.020631 | 0.089932 | 2.124017 | 0.012537 | 0.019986 |
| FOXD3-AS1 | 0.012868 | 0.054273 | 2.076426 | 0.001498 | 0.002875 |
| LINC01558 | 0.5291 | 0.261861 | -1.01474 | 0.000187 | 0.000426 |
| SSTR3 | 0.023483 | 0.047711 | 1.022701 | 5.01E-05 | 0.000126 |
| MYOSLID | 0.060791 | 0.205498 | 1.757201 | 1.43E-06 | 4.63E-06 |
| IGLL5 | 0.306052 | 0.99003 | 1.693694 | 5.77E-08 | 2.25E-07 |
| CERKL | 0.709088 | 0.352197 | -1.00958 | 2.37E-11 | 1.44E-10 |
| AC026780.2 | 0.45354 | 0.202976 | -1.15992 | 6.77E-10 | 3.44E-09 |
| RPS2P24 | 0.025546 | 0.068885 | 1.431097 | 4.64E-08 | 1.83E-07 |
| IGLC6 | 0.296422 | 0.93712 | 1.660582 | 8.17E-06 | 2.34E-05 |
| AC106872.6 | 0.069367 | 0.180138 | 1.37678 | 0.029516 | 0.043288 |
| MIR429 | 1.536255 | 0.754759 | -1.02533 | 0.005296 | 0.009129 |
| WFDC5 | 0.201035 | 2.031715 | 3.33718 | 1.81E-07 | 6.61E-07 |
| FOXN1 | 0.019332 | 0.04702 | 1.282275 | 0.006811 | 0.011473 |
| PI15 | 0.191072 | 0.408792 | 1.097251 | 1.20E-05 | 3.35E-05 |
| APOH | 1.09729 | 17.51512 | 3.996585 | 6.20E-11 | 3.57E-10 |
| PRG4 | 0.13323 | 0.555695 | 2.060373 | 0.001295 | 0.00252 |
| ST8SIA3 | 0.002704 | 0.061166 | 4.499628 | 0.000777 | 0.001577 |
| LINC01524 | 0.006956 | 0.022965 | 1.723221 | 0.032292 | 0.046911 |
| RTBDN | 0.01244 | 0.044583 | 1.841476 | 0.004904 | 0.008519 |
| KRT78 | 0.008107 | 0.038637 | 2.252804 | 4.99E-08 | 1.96E-07 |
| AC012435.2 | 0.013742 | 0.038192 | 1.474703 | 0.020646 | 0.031339 |
| LINC01283 | 0.024579 | 0.049901 | 1.021635 | 0.005478 | 0.009418 |
| EMX2OS | 12.46459 | 6.180218 | -1.01211 | 9.32E-36 | 3.81E-33 |
| PDSS1P1 | 0.062214 | 0.02248 | -1.46861 | 0.02849 | 0.041899 |
| TCHH | 0.142664 | 0.497708 | 1.802682 | 4.30E-07 | 1.49E-06 |
| CDC25C | 0.265637 | 0.611013 | 1.201747 | 2.18E-23 | 6.52E-22 |
| HMSD | 0.024156 | 0.054376 | 1.170587 | 1.31E-08 | 5.57E-08 |
| IGLV6-57 | 16.58658 | 59.61754 | 1.84572 | 1.85E-06 | 5.88E-06 |
| SYN1 | 0.377421 | 0.776699 | 1.041183 | 1.01E-10 | 5.68E-10 |
| GTF2IP7 | 0.026217 | 0.070019 | 1.417221 | 0.016507 | 0.025601 |
| CHRNA9 | 0.007153 | 0.035392 | 2.30679 | 0.001569 | 0.002999 |
| CLVS2 | 0.100826 | 0.043058 | -1.22751 | 9.01E-12 | 5.77E-11 |
| AL355309.1 | 0.315163 | 0.961481 | 1.609161 | 6.55E-10 | 3.33E-09 |
| PPIAP35 | 0.073697 | 0.205707 | 1.480909 | 0.005033 | 0.008716 |
| LUM | 18.369 | 51.33257 | 1.482602 | 5.45E-09 | 2.44E-08 |
| AL137782.1 | 0.083561 | 0.038919 | -1.10234 | 6.53E-11 | 3.75E-10 |
| AC124067.4 | 0.159271 | 0.396395 | 1.315452 | 5.08E-10 | 2.62E-09 |
| IGLV1-51 | 0.598339 | 1.304997 | 1.125011 | 0.002271 | 0.004206 |
| KRT6B | 0.027927 | 0.119502 | 2.097323 | 1.90E-06 | 6.01E-06 |
| PSMD10P2 | 0.022696 | 0.066858 | 1.558652 | 0.011229 | 0.018084 |
| LINC02269 | 0.047007 | 0.143446 | 1.609551 | 8.68E-09 | 3.77E-08 |
| AL117340.1 | 0.096647 | 0.04687 | -1.04406 | 0.004167 | 0.00734 |
| IGHV1-14 | 0.138071 | 0.292429 | 1.082673 | 0.006288 | 0.010666 |
| MOGAT1 | 0.202369 | 0.096947 | -1.06173 | 1.87E-09 | 8.90E-09 |
| IGHGP | 37.4602 | 157.8659 | 2.075269 | 1.13E-10 | 6.30E-10 |
| AC025423.5 | 0.289391 | 0.070118 | -2.04516 | 1.94E-05 | 5.22E-05 |
| AP003174.1 | 0.046731 | 0.09879 | 1.079998 | 0.00055 | 0.001152 |
| SLC17A9 | 1.393824 | 3.020423 | 1.115702 | 1.27E-09 | 6.19E-09 |
| APOC3 | 1.290235 | 20.0963 | 3.961224 | 3.60E-06 | 1.09E-05 |
| AC107373.1 | 0.04161 | 0.10673 | 1.358968 | 1.47E-06 | 4.74E-06 |
| ZNF683 | 0.830394 | 1.759493 | 1.083292 | 6.31E-15 | 5.93E-14 |
| LILRP2 | 0.029915 | 0.070745 | 1.241761 | 7.45E-08 | 2.87E-07 |
| FDCSP | 1.835071 | 22.27412 | 3.601461 | 4.24E-10 | 2.21E-09 |
| RGS7 | 0.621674 | 0.186518 | -1.73684 | 0.009609 | 0.015703 |
| CYP3A4 | 0.413527 | 0.13259 | -1.64101 | 7.17E-05 | 0.000176 |
| ISCA2P1 | 0.051999 | 0.117419 | 1.175118 | 9.05E-07 | 3.01E-06 |
| AL355612.1 | 0.04454 | 0.010851 | -2.03727 | 0.00308 | 0.005564 |
| AC022960.2 | 0.097453 | 0.047048 | -1.05055 | 0.029166 | 0.042825 |
| ZIC4 | 0.004873 | 0.027306 | 2.486226 | 6.65E-05 | 0.000164 |
| ZIC5 | 0.016618 | 0.079745 | 2.262652 | 3.63E-07 | 1.27E-06 |
| AC131009.2 | 0.024496 | 0.096127 | 1.97241 | 0.000382 | 0.000824 |
| AC116021.1 | 0.048823 | 0.13607 | 1.478708 | 3.05E-08 | 1.23E-07 |
| LINC01436 | 0.960776 | 2.182771 | 1.183888 | 1.23E-07 | 4.61E-07 |
| ADRA1D | 0.056114 | 0.132074 | 1.234914 | 0.010788 | 0.017442 |
| AF064860.2 | 0.021582 | 0.04332 | 1.00518 | 1.17E-10 | 6.50E-10 |
| BRS3 | 0.07788 | 0.311977 | 2.002119 | 4.37E-06 | 1.31E-05 |
| LINC01691 | 0.020949 | 0.045424 | 1.116555 | 6.22E-08 | 2.42E-07 |
| IGKJ4 | 2.290103 | 5.705326 | 1.316897 | 0.000886 | 0.00178 |
| AC021231.1 | 0.055858 | 0.02232 | -1.3234 | 1.61E-05 | 4.39E-05 |
| AC024270.4 | 0.296684 | 0.109422 | -1.43902 | 0.004215 | 0.007416 |
| TMPRSS6 | 0.153966 | 0.451382 | 1.551736 | 1.06E-09 | 5.25E-09 |
| C11orf86 | 0.864482 | 3.119512 | 1.851413 | 1.16E-09 | 5.72E-09 |
| APOC1 | 20.32117 | 53.75746 | 1.403482 | 4.73E-20 | 8.86E-19 |
| CBS | 0.018161 | 0.03729 | 1.037992 | 0.000102 | 0.000243 |
| AL589787.1 | 0.004046 | 0.014773 | 1.868428 | 6.51E-10 | 3.31E-09 |
| LL22NC03-63E9.3 | 0.008302 | 0.02627 | 1.661794 | 5.69E-10 | 2.92E-09 |
| CRYBG2 | 0.091617 | 0.209107 | 1.190551 | 2.50E-19 | 4.21E-18 |
| PNLIPRP2 | 0.013353 | 0.034633 | 1.375022 | 1.25E-07 | 4.65E-07 |
| DRP2 | 0.015682 | 0.057135 | 1.865299 | 6.39E-12 | 4.16E-11 |
| IGLV1-40 | 120.753 | 257.8476 | 1.09446 | 3.06E-06 | 9.39E-06 |
| HAO2 | 18.35132 | 8.149568 | -1.17109 | 3.08E-16 | 3.43E-15 |
| HPCAL4 | 0.106633 | 0.289638 | 1.441596 | 6.69E-10 | 3.40E-09 |
| HSD17B6 | 0.338207 | 1.592884 | 2.235661 | 5.28E-06 | 1.56E-05 |
| KIF20A | 1.245255 | 3.039888 | 1.287577 | 1.82E-24 | 6.56E-23 |
| TCL1A | 0.110823 | 0.448335 | 2.016321 | 0.006886 | 0.011586 |
| AL135927.1 | 0.49996 | 0.164601 | -1.60284 | 8.98E-18 | 1.24E-16 |
| RN7SL208P | 0.274942 | 0.574373 | 1.062858 | 0.004345 | 0.007621 |
| MKX | 0.156209 | 0.314734 | 1.010652 | 0.000166 | 0.000382 |
| RPS20P12 | 0.07332 | 0.17281 | 1.236908 | 0.001486 | 0.002855 |
| LRRC15 | 0.173913 | 1.407709 | 3.016913 | 2.81E-11 | 1.69E-10 |
| APOC2 | 0.030304 | 0.06517 | 1.10471 | 0.000146 | 0.00034 |
| GPR79 | 0.055184 | 0.026476 | -1.05954 | 0.000657 | 0.001354 |
| IGLV1-47 | 20.88576 | 57.23495 | 1.454377 | 1.47E-06 | 4.74E-06 |
| SNORA38B | 0.962694 | 0.207085 | -2.21685 | 0.009634 | 0.01574 |
| THRAP3P3 | 0.113785 | 0.027392 | -2.05449 | 0.003675 | 0.006545 |
| PSME2P4 | 0.138899 | 0.054404 | -1.35226 | 1.25E-09 | 6.10E-09 |
| WFDC12 | 0.091964 | 0.228195 | 1.311125 | 1.34E-06 | 4.35E-06 |
| AC105101.1 | 0.014209 | 0.053801 | 1.920842 | 0.019026 | 0.029101 |
| GRIN2B | 0.025987 | 0.053406 | 1.039193 | 1.63E-05 | 4.43E-05 |
| AL445183.2 | 0.019074 | 0.047888 | 1.328016 | 6.16E-09 | 2.73E-08 |
| FGF10 | 0.02517 | 0.057321 | 1.187379 | 0.02278 | 0.034207 |
| LMO1 | 0.302394 | 0.770052 | 1.348527 | 1.77E-09 | 8.48E-09 |
| CCN5 | 0.768427 | 2.314254 | 1.590568 | 1.56E-14 | 1.40E-13 |
| SNORA2B | 0.233931 | 0.090658 | -1.36759 | 0.031745 | 0.04619 |
| AC006270.1 | 0.193013 | 0.444824 | 1.204535 | 1.11E-08 | 4.74E-08 |
| RTL4 | 0.162064 | 0.064688 | -1.32499 | 8.41E-05 | 0.000204 |
| CCNA1 | 0.140823 | 0.549333 | 1.9638 | 2.35E-10 | 1.26E-09 |
| AC003092.1 | 0.28735 | 1.794301 | 2.642542 | 1.39E-10 | 7.65E-10 |
| IGLV2-5 | 0.69832 | 2.044774 | 1.549982 | 4.35E-07 | 1.51E-06 |
| AC025175.2 | 0.118138 | 0.058097 | -1.02394 | 3.78E-05 | 9.70E-05 |
| TIMP1 | 202.9682 | 464.127 | 1.193266 | 2.78E-38 | 2.07E-35 |
| AL445471.1 | 0.008637 | 0.02412 | 1.481522 | 0.002456 | 0.004517 |
| KLK11 | 0.010812 | 0.140756 | 3.702525 | 8.65E-05 | 0.000209 |
| AC080038.1 | 0.183603 | 0.43081 | 1.230465 | 7.47E-07 | 2.51E-06 |
| FRMD5 | 0.038876 | 0.130811 | 1.750515 | 4.15E-14 | 3.51E-13 |
| LINC02725 | 0.017419 | 0.058099 | 1.737876 | 0.000291 | 0.000641 |
| IFNL1 | 0.038353 | 0.109117 | 1.508442 | 4.52E-05 | 0.000115 |
| L1CAM | 1.625957 | 3.259562 | 1.00339 | 0.001002 | 0.001991 |
| AC009518.1 | 0.016681 | 0.086849 | 2.380284 | 2.79E-07 | 9.93E-07 |
| TSGA10IP | 0.041534 | 0.084958 | 1.032457 | 4.49E-06 | 1.34E-05 |
| GATA3-AS1 | 0.038402 | 0.092182 | 1.263302 | 0.004608 | 0.00804 |
| SNORA28 | 0.979747 | 0.425867 | -1.20201 | 0.004327 | 0.007593 |
| IGLV3-16 | 0.35936 | 6.170366 | 4.101856 | 5.71E-05 | 0.000142 |
| ITPKA | 0.538178 | 2.559161 | 2.249515 | 7.96E-24 | 2.56E-22 |
| TRIM63 | 5.172213 | 0.377705 | -3.77545 | 0.010194 | 0.016579 |
| MYBL2 | 1.4063 | 4.219706 | 1.585238 | 1.13E-30 | 1.32E-28 |
| RPL30P11 | 0.027845 | 0.120968 | 2.119159 | 0.012832 | 0.020413 |
| LINC01705 | 0.084661 | 0.276962 | 1.70991 | 2.97E-08 | 1.20E-07 |
| KLK13 | 0.031888 | 0.124245 | 1.96212 | 8.34E-18 | 1.15E-16 |
| EFL1P1 | 0.013535 | 0.028661 | 1.082374 | 0.008576 | 0.01416 |
| AC112715.1 | 0.081139 | 0.217282 | 1.421095 | 1.43E-06 | 4.62E-06 |
| AC119868.2 | 0.046209 | 0.097419 | 1.076041 | 0.000495 | 0.001045 |
| CLVS1 | 0.020134 | 0.055193 | 1.454864 | 0.01678 | 0.02598 |
| AL390071.1 | 0.03622 | 0.081354 | 1.167447 | 0.000175 | 0.0004 |
| KRTAP16-1 | 0.016063 | 0.032924 | 1.035386 | 0.000562 | 0.001176 |
| SRD5A2 | 0.026756 | 0.0645 | 1.269438 | 0.020679 | 0.031384 |
| AHSG | 0.194769 | 1.470345 | 2.916321 | 0.000118 | 0.000278 |
| PRICKLE2-DT | 0.010366 | 0.024788 | 1.257788 | 8.47E-05 | 0.000205 |
| LINC00618 | 0.056361 | 0.153426 | 1.444769 | 0.005862 | 0.01001 |
| RPL23AP48 | 0.11224 | 0.317784 | 1.501463 | 0.000903 | 0.001811 |
| AC078850.1 | 0.223721 | 0.538726 | 1.267848 | 0.014506 | 0.022795 |
| GRAMD4P3 | 0.011091 | 0.050694 | 2.192444 | 0.011174 | 0.018013 |
| AC105384.2 | 0.108081 | 0.257897 | 1.254689 | 0.000135 | 0.000316 |
| LEMD1-DT | 0.006098 | 0.014386 | 1.23823 | 0.001042 | 0.002064 |
| HS6ST3 | 0.679509 | 0.243936 | -1.47799 | 7.91E-08 | 3.03E-07 |
| RNU6-44P | 0.96301 | 0.454382 | -1.08364 | 1.11E-11 | 7.02E-11 |
| PPP1R1A | 5.028155 | 10.28023 | 1.031772 | 4.85E-18 | 6.93E-17 |
| RNFT1P2 | 0.171721 | 0.074141 | -1.21173 | 2.81E-16 | 3.15E-15 |
| TAC3 | 0.02537 | 0.088972 | 1.81025 | 1.77E-06 | 5.62E-06 |
| AP001054.1 | 0.091728 | 0.039414 | -1.21865 | 6.36E-09 | 2.82E-08 |
| SACS-AS1 | 0.008635 | 0.028164 | 1.705567 | 0.00137 | 0.002653 |
| LINC01943 | 0.489774 | 1.015523 | 1.052035 | 4.17E-20 | 7.86E-19 |
| AVPR1B | 3.234139 | 1.585277 | -1.02865 | 8.70E-16 | 9.16E-15 |
| TCAM1P | 0.023001 | 0.084027 | 1.869155 | 0.000901 | 0.001806 |
| UBE2S | 1.81169 | 3.682722 | 1.023436 | 1.87E-40 | 3.10E-37 |
| CPA1 | 0.020288 | 0.048953 | 1.270795 | 0.013943 | 0.021992 |
| C4orf45 | 0.079776 | 0.035538 | -1.16657 | 1.42E-10 | 7.81E-10 |
| LINC01989 | 0.018995 | 0.087664 | 2.206336 | 0.002604 | 0.004765 |
| MMP9 | 4.300202 | 17.51221 | 2.025885 | 1.08E-13 | 8.72E-13 |
| CD177 | 0.177853 | 2.368477 | 3.735201 | 5.29E-05 | 0.000132 |
| KLRF2 | 0.075194 | 0.165352 | 1.136843 | 1.30E-07 | 4.85E-07 |
| LAIR2 | 0.316744 | 1.396463 | 2.140387 | 1.22E-09 | 5.96E-09 |
| IGHG4 | 13.28551 | 41.05772 | 1.6278 | 3.70E-09 | 1.69E-08 |
| SLC18A3 | 0.503083 | 2.479205 | 2.301009 | 0.000251 | 0.000561 |
| REG3G | 0.40981 | 2.236743 | 2.448372 | 2.45E-08 | 1.00E-07 |
| BATF | 2.655711 | 6.01185 | 1.178711 | 6.88E-22 | 1.65E-20 |
| APCDD1L | 0.365573 | 2.468494 | 2.7554 | 6.01E-11 | 3.48E-10 |
| LINC00645 | 0.590138 | 0.105822 | -2.47941 | 0.000351 | 0.000762 |
| ROR1-AS1 | 0.034332 | 0.104624 | 1.607583 | 4.13E-05 | 0.000105 |
| MIR205HG | 0.026439 | 0.101278 | 1.937587 | 1.24E-05 | 3.45E-05 |
| GLRA3 | 0.006376 | 0.016558 | 1.376814 | 0.000835 | 0.001685 |
| AL355076.2 | 0.019612 | 0.056318 | 1.52183 | 0.01449 | 0.022771 |
| IQCA1L | 0.005773 | 0.012959 | 1.166581 | 0.018783 | 0.028768 |
| FAM83A | 0.011214 | 0.098243 | 3.131061 | 1.03E-06 | 3.40E-06 |
| AC004707.1 | 0.027334 | 0.05949 | 1.121942 | 1.35E-06 | 4.37E-06 |
| ST7-AS2 | 0.056994 | 0.019079 | -1.5788 | 0.009442 | 0.015465 |
| AL139317.4 | 0.270374 | 0.133255 | -1.02077 | 7.58E-15 | 7.05E-14 |
| CRYBA4 | 0.033333 | 0.087777 | 1.396909 | 4.65E-09 | 2.09E-08 |
| AC027288.2 | 0.196428 | 0.486156 | 1.307421 | 0.004462 | 0.007806 |
| TNNT1 | 0.18567 | 2.465962 | 3.73134 | 1.89E-23 | 5.73E-22 |
| AP001527.1 | 0.011135 | 0.022748 | 1.03061 | 0.000271 | 0.000601 |
| SPINK2 | 0.237339 | 0.778857 | 1.714408 | 2.52E-09 | 1.18E-08 |
| AC093909.5 | 0.024567 | 0.00902 | -1.44546 | 1.34E-08 | 5.68E-08 |
| LINC00525 | 0.091984 | 0.207685 | 1.174937 | 2.74E-06 | 8.46E-06 |
| NCOA4P3 | 0.032026 | 0.01326 | -1.27216 | 3.06E-06 | 9.40E-06 |
| IGKV1D-16 | 1.159601 | 2.722204 | 1.231147 | 0.000182 | 0.000416 |
| AP001981.2 | 0.02004 | 0.041051 | 1.034564 | 4.25E-06 | 1.28E-05 |
| ERVW-1 | 0.090326 | 0.043238 | -1.06285 | 2.08E-13 | 1.63E-12 |
| ARL9 | 0.089681 | 0.21594 | 1.26776 | 4.30E-08 | 1.71E-07 |
| C1QTNF1-AS1 | 0.201821 | 0.463476 | 1.199418 | 1.45E-14 | 1.30E-13 |
| TEDC2 | 0.298375 | 0.616398 | 1.046735 | 2.10E-23 | 6.33E-22 |
| NANOGP6 | 0.021497 | 0.045799 | 1.091189 | 0.000806 | 0.001631 |
| AC004012.1 | 0.054138 | 0.109219 | 1.012498 | 0.000221 | 0.000498 |
| HPR | 0.38245 | 0.793985 | 1.053841 | 0.001177 | 0.002305 |
| RTP3 | 0.097812 | 0.405338 | 2.051046 | 2.24E-06 | 7.00E-06 |
| GNG4 | 0.242705 | 0.489291 | 1.011488 | 2.40E-06 | 7.50E-06 |
| SNX18P9 | 0.063564 | 0.025164 | -1.33684 | 8.77E-09 | 3.80E-08 |
| LINC00327 | 0.032598 | 0.096653 | 1.568034 | 0.000573 | 0.001197 |
| MYOZ3 | 0.160819 | 0.349226 | 1.118722 | 2.93E-08 | 1.19E-07 |
| AC006262.2 | 0.08002 | 0.337068 | 2.074611 | 1.54E-07 | 5.69E-07 |
| MFAP4 | 12.18407 | 31.81286 | 1.384614 | 6.57E-08 | 2.55E-07 |
| RDH16 | 0.118343 | 0.34087 | 1.526249 | 0.011913 | 0.019089 |
| IGKV1D-17 | 0.265974 | 0.871225 | 1.711757 | 0.01544 | 0.024102 |
| CDC20 | 1.877866 | 4.911233 | 1.386991 | 2.52E-28 | 1.80E-26 |
| NLGN4Y-AS1 | 0.05837 | 0.13773 | 1.238536 | 0.006467 | 0.010945 |
| SAMMSON | 0.004204 | 0.016192 | 1.945312 | 0.004344 | 0.007621 |
| AGTR1 | 6.322227 | 2.904652 | -1.12207 | 3.33E-17 | 4.23E-16 |
| ZDHHC4P1 | 0.07936 | 0.034494 | -1.20207 | 0.001564 | 0.00299 |
| RPL10P5 | 0.04982 | 0.132406 | 1.410167 | 0.004403 | 0.007714 |
| NKX2-5 | 0.022431 | 0.220126 | 3.29475 | 9.23E-05 | 0.000221 |
| KCNS1 | 0.439879 | 0.942319 | 1.099109 | 1.68E-14 | 1.50E-13 |
| PGLYRP2 | 0.099445 | 0.340888 | 1.777329 | 2.37E-06 | 7.41E-06 |
| CIDEC | 0.20329 | 1.655494 | 3.02565 | 3.78E-12 | 2.52E-11 |
| ROPN1 | 0.00247 | 0.0053 | 1.101342 | 3.12E-05 | 8.12E-05 |
| AL356234.2 | 0.005988 | 0.020165 | 1.751789 | 0.000737 | 0.001502 |
| C10orf99 | 7.668809 | 40.0721 | 2.385524 | 1.18E-10 | 6.58E-10 |
| SLC18A2 | 0.702384 | 0.197736 | -1.82868 | 1.58E-17 | 2.10E-16 |
| AL133325.3 | 0.017145 | 0.048808 | 1.509365 | 0.030913 | 0.045112 |
| KCNH3 | 0.102468 | 0.268297 | 1.388653 | 2.67E-07 | 9.54E-07 |
| LCT | 0.007357 | 0.023897 | 1.699562 | 0.014537 | 0.022839 |
| EPS8L3 | 1.027216 | 2.166055 | 1.07633 | 0.00075 | 0.001526 |
| MMP23B | 0.027796 | 0.060847 | 1.130294 | 5.44E-10 | 2.80E-09 |
| KISS1 | 0.341235 | 1.007839 | 1.562427 | 8.59E-05 | 0.000207 |
| TBX18 | 0.198549 | 0.433204 | 1.125553 | 1.00E-06 | 3.30E-06 |
| TRPM8 | 0.095167 | 0.225389 | 1.243889 | 3.33E-11 | 1.99E-10 |
| AC134312.5 | 0.027327 | 0.086492 | 1.662224 | 3.25E-13 | 2.49E-12 |
| ANKRD18B | 0.013556 | 0.05993 | 2.144391 | 1.13E-05 | 3.17E-05 |
| CPLX2 | 0.025846 | 0.084005 | 1.700526 | 0.018366 | 0.028192 |
| KDELR3 | 7.63592 | 16.80712 | 1.138199 | 1.36E-26 | 7.13E-25 |
| PTGIS | 2.598071 | 5.645182 | 1.119579 | 0.000134 | 0.000313 |
| LINC02387 | 0.020375 | 0.079132 | 1.957478 | 0.010673 | 0.017272 |
| PF4 | 0.209565 | 0.563321 | 1.426558 | 1.78E-07 | 6.51E-07 |
| AC008080.2 | 0.061777 | 0.022166 | -1.4787 | 1.10E-06 | 3.62E-06 |
| AC069360.1 | 0.046319 | 0.095132 | 1.038321 | 1.36E-07 | 5.05E-07 |
| AC092881.1 | 0.16978 | 0.064183 | -1.40339 | 5.30E-11 | 3.09E-10 |
| AICDA | 0.203192 | 0.601384 | 1.565441 | 0.003977 | 0.007034 |
| LINC01411 | 0.119035 | 0.42603 | 1.839566 | 5.41E-11 | 3.15E-10 |
| ETS1-AS1 | 0.042768 | 0.019946 | -1.10045 | 0.003648 | 0.0065 |
| TP53AIP1 | 0.202122 | 0.05206 | -1.95698 | 0.014268 | 0.022456 |
| LINC02029 | 0.01686 | 0.034577 | 1.036187 | 0.000575 | 0.001201 |
| CCNE1 | 0.571576 | 1.179972 | 1.045735 | 7.62E-20 | 1.38E-18 |
| MTND4LP1 | 0.099698 | 0.316765 | 1.667774 | 0.000361 | 0.000782 |
| RPL10P13 | 0.264489 | 0.641818 | 1.278959 | 2.46E-05 | 6.50E-05 |
| LINC01711 | 0.113161 | 0.777358 | 2.780196 | 2.62E-15 | 2.59E-14 |
| ATP2A1-AS1 | 0.052521 | 0.139021 | 1.40434 | 5.01E-13 | 3.74E-12 |
| AL133492.1 | 0.008637 | 0.045416 | 2.394534 | 0.005073 | 0.008779 |
| MEI1 | 0.301178 | 0.632523 | 1.070501 | 3.55E-15 | 3.44E-14 |
| IGHJ4 | 3.938639 | 9.966436 | 1.33938 | 4.14E-06 | 1.25E-05 |
| AC005865.2 | 0.207865 | 0.42827 | 1.042874 | 9.43E-09 | 4.07E-08 |
| CIBAR2 | 0.058193 | 0.152441 | 1.389322 | 2.48E-06 | 7.70E-06 |
| TNNI3 | 0.022581 | 0.146813 | 2.700787 | 7.51E-09 | 3.29E-08 |
| GBA3 | 41.22835 | 20.1243 | -1.0347 | 5.95E-23 | 1.67E-21 |
| DIPK1C | 0.109595 | 0.247629 | 1.175999 | 0.003601 | 0.006428 |
| LINC01822 | 0.033688 | 0.009475 | -1.83001 | 2.45E-09 | 1.15E-08 |
| OTX1 | 0.024002 | 0.074081 | 1.625956 | 6.43E-06 | 1.88E-05 |
| ELFN1-AS1 | 0.039111 | 0.189286 | 2.274922 | 0.000724 | 0.001478 |
| CHST4 | 0.031686 | 0.067424 | 1.089426 | 3.24E-07 | 1.14E-06 |
| RPS20P2 | 0.099529 | 0.26202 | 1.396488 | 6.83E-06 | 1.98E-05 |
| AC024270.1 | 0.189429 | 0.088759 | -1.0937 | 0.004926 | 0.008552 |
| TUB-AS1 | 0.046155 | 0.017506 | -1.39865 | 3.22E-14 | 2.77E-13 |
| CXCR3 | 2.402711 | 4.849602 | 1.013203 | 4.15E-12 | 2.75E-11 |
| AC110792.2 | 0.050797 | 0.023496 | -1.11232 | 0.010369 | 0.016829 |
| LRIT2 | 0.030503 | 0.081508 | 1.417964 | 3.62E-05 | 9.33E-05 |
| AC068228.3 | 0.113108 | 0.262444 | 1.214313 | 0.022709 | 0.034109 |
| NKX3-2 | 0.025593 | 0.088448 | 1.789075 | 2.50E-05 | 6.61E-05 |
| LINC01579 | 0.00633 | 0.016756 | 1.404309 | 0.005529 | 0.009499 |
| TAF9BP1 | 0.079203 | 0.034653 | -1.19256 | 1.39E-07 | 5.16E-07 |
| MCIDAS | 0.021314 | 0.063312 | 1.570642 | 2.02E-07 | 7.33E-07 |
| TCN1 | 0.15668 | 1.393476 | 3.152794 | 5.11E-08 | 2.01E-07 |
| DLGAP5 | 0.715637 | 1.441582 | 1.010354 | 3.56E-14 | 3.05E-13 |
| C8G | 0.716702 | 1.521093 | 1.085663 | 5.18E-06 | 1.53E-05 |
| COL1A1 | 69.37055 | 192.9404 | 1.47576 | 5.69E-15 | 5.38E-14 |
| LINC00582 | 0.109618 | 0.276897 | 1.336864 | 0.00012 | 0.000283 |
| AC109809.1 | 0.041651 | 0.086115 | 1.047925 | 0.000239 | 0.000535 |
| TRHDE | 2.478045 | 1.19742 | -1.04927 | 3.70E-24 | 1.27E-22 |
| AL031289.2 | 0.012201 | 0.027799 | 1.188054 | 0.005424 | 0.009333 |
| KLK5 | 0.024304 | 0.073598 | 1.598475 | 5.62E-07 | 1.92E-06 |
| PKHD1 | 5.60114 | 2.619429 | -1.09647 | 8.81E-28 | 5.62E-26 |
| CCL19 | 3.457308 | 12.72696 | 1.880167 | 9.99E-13 | 7.16E-12 |
| IGHV4-34 | 13.48443 | 43.99136 | 1.705926 | 1.86E-05 | 5.01E-05 |
| B4GALNT4 | 0.549267 | 1.195486 | 1.122018 | 5.49E-10 | 2.82E-09 |
| MIR3945HG | 0.030715 | 0.062516 | 1.025258 | 0.009484 | 0.015527 |
| COL11A1 | 0.355743 | 1.925423 | 2.436269 | 2.16E-08 | 8.92E-08 |
| SLC12A5-AS1 | 0.025107 | 0.057805 | 1.203122 | 1.58E-07 | 5.80E-07 |
| RN7SL127P | 0.170472 | 0.078901 | -1.11143 | 0.02047 | 0.031092 |
| CNR2 | 0.04908 | 0.118883 | 1.276329 | 0.000174 | 0.000398 |
| PITX3 | 0.010003 | 0.0329 | 1.717592 | 1.81E-06 | 5.74E-06 |
| ZAN | 0.020792 | 0.077861 | 1.904865 | 0.025346 | 0.037692 |
| AC026780.1 | 0.125674 | 0.056986 | -1.14099 | 5.18E-12 | 3.40E-11 |
| AL160408.1 | 0.054185 | 0.022489 | -1.26869 | 0.005451 | 0.009377 |
| LYNX1-SLURP2 | 0.019068 | 0.040808 | 1.097678 | 2.18E-05 | 5.82E-05 |
| GTSF1L | 0.084555 | 0.190852 | 1.1745 | 4.08E-05 | 0.000104 |
| IGLC7 | 3.373598 | 10.24365 | 1.602369 | 1.59E-08 | 6.65E-08 |
| C17orf98 | 0.054092 | 0.148447 | 1.456454 | 0.007258 | 0.012159 |
| MIR7-3HG | 0.009467 | 0.032495 | 1.77933 | 0.000755 | 0.001537 |
| CTXND1 | 0.651939 | 0.281556 | -1.21131 | 0.000105 | 0.00025 |
| MTNR1A | 0.119956 | 0.053548 | -1.16359 | 0.004692 | 0.008174 |
| AL359314.1 | 0.327875 | 0.156917 | -1.06314 | 0.001338 | 0.002596 |
| SLC7A3 | 0.005973 | 0.023998 | 2.006319 | 7.93E-05 | 0.000193 |
| TRBV8-2 | 0.164583 | 0.41085 | 1.319795 | 5.40E-07 | 1.85E-06 |
| AURKB | 0.833454 | 2.376894 | 1.511903 | 8.63E-29 | 6.63E-27 |
| DIO2 | 0.596636 | 1.361095 | 1.189845 | 0.000144 | 0.000335 |
| ODAPH | 0.052324 | 0.137424 | 1.39309 | 2.61E-09 | 1.22E-08 |
| EVX1 | 0.021095 | 0.047617 | 1.174595 | 3.07E-08 | 1.24E-07 |
| RNA5SP46 | 0.248726 | 0.087877 | -1.50099 | 3.78E-15 | 3.65E-14 |
| ATP13A4 | 0.74109 | 0.368886 | -1.00647 | 9.45E-10 | 4.70E-09 |
| IGHV1-67 | 0.266457 | 0.834664 | 1.647295 | 0.001443 | 0.00278 |
| C1QL1 | 9.312923 | 39.25661 | 2.075629 | 6.88E-20 | 1.26E-18 |
| PITX1 | 0.225351 | 0.816764 | 1.857744 | 2.03E-14 | 1.79E-13 |
| LINC02416 | 0.128564 | 0.300417 | 1.224479 | 0.000716 | 0.001464 |
| DLX4 | 0.197727 | 0.44066 | 1.156157 | 7.47E-21 | 1.56E-19 |
| IGKV6D-21 | 1.207889 | 2.831644 | 1.229152 | 0.000824 | 0.001664 |
| RNA5SP425 | 0.206911 | 0.484429 | 1.227272 | 7.75E-05 | 0.000189 |
| AC010931.1 | 0.014571 | 0.035679 | 1.291965 | 0.024648 | 0.036736 |
| AC083822.1 | 0.087465 | 0.186119 | 1.089452 | 0.013119 | 0.020826 |
| SDR16C5 | 0.016628 | 0.055954 | 1.750651 | 9.97E-05 | 0.000238 |
| ZNF587P1 | 0.039879 | 0.096 | 1.267423 | 0.018923 | 0.028962 |
| PEBP1P3 | 0.074047 | 0.149799 | 1.016517 | 0.005192 | 0.008967 |
| RPL23AP77 | 0.073941 | 0.277751 | 1.909341 | 7.37E-14 | 6.08E-13 |
| PTPRN | 0.419274 | 1.860466 | 2.149699 | 1.40E-07 | 5.19E-07 |
| IGF2BP2-AS1 | 0.015782 | 0.033983 | 1.106506 | 0.000753 | 0.001533 |
| AC139491.5 | 0.052557 | 0.019446 | -1.43443 | 0.000212 | 0.000478 |
| BIRC5 | 1.30831 | 3.350194 | 1.35654 | 5.07E-27 | 2.80E-25 |
| AC083805.1 | 0.012712 | 0.028767 | 1.178216 | 0.004607 | 0.008038 |
| AC005906.3 | 0.054636 | 0.200318 | 1.874368 | 2.23E-09 | 1.05E-08 |
| ADAMDEC1 | 1.809481 | 3.854581 | 1.090998 | 1.98E-05 | 5.32E-05 |
| CD7 | 2.123074 | 6.338866 | 1.57807 | 5.50E-20 | 1.02E-18 |
| LINC01357 | 0.024714 | 0.062349 | 1.335064 | 3.37E-05 | 8.72E-05 |
| AC020658.4 | 0.015773 | 0.034715 | 1.138093 | 7.47E-07 | 2.51E-06 |
| MLANA | 1.236018 | 0.154417 | -3.0008 | 3.14E-09 | 1.45E-08 |
| LINC01281 | 0.02599 | 0.054913 | 1.079197 | 5.84E-09 | 2.60E-08 |
| PITX2 | 0.319106 | 0.685406 | 1.102922 | 1.74E-06 | 5.54E-06 |
| IGHV5-51 | 36.49846 | 83.70434 | 1.197467 | 0.000417 | 0.000893 |
| IGHV4-39 | 24.87796 | 99.56146 | 2.000719 | 1.28E-05 | 3.55E-05 |
| LINC01482 | 0.032801 | 0.067723 | 1.045888 | 0.000834 | 0.001684 |
| AL035446.1 | 0.291478 | 0.641855 | 1.138861 | 2.17E-06 | 6.80E-06 |
| IGLV8-61 | 13.71175 | 29.21125 | 1.091112 | 0.000535 | 0.001123 |
| LINC01502 | 0.013335 | 0.047811 | 1.84209 | 0.001405 | 0.002714 |
| CALY | 0.025785 | 0.272349 | 3.400847 | 3.51E-13 | 2.68E-12 |
| KCNN4 | 0.587282 | 1.440147 | 1.294092 | 2.74E-15 | 2.70E-14 |
| OR2H2 | 0.007582 | 0.016032 | 1.080237 | 0.009023 | 0.014843 |
| SYNGR4 | 0.084348 | 0.192092 | 1.187369 | 1.91E-09 | 9.11E-09 |
| PRSS57 | 0.116394 | 0.243441 | 1.064557 | 3.44E-06 | 1.05E-05 |
| NALCN-AS1 | 0.018661 | 0.047622 | 1.351579 | 5.25E-08 | 2.06E-07 |
| AC002378.1 | 0.020437 | 0.044363 | 1.118191 | 0.001916 | 0.003604 |
| WFDC3 | 0.230529 | 0.622091 | 1.432179 | 7.34E-16 | 7.79E-15 |
| AP005131.1 | 0.111146 | 0.042689 | -1.38053 | 6.78E-06 | 1.97E-05 |
| MISP | 0.98509 | 2.810077 | 1.512282 | 4.16E-13 | 3.14E-12 |
| C4orf48 | 1.226224 | 4.201319 | 1.77662 | 2.38E-30 | 2.59E-28 |
| RNU6-62P | 0.734196 | 0.299331 | -1.29442 | 3.89E-20 | 7.37E-19 |
| TRIM54 | 1.128779 | 3.121194 | 1.467334 | 2.01E-11 | 1.23E-10 |
| AC007993.3 | 0.018642 | 0.040046 | 1.103141 | 0.010802 | 0.017462 |
| GRP | 0.097143 | 0.497088 | 2.355318 | 7.85E-11 | 4.46E-10 |
| AC004917.1 | 0.017987 | 0.045528 | 1.339813 | 3.78E-05 | 9.70E-05 |
| CCDC190 | 0.01118 | 0.029488 | 1.399272 | 0.018583 | 0.028482 |
| HIGD2AP2 | 0.060111 | 0.166275 | 1.467871 | 0.030683 | 0.044831 |
| MSMB | 0.037662 | 0.18443 | 2.291884 | 0.001798 | 0.003398 |
| AC114316.1 | 0.295826 | 0.131232 | -1.17262 | 1.73E-11 | 1.07E-10 |
| IGKV1D-43 | 0.190623 | 1.080495 | 2.502898 | 7.05E-05 | 0.000173 |
| SLC22A12 | 24.22384 | 9.606389 | -1.33436 | 3.15E-17 | 4.02E-16 |
| LINC02576 | 0.084969 | 0.177797 | 1.065224 | 0.005572 | 0.009566 |
| DGAT2L7P | 0.086076 | 0.225892 | 1.391948 | 0.007551 | 0.012604 |
| MTTP | 0.398831 | 0.943908 | 1.242869 | 0.000219 | 0.000494 |
| CD79A | 2.635947 | 8.209128 | 1.638908 | 1.37E-11 | 8.57E-11 |
| AC104088.3 | 0.201631 | 0.092191 | -1.12902 | 5.95E-15 | 5.62E-14 |
| PTGER1 | 0.144659 | 0.428044 | 1.565103 | 9.68E-11 | 5.44E-10 |
| AC138625.1 | 0.049127 | 0.150648 | 1.61658 | 9.93E-05 | 0.000237 |
| LINC02577 | 0.230888 | 0.83928 | 1.861956 | 4.37E-15 | 4.18E-14 |
| EGFL6 | 0.18165 | 1.697163 | 3.223893 | 7.36E-09 | 3.23E-08 |
| S100G | 0.438744 | 2.862242 | 2.705696 | 6.63E-06 | 1.93E-05 |
| ZNF350-AS1 | 0.742948 | 0.294099 | -1.33696 | 0.000166 | 0.000382 |
| AC103563.3 | 0.172933 | 0.386852 | 1.161569 | 0.001967 | 0.003691 |
| NCAN | 0.008288 | 0.022994 | 1.472233 | 7.04E-06 | 2.04E-05 |
| FMR1NB | 0.014057 | 0.096511 | 2.779434 | 0.002154 | 0.004008 |
| PYCARD | 4.757552 | 10.57672 | 1.1526 | 5.01E-34 | 1.25E-31 |
| KRT8P30 | 0.031128 | 0.086152 | 1.468678 | 5.48E-07 | 1.88E-06 |
| MYO1A | 0.08007 | 0.180554 | 1.173091 | 7.27E-06 | 2.10E-05 |
| AL138889.1 | 0.004132 | 0.008456 | 1.033044 | 1.76E-05 | 4.76E-05 |
| IMPDH1P5 | 0.114174 | 0.267207 | 1.226726 | 0.00077 | 0.001564 |
| ENAM | 1.999829 | 0.755995 | -1.40343 | 4.02E-25 | 1.61E-23 |
| AC006974.2 | 0.013867 | 0.028723 | 1.050614 | 0.0025 | 0.004593 |
| WFDC13 | 0.0248 | 0.063174 | 1.34899 | 1.49E-06 | 4.79E-06 |
| AC007362.1 | 0.086861 | 0.040276 | -1.10878 | 0.000112 | 0.000265 |
| ANKRD36BP2 | 0.172464 | 0.390643 | 1.179555 | 0.009597 | 0.015687 |
| AC024610.2 | 0.004456 | 0.017164 | 1.945552 | 9.65E-05 | 0.000231 |
| AC103702.2 | 1.28894 | 3.004364 | 1.220874 | 6.85E-10 | 3.47E-09 |
| C6orf118 | 0.01604 | 0.039657 | 1.305867 | 1.31E-09 | 6.38E-09 |
| AP005131.4 | 0.103305 | 0.043744 | -1.23974 | 0.025206 | 0.037504 |
| CHST6 | 0.02858 | 0.082142 | 1.523119 | 1.51E-09 | 7.31E-09 |
| AC004687.3 | 0.00569 | 0.02455 | 2.10908 | 0.017764 | 0.02736 |
| SERPINB5 | 0.011612 | 0.110092 | 3.245034 | 0.001033 | 0.002048 |
| LINC01121 | 0.005712 | 0.023002 | 2.00974 | 5.92E-06 | 1.74E-05 |
| AC097658.3 | 0.058045 | 0.019961 | -1.53999 | 0.00527 | 0.009088 |
| SLC5A1 | 17.14138 | 7.700241 | -1.15451 | 1.46E-11 | 9.08E-11 |
| GFAP | 0.145627 | 0.315965 | 1.117491 | 4.60E-09 | 2.08E-08 |
| AC092675.1 | 0.021129 | 0.125122 | 2.56607 | 2.37E-09 | 1.12E-08 |
| AL357497.1 | 0.07987 | 0.244518 | 1.61421 | 0.000324 | 0.000707 |
| DNER | 0.850909 | 2.459656 | 1.53138 | 3.02E-08 | 1.22E-07 |
| LY6H | 0.870978 | 1.791958 | 1.040828 | 1.95E-05 | 5.26E-05 |
| NBPF4 | 0.00807 | 0.016747 | 1.053185 | 0.000723 | 0.001476 |
| SEC61G-DT | 0.049854 | 0.12895 | 1.371039 | 1.71E-09 | 8.20E-09 |
| AC007569.1 | 0.092918 | 0.193201 | 1.05607 | 0.000537 | 0.001128 |
| AC099066.2 | 0.054856 | 0.138967 | 1.341022 | 1.37E-10 | 7.59E-10 |
| TENM3 | 0.345754 | 0.952457 | 1.46191 | 9.76E-11 | 5.48E-10 |
| RPL22P2 | 0.215294 | 0.471925 | 1.132251 | 7.63E-06 | 2.20E-05 |
| IGLV2-11 | 25.32502 | 63.6592 | 1.329806 | 6.36E-06 | 1.86E-05 |
| DEPDC1P2 | 0.017046 | 0.007886 | -1.11198 | 1.43E-06 | 4.62E-06 |
| AC044802.2 | 0.03009 | 0.074104 | 1.300253 | 1.54E-05 | 4.22E-05 |
| LINC01507 | 4.112732 | 1.970151 | -1.06179 | 1.77E-32 | 3.20E-30 |
| CD300LG | 0.886065 | 0.36625 | -1.27458 | 1.28E-16 | 1.50E-15 |
| AL109741.2 | 0.178334 | 0.376546 | 1.078242 | 4.22E-08 | 1.68E-07 |
| AC093330.3 | 0.060186 | 0.028978 | -1.05448 | 0.00143 | 0.002758 |
| COL1A2 | 60.83814 | 123.2581 | 1.018634 | 5.56E-09 | 2.48E-08 |
| SLC38A3 | 0.214911 | 0.716183 | 1.736592 | 0.000417 | 0.000893 |
| ITGB1-DT | 0.06884 | 0.157514 | 1.194158 | 5.19E-11 | 3.03E-10 |
| AL356234.3 | 0.156158 | 0.486158 | 1.638415 | 9.25E-07 | 3.06E-06 |
| AC113346.1 | 0.184619 | 0.673644 | 1.867432 | 5.12E-18 | 7.29E-17 |
| AC098483.2 | 0.104422 | 0.037252 | -1.48703 | 5.57E-06 | 1.64E-05 |
| RPL10P8 | 0.135019 | 0.278323 | 1.043598 | 3.50E-05 | 9.04E-05 |
| NIPAL4 | 0.084281 | 0.249713 | 1.566992 | 9.95E-13 | 7.13E-12 |
| C17orf64 | 0.095602 | 0.248029 | 1.375397 | 5.85E-08 | 2.28E-07 |
| STMN2 | 0.146742 | 1.453862 | 3.308537 | 6.72E-10 | 3.41E-09 |
| PRKCG | 0.016986 | 0.077307 | 2.186269 | 2.38E-06 | 7.43E-06 |
| OR10A2 | 0.015657 | 0.037541 | 1.261631 | 0.026558 | 0.039307 |
| MT2A | 138.9159 | 290.4076 | 1.063867 | 9.12E-20 | 1.63E-18 |
| AP005212.4 | 0.025921 | 0.075943 | 1.550776 | 0.001501 | 0.00288 |
| FLNC | 1.286896 | 4.089967 | 1.668194 | 2.86E-09 | 1.33E-08 |
| AC004000.1 | 0.220307 | 0.444436 | 1.012463 | 5.01E-08 | 1.97E-07 |
| LINC00922 | 0.007628 | 0.290127 | 5.249197 | 3.35E-10 | 1.77E-09 |
| RNU6-1176P | 0.309051 | 0.135703 | -1.18739 | 0.021605 | 0.032617 |
| EPO | 4.252291 | 10.96466 | 1.366549 | 0.023808 | 0.035617 |
| FAM133A | 0.017269 | 0.203965 | 3.562041 | 0.000924 | 0.001849 |
| C12orf50 | 0.007832 | 0.016741 | 1.095997 | 0.00237 | 0.004373 |
| EN1 | 0.117356 | 0.274174 | 1.224201 | 6.75E-11 | 3.87E-10 |
| AC092574.2 | 0.146042 | 0.070486 | -1.05096 | 1.81E-06 | 5.76E-06 |
| MZB1 | 2.496118 | 8.197752 | 1.715542 | 5.27E-12 | 3.46E-11 |
| FGF8 | 0.019439 | 0.081715 | 2.071617 | 0.010504 | 0.017024 |
| AC104339.1 | 0.113381 | 0.314618 | 1.472429 | 1.79E-05 | 4.86E-05 |
| PADI3 | 0.250395 | 1.973873 | 2.97875 | 6.29E-10 | 3.21E-09 |
| RNU4-62P | 1.125148 | 3.126538 | 1.474451 | 2.56E-06 | 7.95E-06 |
| LINC01305 | 0.00704 | 0.042341 | 2.5884 | 0.011141 | 0.017964 |
| RNASEH2A | 0.021456 | 0.048592 | 1.179365 | 0.008189 | 0.013574 |
| RPL18AP15 | 0.018568 | 0.041012 | 1.143212 | 0.00015 | 0.000347 |
| AC073316.1 | 0.039973 | 0.108008 | 1.434018 | 0.016513 | 0.025608 |
| CPXM1 | 1.694847 | 5.035292 | 1.57092 | 2.04E-15 | 2.05E-14 |
| AC018695.6 | 0.393433 | 0.903614 | 1.199588 | 4.92E-09 | 2.21E-08 |
| AL359538.1 | 0.013725 | 0.047008 | 1.776148 | 0.005563 | 0.009553 |
| HSPB3 | 0.049006 | 0.412953 | 3.074935 | 0.004252 | 0.007474 |
| POU3F2 | 0.009746 | 0.0227 | 1.219812 | 0.002056 | 0.00384 |
| ALDH1L1 | 9.251294 | 4.257187 | -1.11975 | 1.36E-25 | 5.98E-24 |
| TPX2 | 2.888638 | 6.429707 | 1.154364 | 2.75E-21 | 6.07E-20 |
| UNC5B-AS1 | 0.27738 | 0.564616 | 1.025404 | 1.93E-11 | 1.18E-10 |
| LINC02225 | 0.008434 | 0.02382 | 1.497845 | 3.13E-05 | 8.14E-05 |
| CSF2 | 0.042954 | 0.121389 | 1.498765 | 5.06E-10 | 2.61E-09 |
| COL8A2 | 1.478308 | 3.210894 | 1.119029 | 3.82E-09 | 1.74E-08 |
| CCL26 | 0.287717 | 1.129317 | 1.972728 | 1.81E-16 | 2.08E-15 |
| ANLN | 1.365364 | 3.043907 | 1.156639 | 3.34E-12 | 2.24E-11 |
| AC022092.1 | 0.050209 | 0.147333 | 1.553063 | 6.94E-10 | 3.52E-09 |
| LINC02389 | 0.003621 | 0.008611 | 1.249905 | 0.032296 | 0.046915 |
| CPB2 | 0.029348 | 0.122161 | 2.057452 | 0.002399 | 0.004422 |
| AC005622.1 | 0.018126 | 0.053987 | 1.574527 | 8.86E-06 | 2.53E-05 |
| IGKV3D-11 | 1.27104 | 3.938674 | 1.6317 | 1.38E-05 | 3.81E-05 |
| SPC24 | 0.890267 | 2.127471 | 1.256829 | 1.25E-28 | 9.21E-27 |
| AC061965.2 | 0.013816 | 0.006694 | -1.04537 | 6.71E-09 | 2.96E-08 |
| SFRP4 | 2.99048 | 6.191002 | 1.049796 | 9.45E-07 | 3.13E-06 |
| CYP17A1 | 3.057957 | 0.176014 | -4.1188 | 2.10E-06 | 6.60E-06 |
| HSD11B1 | 1.165581 | 2.804012 | 1.266444 | 1.65E-06 | 5.29E-06 |
| SOX1 | 0.008311 | 0.054962 | 2.725402 | 7.60E-05 | 0.000185 |
| VIL1 | 5.335402 | 2.557268 | -1.06099 | 1.33E-09 | 6.47E-09 |
| AC005586.1 | 0.35666 | 0.785622 | 1.139286 | 2.27E-12 | 1.55E-11 |
| GOT2P7 | 0.036668 | 0.01437 | -1.35143 | 0.000195 | 0.000443 |
| AC107419.1 | 0.006868 | 0.016709 | 1.282603 | 0.004568 | 0.007977 |
| AC073592.8 | 0.018752 | 0.037923 | 1.016045 | 7.05E-05 | 0.000173 |
| HBA1 | 0.328982 | 1.101625 | 1.743554 | 0.0191 | 0.029201 |
| RN7SKP292 | 0.142063 | 0.069018 | -1.04148 | 0.012609 | 0.020095 |
| EPHB2 | 0.441805 | 0.919912 | 1.058085 | 1.82E-07 | 6.64E-07 |
| LINC00906 | 0.238385 | 0.105778 | -1.17225 | 0.006939 | 0.011668 |
| GABRA3 | 0.019812 | 0.184345 | 3.21793 | 0.006175 | 0.010496 |
| RPS23P6 | 0.518026 | 1.105064 | 1.093034 | 7.89E-07 | 2.64E-06 |
| SLC22A31 | 0.033758 | 0.088475 | 1.390037 | 5.15E-09 | 2.31E-08 |
| CCDC26 | 0.006196 | 0.014842 | 1.260296 | 0.01082 | 0.017487 |
| AC105118.1 | 0.16229 | 0.445447 | 1.456678 | 4.17E-06 | 1.25E-05 |
| TEX19 | 0.010084 | 0.065674 | 2.703323 | 8.23E-11 | 4.66E-10 |
| IGLV3-27 | 2.73419 | 6.305806 | 1.205567 | 0.000323 | 0.000706 |
| TBX18-AS1 | 0.018084 | 0.036852 | 1.026978 | 0.001221 | 0.002385 |
| AC096751.2 | 0.041388 | 0.088506 | 1.096568 | 7.47E-06 | 2.15E-05 |
| AC092078.3 | 0.165339 | 0.047447 | -1.80103 | 1.65E-07 | 6.03E-07 |
| LINC02109 | 0.006669 | 0.017978 | 1.430611 | 0.014574 | 0.02289 |
| SBSN | 0.043759 | 0.679226 | 3.956231 | 9.77E-16 | 1.02E-14 |
| AGAP2-AS1 | 0.307253 | 0.68015 | 1.146426 | 6.97E-24 | 2.27E-22 |
| GCKR | 0.105743 | 0.270246 | 1.353707 | 1.41E-10 | 7.79E-10 |
| RN7SL726P | 0.302091 | 0.10776 | -1.48716 | 6.62E-19 | 1.06E-17 |
| AL021920.2 | 0.126007 | 0.062179 | -1.01901 | 1.62E-06 | 5.18E-06 |
| SNORA79B | 4.359134 | 0.535116 | -3.02612 | 1.65E-07 | 6.06E-07 |
| AC022395.1 | 0.084157 | 0.03842 | -1.13125 | 1.68E-15 | 1.70E-14 |
| LINC02257 | 0.046151 | 0.192668 | 2.061673 | 8.04E-06 | 2.31E-05 |
| SLC52A1 | 0.132078 | 0.345279 | 1.386366 | 1.20E-12 | 8.50E-12 |
| AC018742.1 | 2.275479 | 1.027673 | -1.14679 | 3.54E-09 | 1.62E-08 |
| AL596442.4 | 1.230406 | 0.551032 | -1.15893 | 1.59E-11 | 9.89E-11 |
| ADGRF5-AS1 | 0.047123 | 0.020621 | -1.19233 | 0.029205 | 0.042874 |
| FAM225A | 0.059338 | 0.130743 | 1.139708 | 2.32E-22 | 6.01E-21 |
| LRP2 | 50.96494 | 22.82521 | -1.15888 | 6.73E-25 | 2.64E-23 |
| AC015818.2 | 0.052147 | 0.193485 | 1.891556 | 7.31E-12 | 4.73E-11 |
| RNU1-56P | 0.093722 | 0.220692 | 1.235578 | 0.002325 | 0.004295 |
| AC005871.1 | 0.092105 | 0.035733 | -1.36601 | 1.83E-10 | 9.96E-10 |
| IGLV4-69 | 7.777345 | 69.13415 | 3.152049 | 2.81E-05 | 7.37E-05 |
| VGF | 0.080552 | 0.403005 | 2.322798 | 1.02E-07 | 3.87E-07 |
| TLX2 | 0.004879 | 0.027367 | 2.487922 | 0.000201 | 0.000456 |
| TMEM174 | 13.78922 | 6.345273 | -1.11979 | 2.91E-15 | 2.86E-14 |
| MORF4L1P3 | 0.043205 | 0.020759 | -1.05747 | 0.011828 | 0.018962 |
| AL049775.1 | 0.012129 | 0.025356 | 1.063822 | 0.002452 | 0.004511 |
| AC022730.1 | 0.026793 | 0.085644 | 1.676484 | 0.000151 | 0.00035 |
| AC012213.2 | 0.029642 | 0.059563 | 1.006775 | 0.003727 | 0.006629 |
| MIR507 | 0.302637 | 0.142142 | -1.09026 | 0.000138 | 0.000322 |
| RPL7P38 | 0.028556 | 0.059997 | 1.071096 | 0.011197 | 0.018045 |
| AL390774.2 | 0.051872 | 0.018492 | -1.48806 | 0.015388 | 0.024031 |
| AC003070.2 | 0.076563 | 0.225587 | 1.558961 | 0.007854 | 0.013067 |
| HAS2 | 0.254853 | 0.586079 | 1.201431 | 9.08E-07 | 3.01E-06 |
| GFPT2 | 1.461741 | 5.070872 | 1.794546 | 1.80E-21 | 4.09E-20 |
| AC011944.2 | 0.122396 | 0.046313 | -1.40206 | 1.83E-05 | 4.94E-05 |
| AL035251.1 | 0.006322 | 0.013176 | 1.059467 | 0.032451 | 0.047116 |
| AC004847.1 | 0.635439 | 1.332545 | 1.068359 | 4.59E-11 | 2.70E-10 |
| IGHV1-18 | 26.91837 | 91.48706 | 1.764976 | 0.000413 | 0.000887 |
| AC078909.2 | 0.116296 | 0.248917 | 1.097867 | 4.67E-06 | 1.39E-05 |
| IGHD | 5.393377 | 11.60257 | 1.105184 | 0.002293 | 0.004241 |
| ESPNP | 0.147566 | 0.062527 | -1.23881 | 2.34E-09 | 1.10E-08 |
| OR7E100P | 0.036259 | 0.08677 | 1.258861 | 0.011108 | 0.017914 |
| CENPA | 0.437121 | 1.096544 | 1.32686 | 5.15E-21 | 1.10E-19 |
| LDLRAD4-AS1 | 0.082549 | 0.041195 | -1.00279 | 0.001451 | 0.002794 |
| COMP | 0.900877 | 3.370707 | 1.903649 | 1.99E-10 | 1.08E-09 |
| IGHV3-11 | 15.84194 | 33.3907 | 1.075697 | 0.0002 | 0.000452 |
| SPATA3-AS1 | 0.009064 | 0.020462 | 1.174724 | 3.47E-05 | 8.96E-05 |
| RPS26P8 | 0.181255 | 0.490827 | 1.437197 | 0.004228 | 0.007436 |
| ADH4 | 0.193238 | 1.43609 | 2.893697 | 0.017478 | 0.026956 |
| AC025419.1 | 0.010451 | 0.026376 | 1.335598 | 7.48E-06 | 2.16E-05 |
| PCOLCE | 5.892242 | 12.85027 | 1.12491 | 7.33E-20 | 1.33E-18 |
| AC011752.1 | 0.169016 | 0.051204 | -1.72284 | 4.70E-11 | 2.76E-10 |
| MAGEC3 | 0.016007 | 0.19168 | 3.581904 | 0.018571 | 0.028467 |
| LGI1 | 0.011952 | 0.03708 | 1.633332 | 0.000285 | 0.000629 |
| PLA2G2D | 0.592408 | 1.926795 | 1.70154 | 1.24E-06 | 4.05E-06 |
| RPSAP52 | 0.016967 | 0.135093 | 2.99316 | 0.010756 | 0.01739 |
| AC010907.2 | 0.115211 | 0.024598 | -2.22767 | 0.00735 | 0.012298 |
| PRELID1P2 | 0.10912 | 0.04072 | -1.42212 | 5.39E-10 | 2.78E-09 |
| AC008991.1 | 0.60169 | 0.256751 | -1.22865 | 0.001265 | 0.002465 |
| HS3ST3A1 | 0.120132 | 0.801991 | 2.738965 | 1.10E-05 | 3.09E-05 |
| IGHV3-30 | 22.1401 | 89.52661 | 2.015655 | 0.000372 | 0.000804 |
| CSMD3 | 0.003745 | 0.016095 | 2.103387 | 0.000787 | 0.001596 |
| GNG8 | 0.258657 | 0.611377 | 1.241023 | 8.19E-10 | 4.11E-09 |
| AC002401.4 | 0.469019 | 1.246141 | 1.409747 | 7.26E-12 | 4.70E-11 |
| KLF17 | 0.005782 | 0.031132 | 2.428642 | 4.89E-11 | 2.86E-10 |
| PDPN | 0.878045 | 1.935906 | 1.140643 | 1.32E-08 | 5.59E-08 |
| CCL5 | 36.99394 | 74.39011 | 1.007822 | 7.09E-14 | 5.86E-13 |
| AC124067.2 | 0.132052 | 0.305348 | 1.209348 | 1.96E-10 | 1.06E-09 |
| HJURP | 0.636363 | 1.60743 | 1.336834 | 4.19E-22 | 1.04E-20 |
| OPN4 | 0.127472 | 0.260239 | 1.029657 | 0.029141 | 0.042793 |
| ATP11AUN | 0.259498 | 0.030786 | -3.07539 | 8.45E-07 | 2.82E-06 |
| HNRNPRP1 | 0.037799 | 0.017269 | -1.13017 | 0.009504 | 0.015552 |
| LINC01060 | 0.644334 | 0.285334 | -1.17516 | 5.03E-14 | 4.22E-13 |
| GMPSP1 | 0.097089 | 0.201726 | 1.055019 | 1.48E-08 | 6.24E-08 |
| LINC00460 | 0.102102 | 0.560093 | 2.455654 | 3.86E-20 | 7.29E-19 |
| CDH15 | 0.085243 | 0.192967 | 1.178695 | 1.79E-13 | 1.41E-12 |
| AP002755.1 | 0.224035 | 0.090939 | -1.30075 | 0.000109 | 0.00026 |
| ADGRF4 | 0.196842 | 0.408989 | 1.055028 | 0.003773 | 0.006706 |
| CNTNAP5 | 0.795402 | 0.305039 | -1.38269 | 0.013349 | 0.021156 |
| GK-IT1 | 0.160398 | 0.361235 | 1.171278 | 0.000188 | 0.000429 |
| CHST8 | 0.039997 | 0.096653 | 1.272927 | 0.000373 | 0.000807 |
| AL356234.1 | 0.058185 | 0.216138 | 1.893243 | 0.003419 | 0.00613 |
| AC009236.1 | 0.059349 | 0.176339 | 1.571045 | 2.82E-07 | 1.00E-06 |
| AL022323.1 | 0.049087 | 0.023302 | -1.07489 | 0.001381 | 0.002671 |
| AC006262.1 | 0.049285 | 0.108798 | 1.14242 | 0.000262 | 0.000583 |
| GNA14-AS1 | 0.085555 | 0.197499 | 1.206921 | 0.008862 | 0.01459 |
| AC094019.2 | 0.159561 | 0.076497 | -1.06064 | 7.23E-09 | 3.17E-08 |
| TGM5 | 0.107445 | 0.288736 | 1.426148 | 5.67E-09 | 2.53E-08 |
| AC024884.2 | 0.025065 | 0.054109 | 1.110232 | 1.45E-06 | 4.66E-06 |
| AC055733.4 | 0.019414 | 0.043006 | 1.14743 | 0.004434 | 0.007762 |
| AC099314.1 | 0.023044 | 0.051232 | 1.152667 | 0.006391 | 0.01083 |
| AL807761.4 | 0.025497 | 0.011755 | -1.11704 | 0.002535 | 0.004654 |
| AC239799.2 | 0.016264 | 0.041602 | 1.35496 | 0.00018 | 0.000411 |
| AC025176.1 | 0.142474 | 0.287713 | 1.013934 | 0.001856 | 0.0035 |
| AC005100.1 | 0.023245 | 0.070796 | 1.606722 | 2.74E-09 | 1.28E-08 |
| LINC00973 | 0.036375 | 0.709452 | 4.285701 | 5.90E-09 | 2.63E-08 |
| ZNF804B | 0.07028 | 0.029189 | -1.26766 | 2.57E-06 | 7.98E-06 |
| MAB21L3 | 1.051101 | 0.508253 | -1.04828 | 0.001538 | 0.002947 |
| TMEM252 | 16.59385 | 7.683776 | -1.11076 | 1.79E-23 | 5.44E-22 |
| AL021391.1 | 0.025701 | 0.059963 | 1.222229 | 0.003916 | 0.006937 |
| AC002310.2 | 0.007526 | 0.018042 | 1.261358 | 0.030278 | 0.044298 |
| AC110992.1 | 0.048125 | 0.023863 | -1.01201 | 0.022577 | 0.033933 |
| RPL12P42 | 0.128364 | 0.27065 | 1.076193 | 0.000445 | 0.000949 |
| SLC5A8 | 14.91404 | 6.588005 | -1.17876 | 6.68E-19 | 1.07E-17 |
| LINC01713 | 0.024191 | 0.053761 | 1.152056 | 0.015018 | 0.023509 |
| WDR72 | 10.49182 | 4.535308 | -1.20999 | 3.24E-32 | 5.52E-30 |
| NUP35P1 | 0.049148 | 0.021213 | -1.2122 | 0.000143 | 0.000332 |
| A1CF | 5.756628 | 2.701247 | -1.0916 | 3.17E-19 | 5.25E-18 |
| AC245041.2 | 0.031946 | 0.146806 | 2.200219 | 1.34E-07 | 4.96E-07 |
| AC091729.2 | 0.757349 | 0.283657 | -1.41681 | 0.00596 | 0.010159 |
| GUCY2D | 0.07026 | 0.178088 | 1.341803 | 3.11E-12 | 2.10E-11 |
| LINC00671 | 13.88368 | 6.924534 | -1.0036 | 1.32E-14 | 1.20E-13 |
| IGKV3D-20 | 2.74862 | 8.950648 | 1.703284 | 6.42E-05 | 0.000159 |
| ZP1 | 0.204652 | 0.507792 | 1.311067 | 4.83E-12 | 3.18E-11 |
| AC091286.1 | 0.223178 | 0.100014 | -1.158 | 1.22E-11 | 7.66E-11 |
| IGHM | 79.27578 | 223.6764 | 1.496461 | 4.29E-10 | 2.24E-09 |
| AF107885.2 | 0.031254 | 0.009163 | -1.7702 | 0.014707 | 0.023082 |
| NFE4 | 0.070113 | 0.962115 | 3.778462 | 0.007401 | 0.012373 |
| SPOCD1 | 0.091498 | 0.272394 | 1.573886 | 4.92E-17 | 6.11E-16 |
| AC026355.3 | 0.030456 | 0.193344 | 2.666356 | 0.000393 | 0.000847 |
| KCNK17 | 0.430202 | 0.881069 | 1.03424 | 1.10E-09 | 5.44E-09 |
| LHX8 | 0.128628 | 0.264606 | 1.040639 | 0.013263 | 0.021034 |
| PLAC9P1 | 0.056568 | 0.154394 | 1.448558 | 0.018919 | 0.028958 |
| FOXP3 | 0.635246 | 1.299065 | 1.032086 | 1.07E-13 | 8.67E-13 |
| IL21 | 0.012419 | 0.031014 | 1.320372 | 5.88E-07 | 2.00E-06 |
| RPL36AP26 | 0.115807 | 0.352745 | 1.606903 | 1.58E-05 | 4.33E-05 |
| AC053503.5 | 0.015285 | 0.039174 | 1.357758 | 0.008248 | 0.013662 |
| PDGFRL | 1.067028 | 2.77392 | 1.378328 | 6.64E-17 | 8.12E-16 |
| APOA1 | 0.304 | 3.406352 | 3.486086 | 5.37E-10 | 2.77E-09 |
| KLK10 | 0.028077 | 0.151286 | 2.429816 | 1.82E-08 | 7.58E-08 |
| TRAV34 | 0.087506 | 0.204873 | 1.227279 | 0.001419 | 0.002738 |
| AC007496.3 | 0.106892 | 0.030831 | -1.79369 | 0.003743 | 0.006656 |
| NAV3 | 0.138016 | 0.286013 | 1.05124 | 0.000191 | 0.000435 |
| RUFY4 | 0.181193 | 0.426773 | 1.235942 | 8.75E-05 | 0.000211 |
| AC097518.1 | 0.128981 | 0.334847 | 1.376344 | 0.002286 | 0.004231 |
| AC092306.1 | 0.154019 | 0.380522 | 1.304868 | 8.73E-05 | 0.00021 |
| AC124312.4 | 0.105222 | 0.051116 | -1.0416 | 1.07E-05 | 3.01E-05 |
| SIX3 | 0.020271 | 0.255098 | 3.653553 | 0.000453 | 0.000963 |
| MIR9-1HG | 0.014296 | 0.041198 | 1.526981 | 9.78E-05 | 0.000234 |
| SCAT1 | 0.040147 | 0.14371 | 1.83981 | 1.00E-08 | 4.32E-08 |
| PNPT1P1 | 0.014183 | 0.005477 | -1.37278 | 0.01309 | 0.020783 |
| ALLC | 0.123843 | 0.034492 | -1.84418 | 0.032948 | 0.047749 |
| HMGA1 | 9.497346 | 20.7539 | 1.127786 | 1.34E-10 | 7.38E-10 |
| AL139415.1 | 0.068854 | 0.141938 | 1.043647 | 0.000631 | 0.001308 |
| ADAM7 | 0.050832 | 0.59279 | 3.543701 | 0.000234 | 0.000525 |
| AC013549.1 | 0.091279 | 0.262483 | 1.523867 | 0.003765 | 0.006694 |
| SLC2A14 | 0.10382 | 0.21548 | 1.053467 | 0.000123 | 0.00029 |
| POM121L2 | 0.009184 | 0.019215 | 1.065069 | 0.001492 | 0.002865 |
| AF064858.2 | 5.75958 | 2.226619 | -1.37111 | 1.01E-16 | 1.20E-15 |
| AL157935.1 | 0.647542 | 0.27465 | -1.23738 | 0.000925 | 0.001851 |
| AC010255.2 | 0.025727 | 0.279057 | 3.439216 | 0.020337 | 0.030912 |
| BASP1-AS1 | 0.008737 | 0.02453 | 1.489338 | 0.02334 | 0.03498 |
| SPDEF | 0.219551 | 0.707249 | 1.687664 | 2.58E-05 | 6.79E-05 |
| ELANE | 0.108984 | 0.336401 | 1.626058 | 0.000126 | 0.000296 |
| MYO18B | 0.019011 | 0.051218 | 1.429802 | 0.009504 | 0.015552 |
| SLC22A6 | 19.2483 | 8.134553 | -1.2426 | 5.94E-10 | 3.04E-09 |
| CNGA3 | 0.004212 | 0.025592 | 2.603004 | 0.004207 | 0.007403 |
| AC107294.1 | 0.113443 | 0.317383 | 1.484255 | 0.010293 | 0.016719 |
| MUC12 | 0.071072 | 0.147329 | 1.051692 | 7.87E-14 | 6.47E-13 |
| P3H3 | 1.7326 | 5.028398 | 1.53716 | 8.30E-15 | 7.69E-14 |
| MIR8089 | 0.34336 | 0.152734 | -1.1687 | 1.25E-11 | 7.87E-11 |
| IGKV3-20 | 85.22822 | 262.5988 | 1.623457 | 2.10E-06 | 6.61E-06 |
| MYL10 | 0.015666 | 0.054685 | 1.803525 | 0.00015 | 0.000348 |
| ARHGEF7-AS1 | 0.055343 | 0.024185 | -1.1943 | 3.24E-10 | 1.71E-09 |
| LINC00896 | 0.060153 | 0.155468 | 1.369921 | 4.25E-06 | 1.27E-05 |
| AC021134.1 | 0.008459 | 0.032508 | 1.942296 | 0.021068 | 0.031902 |
| IL17C | 0.02619 | 0.053018 | 1.017448 | 0.00926 | 0.015199 |
| RTL1 | 0.004516 | 0.758005 | 7.391044 | 3.30E-06 | 1.01E-05 |
| COL6A3 | 8.21798 | 17.78118 | 1.113496 | 3.06E-08 | 1.23E-07 |
| TFAP2A-AS1 | 0.088335 | 0.213605 | 1.27388 | 8.84E-10 | 4.41E-09 |
| E2F7 | 0.239056 | 0.480072 | 1.005901 | 3.23E-05 | 8.39E-05 |
| TMEM40 | 0.018305 | 0.050908 | 1.47564 | 0.001967 | 0.003691 |
| AL356515.1 | 0.054551 | 0.18618 | 1.771026 | 1.10E-09 | 5.44E-09 |
| MS4A15 | 0.021271 | 0.0448 | 1.074645 | 0.002425 | 0.004466 |
| TNFRSF18 | 0.778578 | 1.886858 | 1.277073 | 9.02E-26 | 4.10E-24 |
| AL359313.1 | 0.011115 | 0.080291 | 2.852741 | 7.61E-14 | 6.27E-13 |
| DEPDC1-AS1 | 0.009812 | 0.025558 | 1.38113 | 0.021282 | 0.032179 |
| CPA5 | 0.02296 | 0.066932 | 1.543563 | 6.84E-13 | 4.99E-12 |
| TGFA-IT1 | 0.085103 | 0.029184 | -1.54402 | 0.000692 | 0.001421 |
| AL353801.1 | 1.479984 | 0.710994 | -1.05767 | 1.88E-17 | 2.47E-16 |
| CPA4 | 0.425864 | 2.682998 | 2.65538 | 5.21E-15 | 4.95E-14 |
| KRTAP2-3 | 0.046807 | 0.161027 | 1.782518 | 7.40E-13 | 5.38E-12 |
| FOXM1 | 1.297525 | 2.870543 | 1.145562 | 9.90E-14 | 8.07E-13 |
| FCER2 | 0.098643 | 0.387712 | 1.974691 | 0.012417 | 0.019818 |
| SCRT1 | 0.099608 | 0.011754 | -3.08314 | 3.61E-05 | 9.30E-05 |
| ATP4B | 0.112937 | 0.035876 | -1.65442 | 0.000525 | 0.001105 |
| AL445649.1 | 0.029813 | 0.067987 | 1.189306 | 0.013683 | 0.021631 |
| RPS2P6 | 0.021767 | 0.048776 | 1.164053 | 7.34E-05 | 0.000179 |
| LINC02454 | 0.029805 | 0.099805 | 1.743535 | 4.68E-11 | 2.75E-10 |
| CCDC63 | 0.008965 | 0.022711 | 1.340997 | 0.022302 | 0.033558 |
| AC110296.1 | 0.153901 | 0.023941 | -2.68445 | 0.011007 | 0.01777 |
| MMP7 | 35.11447 | 80.23313 | 1.192132 | 6.15E-11 | 3.55E-10 |
| TRBV2 | 0.799274 | 1.798411 | 1.169961 | 3.68E-06 | 1.11E-05 |
| SPDYC | 0.017339 | 0.040824 | 1.23537 | 0.009527 | 0.015583 |
| PAIP2B | 4.042617 | 1.744101 | -1.21281 | 4.17E-32 | 6.89E-30 |
| MMP12 | 0.520292 | 1.953956 | 1.909005 | 1.95E-07 | 7.10E-07 |
| AC010533.1 | 0.025456 | 0.052308 | 1.039022 | 3.38E-05 | 8.74E-05 |
| IGF2BP3 | 0.121956 | 0.529577 | 2.118483 | 2.24E-10 | 1.21E-09 |
| CTHRC1 | 10.79635 | 24.38958 | 1.175721 | 2.59E-16 | 2.92E-15 |
| AQP8 | 0.050254 | 0.113984 | 1.181527 | 0.017586 | 0.027106 |
| AL589787.2 | 1.56599 | 5.291575 | 1.756622 | 6.47E-05 | 0.00016 |
| TWIST2 | 0.238094 | 0.565092 | 1.246957 | 5.26E-07 | 1.81E-06 |
| PAH | 8.430434 | 2.380652 | -1.82425 | 1.91E-07 | 6.95E-07 |
| NCR3LG1 | 2.782618 | 1.057908 | -1.39523 | 2.97E-37 | 1.65E-34 |
| TRBV13 | 0.353016 | 0.922992 | 1.386583 | 1.21E-05 | 3.37E-05 |
| FSTL5 | 0.009208 | 0.046524 | 2.337104 | 1.43E-05 | 3.93E-05 |
| UCN2 | 0.039393 | 0.118142 | 1.584508 | 1.47E-08 | 6.19E-08 |
| LINC01605 | 0.10196 | 0.347391 | 1.768556 | 7.59E-14 | 6.26E-13 |
| TRPC2 | 0.545687 | 1.401293 | 1.360614 | 9.26E-09 | 4.00E-08 |
| MROH3P | 0.055939 | 0.12008 | 1.102065 | 0.003838 | 0.006811 |
| KCNK15-AS1 | 0.0425 | 0.097828 | 1.202784 | 1.79E-12 | 1.25E-11 |
| MIR4539 | 0.47963 | 1.375408 | 1.519866 | 0.000814 | 0.001647 |
| IGHV4-4 | 8.94992 | 30.58733 | 1.772987 | 0.00024 | 0.000536 |
| AC010997.6 | 0.093334 | 0.017535 | -2.41217 | 0.034269 | 0.049458 |
| IGKV3-11 | 41.97471 | 120.5456 | 1.521986 | 3.10E-05 | 8.08E-05 |
| PTMAP10 | 0.496195 | 1.055492 | 1.088937 | 0.00832 | 0.013774 |
| TACR3 | 0.017147 | 0.006605 | -1.37644 | 0.008925 | 0.014688 |
| FOSL1P1 | 0.112755 | 0.258141 | 1.194974 | 0.0265 | 0.039232 |
| AL589745.2 | 0.094739 | 0.038563 | -1.29673 | 7.14E-09 | 3.13E-08 |
| CBLC | 0.669453 | 1.374405 | 1.037753 | 9.38E-09 | 4.06E-08 |
| CAMKV | 0.01367 | 0.071685 | 2.390613 | 0.002949 | 0.005344 |
| DDX43P3 | 0.177938 | 0.617546 | 1.795171 | 1.11E-10 | 6.19E-10 |
| ZSCAN10 | 0.00691 | 0.027926 | 2.014836 | 0.006247 | 0.010605 |
| FEZF1-AS1 | 0.020874 | 0.046795 | 1.164661 | 1.31E-08 | 5.57E-08 |
| RNU6-46P | 0.548117 | 0.269618 | -1.02357 | 1.06E-10 | 5.93E-10 |
| AL390729.1 | 0.136837 | 0.297196 | 1.118957 | 0.000334 | 0.000728 |
| RN7SL803P | 0.13058 | 0.041808 | -1.64309 | 1.34E-05 | 3.70E-05 |
| C16orf74 | 1.77863 | 4.410218 | 1.310084 | 1.00E-17 | 1.37E-16 |
| SLPI | 31.89787 | 71.83175 | 1.171161 | 6.68E-15 | 6.27E-14 |
| KRT14 | 0.143916 | 0.535724 | 1.896262 | 6.52E-08 | 2.53E-07 |
| NKX6-3 | 0.008088 | 0.025582 | 1.661166 | 0.002077 | 0.003875 |
| IGKV1D-33 | 0.440102 | 1.295238 | 1.557308 | 0.001093 | 0.002155 |
| DDIT4L | 13.45319 | 6.149567 | -1.12939 | 1.39E-13 | 1.11E-12 |
| AL590550.1 | 0.067842 | 0.14167 | 1.062282 | 9.00E-07 | 2.99E-06 |
| AC093240.1 | 0.02445 | 0.058419 | 1.256615 | 1.95E-07 | 7.09E-07 |
| KRT18P10 | 0.076747 | 0.165523 | 1.108849 | 9.42E-11 | 5.30E-10 |
| PSME2P6 | 0.05696 | 0.132202 | 1.214735 | 0.01519 | 0.023753 |
| SERPINF1 | 12.45713 | 29.63784 | 1.250468 | 5.25E-22 | 1.28E-20 |
| AC008571.2 | 0.008617 | 0.027015 | 1.648425 | 4.68E-05 | 0.000118 |
| SHD | 0.017261 | 0.074049 | 2.100991 | 0.000664 | 0.001368 |
| COL7A1 | 0.493397 | 1.559541 | 1.6603 | 1.61E-09 | 7.73E-09 |
| MMP13 | 0.074332 | 0.878362 | 3.562758 | 3.27E-11 | 1.96E-10 |
| CDH9 | 0.246417 | 0.113217 | -1.12201 | 0.000143 | 0.000333 |
| CCNB2 | 1.363138 | 2.769017 | 1.022442 | 4.78E-19 | 7.74E-18 |
| LINC02200 | 0.028145 | 0.012536 | -1.1668 | 0.000474 | 0.001004 |
| ALDH6A1 | 11.97997 | 5.822359 | -1.04095 | 1.14E-30 | 1.33E-28 |
| HMGA2 | 0.02997 | 0.42482 | 3.825266 | 9.67E-11 | 5.43E-10 |
| ARTN | 0.300676 | 0.694268 | 1.207282 | 1.69E-10 | 9.24E-10 |
| AL445433.2 | 0.004234 | 0.015713 | 1.891936 | 0.001544 | 0.002956 |
| FRMD3 | 11.22405 | 5.611441 | -1.00015 | 3.24E-32 | 5.52E-30 |
| SLC16A9 | 30.59673 | 13.09704 | -1.22414 | 1.35E-19 | 2.35E-18 |
| AC107373.2 | 1.266854 | 3.465246 | 1.451708 | 1.58E-13 | 1.26E-12 |
| RPL13AP25 | 4.424218 | 9.789958 | 1.14588 | 3.68E-17 | 4.66E-16 |
| KRTAP1-5 | 0.02317 | 0.0493 | 1.089349 | 1.40E-06 | 4.52E-06 |
| IL17B | 0.279204 | 0.59035 | 1.080252 | 9.60E-09 | 4.14E-08 |
| P2RX5 | 0.122386 | 0.265095 | 1.11507 | 2.80E-08 | 1.14E-07 |
| ZIC1 | 0.008575 | 0.044697 | 2.381947 | 0.025572 | 0.037987 |
| IGF2BP2 | 0.772077 | 1.93481 | 1.325376 | 5.97E-12 | 3.90E-11 |
| CLIC3 | 1.222134 | 3.03732 | 1.313397 | 6.95E-25 | 2.71E-23 |
| LINC02625 | 0.423856 | 0.170677 | -1.3123 | 4.30E-19 | 7.02E-18 |
| AC008507.2 | 0.100393 | 0.042702 | -1.23327 | 8.25E-13 | 5.96E-12 |
| SIM1 | 1.60904 | 0.789877 | -1.0265 | 1.94E-05 | 5.21E-05 |
| CENPW | 1.780921 | 3.753402 | 1.075575 | 2.58E-41 | 6.08E-38 |
| DPF1 | 0.041016 | 0.105488 | 1.362808 | 5.31E-11 | 3.09E-10 |
| REG1B | 0.372524 | 1.628676 | 2.128296 | 0.000327 | 0.000713 |
| MYH6 | 0.003183 | 0.007062 | 1.149914 | 0.003805 | 0.006759 |
| AC010480.1 | 0.212947 | 0.048691 | -2.12878 | 0.000902 | 0.001809 |
| FABP4 | 13.5545 | 5.771888 | -1.23166 | 2.00E-08 | 8.29E-08 |
