# Supplementary material for: NUDT1 Could Be a Prognostic Biomarker and Correlated with Immune Infiltration in Clear Cell Renal Cell Carcinoma
Source: Appl Bionics Biomech. 2022 Dec 26;2022:3669296. doi: 10.1155/2022/3669296 (PMC9808898; doi:10.1155/2022/3669296)
Supplement: Supplementary 4 — GO enrichment analysis of DEGs. [file 3669296.f4.docx]

GO enrichment analysis of DEGs

| ONTOLOGY | ID | Description | GeneRatio | BgRatio | pvalue | p.adjust | qvalue | geneID | Count |
| --- | --- | --- | --- | --- | --- | --- | --- | --- | --- |
| BP | GO:0006959 | humoral immune response | 72/887 | 317/18800 | 1.20E-29 | 5.58E-26 | 5.10E-26 | IFNG/PDCD1/TRBC2/PRTN3/IGHV4-59/HPX/IGLC2/REG1A/C1R/IGHV5-10-1/IGHV3-33/CFHR1/IGLC3/CXCL5/ACOD1/IGHV3-35/PRSS3/TF/IGKC/F2/IGHV1-69/PI3/PRSS2/POU2AF1/WFDC10B/IGHV3-21/IGHV3-73/IGHV6-1/IGHV1-69D/IGHA2/PLA2G1B/IGHA1/IGHV3-64D/AZU1/IGHG2/IGHG1/IGHV3-49/KRT6A/CXCL13/IGHV3-72/CRP/S100A7/IGHV2-70/IGHV2-5/IGHV3-13/IGLL5/IGLC6/WFDC5/WFDC12/IGHG4/REG3G/MFAP4/PF4/C8G/KLK5/IGHV4-34/IGLC7/IGHV5-51/IGHV4-39/WFDC3/WFDC13/IGHV1-18/IGHD/IGHV3-11/IGHV3-30/IGHM/ELANE/IGKV3-20/FCER2/IGHV4-4/SLPI/REG1B | 72 |
| BP | GO:0006958 | complement activation, classical pathway | 39/887 | 108/18800 | 1.42E-24 | 1.83E-21 | 1.68E-21 | TRBC2/IGHV4-59/IGLC2/C1R/IGHV5-10-1/IGHV3-33/IGLC3/IGHV3-35/IGKC/IGHV1-69/IGHV3-21/IGHV3-73/IGHV6-1/IGHV1-69D/IGHA2/IGHA1/IGHV3-64D/IGHG2/IGHG1/IGHV3-49/IGHV3-72/CRP/IGHV2-70/IGHV2-5/IGHV3-13/IGLL5/IGLC6/IGHG4/C8G/IGHV4-34/IGLC7/IGHV5-51/IGHV4-39/IGHV1-18/IGHD/IGHV3-11/IGHV3-30/IGHM/IGHV4-4 | 39 |
| BP | GO:0006910 | phagocytosis, recognition | 38/887 | 102/18800 | 1.50E-24 | 1.83E-21 | 1.68E-21 | TRBC2/IGHV4-59/IGLC2/IGHV5-10-1/IGHV3-33/IGLC3/IGHV3-35/IGKC/PTX3/IGHV1-69/IGHV3-21/IGHV3-73/IGHV6-1/IGHV1-69D/IGHA2/IGHA1/IGHV3-64D/IGHG2/IGHG1/IGHV3-49/IGHV3-72/CRP/IGHV2-70/IGHV2-5/IGHV3-13/IGLL5/IGLC6/IGHG4/IGHV4-34/IGLC7/IGHV5-51/IGHV4-39/IGHV1-18/IGHD/IGHV3-11/IGHV3-30/IGHM/IGHV4-4 | 38 |
| BP | GO:0002455 | humoral immune response mediated by circulating immunoglobulin | 41/887 | 121/18800 | 1.58E-24 | 1.83E-21 | 1.68E-21 | TRBC2/IGHV4-59/HPX/IGLC2/C1R/IGHV5-10-1/IGHV3-33/IGLC3/IGHV3-35/IGKC/IGHV1-69/IGHV3-21/IGHV3-73/IGHV6-1/IGHV1-69D/IGHA2/IGHA1/IGHV3-64D/IGHG2/IGHG1/IGHV3-49/IGHV3-72/CRP/IGHV2-70/IGHV2-5/IGHV3-13/IGLL5/IGLC6/IGHG4/C8G/IGHV4-34/IGLC7/IGHV5-51/IGHV4-39/IGHV1-18/IGHD/IGHV3-11/IGHV3-30/IGHM/FCER2/IGHV4-4 | 41 |
| BP | GO:0006956 | complement activation | 41/887 | 131/18800 | 5.08E-23 | 4.71E-20 | 4.30E-20 | TRBC2/IGHV4-59/IGLC2/C1R/IGHV5-10-1/IGHV3-33/CFHR1/IGLC3/IGHV3-35/IGKC/IGHV1-69/IGHV3-21/IGHV3-73/IGHV6-1/IGHV1-69D/IGHA2/IGHA1/IGHV3-64D/IGHG2/IGHG1/IGHV3-49/IGHV3-72/CRP/IGHV2-70/IGHV2-5/IGHV3-13/IGLL5/IGLC6/IGHG4/MFAP4/C8G/IGHV4-34/IGLC7/IGHV5-51/IGHV4-39/IGHV1-18/IGHD/IGHV3-11/IGHV3-30/IGHM/IGHV4-4 | 41 |
| BP | GO:0002377 | immunoglobulin production | 52/887 | 218/18800 | 9.74E-23 | 7.53E-20 | 6.88E-20 | IGKV2-24/IGKV2-29/IGLV2-23/IGLV1-36/HPX/IGLV2-33/IGLV3-9/IGLV9-49/TRAV38-2DV8/IGLV4-60/VPREB3/IGKC/IGKV1-9/IGKV2-28/IGKV1-17/IGKV2D-24/IGLV10-54/IGLV1-44/IGLV3-10/IGKV1-6/IGKV3D-15/IGLV5-37/IGLV7-46/IGLV7-43/IGKV1D-13/IGKV1-13/IGKV2D-28/IGKV3-15/IGLV3-19/IGLV2-14/IGLV6-57/IGLV1-51/IGLV1-40/IGLV1-47/IGLV3-16/BATF/IGKV1D-17/AICDA/IGKV6D-21/IGLV8-61/IGKV1D-43/IGLV2-11/MZB1/IGKV3D-11/EPHB2/IGLV3-27/IGLV4-69/IGKV3D-20/FOXP3/IL21/IGKV3-20/IGKV1D-33 | 52 |
| BP | GO:0042742 | defense response to bacterium | 67/887 | 364/18800 | 3.78E-22 | 2.50E-19 | 2.29E-19 | TRBC2/IGHV4-59/MPO/EMILIN1/IGLC2/IGHV5-10-1/COCH/IGHV3-33/IGLC3/IGHV3-35/TF/IGKC/LPO/F2/IGHV1-69/SERPINE1/PI3/CHGA/WFDC10B/IGHV3-21/IGHV3-73/IGHV6-1/IGHV1-69D/IGHA2/PLA2G1B/IGHA1/HAMP/IGHV3-64D/AZU1/IGHG2/IGHG1/IGHV3-49/KRT6A/CXCL13/IGHV3-72/CRP/S100A7/IGHV2-70/IGHV2-5/IGHV3-13/IGLL5/IGLC6/WFDC5/WFDC12/IGHG4/REG3G/PGLYRP2/C10orf99/AICDA/KLK5/IGHV4-34/IGLC7/IGHV5-51/IGHV4-39/WFDC3/PYCARD/WFDC13/VGF/IGHV1-18/IGHD/IGHV3-11/IGHV3-30/IGHM/ELANE/IGKV3-20/IGHV4-4/SLPI | 67 |
| BP | GO:0050853 | B cell receptor signaling pathway | 39/887 | 131/18800 | 4.43E-21 | 2.57E-18 | 2.35E-18 | TRBC2/IGHV4-59/IGLC2/IGHV5-10-1/IGHV3-33/CD19/IGLC3/IGHV3-35/IGKC/IGHV1-69/IGHV3-21/IGHV3-73/IGHV6-1/IGHV1-69D/IGHA2/IGHA1/IGHV3-64D/IGHG2/IGHG1/IGHV3-49/BLK/IGHV3-72/IGHV2-70/IGHV2-5/IGHV3-13/IGLL5/IGLC6/IGHG4/IGHV4-34/IGLC7/IGHV5-51/IGHV4-39/CD79A/IGHV1-18/IGHD/IGHV3-11/IGHV3-30/IGHM/IGHV4-4 | 39 |
| BP | GO:0006911 | phagocytosis, engulfment | 37/887 | 128/18800 | 1.40E-19 | 7.22E-17 | 6.60E-17 | TRBC2/IGHV4-59/IGLC2/IGHV5-10-1/IGHV3-33/IGLC3/IGHV3-35/IGKC/IGHV1-69/IGHV3-21/IGHV3-73/IGHV6-1/IGHV1-69D/IGHA2/IGHA1/IGHV3-64D/IGHG2/IGHG1/IGHV3-49/XKR7/IGHV3-72/IGHV2-70/IGHV2-5/IGHV3-13/IGLL5/IGLC6/IGHG4/IGHV4-34/IGLC7/IGHV5-51/IGHV4-39/IGHV1-18/IGHD/IGHV3-11/IGHV3-30/IGHM/IGHV4-4 | 37 |
| BP | GO:0008037 | cell recognition | 49/887 | 228/18800 | 1.99E-19 | 9.25E-17 | 8.45E-17 | SPACA3/TRBC2/IGHV4-59/IGLC2/IGHV5-10-1/IGHV3-33/IGLC3/IGHV3-35/SEMA3A/IGKC/PTX3/IGHV1-69/FOXG1/IGHV3-21/IGHV3-73/IGHV6-1/IGHV1-69D/IGHA2/IGHA1/IGHV3-64D/IGHG2/IGHG1/CCL21/CNTN2/IGHV3-49/VSTM2L/IGHV3-72/CRP/IGHV2-70/IGHV2-5/IGHV3-13/NCAM2/IGLL5/IGLC6/IGHG4/CCL19/IGHV4-34/ZAN/IGLC7/IGHV5-51/IGHV4-39/EPHB2/IGHV1-18/IGHD/IGHV3-11/IGHV3-30/ZP1/IGHM/IGHV4-4 | 49 |
| BP | GO:0099024 | plasma membrane invagination | 38/887 | 137/18800 | 2.19E-19 | 9.25E-17 | 8.45E-17 | TRBC2/IGHV4-59/IGLC2/IGHV5-10-1/IGHV3-33/IGLC3/IGHV3-35/IGKC/IGHV1-69/IGHV3-21/IGHV3-73/IGHV6-1/IGHV1-69D/IGHA2/IGHA1/IGHV3-64D/IGHG2/IGHG1/IGHV3-49/XKR7/IGHV3-72/IGHV2-70/IGHV2-5/IGHV3-13/IGLL5/IGLC6/IGHG4/IGHV4-34/IGLC7/AURKB/IGHV5-51/IGHV4-39/IGHV1-18/IGHD/IGHV3-11/IGHV3-30/IGHM/IGHV4-4 | 38 |
| BP | GO:0010324 | membrane invagination | 38/887 | 144/18800 | 1.44E-18 | 5.29E-16 | 4.83E-16 | TRBC2/IGHV4-59/IGLC2/IGHV5-10-1/IGHV3-33/IGLC3/IGHV3-35/IGKC/IGHV1-69/IGHV3-21/IGHV3-73/IGHV6-1/IGHV1-69D/IGHA2/IGHA1/IGHV3-64D/IGHG2/IGHG1/IGHV3-49/XKR7/IGHV3-72/IGHV2-70/IGHV2-5/IGHV3-13/IGLL5/IGLC6/IGHG4/IGHV4-34/IGLC7/AURKB/IGHV5-51/IGHV4-39/IGHV1-18/IGHD/IGHV3-11/IGHV3-30/IGHM/IGHV4-4 | 38 |
| BP | GO:0050871 | positive regulation of B cell activation | 39/887 | 152/18800 | 1.48E-18 | 5.29E-16 | 4.83E-16 | TRBC2/IGHV4-59/IGLC2/IGHV5-10-1/IGHV3-33/IGLC3/IGHV3-35/IGKC/IGHV1-69/IGHV3-21/IGHV3-73/IGHV6-1/IGHV1-69D/IGHA2/IGHA1/IGHV3-64D/IGHG2/IGHG1/IGHV3-49/CARD11/IGHV3-72/IGHV2-70/IGHV2-5/IGHV3-13/IGLL5/IGLC6/IGHG4/IGHV4-34/IGLC7/IGHV5-51/IGHV4-39/EPHB2/IGHV1-18/IGHD/IGHV3-11/IGHV3-30/IGHM/IL21/IGHV4-4 | 39 |
| BP | GO:0002440 | production of molecular mediator of immune response | 56/887 | 312/18800 | 3.62E-18 | 1.20E-15 | 1.10E-15 | IGKV2-24/IGKV2-29/IGLV2-23/IGLV1-36/HPX/IGLV2-33/IGLV3-9/IGLV9-49/TRAV38-2DV8/IGLV4-60/VPREB3/IGKC/IGKV1-9/IGKV2-28/IGKV1-17/IGKV2D-24/IGLV10-54/IGLV1-44/IGLV3-10/IGKV1-6/SLAMF9/IGKV3D-15/IGLV5-37/IGLV7-46/IGLV7-43/IGKV1D-13/IGKV1-13/IGKV2D-28/IGKV3-15/IGLV3-19/IGLV2-14/IGLV6-57/IGLV1-51/IGLV1-40/IGLV1-47/IGLV3-16/BATF/IGKV1D-17/AICDA/KLK5/IGKV6D-21/IGLV8-61/IGKV1D-43/IGLV2-11/MZB1/IGKV3D-11/EPHB2/IGLV3-27/IGLV4-69/IGKV3D-20/FOXP3/IL21/APOA1/ELANE/IGKV3-20/IGKV1D-33 | 56 |
| BP | GO:0050864 | regulation of B cell activation | 44/887 | 200/18800 | 5.55E-18 | 1.72E-15 | 1.57E-15 | TRBC2/IGHV4-59/IGLC2/IGHV5-10-1/IGHV3-33/CD19/IGLC3/IGHV3-35/IGKC/IGHV1-69/IGHV3-21/IGHV3-73/IGHV6-1/IGHV1-69D/IGHA2/IGHA1/IGHV3-64D/TNFRSF13B/IGHG2/IGHG1/IGHV3-49/BLK/CARD11/IGHV3-72/IGHV2-70/IGHV2-5/IGHV3-13/IGLL5/IGLC6/IGHG4/IGHV4-34/IGLC7/IGHV5-51/IGHV4-39/MZB1/EPHB2/IGHV1-18/IGHD/IGHV3-11/IGHV3-30/IGHM/FOXP3/IL21/IGHV4-4 | 44 |
| BP | GO:0016064 | immunoglobulin mediated immune response | 45/887 | 216/18800 | 2.16E-17 | 6.27E-15 | 5.73E-15 | TRBC2/IGHV4-59/HPX/IGLC2/C1R/IGHV5-10-1/IGHV3-33/CD19/IGLC3/IGHV3-35/IGKC/IGHV1-69/IGHV3-21/IGHV3-73/IGHV6-1/IGHV1-69D/IGHA2/IGHA1/IGHV3-64D/IGHG2/IGHG1/IGHV3-49/IGHV3-72/CRP/IGHV2-70/IGHV2-5/IGHV3-13/IGLL5/IGLC6/IGHG4/BATF/AICDA/C8G/IGHV4-34/IGLC7/IGHV5-51/IGHV4-39/IGHV1-18/IGHD/IGHV3-11/IGHV3-30/IGHM/FOXP3/FCER2/IGHV4-4 | 45 |
| BP | GO:0019724 | B cell mediated immunity | 45/887 | 219/18800 | 3.78E-17 | 1.03E-14 | 9.43E-15 | TRBC2/IGHV4-59/HPX/IGLC2/C1R/IGHV5-10-1/IGHV3-33/CD19/IGLC3/IGHV3-35/IGKC/IGHV1-69/IGHV3-21/IGHV3-73/IGHV6-1/IGHV1-69D/IGHA2/IGHA1/IGHV3-64D/IGHG2/IGHG1/IGHV3-49/IGHV3-72/CRP/IGHV2-70/IGHV2-5/IGHV3-13/IGLL5/IGLC6/IGHG4/BATF/AICDA/C8G/IGHV4-34/IGLC7/IGHV5-51/IGHV4-39/IGHV1-18/IGHD/IGHV3-11/IGHV3-30/IGHM/FOXP3/FCER2/IGHV4-4 | 45 |
| BP | GO:0006909 | phagocytosis | 50/887 | 310/18800 | 2.00E-14 | 5.15E-12 | 4.70E-12 | SPACA3/IFNG/TRBC2/PRTN3/IGHV4-59/SRPX/IGLC2/IGHV5-10-1/IGHV3-33/IGLC3/IL2RG/IGHV3-35/IGKC/PTX3/IGHV1-69/IGHV3-21/IGHV3-73/IGHV6-1/IGHV1-69D/IGHA2/IGHA1/IGHV3-64D/AZU1/IGHG2/IGHG1/IGHV3-49/XKR7/IGHV3-72/CRP/IGHV2-70/IGHV2-5/IGHV3-13/IGLL5/IGLC6/AHSG/IGHG4/IGHV4-34/IGLC7/IGHV5-51/IGHV4-39/PYCARD/PRKCG/IGHV1-18/IGHD/IGHV3-11/IGHV3-30/IGHM/APOA1/ELANE/IGHV4-4 | 50 |
| BP | GO:0050851 | antigen receptor-mediated signaling pathway | 43/887 | 244/18800 | 5.96E-14 | 1.45E-11 | 1.33E-11 | TRBC2/IGHV4-59/IGLC2/IGHV5-10-1/IGHV3-33/CD19/IGLC3/IGHV3-35/IGKC/IGHV1-69/IGHV3-21/IGHV3-73/IGHV6-1/IGHV1-69D/IGHA2/IGHA1/IGHV3-64D/FCHO1/IGHG2/IGHG1/IGHV3-49/BLK/CARD11/IGHV3-72/IGHV2-70/IGHV2-5/IGHV3-13/IGLL5/IGLC6/IGHG4/IGHV4-34/IGLC7/IGHV5-51/IGHV4-39/KCNN4/CD79A/IGHV1-18/IGHD/IGHV3-11/IGHV3-30/IGHM/FOXP3/IGHV4-4 | 43 |
| BP | GO:0042113 | B cell activation | 51/887 | 336/18800 | 1.29E-13 | 2.99E-11 | 2.73E-11 | TRBC2/IGHV4-59/IGLC2/IGHV5-10-1/IGHV3-33/CD19/IGLC3/IGHV3-35/IL11/IGKC/IGHV1-69/ONECUT1/POU2AF1/LGALS1/IGHV3-21/IGHV3-73/IGHV6-1/IGHV1-69D/IGHA2/IGHA1/IGHV3-64D/TNFRSF13B/IGHG2/IGHG1/IGHV3-49/BLK/CARD11/IGHV3-72/IGHV2-70/IGHV2-5/IGHV3-13/IGLL5/IGLC6/IGHG4/BATF/AICDA/IGHV4-34/IGLC7/IGHV5-51/IGHV4-39/CD79A/MZB1/EPHB2/IGHV1-18/IGHD/IGHV3-11/IGHV3-30/IGHM/FOXP3/IL21/IGHV4-4 | 51 |
| BP | GO:0019730 | antimicrobial humoral response | 29/887 | 122/18800 | 3.22E-13 | 7.11E-11 | 6.50E-11 | PRTN3/REG1A/CXCL5/ACOD1/PRSS3/TF/F2/PI3/PRSS2/WFDC10B/IGHA2/PLA2G1B/IGHA1/AZU1/KRT6A/CXCL13/S100A7/WFDC5/WFDC12/REG3G/PF4/KLK5/WFDC3/WFDC13/IGHM/ELANE/IGKV3-20/SLPI/REG1B | 29 |
| BP | GO:0002443 | leukocyte mediated immunity | 59/887 | 457/18800 | 1.96E-12 | 4.14E-10 | 3.78E-10 | TRBC2/IGHV4-59/SLC22A13/HPX/IGLC2/LAG3/C1R/IGHV5-10-1/IGHV3-33/CD19/IGLC3/IGHV3-35/IGKC/F2/IGHV1-69/CHGA/IGHV3-21/IGHV3-73/IGHV6-1/IGHV1-69D/IGHA2/PLA2G1B/IGHA1/IGHV3-64D/AZU1/IL20RB/IGHG2/IGHG1/SLAMF9/IGHV3-49/BLK/IGHV3-72/CRP/IGHV2-70/IGHV2-5/IGHV3-13/IGLL5/IGLC6/CD177/KLRF2/IGHG4/BATF/CPLX2/AICDA/C8G/IGHV4-34/IGLC7/IGHV5-51/IGHV4-39/IGHV1-18/IGHD/IGHV3-11/IGHV3-30/IGHM/FOXP3/IL21/ELANE/FCER2/IGHV4-4 | 59 |
| BP | GO:0002696 | positive regulation of leukocyte activation | 56/887 | 421/18800 | 2.16E-12 | 4.37E-10 | 3.99E-10 | SPACA3/IFNG/MDK/TRBC2/IGHV4-59/IGLC2/IGHV5-10-1/IGHV3-33/IGLC3/IGHV3-35/IGKC/IL1RL1/IGHV1-69/LGALS1/IGHV3-21/IGHV3-73/IGHV6-1/IGHV1-69D/IGHA2/IGHA1/HAMP/IGHV3-64D/FCHO1/PCK1/IGHG2/IGHG1/CCL21/IGHV3-49/CARD11/TAFA3/IGHV3-72/IGHV2-70/IGHV2-5/IGHV3-13/IGLL5/IGLC6/FGF10/CD177/IGHG4/CCL19/IGHV4-34/IGLC7/IGHV5-51/IGHV4-39/PYCARD/EPO/EPHB2/IGHV1-18/IGHD/IGHV3-11/IGHV3-30/CCL5/IGHM/FOXP3/IL21/IGHV4-4 | 56 |
| BP | GO:0002460 | adaptive immune response based on somatic recombination of immune receptors built from immunoglobulin superfamily domains | 51/887 | 370/18800 | 5.57E-12 | 1.08E-09 | 9.85E-10 | TRBC2/IGHV4-59/SLC22A13/HPX/IGLC2/C1R/IGHV5-10-1/IGHV3-33/CD19/IGLC3/IGHV3-35/IGKC/IL1RL1/IGHV1-69/IGHV3-21/IGHV3-73/IGHV6-1/IGHV1-69D/IGHA2/IGHA1/IGHV3-64D/IL20RB/IGHG2/IGHG1/IGHV3-49/CXCL13/IGHV3-72/CRP/IGHV2-70/IGHV2-5/IGHV3-13/IGLL5/IGLC6/IGHG4/BATF/AICDA/C8G/CCL19/IGHV4-34/IGLC7/IGHV5-51/IGHV4-39/EPHB2/IGHV1-18/IGHD/IGHV3-11/IGHV3-30/IGHM/FOXP3/FCER2/IGHV4-4 | 51 |
| BP | GO:0051251 | positive regulation of lymphocyte activation | 51/887 | 371/18800 | 6.18E-12 | 1.15E-09 | 1.05E-09 | IFNG/MDK/TRBC2/IGHV4-59/IGLC2/IGHV5-10-1/IGHV3-33/IGLC3/IGHV3-35/IGKC/IGHV1-69/LGALS1/IGHV3-21/IGHV3-73/IGHV6-1/IGHV1-69D/IGHA2/IGHA1/IGHV3-64D/FCHO1/PCK1/IGHG2/IGHG1/CCL21/IGHV3-49/CARD11/IGHV3-72/IGHV2-70/IGHV2-5/IGHV3-13/IGLL5/IGLC6/FGF10/IGHG4/CCL19/IGHV4-34/IGLC7/IGHV5-51/IGHV4-39/PYCARD/EPO/EPHB2/IGHV1-18/IGHD/IGHV3-11/IGHV3-30/CCL5/IGHM/FOXP3/IL21/IGHV4-4 | 51 |
| BP | GO:0050867 | positive regulation of cell activation | 56/887 | 436/18800 | 8.97E-12 | 1.60E-09 | 1.46E-09 | SPACA3/IFNG/MDK/TRBC2/IGHV4-59/IGLC2/IGHV5-10-1/IGHV3-33/IGLC3/IGHV3-35/IGKC/IL1RL1/IGHV1-69/LGALS1/IGHV3-21/IGHV3-73/IGHV6-1/IGHV1-69D/IGHA2/IGHA1/HAMP/IGHV3-64D/FCHO1/PCK1/IGHG2/IGHG1/CCL21/IGHV3-49/CARD11/TAFA3/IGHV3-72/IGHV2-70/IGHV2-5/IGHV3-13/IGLL5/IGLC6/FGF10/CD177/IGHG4/CCL19/IGHV4-34/IGLC7/IGHV5-51/IGHV4-39/PYCARD/EPO/EPHB2/IGHV1-18/IGHD/IGHV3-11/IGHV3-30/CCL5/IGHM/FOXP3/IL21/IGHV4-4 | 56 |
| BP | GO:0002449 | lymphocyte mediated immunity | 50/887 | 365/18800 | 1.14E-11 | 1.96E-09 | 1.79E-09 | TRBC2/IGHV4-59/SLC22A13/HPX/IGLC2/LAG3/C1R/IGHV5-10-1/IGHV3-33/CD19/IGLC3/IGHV3-35/IGKC/IGHV1-69/IGHV3-21/IGHV3-73/IGHV6-1/IGHV1-69D/IGHA2/IGHA1/IGHV3-64D/IL20RB/IGHG2/IGHG1/IGHV3-49/IGHV3-72/CRP/IGHV2-70/IGHV2-5/IGHV3-13/IGLL5/IGLC6/KLRF2/IGHG4/BATF/AICDA/C8G/IGHV4-34/IGLC7/IGHV5-51/IGHV4-39/IGHV1-18/IGHD/IGHV3-11/IGHV3-30/IGHM/FOXP3/IL21/FCER2/IGHV4-4 | 50 |
| BP | GO:0002253 | activation of immune response | 51/887 | 386/18800 | 2.74E-11 | 4.55E-09 | 4.15E-09 | TRBC2/IGHV4-59/IGLC2/C1R/IGHV5-10-1/IGHV3-33/CFHR1/CD19/IGLC3/IGHV3-35/IGKC/IGHV1-69/IGHV3-21/IGHV3-73/IGHV6-1/IGHV1-69D/IGHA2/IGHA1/IGHV3-64D/FCHO1/IGHG2/IGHG1/IGHV3-49/BLK/CARD11/AIM2/IGHV3-72/CRP/IGHV2-70/IGHV2-5/IGHV3-13/IGLL5/IGLC6/HMSD/IGHG4/MFAP4/C8G/IGHV4-34/IGLC7/IGHV5-51/IGHV4-39/KCNN4/CD79A/PYCARD/IGHV1-18/IGHD/IGHV3-11/IGHV3-30/IGHM/FOXP3/IGHV4-4 | 51 |
| BP | GO:0002429 | immune response-activating cell surface receptor signaling pathway | 43/887 | 300/18800 | 7.42E-11 | 1.15E-08 | 1.05E-08 | TRBC2/IGHV4-59/IGLC2/IGHV5-10-1/IGHV3-33/CD19/IGLC3/IGHV3-35/IGKC/IGHV1-69/IGHV3-21/IGHV3-73/IGHV6-1/IGHV1-69D/IGHA2/IGHA1/IGHV3-64D/FCHO1/IGHG2/IGHG1/IGHV3-49/BLK/CARD11/IGHV3-72/IGHV2-70/IGHV2-5/IGHV3-13/IGLL5/IGLC6/IGHG4/IGHV4-34/IGLC7/IGHV5-51/IGHV4-39/KCNN4/CD79A/IGHV1-18/IGHD/IGHV3-11/IGHV3-30/IGHM/FOXP3/IGHV4-4 | 43 |
| BP | GO:0002757 | immune response-activating signal transduction | 43/887 | 300/18800 | 7.42E-11 | 1.15E-08 | 1.05E-08 | TRBC2/IGHV4-59/IGLC2/IGHV5-10-1/IGHV3-33/CD19/IGLC3/IGHV3-35/IGKC/IGHV1-69/IGHV3-21/IGHV3-73/IGHV6-1/IGHV1-69D/IGHA2/IGHA1/IGHV3-64D/FCHO1/IGHG2/IGHG1/IGHV3-49/BLK/CARD11/IGHV3-72/IGHV2-70/IGHV2-5/IGHV3-13/IGLL5/IGLC6/IGHG4/IGHV4-34/IGLC7/IGHV5-51/IGHV4-39/KCNN4/CD79A/IGHV1-18/IGHD/IGHV3-11/IGHV3-30/IGHM/FOXP3/IGHV4-4 | 43 |
| BP | GO:0030198 | extracellular matrix organization | 42/887 | 307/18800 | 5.39E-10 | 8.06E-08 | 7.37E-08 | HAS1/MELTF/SFRP2/ADAMTS14/APLP1/EMILIN1/COL22A1/COLGALT2/IBSP/PTX3/TLL2/PRSS2/GREM1/LOXL1/TGFBI/PDGFRA/COL10A1/COL19A1/DMP1/COL5A1/LUM/TMPRSS6/MMP9/MFAP4/MMP23B/COL1A1/KLK5/COL11A1/EGFL6/SERPINB5/GFAP/COL1A2/COL8A2/CPB2/HAS2/COMP/PDPN/WDR72/ELANE/MMP7/MMP12/MMP13 | 42 |
| BP | GO:0043062 | extracellular structure organization | 42/887 | 308/18800 | 5.96E-10 | 8.65E-08 | 7.90E-08 | HAS1/MELTF/SFRP2/ADAMTS14/APLP1/EMILIN1/COL22A1/COLGALT2/IBSP/PTX3/TLL2/PRSS2/GREM1/LOXL1/TGFBI/PDGFRA/COL10A1/COL19A1/DMP1/COL5A1/LUM/TMPRSS6/MMP9/MFAP4/MMP23B/COL1A1/KLK5/COL11A1/EGFL6/SERPINB5/GFAP/COL1A2/COL8A2/CPB2/HAS2/COMP/PDPN/WDR72/ELANE/MMP7/MMP12/MMP13 | 42 |
| BP | GO:0045229 | external encapsulating structure organization | 42/887 | 310/18800 | 7.29E-10 | 1.03E-07 | 9.37E-08 | HAS1/MELTF/SFRP2/ADAMTS14/APLP1/EMILIN1/COL22A1/COLGALT2/IBSP/PTX3/TLL2/PRSS2/GREM1/LOXL1/TGFBI/PDGFRA/COL10A1/COL19A1/DMP1/COL5A1/LUM/TMPRSS6/MMP9/MFAP4/MMP23B/COL1A1/KLK5/COL11A1/EGFL6/SERPINB5/GFAP/COL1A2/COL8A2/CPB2/HAS2/COMP/PDPN/WDR72/ELANE/MMP7/MMP12/MMP13 | 42 |
| BP | GO:0002768 | immune response-regulating cell surface receptor signaling pathway | 43/887 | 328/18800 | 1.29E-09 | 1.76E-07 | 1.61E-07 | TRBC2/IGHV4-59/IGLC2/IGHV5-10-1/IGHV3-33/CD19/IGLC3/IGHV3-35/IGKC/IGHV1-69/IGHV3-21/IGHV3-73/IGHV6-1/IGHV1-69D/IGHA2/IGHA1/IGHV3-64D/FCHO1/IGHG2/IGHG1/IGHV3-49/BLK/CARD11/IGHV3-72/IGHV2-70/IGHV2-5/IGHV3-13/IGLL5/IGLC6/IGHG4/IGHV4-34/IGLC7/IGHV5-51/IGHV4-39/KCNN4/CD79A/IGHV1-18/IGHD/IGHV3-11/IGHV3-30/IGHM/FOXP3/IGHV4-4 | 43 |
| BP | GO:0045109 | intermediate filament organization | 17/887 | 68/18800 | 1.13E-08 | 1.50E-06 | 1.37E-06 | KRT39/KRT13/INA/KRT5/KRT4/KRT79/KRT19/KRT15/KRT6A/KRT25/KRT16/DES/KRT78/TCHH/KRT6B/GFAP/KRT14 | 17 |
| BP | GO:0035270 | endocrine system development | 23/887 | 132/18800 | 5.30E-08 | 6.83E-06 | 6.24E-06 | MDK/CGA/OTP/SOX2/ONECUT1/STRA6/BMP5/MNX1/FOXE1/FOXA2/PDGFRA/PCSK1/ONECUT2/NKX6-1/WNT4/FGF10/NKX2-5/PITX1/PITX2/FGF8/POU3F2/APOA1/SIX3 | 23 |
| BP | GO:0019731 | antibacterial humoral response | 15/887 | 60/18800 | 8.25E-08 | 1.03E-05 | 9.45E-06 | TF/PI3/WFDC10B/IGHA2/PLA2G1B/IGHA1/WFDC5/WFDC12/KLK5/WFDC3/WFDC13/IGHM/ELANE/IGKV3-20/SLPI | 15 |
| BP | GO:0045165 | cell fate commitment | 33/887 | 266/18800 | 3.94E-07 | 4.82E-05 | 4.40E-05 | SFRP2/WNT7B/PRRX1/WNT2/DMRTA2/SOX2/ONECUT1/FOXG1/MNX1/DMRT3/MYL2/ISL2/SOSTDC1/SIX2/FOXA1/FOXA2/WNT10A/ONECUT2/WNT4/SOX6/FOXN1/FGF10/BATF/NKX2-5/TBX18/PITX1/FGF8/POU3F2/SOX1/PDPN/FOXP3/SPDEF/NKX6-3 | 33 |
| BP | GO:0045104 | intermediate filament cytoskeleton organization | 17/887 | 88/18800 | 6.36E-07 | 7.57E-05 | 6.92E-05 | KRT39/KRT13/INA/KRT5/KRT4/KRT79/KRT19/KRT15/KRT6A/KRT25/KRT16/DES/KRT78/TCHH/KRT6B/GFAP/KRT14 | 17 |
| BP | GO:0045103 | intermediate filament-based process | 17/887 | 89/18800 | 7.53E-07 | 8.73E-05 | 7.98E-05 | KRT39/KRT13/INA/KRT5/KRT4/KRT79/KRT19/KRT15/KRT6A/KRT25/KRT16/DES/KRT78/TCHH/KRT6B/GFAP/KRT14 | 17 |
| BP | GO:0032963 | collagen metabolic process | 18/887 | 101/18800 | 1.04E-06 | 0.000118 | 0.000108 | PRTN3/ADAMTS14/EMILIN1/F2/PRSS2/MRC2/WNT4/COL5A1/TMPRSS6/MMP9/MFAP4/MMP23B/COL1A1/COL1A2/P3H3/MMP7/MMP12/MMP13 | 18 |
| BP | GO:0021536 | diencephalon development | 15/887 | 75/18800 | 1.82E-06 | 0.000201 | 0.000184 | OTP/FOXB1/SEMA3A/SOX2/PCSK1/WNT4/GBX2/FGF10/SRD5A2/OTX1/PITX1/PITX2/FGF8/POU3F2/SIX3 | 15 |
| BP | GO:0010466 | negative regulation of peptidase activity | 31/887 | 262/18800 | 2.44E-06 | 0.000263 | 0.00024 | SFRP2/RARRES1/NLRP7/ECM1/SERPINE1/PI3/SPINK13/CST6/WFDC10B/PTTG1/ANXA8/ITIH3/ITIH1/TFPI2/WFDC5/PI15/HMSD/WFDC12/TIMP1/AHSG/MMP9/SPINK2/BIRC5/WFDC3/WFDC13/SERPINB5/VIL1/COL6A3/SLPI/SERPINF1/COL7A1 | 31 |
| BP | GO:0042475 | odontogenesis of dentin-containing tooth | 16/887 | 90/18800 | 4.25E-06 | 0.000448 | 0.00041 | FST/SERPINE1/DMRT3/MSX2/SOSTDC1/PDGFRA/WNT10A/RSPO2/DMP1/FGF10/KLK5/ODAPH/ENAM/FGF8/WDR72/LHX8 | 16 |
| BP | GO:0002764 | immune response-regulating signaling pathway | 46/887 | 482/18800 | 4.74E-06 | 0.000489 | 0.000447 | TRBC2/IGHV4-59/IGLC2/IGHV5-10-1/IGHV3-33/CD19/IGLC3/ACOD1/IGHV3-35/IGKC/IGHV1-69/IGHV3-21/IGHV3-73/IGHV6-1/IGHV1-69D/IGHA2/IGHA1/IGHV3-64D/FCHO1/IL20RB/IGHG2/IGHG1/IGHV3-49/BLK/CARD11/IGHV3-72/IGHV2-70/IGHV2-5/IGHV3-13/IGLL5/IGLC6/IGHG4/REG3G/IGHV4-34/IGLC7/IGHV5-51/IGHV4-39/KCNN4/CD79A/IGHV1-18/IGHD/IGHV3-11/IGHV3-30/IGHM/FOXP3/IGHV4-4 | 46 |
| BP | GO:0030199 | collagen fibril organization | 13/887 | 62/18800 | 5.09E-06 | 0.000514 | 0.000469 | SFRP2/ADAMTS14/EMILIN1/COLGALT2/TLL2/GREM1/LOXL1/COL5A1/LUM/COL1A1/COL11A1/COL1A2/COMP | 13 |
| BP | GO:0051216 | cartilage development | 25/887 | 195/18800 | 5.51E-06 | 0.000544 | 0.000497 | MDK/SFRP2/WNT7B/EPYC/PRRX1/GDF5/ECM1/RFLNA/BMP5/GREM1/CHRDL2/TGFBI/MSX2/SIX2/SHOX2/RSPO2/SOX6/TIMP1/NKX3-2/COL1A1/COL11A1/PITX1/COMP/MMP13/HMGA2 | 25 |
| BP | GO:0048732 | gland development | 42/887 | 431/18800 | 7.33E-06 | 0.000709 | 0.000648 | IRF6/PRLR/MDK/WNT7B/CGA/CCKBR/OTP/WNT2/FOXB1/CELA1/SEMA3A/SOX2/ONECUT1/STRA6/HAMP/CAV3/PCK1/FOXE1/MSX2/SOSTDC1/FOXA1/PDGFRA/CYP19A1/WNT10A/PCSK1/ONECUT2/CCL11/WNT4/FOXN1/FGF10/NKX2-5/PITX1/PITX2/SERPINB5/FGF8/POU3F2/CPB2/APOA1/SIX3/E2F7/SERPINF1/CCNB2 | 42 |
| BP | GO:0010951 | negative regulation of endopeptidase activity | 29/887 | 251/18800 | 8.15E-06 | 0.000772 | 0.000705 | SFRP2/RARRES1/NLRP7/SERPINE1/PI3/SPINK13/CST6/WFDC10B/PTTG1/ANXA8/ITIH3/ITIH1/TFPI2/WFDC5/HMSD/WFDC12/TIMP1/AHSG/MMP9/SPINK2/BIRC5/WFDC3/WFDC13/SERPINB5/VIL1/COL6A3/SLPI/SERPINF1/COL7A1 | 29 |
| BP | GO:0042476 | odontogenesis | 19/887 | 130/18800 | 1.11E-05 | 0.001033 | 0.000944 | FST/SERPINE1/DMRT3/MSX2/SOSTDC1/PDGFRA/WNT10A/RSPO2/DMP1/FGF10/COL1A1/KLK5/ODAPH/PITX2/ENAM/COL1A2/FGF8/WDR72/LHX8 | 19 |
| BP | GO:0008544 | epidermis development | 36/887 | 355/18800 | 1.38E-05 | 0.001253 | 0.001145 | IRF6/FST/LHX2/CRABP2/ETV4/CST6/KRT5/KRT4/IVL/KRT79/CNFN/SULT2B1/FOXE1/MSX2/SOSTDC1/KRT15/KRT6A/WNT10A/EREG/ZBED2/S100A7/KRT25/KRT16/FOXN1/KRT78/TCHH/KRT6B/FGF10/REG3G/KLK5/PITX2/POU3F2/SFRP4/TGM5/KRT14/COL7A1 | 36 |
| BP | GO:0021983 | pituitary gland development | 10/887 | 41/18800 | 1.53E-05 | 0.001368 | 0.00125 | OTP/SOX2/PCSK1/WNT4/FGF10/PITX1/PITX2/FGF8/POU3F2/SIX3 | 10 |
| BP | GO:0061448 | connective tissue development | 29/887 | 260/18800 | 1.60E-05 | 0.001405 | 0.001284 | MDK/SFRP2/WNT7B/EPYC/PRRX1/GDF5/PPARGC1A/ECM1/RFLNA/CASR/BMP5/GREM1/CHRDL2/TGFBI/MSX2/SIX2/FOXA1/SHOX2/RSPO2/COL5A1/SOX6/TIMP1/NKX3-2/COL1A1/COL11A1/PITX1/COMP/MMP13/HMGA2 | 29 |
| BP | GO:0030574 | collagen catabolic process | 10/887 | 42/18800 | 1.93E-05 | 0.001657 | 0.001513 | PRTN3/ADAMTS14/PRSS2/MRC2/TMPRSS6/MMP9/MMP23B/MMP7/MMP12/MMP13 | 10 |
| BP | GO:0010817 | regulation of hormone levels | 45/887 | 496/18800 | 2.16E-05 | 0.001826 | 0.001668 | IFNG/CRABP1/ILDR2/CGA/GALR1/CRABP2/IL11/SLC5A5/CASR/ADH1C/CHGA/BMP5/AKR1D1/EFNA5/FOXE1/FOXA1/FOXA2/PDGFRA/AWAT2/ADCYAP1/CYP19A1/PCSK1/BLK/GCK/C1QTNF1/SCG5/NKX6-1/GNB3/EDN3/WNT4/CYP3A4/HSD17B6/SRD5A2/RDH16/AGTR1/KISS1/DIO2/SDR16C5/PTPRN/GRP/CYP17A1/VGF/ADH4/CCL5/CHST8 | 45 |
| BP | GO:0003228 | atrial cardiac muscle tissue development | 7/887 | 20/18800 | 2.29E-05 | 0.001901 | 0.001737 | GJB6/WNT2/CACNA1G/SHOX2/NKX2-5/TBX18/MYH6 | 7 |
| BP | GO:0045861 | negative regulation of proteolysis | 35/887 | 350/18800 | 2.43E-05 | 0.001978 | 0.001807 | IL1R2/SFRP2/RARRES1/NLRP7/ECM1/F2/SERPINE1/PI3/SPINK13/CST6/WFDC10B/PTTG1/ANXA8/ITIH3/ITIH1/TFPI2/WFDC5/PI15/HMSD/WFDC12/TIMP1/AHSG/MMP9/SPINK2/BIRC5/WFDC3/WFDC13/SERPINB5/PRKCG/CPB2/VIL1/COL6A3/SLPI/SERPINF1/COL7A1 | 35 |
| BP | GO:0043588 | skin development | 31/887 | 296/18800 | 2.89E-05 | 0.002311 | 0.002111 | IRF6/FST/LHX2/LTB/ETV4/KRT5/KRT4/IVL/KRT79/CNFN/FOXE1/MSX2/SOSTDC1/KRT6A/WNT10A/EREG/ZBED2/S100A7/KRT25/KRT16/COL5A1/FOXN1/KRT78/TCHH/KRT6B/FGF10/REG3G/COL1A1/KLK5/COL1A2/COMP | 31 |
| BP | GO:0051346 | negative regulation of hydrolase activity | 36/887 | 371/18800 | 3.55E-05 | 0.002794 | 0.002553 | SFRP2/RARRES1/NLRP7/ECM1/PTX3/SERPINE1/PI3/SPINK13/CST6/WFDC10B/PTTG1/ANXA8/ITIH3/ITIH1/PLXNB3/TFPI2/WFDC5/PI15/HMSD/APOC3/APOC1/WFDC12/TIMP1/AHSG/MMP9/SPINK2/BIRC5/WFDC3/WFDC13/SERPINB5/VIL1/APOA1/COL6A3/SLPI/SERPINF1/COL7A1 | 36 |
| BP | GO:0071542 | dopaminergic neuron differentiation | 9/887 | 37/18800 | 4.19E-05 | 0.003242 | 0.002962 | SFRP2/WNT2/DMRTA2/FOXA1/FOXA2/RSPO2/PITX3/EN1/FGF8 | 9 |
| BP | GO:0034368 | protein-lipid complex remodeling | 8/887 | 30/18800 | 5.51E-05 | 0.004056 | 0.003705 | MPO/PLTP/APOC3/APOC1/APOC2/AGTR1/MTTP/APOA1 | 8 |
| BP | GO:0034369 | plasma lipoprotein particle remodeling | 8/887 | 30/18800 | 5.51E-05 | 0.004056 | 0.003705 | MPO/PLTP/APOC3/APOC1/APOC2/AGTR1/MTTP/APOA1 | 8 |
| BP | GO:0051873 | killing by host of symbiont cells | 8/887 | 30/18800 | 5.51E-05 | 0.004056 | 0.003705 | CFHR1/F2/APOL1/AZU1/KRT6A/REG3G/PF4/ELANE | 8 |
| BP | GO:0006874 | cellular calcium ion homeostasis | 41/887 | 456/18800 | 6.09E-05 | 0.004414 | 0.004033 | GRIN1/TRPV3/GALR1/CCKBR/HCRT/CD19/JSRP1/GRIN2D/F2/CASR/PLA2G1B/RYR2/CAV3/CACNA1B/CCL21/PDGFRA/CXCL13/ADCYAP1/CCL7/C1QTNF1/CCL11/SLC8A2/CEMIP/EDN3/CHRNA9/ADRA1D/GRIN2B/AVPR1B/AGTR1/KISS1/TRPM8/TNNI3/CXCR3/PKHD1/CCL19/ATP13A4/PTGER1/EPO/CCL5/ELANE/P2RX5 | 41 |
| BP | GO:0090596 | sensory organ morphogenesis | 28/887 | 266/18800 | 6.33E-05 | 0.00452 | 0.004129 | PRRX1/GJB6/WNT2/MFSD2A/FOXG1/STRA6/RORB/MFAP2/SIX2/FZD2/COL5A1/GBX2/CHRNA9/FGF10/TBX18/OTX1/NKX3-2/COL11A1/PITX3/PITX2/TENM3/FGF8/COL8A2/SOX1/EPHB2/SIX3/CTHRC1/ZIC1 | 28 |
| BP | GO:0007586 | digestion | 18/887 | 137/18800 | 7.97E-05 | 0.005602 | 0.005118 | MDK/CCKBR/PPARGC1A/PRSS3/PRSS2/AKR1D1/HAMP/TFF2/CHRM3/NPR3/TFF1/PNLIPRP2/FGF10/KCNN4/SLC5A1/VIL1/APOA1/UCN2 | 18 |
| BP | GO:0072503 | cellular divalent inorganic cation homeostasis | 43/887 | 494/18800 | 8.55E-05 | 0.005925 | 0.005412 | GRIN1/TRPV3/GALR1/SLC30A3/CCKBR/HCRT/CD19/JSRP1/GRIN2D/F2/CASR/PLA2G1B/RYR2/CAV3/CACNA1B/CCL21/PDGFRA/CXCL13/ADCYAP1/CCL7/C1QTNF1/CCL11/SLC8A2/CEMIP/EDN3/CHRNA9/ADRA1D/GRIN2B/AVPR1B/AGTR1/KISS1/TRPM8/TNNI3/CXCR3/PKHD1/CCL19/ATP13A4/PTGER1/MT2A/EPO/CCL5/ELANE/P2RX5 | 43 |
| BP | GO:0034367 | protein-containing complex remodeling | 8/887 | 32/18800 | 9.10E-05 | 0.006185 | 0.00565 | MPO/PLTP/APOC3/APOC1/APOC2/AGTR1/MTTP/APOA1 | 8 |
| BP | GO:0042445 | hormone metabolic process | 25/887 | 230/18800 | 9.20E-05 | 0.006185 | 0.00565 | CRABP1/CGA/CRABP2/SLC5A5/ADH1C/BMP5/AKR1D1/FOXE1/FOXA1/PDGFRA/AWAT2/CYP19A1/PCSK1/SCG5/GNB3/WNT4/CYP3A4/HSD17B6/SRD5A2/RDH16/DIO2/SDR16C5/CYP17A1/ADH4/CHST8 | 25 |
| BP | GO:0055074 | calcium ion homeostasis | 41/887 | 468/18800 | 0.000108 | 0.007155 | 0.006536 | GRIN1/TRPV3/GALR1/CCKBR/HCRT/CD19/JSRP1/GRIN2D/F2/CASR/PLA2G1B/RYR2/CAV3/CACNA1B/CCL21/PDGFRA/CXCL13/ADCYAP1/CCL7/C1QTNF1/CCL11/SLC8A2/CEMIP/EDN3/CHRNA9/ADRA1D/GRIN2B/AVPR1B/AGTR1/KISS1/TRPM8/TNNI3/CXCR3/PKHD1/CCL19/ATP13A4/PTGER1/EPO/CCL5/ELANE/P2RX5 | 41 |
| BP | GO:0007411 | axon guidance | 25/887 | 234/18800 | 0.000121 | 0.007924 | 0.007239 | LHX2/EPHA8/DPYSL5/CDK5R2/SEMA3A/EFNA2/SEMA3D/FOXG1/EPHA10/UNC5A/EFNA5/ISL2/CNTN2/VSTM2L/TUBB3/GFRA3/LRTM2/PLXNB3/EDN3/GBX2/L1CAM/EVX1/EPHB2/LGI1/ARTN | 25 |
| BP | GO:0097485 | neuron projection guidance | 25/887 | 235/18800 | 0.00013 | 0.008363 | 0.00764 | LHX2/EPHA8/DPYSL5/CDK5R2/SEMA3A/EFNA2/SEMA3D/FOXG1/EPHA10/UNC5A/EFNA5/ISL2/CNTN2/VSTM2L/TUBB3/GFRA3/LRTM2/PLXNB3/EDN3/GBX2/L1CAM/EVX1/EPHB2/LGI1/ARTN | 25 |
| BP | GO:0022617 | extracellular matrix disassembly | 11/887 | 62/18800 | 0.000135 | 0.008607 | 0.007863 | MELTF/PRSS2/TMPRSS6/MMP9/KLK5/PDPN/WDR72/ELANE/MMP7/MMP12/MMP13 | 11 |
| BP | GO:0050900 | leukocyte migration | 35/887 | 384/18800 | 0.000158 | 0.009932 | 0.009073 | MDK/PRTN3/EMILIN1/CXCL5/ECM1/SCG2/SERPINE1/CCL25/CHGA/BMP5/GREM1/PLA2G1B/AZU1/CCL21/CXCL13/CYP19A1/CCL7/CCL11/ADD2/S100A7/EDN3/MMP9/CD177/C10orf99/PF4/CHST4/CXCR3/CCL19/CNR2/PYCARD/CCL26/CCL5/ELANE/TNFRSF18/ARTN | 35 |
| BP | GO:0007204 | positive regulation of cytosolic calcium ion concentration | 31/887 | 325/18800 | 0.000165 | 0.010233 | 0.009349 | GRIN1/TRPV3/GALR1/CCKBR/HCRT/CD19/JSRP1/GRIN2D/F2/PLA2G1B/RYR2/CAV3/CACNA1B/CCL21/PDGFRA/CXCL13/ADCYAP1/C1QTNF1/SLC8A2/CEMIP/CHRNA9/ADRA1D/GRIN2B/AVPR1B/AGTR1/KISS1/CXCR3/CCL19/PTGER1/EPO/P2RX5 | 31 |
| BP | GO:0048245 | eosinophil chemotaxis | 6/887 | 19/18800 | 0.000173 | 0.010579 | 0.009665 | SCG2/CCL21/CCL7/CCL11/CCL26/CCL5 | 6 |
| BP | GO:0002062 | chondrocyte differentiation | 15/887 | 110/18800 | 0.000202 | 0.01219 | 0.011136 | MDK/SFRP2/GDF5/ECM1/RFLNA/GREM1/TGFBI/MSX2/SIX2/SHOX2/SOX6/NKX3-2/COL11A1/COMP/HMGA2 | 15 |
| BP | GO:0071827 | plasma lipoprotein particle organization | 9/887 | 45/18800 | 0.000213 | 0.012661 | 0.011567 | MPO/MFSD2A/PLTP/APOC3/APOC1/APOC2/AGTR1/MTTP/APOA1 | 9 |
| BP | GO:0042471 | ear morphogenesis | 16/887 | 123/18800 | 0.000219 | 0.012884 | 0.01177 | PRRX1/GJB6/FOXG1/SIX2/FZD2/GBX2/CHRNA9/FGF10/TBX18/OTX1/NKX3-2/COL11A1/FGF8/EPHB2/CTHRC1/ZIC1 | 16 |
| BP | GO:0060485 | mesenchyme development | 29/887 | 301/18800 | 0.000224 | 0.012988 | 0.011865 | MDK/SFRP2/DLL3/WNT2/ALX1/SEMA3A/SEMA3D/DAND5/BMP5/GREM1/MSX2/SIX2/FOXA1/FOXA2/AMH/WNT10A/IL17RD/BASP1/EDN3/WNT4/GBX2/FGF10/NKX2-5/COL1A1/PITX2/FGF8/HAS2/PDPN/HMGA2 | 29 |
| BP | GO:0007568 | aging | 19/887 | 163/18800 | 0.000254 | 0.014552 | 0.013294 | IGFBP1/MPO/GJB6/FOXG1/HAMP/PCK1/NUDT1/AMH/KRT25/KRT16/TIMP1/AVPR1B/PITX3/AURKB/EPO/COMP/TACR3/KRT14/SERPINF1 | 19 |
| BP | GO:0048736 | appendage development | 20/887 | 177/18800 | 0.000269 | 0.015014 | 0.013716 | IRF6/SFRP2/PRRX1/CRABP2/GDF5/KREMEN2/RSPO4/GREM1/FREM2/MSX2/SHOX2/HOXD12/RSPO2/FOXN1/FGF10/PITX1/PITX2/EN1/FGF8/COMP | 20 |
| BP | GO:0060173 | limb development | 20/887 | 177/18800 | 0.000269 | 0.015014 | 0.013716 | IRF6/SFRP2/PRRX1/CRABP2/GDF5/KREMEN2/RSPO4/GREM1/FREM2/MSX2/SHOX2/HOXD12/RSPO2/FOXN1/FGF10/PITX1/PITX2/EN1/FGF8/COMP | 20 |
| BP | GO:0002526 | acute inflammatory response | 15/887 | 113/18800 | 0.000273 | 0.015087 | 0.013783 | IL31RA/F3/F2/HAMP/IL20RB/ORM1/IGHG1/ORM2/CRP/AHSG/REG3G/HPR/EPO/PLA2G2D/ELANE | 15 |
| BP | GO:0003206 | cardiac chamber morphogenesis | 16/887 | 126/18800 | 0.00029 | 0.015834 | 0.014466 | SFRP2/WNT2/BMP5/RYR2/CAV3/MYL2/MSX2/SHOX2/MYH7/FZD2/NKX2-5/TNNI3/COL11A1/FGF8/LRP2/MYH6 | 16 |
| BP | GO:0043616 | keratinocyte proliferation | 9/887 | 47/18800 | 0.000301 | 0.016227 | 0.014825 | IRF6/FST/MDK/CRNN/EREG/FGF10/REG3G/SDR16C5/HAS2 | 9 |
| BP | GO:0061844 | antimicrobial humoral immune response mediated by antimicrobial peptide | 12/887 | 79/18800 | 0.000313 | 0.016417 | 0.014998 | REG1A/CXCL5/F2/PLA2G1B/KRT6A/CXCL13/S100A7/REG3G/PF4/KLK5/ELANE/REG1B | 12 |
| BP | GO:0061180 | mammary gland epithelium development | 11/887 | 68/18800 | 0.000316 | 0.016417 | 0.014998 | IRF6/PRLR/WNT7B/WNT2/FOXB1/CAV3/MSX2/SOSTDC1/CCL11/WNT4/FGF10 | 11 |
| BP | GO:1900046 | regulation of hemostasis | 11/887 | 68/18800 | 0.000316 | 0.016417 | 0.014998 | F3/F2/SERPINE1/FOXA2/PDGFRA/PLAU/C1QTNF1/APOH/CPB2/EPHB2/COMP | 11 |
| BP | GO:0008209 | androgen metabolic process | 7/887 | 29/18800 | 0.000318 | 0.016417 | 0.014998 | AKR1D1/CYP19A1/WNT4/CYP3A4/HSD17B6/SRD5A2/CYP17A1 | 7 |
| BP | GO:0010838 | positive regulation of keratinocyte proliferation | 5/887 | 14/18800 | 0.000324 | 0.016516 | 0.015088 | MDK/CRNN/FGF10/REG3G/HAS2 | 5 |
| BP | GO:0030216 | keratinocyte differentiation | 19/887 | 167/18800 | 0.000347 | 0.017333 | 0.015835 | IRF6/ETV4/KRT5/KRT4/IVL/KRT79/CNFN/MSX2/KRT6A/EREG/ZBED2/S100A7/KRT16/FOXN1/KRT78/TCHH/KRT6B/REG3G/KLK5 | 19 |
| BP | GO:0007389 | pattern specification process | 39/887 | 463/18800 | 0.000349 | 0.017333 | 0.015835 | FST/LHX2/SFRP2/WNT7B/DLL3/WNT2/DMRTA2/FOXB1/SEMA3A/TLL2/DAND5/FOXG1/BMP5/GREM1/DMRT3/MDFI/BARX1/MSX2/SOSTDC1/SIX2/FOXA1/FOXA2/LEFTY1/DAW1/HOXD12/BASP1/GBX2/FGF10/NKX2-5/TBX18/OTX1/NKX3-2/PITX2/EN1/FGF8/SOX1/LRP2/SIX3/ZIC1 | 39 |
| BP | GO:0070555 | response to interleukin-1 | 17/887 | 141/18800 | 0.000354 | 0.017333 | 0.015835 | IL1R2/ACOD1/NLRP7/CCL25/PCK1/CCL21/PCSK1/CCL7/CCL11/TRIM63/PTGIS/CCL19/PYCARD/EPO/CCL26/HAS2/CCL5 | 17 |
| BP | GO:0071825 | protein-lipid complex subunit organization | 9/887 | 48/18800 | 0.000355 | 0.017333 | 0.015835 | MPO/MFSD2A/PLTP/APOC3/APOC1/APOC2/AGTR1/MTTP/APOA1 | 9 |
| BP | GO:0048665 | neuron fate specification | 7/887 | 30/18800 | 0.000399 | 0.019265 | 0.0176 | DMRTA2/MNX1/DMRT3/ISL2/FOXA1/POU3F2/SOX1 | 7 |
| BP | GO:0061351 | neural precursor cell proliferation | 17/887 | 143/18800 | 0.000418 | 0.020011 | 0.018281 | LHX2/MDK/OTP/WNT2/DMRTA2/FOXG1/TRNP1/PTPRZ1/TRIM71/TAFA3/GBX2/PITX3/FGF8/POU3F2/LRP2/SIX3/ARTN | 17 |
| BP | GO:0052547 | regulation of peptidase activity | 38/887 | 456/18800 | 0.000509 | 0.024098 | 0.022015 | GRIN1/SFRP2/RARRES1/F3/NLRP7/ECM1/SOX2/SERPINE1/PI3/SPINK13/CST6/WFDC10B/PTTG1/ANXA8/ITIH3/ITIH1/AIM2/TFPI2/WFDC5/PI15/HMSD/WFDC12/GRIN2B/TIMP1/AHSG/MMP9/SPINK2/BIRC5/WFDC3/PYCARD/WFDC13/SERPINB5/VIL1/PCOLCE/COL6A3/SLPI/SERPINF1/COL7A1 | 38 |
| BP | GO:0097529 | myeloid leukocyte migration | 23/887 | 229/18800 | 0.000542 | 0.02538 | 0.023186 | MDK/PRTN3/EMILIN1/CXCL5/SCG2/SERPINE1/CCL25/CHGA/GREM1/PLA2G1B/AZU1/CCL21/CXCL13/CYP19A1/CCL7/CCL11/S100A7/EDN3/CD177/PF4/CCL19/CCL26/CCL5 | 23 |
| BP | GO:0072677 | eosinophil migration | 6/887 | 23/18800 | 0.000548 | 0.025442 | 0.023243 | SCG2/CCL21/CCL7/CCL11/CCL26/CCL5 | 6 |
| BP | GO:0048762 | mesenchymal cell differentiation | 24/887 | 244/18800 | 0.000561 | 0.02566 | 0.023443 | MDK/SFRP2/WNT2/ALX1/SEMA3A/SEMA3D/BMP5/GREM1/MSX2/SIX2/FOXA1/FOXA2/WNT10A/IL17RD/EDN3/WNT4/GBX2/FGF10/COL1A1/PITX2/FGF8/HAS2/PDPN/HMGA2 | 24 |
| BP | GO:0001708 | cell fate specification | 13/887 | 96/18800 | 0.000564 | 0.02566 | 0.023443 | SFRP2/DMRTA2/SOX2/MNX1/DMRT3/MYL2/ISL2/SIX2/FOXA1/FOXA2/TBX18/POU3F2/SOX1 | 13 |
| BP | GO:0060415 | muscle tissue morphogenesis | 11/887 | 73/18800 | 0.000592 | 0.026651 | 0.024348 | WNT2/RYR2/MYL2/SHOX2/MYH7/FZD2/NKX2-5/TNNI3/COL11A1/LRP2/MYH6 | 11 |
| BP | GO:0002063 | chondrocyte development | 7/887 | 32/18800 | 0.000607 | 0.027079 | 0.024738 | SFRP2/ECM1/RFLNA/MSX2/SHOX2/COL11A1/COMP | 7 |
| BP | GO:0010810 | regulation of cell-substrate adhesion | 22/887 | 217/18800 | 0.000624 | 0.027574 | 0.025191 | MELTF/MDK/CDKN2A/EMILIN1/SERPINE1/CCL25/ONECUT1/GREM1/EFNA5/CCL21/PLAU/ONECUT2/DMP1/COL26A1/WNT4/COL1A1/PKHD1/EGFL6/HAS2/PDPN/APOA1/MMP12 | 22 |
| BP | GO:0043583 | ear development | 22/887 | 219/18800 | 0.000706 | 0.030908 | 0.028236 | PRRX1/GJB6/SOX2/FOXG1/STRA6/BMP5/FREM2/LIN7A/GABRB2/SIX2/FZD2/GBX2/CHRNA9/FGF10/TBX18/OTX1/NKX3-2/COL11A1/FGF8/EPHB2/CTHRC1/ZIC1 | 22 |
| BP | GO:0003002 | regionalization | 31/887 | 354/18800 | 0.000723 | 0.031118 | 0.028429 | LHX2/SFRP2/WNT7B/DLL3/WNT2/DMRTA2/FOXB1/SEMA3A/TLL2/FOXG1/GREM1/DMRT3/MDFI/BARX1/MSX2/SOSTDC1/SIX2/FOXA1/FOXA2/BASP1/GBX2/FGF10/NKX2-5/TBX18/OTX1/PITX2/EN1/FGF8/SOX1/LRP2/SIX3 | 31 |
| BP | GO:0071347 | cellular response to interleukin-1 | 14/887 | 111/18800 | 0.000728 | 0.031118 | 0.028429 | IL1R2/ACOD1/NLRP7/CCL25/PCK1/CCL21/CCL7/CCL11/PTGIS/CCL19/PYCARD/CCL26/HAS2/CCL5 | 14 |
| BP | GO:0048738 | cardiac muscle tissue development | 23/887 | 234/18800 | 0.000731 | 0.031118 | 0.028429 | GJB6/WNT2/CACNA1G/BMP5/NEB/GREM1/HAMP/RYR2/CAV3/MYL2/MSX2/SHOX2/PDGFRA/MYH7/SOX6/NKX2-5/TBX18/TNNI3/COL11A1/FGF8/LRP2/MYO18B/MYH6 | 23 |
| BP | GO:0034754 | cellular hormone metabolic process | 16/887 | 137/18800 | 0.000743 | 0.031349 | 0.02864 | CRABP1/CRABP2/ADH1C/BMP5/AKR1D1/PDGFRA/AWAT2/CYP19A1/WNT4/CYP3A4/HSD17B6/SRD5A2/RDH16/SDR16C5/CYP17A1/ADH4 | 16 |
| BP | GO:0048247 | lymphocyte chemotaxis | 10/887 | 64/18800 | 0.000777 | 0.032462 | 0.029656 | CCL25/CCL21/CXCL13/CCL7/CCL11/S100A7/C10orf99/CCL19/CCL26/CCL5 | 10 |
| BP | GO:0051480 | regulation of cytosolic calcium ion concentration | 31/887 | 356/18800 | 0.000793 | 0.032854 | 0.030014 | GRIN1/TRPV3/GALR1/CCKBR/HCRT/CD19/JSRP1/GRIN2D/F2/PLA2G1B/RYR2/CAV3/CACNA1B/CCL21/PDGFRA/CXCL13/ADCYAP1/C1QTNF1/SLC8A2/CEMIP/CHRNA9/ADRA1D/GRIN2B/AVPR1B/AGTR1/KISS1/CXCR3/CCL19/PTGER1/EPO/P2RX5 | 31 |
| BP | GO:0006702 | androgen biosynthetic process | 4/887 | 10/18800 | 0.000822 | 0.033181 | 0.030313 | WNT4/HSD17B6/SRD5A2/CYP17A1 | 4 |
| BP | GO:0034379 | very-low-density lipoprotein particle assembly | 4/887 | 10/18800 | 0.000822 | 0.033181 | 0.030313 | MFSD2A/APOC3/APOC1/MTTP | 4 |
| BP | GO:0051918 | negative regulation of fibrinolysis | 4/887 | 10/18800 | 0.000822 | 0.033181 | 0.030313 | F2/SERPINE1/APOH/CPB2 | 4 |
| BP | GO:0035821 | modulation of process of another organism | 6/887 | 25/18800 | 0.000887 | 0.034893 | 0.031877 | IFNG/REG1A/REG3G/FCER2/SLPI/REG1B | 6 |
| BP | GO:0050927 | positive regulation of positive chemotaxis | 6/887 | 25/18800 | 0.000887 | 0.034893 | 0.031877 | F3/SCG2/CASR/AZU1/FGF10/ARTN | 6 |
| BP | GO:0097186 | amelogenesis | 6/887 | 25/18800 | 0.000887 | 0.034893 | 0.031877 | MSX2/DMP1/KLK5/ODAPH/ENAM/WDR72 | 6 |
| BP | GO:0003205 | cardiac chamber development | 18/887 | 167/18800 | 0.000933 | 0.036221 | 0.03309 | SFRP2/WNT2/DAND5/STRA6/BMP5/RYR2/CAV3/MYL2/MSX2/SHOX2/MYH7/FZD2/NKX2-5/TNNI3/COL11A1/FGF8/LRP2/MYH6 | 18 |
| BP | GO:0050921 | positive regulation of chemotaxis | 16/887 | 140/18800 | 0.000941 | 0.036221 | 0.03309 | MDK/F3/SCG2/SERPINE1/CASR/AZU1/CCL21/CXCL13/CCL7/S100A7/EDN3/FGF10/CCL19/CCL26/CCL5/ARTN | 16 |
| BP | GO:0070098 | chemokine-mediated signaling pathway | 12/887 | 89/18800 | 0.000945 | 0.036221 | 0.03309 | CXCL5/CCL25/CCL21/TFF2/CXCL13/CCL7/CCL11/PF4/CXCR3/CCL19/CCL26/CCL5 | 12 |
| BP | GO:0097530 | granulocyte migration | 17/887 | 154/18800 | 0.00098 | 0.037258 | 0.034038 | MDK/PRTN3/CXCL5/SCG2/CCL25/PLA2G1B/CCL21/CXCL13/CCL7/CCL11/S100A7/EDN3/CD177/PF4/CCL19/CCL26/CCL5 | 17 |
| BP | GO:0030193 | regulation of blood coagulation | 10/887 | 66/18800 | 0.000994 | 0.037485 | 0.034245 | F3/F2/SERPINE1/FOXA2/PDGFRA/PLAU/C1QTNF1/APOH/CPB2/EPHB2 | 10 |
| BP | GO:0050920 | regulation of chemotaxis | 22/887 | 225/18800 | 0.001011 | 0.037831 | 0.034561 | MDK/F3/SEMA3A/SCG2/SERPINE1/SEMA3D/CASR/GREM1/AZU1/CCL21/PDGFRA/CXCL13/CYP19A1/CCL7/S100A7/EDN3/FGF10/CCL19/CCL26/CCL5/ELANE/ARTN | 22 |
| BP | GO:0071621 | granulocyte chemotaxis | 15/887 | 128/18800 | 0.001037 | 0.038188 | 0.034887 | MDK/CXCL5/SCG2/CCL25/PLA2G1B/CCL21/CXCL13/CCL7/CCL11/S100A7/EDN3/PF4/CCL19/CCL26/CCL5 | 15 |
| BP | GO:1990266 | neutrophil migration | 15/887 | 128/18800 | 0.001037 | 0.038188 | 0.034887 | MDK/PRTN3/CXCL5/CCL25/PLA2G1B/CCL21/CXCL13/CCL7/CCL11/EDN3/CD177/PF4/CCL19/CCL26/CCL5 | 15 |
| BP | GO:0032103 | positive regulation of response to external stimulus | 36/887 | 442/18800 | 0.001064 | 0.038697 | 0.035352 | IFNG/MDK/HPX/EMILIN1/F3/LAG3/COCH/ACOD1/SCG2/F2/IL1RL1/SERPINE1/CASR/LGALS1/AZU1/CCL21/CXCL13/CCL7/AIM2/TAFA3/EREG/S100A7/EDN3/APOH/FGF10/AGTR1/KLK5/CCL19/PYCARD/CCL26/CPB2/CCL5/IL21/MMP12/ARTN/FABP4 | 36 |
| BP | GO:0052548 | regulation of endopeptidase activity | 35/887 | 426/18800 | 0.001067 | 0.038697 | 0.035352 | GRIN1/SFRP2/RARRES1/F3/NLRP7/SOX2/SERPINE1/PI3/SPINK13/CST6/WFDC10B/PTTG1/ANXA8/ITIH3/ITIH1/AIM2/TFPI2/WFDC5/HMSD/WFDC12/GRIN2B/TIMP1/AHSG/MMP9/SPINK2/BIRC5/WFDC3/PYCARD/WFDC13/SERPINB5/VIL1/COL6A3/SLPI/SERPINF1/COL7A1 | 35 |
| BP | GO:0006641 | triglyceride metabolic process | 13/887 | 103/18800 | 0.001106 | 0.038948 | 0.035581 | AADAC/MFSD2A/CAV3/PCK1/GNB3/APOH/MOGAT1/APOC3/APOC1/PNLIPRP2/APOC2/MTTP/APOA1 | 13 |
| BP | GO:0042472 | inner ear morphogenesis | 13/887 | 103/18800 | 0.001106 | 0.038948 | 0.035581 | PRRX1/FOXG1/FZD2/GBX2/CHRNA9/FGF10/TBX18/OTX1/COL11A1/FGF8/EPHB2/CTHRC1/ZIC1 | 13 |
| BP | GO:0045745 | positive regulation of G protein-coupled receptor signaling pathway | 6/887 | 26/18800 | 0.001108 | 0.038948 | 0.035581 | F2/CHGA/CNTN2/KLK5/GRP/FGF8 | 6 |
| BP | GO:0050926 | regulation of positive chemotaxis | 6/887 | 26/18800 | 0.001108 | 0.038948 | 0.035581 | F3/SCG2/CASR/AZU1/FGF10/ARTN | 6 |
| BP | GO:0030540 | female genitalia development | 5/887 | 18/18800 | 0.001184 | 0.041312 | 0.037741 | STRA6/CYP19A1/FGF10/SRD5A2/LRP2 | 5 |
| BP | GO:0007517 | muscle organ development | 29/887 | 334/18800 | 0.001205 | 0.041731 | 0.038124 | WNT2/DMRTA2/SPEG/TLL2/STRA6/NEB/RYR2/POPDC3/CAV3/MYMX/MYL2/CHRNA1/SHOX2/MYH7/COL19A1/FZD2/BASP1/SOX6/DES/MKX/NKX2-5/TNNI3/COL11A1/PITX1/DNER/FGF8/LRP2/COL6A3/MYH6 | 29 |
| BP | GO:0003163 | sinoatrial node development | 4/887 | 11/18800 | 0.001244 | 0.041769 | 0.038159 | GJB6/CACNA1G/SHOX2/TBX18 | 4 |
| BP | GO:0032429 | regulation of phospholipase A2 activity | 4/887 | 11/18800 | 0.001244 | 0.041769 | 0.038159 | PLA2G1B/ANXA8/AVPR1B/AGTR1 | 4 |
| BP | GO:0042487 | regulation of odontogenesis of dentin-containing tooth | 4/887 | 11/18800 | 0.001244 | 0.041769 | 0.038159 | DMRT3/WNT10A/RSPO2/FGF8 | 4 |
| BP | GO:1904668 | positive regulation of ubiquitin protein ligase activity | 4/887 | 11/18800 | 0.001244 | 0.041769 | 0.038159 | UBE2C/PLK1/UBE2S/CDC20 | 4 |
| BP | GO:0048806 | genitalia development | 8/887 | 46/18800 | 0.001259 | 0.041769 | 0.038159 | STRA6/BMP5/PDGFRA/CYP19A1/FGF10/SRD5A2/FGF8/LRP2 | 8 |
| BP | GO:0002065 | columnar/cuboidal epithelial cell differentiation | 12/887 | 92/18800 | 0.001269 | 0.041769 | 0.038159 | ROS1/IL31RA/SERPINE1/BMP5/FOXA1/NKX6-1/WNT4/NKX3-2/ENAM/FGF8/SPDEF/NKX6-3 | 12 |
| BP | GO:0009953 | dorsal/ventral pattern formation | 12/887 | 92/18800 | 0.001269 | 0.041769 | 0.038159 | LHX2/TLL2/FOXG1/GREM1/DMRT3/MDFI/SOSTDC1/FOXA1/EN1/FGF8/SOX1/SIX3 | 12 |
| BP | GO:0048644 | muscle organ morphogenesis | 11/887 | 80/18800 | 0.001292 | 0.042211 | 0.038562 | WNT2/RYR2/MYL2/SHOX2/MYH7/FZD2/NKX2-5/TNNI3/COL11A1/LRP2/MYH6 | 11 |
| BP | GO:0006639 | acylglycerol metabolic process | 15/887 | 131/18800 | 0.001315 | 0.042365 | 0.038703 | AADAC/MFSD2A/DGKI/CAV3/PCK1/AWAT2/GNB3/APOH/MOGAT1/APOC3/APOC1/PNLIPRP2/APOC2/MTTP/APOA1 | 15 |
| BP | GO:0060078 | regulation of postsynaptic membrane potential | 15/887 | 131/18800 | 0.001315 | 0.042365 | 0.038703 | GRIN1/P2RX6/HCRT/GRIN2D/MAPK8IP2/GABRG1/GABRB2/CHRNA1/GRIK4/SLC8A2/CHRNA9/GRIN2B/GLRA3/GABRA3/P2RX5 | 15 |
| BP | GO:0009913 | epidermal cell differentiation | 22/887 | 230/18800 | 0.001346 | 0.043068 | 0.039346 | IRF6/ETV4/KRT5/KRT4/IVL/KRT79/CNFN/SULT2B1/MSX2/KRT6A/EREG/ZBED2/S100A7/KRT16/FOXN1/KRT78/TCHH/KRT6B/REG3G/KLK5/PITX2/SFRP4 | 22 |
| BP | GO:0006706 | steroid catabolic process | 6/887 | 27/18800 | 0.001368 | 0.043192 | 0.039458 | AKR1D1/CYP19A1/CYP3A4/HSD17B6/SRD5A2/HSD11B1 | 6 |
| BP | GO:0034505 | tooth mineralization | 6/887 | 27/18800 | 0.001368 | 0.043192 | 0.039458 | MSX2/DMP1/COL1A1/ODAPH/ENAM/WDR72 | 6 |
| BP | GO:0010469 | regulation of signaling receptor activity | 18/887 | 173/18800 | 0.0014 | 0.043903 | 0.040108 | IFNG/CACNG7/MTRNR2L3/CGA/MTRNR2L6/CACNG4/SERPINE1/GREM1/MAPK8IP2/LYPD1/CAV3/MTRNR2L1/PLAU/EREG/DKKL1/LY6H/EPHB2/CBLC | 18 |
| BP | GO:0006638 | neutral lipid metabolic process | 15/887 | 132/18800 | 0.00142 | 0.044228 | 0.040405 | AADAC/MFSD2A/DGKI/CAV3/PCK1/AWAT2/GNB3/APOH/MOGAT1/APOC3/APOC1/PNLIPRP2/APOC2/MTTP/APOA1 | 15 |
| BP | GO:0031016 | pancreas development | 11/887 | 81/18800 | 0.001432 | 0.044309 | 0.040479 | ILDR2/CELA1/ONECUT1/BMP5/MNX1/FOXA2/PCSK1/ONECUT2/NKX6-1/FGF10/NKX3-2 | 11 |
| BP | GO:0030593 | neutrophil chemotaxis | 13/887 | 106/18800 | 0.001444 | 0.044377 | 0.040541 | MDK/CXCL5/CCL25/PLA2G1B/CCL21/CXCL13/CCL7/CCL11/EDN3/PF4/CCL19/CCL26/CCL5 | 13 |
| BP | GO:0055010 | ventricular cardiac muscle tissue morphogenesis | 8/887 | 47/18800 | 0.001455 | 0.044421 | 0.040582 | RYR2/MYL2/MYH7/NKX2-5/TNNI3/COL11A1/LRP2/MYH6 | 8 |
| BP | GO:0030178 | negative regulation of Wnt signaling pathway | 18/887 | 174/18800 | 0.001495 | 0.045336 | 0.041417 | IGFBP1/MDK/SFRP2/TRABD2A/TPBGL/SOX2/WIF1/GREM1/SFRP5/MDFI/BARX1/SOSTDC1/DKKL1/APCDD1L/NKX2-5/TBX18/SFRP4/CTHRC1 | 18 |
| BP | GO:0002685 | regulation of leukocyte migration | 21/887 | 218/18800 | 0.001568 | 0.047217 | 0.043136 | MDK/EMILIN1/ECM1/SERPINE1/CCL25/BMP5/GREM1/CCL21/CXCL13/CYP19A1/CCL7/S100A7/EDN3/C10orf99/CHST4/CXCR3/CCL19/PYCARD/CCL5/ELANE/TNFRSF18 | 21 |
| BP | GO:0002548 | monocyte chemotaxis | 10/887 | 70/18800 | 0.001577 | 0.047217 | 0.043136 | SERPINE1/CCL25/GREM1/CCL21/CCL7/CCL11/S100A7/CCL19/CCL26/CCL5 | 10 |
| BP | GO:0014706 | striated muscle tissue development | 23/887 | 248/18800 | 0.001596 | 0.047477 | 0.043373 | GJB6/WNT2/CACNA1G/BMP5/NEB/GREM1/HAMP/RYR2/CAV3/MYL2/MSX2/SHOX2/PDGFRA/MYH7/SOX6/NKX2-5/TBX18/TNNI3/COL11A1/FGF8/LRP2/MYO18B/MYH6 | 23 |
| BP | GO:0048562 | embryonic organ morphogenesis | 26/887 | 294/18800 | 0.001629 | 0.048147 | 0.043985 | PRRX1/GJB6/ALX1/FOXG1/STRA6/RYR2/MFAP2/MDFI/FOXE1/SIX2/SHOX2/PDGFRA/FZD2/GBX2/CHRNA9/FGF10/NKX2-5/TBX18/OTX1/NKX3-2/COL11A1/FGF8/EPHB2/SIX3/CTHRC1/ZIC1 | 26 |
| BP | GO:0006953 | acute-phase response | 8/887 | 48/18800 | 0.001675 | 0.049197 | 0.044945 | F2/HAMP/ORM1/ORM2/CRP/AHSG/REG3G/EPO | 8 |
| BP | GO:0007218 | neuropeptide signaling pathway | 13/887 | 108/18800 | 0.001715 | 0.050034 | 0.04571 | GPR84/GALR1/NPBWR1/HCRT/NMU/OPRD1/NPFFR2/ADCYAP1/SCG5/SSTR3/TAC3/GLRA3/GRP | 13 |
| BP | GO:0050818 | regulation of coagulation | 10/887 | 71/18800 | 0.00176 | 0.051035 | 0.046624 | F3/F2/SERPINE1/FOXA2/PDGFRA/PLAU/C1QTNF1/APOH/CPB2/EPHB2 | 10 |
| BP | GO:0006869 | lipid transport | 32/887 | 391/18800 | 0.001821 | 0.05231 | 0.047789 | CRABP1/ERFE/GALR1/CRABP2/ABCA13/MFSD2A/CYP4A11/SLC27A2/APOL1/STRA6/PLTP/PLA2G1B/XKR7/SLC10A2/CYP19A1/C1QTNF1/FABP6/APOLD1/SLC22A24/APOH/APOC3/APOC1/APOC2/AVPR1B/AGTR1/CIDEC/KCNN4/MTTP/PLA2G2D/SLC5A8/APOA1/FABP4 | 32 |
| BP | GO:0007548 | sex differentiation | 25/887 | 281/18800 | 0.001826 | 0.05231 | 0.047789 | FST/SFRP2/DMRTA2/SEMA3A/PTX3/STRA6/BMP5/DMRT3/NUDT1/PDGFRA/AMH/CYP19A1/INSL3/EREG/BASP1/WNT4/FGF10/SRD5A2/PTPRN/FGF8/CYP17A1/TEX19/LRP2/VGF/LHX8 | 25 |
| BP | GO:0030595 | leukocyte chemotaxis | 22/887 | 236/18800 | 0.001869 | 0.053212 | 0.048612 | MDK/CXCL5/SCG2/SERPINE1/CCL25/CHGA/GREM1/PLA2G1B/AZU1/CCL21/CXCL13/CYP19A1/CCL7/CCL11/S100A7/EDN3/C10orf99/PF4/CCL19/CNR2/CCL26/CCL5 | 22 |
| BP | GO:0055008 | cardiac muscle tissue morphogenesis | 9/887 | 60/18800 | 0.001889 | 0.05342 | 0.048803 | WNT2/RYR2/MYL2/MYH7/NKX2-5/TNNI3/COL11A1/LRP2/MYH6 | 9 |
| BP | GO:0030900 | forebrain development | 31/887 | 376/18800 | 0.0019 | 0.05342 | 0.048803 | LHX2/MDK/WNT7B/CDK5R2/APLP1/OTP/DMRTA2/MFSD2A/FOXB1/SEMA3A/SOX2/EFNA2/FOXG1/KIRREL3/CNTN2/PCSK1/WNT4/GBX2/SSTR3/FGF10/SRD5A2/OTX1/PITX1/PITX2/FGF8/POU3F2/SOX1/EPHB2/LRP2/LHX8/SIX3 | 31 |
| BP | GO:1900047 | negative regulation of hemostasis | 8/887 | 49/18800 | 0.001921 | 0.05369 | 0.049049 | F2/SERPINE1/PDGFRA/PLAU/C1QTNF1/APOH/CPB2/COMP | 8 |
| BP | GO:0051004 | regulation of lipoprotein lipase activity | 5/887 | 20/18800 | 0.001981 | 0.054389 | 0.049688 | APOH/APOC3/APOC1/APOC2/APOA1 | 5 |
| BP | GO:0070166 | enamel mineralization | 5/887 | 20/18800 | 0.001981 | 0.054389 | 0.049688 | MSX2/DMP1/ODAPH/ENAM/WDR72 | 5 |
| BP | GO:0140131 | positive regulation of lymphocyte chemotaxis | 5/887 | 20/18800 | 0.001981 | 0.054389 | 0.049688 | CCL21/CXCL13/CCL7/S100A7/CCL5 | 5 |
| BP | GO:1990868 | response to chemokine | 12/887 | 97/18800 | 0.002013 | 0.054615 | 0.049894 | CXCL5/CCL25/CCL21/TFF2/CXCL13/CCL7/CCL11/PF4/CXCR3/CCL19/CCL26/CCL5 | 12 |
| BP | GO:1990869 | cellular response to chemokine | 12/887 | 97/18800 | 0.002013 | 0.054615 | 0.049894 | CXCL5/CCL25/CCL21/TFF2/CXCL13/CCL7/CCL11/PF4/CXCR3/CCL19/CCL26/CCL5 | 12 |
| CC | GO:0019814 | immunoglobulin complex | 80/930 | 167/19594 | 9.85E-61 | 4.60E-58 | 4.04E-58 | IGKV2-24/TRBC2/IGKV2-29/IGHV4-59/IGLV2-23/IGLV1-36/IGLV2-33/IGLV3-9/IGLV9-49/IGLC2/IGHV5-10-1/IGLV4-60/IGHV3-33/IGLC3/IGHV3-35/IGKC/IGKV1-9/IGHV1-69/IGKV2-28/IGKV1-17/IGKV2D-24/IGHV3-21/IGHV3-73/IGHV6-1/IGHV1-69D/IGHA2/IGLV10-54/IGLV1-44/IGHA1/IGHV3-64D/IGLV3-10/IGHJ1/IGHG2/IGHG1/IGKV1-6/IGHV3-49/IGKV3D-15/IGLV5-37/IGLV7-46/IGLV7-43/IGKV1D-13/IGKV1-13/IGHV3-72/IGKV2D-28/IGKV3-15/IGHV2-70/IGHV2-5/IGHV3-13/IGLV3-19/IGLV2-14/IGLL5/IGLC6/IGLV6-57/IGLV1-51/IGLV1-40/IGLV1-47/IGLV3-16/IGHG4/IGKV1D-17/IGHV4-34/IGLC7/IGKV6D-21/IGHV5-51/IGHV4-39/IGLV8-61/IGKV1D-43/CD79A/IGLV2-11/IGKV3D-11/IGLV3-27/IGLV4-69/IGHV1-18/IGHD/IGHV3-11/IGHV3-30/IGKV3D-20/IGHM/IGKV3-20/IGHV4-4/IGKV1D-33 | 80 |
| CC | GO:0042571 | immunoglobulin complex, circulating | 37/930 | 77/19594 | 1.11E-28 | 2.60E-26 | 2.28E-26 | TRBC2/IGHV4-59/IGLC2/IGHV5-10-1/IGHV3-33/IGLC3/IGHV3-35/IGKC/IGHV1-69/IGHV3-21/IGHV3-73/IGHV6-1/IGHV1-69D/IGHA2/IGHA1/IGHV3-64D/IGHG2/IGHG1/IGHV3-49/IGHV3-72/IGHV2-70/IGHV2-5/IGHV3-13/IGLL5/IGLC6/IGHG4/IGHV4-34/IGLC7/IGHV5-51/IGHV4-39/IGHV1-18/IGHD/IGHV3-11/IGHV3-30/IGHM/IGKV3-20/IGHV4-4 | 37 |
| CC | GO:0062023 | collagen-containing extracellular matrix | 68/930 | 429/19594 | 1.27E-18 | 1.97E-16 | 1.74E-16 | MDK/SFRP2/PRTN3/HPX/APLP1/THBS2/SRPX/EMILIN1/SRPX2/WNT2/F3/CILP/COCH/ECM1/F2/SERPINE1/GREM1/LGALS1/FREM2/FREM1/MFAP2/LOXL1/PTPRZ1/ANXA8/EFNA5/ORM1/TGFBI/ITIH1/COL25A1/MATN4/COL10A1/INHBE/ORM2/COL19A1/S100A7/COL26A1/SPON1/COL5A1/APOH/PRG4/RTBDN/LUM/APOC3/LRRC15/FGF10/TIMP1/L1CAM/AHSG/MMP9/MFAP4/MMP23B/PF4/COL1A1/COL11A1/ADAMDEC1/EGFL6/COL1A2/COL8A2/COMP/PCOLCE/ZP1/APOA1/ELANE/COL6A3/CTHRC1/SLPI/SERPINF1/COL7A1 | 68 |
| CC | GO:0072562 | blood microparticle | 36/930 | 147/19594 | 1.95E-16 | 2.28E-14 | 2.01E-14 | HPX/IGLC2/C1R/CFHR3/PON1/CFHR1/IGLC3/TF/IGKC/HBA2/F2/APOL1/IGKV1-17/ITGA2B/IGHA2/IGHA1/IGHG2/ORM1/IGHG1/ITIH1/ORM2/IGKV2D-28/IGKV3-15/IGHV3-13/IGLV1-47/AHSG/IGHG4/HPR/C8G/IGKV3D-11/HBA1/IGHD/IGHM/APOA1/IGKV3-20/IGKV1D-33 | 36 |
| CC | GO:0009897 | external side of plasma membrane | 63/930 | 455/19594 | 1.82E-14 | 1.70E-12 | 1.49E-12 | CA4/PDCD1/PRLR/TRBC2/IGHV4-59/IL31RA/WNT2/IGLC2/F3/LAG3/IGHV5-10-1/IGHV3-33/CD19/IGLC3/IL2RG/IGHV3-35/TF/IGKC/F2/IL1RL1/IGHV1-69/ITGA2B/IGHV3-21/IGHV3-73/IGHV6-1/IGHV1-69D/IGHA2/IGHA1/IGHV3-64D/EFNA5/IGHG2/IGHG1/SLAMF9/IGHV3-49/CEACAM5/GFRA3/PDGFRA/ASGR1/IGHV3-72/IGHV2-70/IGHV2-5/IGHV3-13/IGLL5/IGLC6/RTBDN/IGHG4/TRPM8/CXCR3/PKHD1/IGHV4-34/IGLC7/IGHV5-51/IGHV4-39/CD79A/LRP2/IGHV1-18/IGHD/IGHV3-11/IGHV3-30/IGHM/TNFRSF18/FCER2/IGHV4-4 | 63 |
| CC | GO:0005581 | collagen trimer | 17/930 | 86/19594 | 4.93E-07 | 3.83E-05 | 3.37E-05 | EMILIN1/COL22A1/COL25A1/COL10A1/C1QTNF1/COL19A1/COL26A1/COL5A1/LUM/COL1A1/COL11A1/C1QL1/COL1A2/COL8A2/COL6A3/CTHRC1/COL7A1 | 17 |
| CC | GO:0034364 | high-density lipoprotein particle | 9/930 | 27/19594 | 2.54E-06 | 0.00017 | 0.000149 | PON1/APOL1/PLTP/APOH/APOC3/APOC1/APOC2/HPR/APOA1 | 9 |
| CC | GO:0005788 | endoplasmic reticulum lumen | 34/930 | 311/19594 | 5.48E-06 | 0.00032 | 0.000281 | IGFBP1/MELTF/WNT7B/CHGB/COL22A1/SLC27A2/TF/SCG2/COLGALT2/F2/APOL1/LGALS1/COL25A1/P4HA3/COL10A1/COL19A1/DMP1/COL26A1/SPON1/WNT4/COL5A1/TIMP1/AHSG/COL1A1/COL11A1/MTTP/ENAM/COL1A2/MZB1/COL8A2/VGF/APOA1/COL6A3/COL7A1 | 34 |
| CC | GO:0034358 | plasma lipoprotein particle | 9/930 | 36/19594 | 3.47E-05 | 0.001622 | 0.001426 | PON1/APOL1/PLTP/APOH/APOC3/APOC1/APOC2/HPR/APOA1 | 9 |
| CC | GO:1990777 | lipoprotein particle | 9/930 | 36/19594 | 3.47E-05 | 0.001622 | 0.001426 | PON1/APOL1/PLTP/APOH/APOC3/APOC1/APOC2/HPR/APOA1 | 9 |
| CC | GO:0098644 | complex of collagen trimers | 7/930 | 22/19594 | 4.83E-05 | 0.002051 | 0.001803 | COL10A1/COL5A1/LUM/COL1A1/COL11A1/COL1A2/COL7A1 | 7 |
| CC | GO:0032994 | protein-lipid complex | 9/930 | 39/19594 | 6.88E-05 | 0.002677 | 0.002353 | PON1/APOL1/PLTP/APOH/APOC3/APOC1/APOC2/HPR/APOA1 | 9 |
| CC | GO:0005583 | fibrillar collagen trimer | 5/930 | 12/19594 | 0.000143 | 0.00476 | 0.004185 | COL5A1/LUM/COL1A1/COL11A1/COL1A2 | 5 |
| CC | GO:0098643 | banded collagen fibril | 5/930 | 12/19594 | 0.000143 | 0.00476 | 0.004185 | COL5A1/LUM/COL1A1/COL11A1/COL1A2 | 5 |
| CC | GO:0042627 | chylomicron | 5/930 | 13/19594 | 0.000223 | 0.006749 | 0.005933 | APOH/APOC3/APOC1/APOC2/APOA1 | 5 |
| CC | GO:0034361 | very-low-density lipoprotein particle | 6/930 | 20/19594 | 0.000246 | 0.006749 | 0.005933 | APOL1/APOH/APOC3/APOC1/APOC2/APOA1 | 6 |
| CC | GO:0034385 | triglyceride-rich plasma lipoprotein particle | 6/930 | 20/19594 | 0.000246 | 0.006749 | 0.005933 | APOL1/APOH/APOC3/APOC1/APOC2/APOA1 | 6 |
| CC | GO:0005604 | basement membrane | 13/930 | 95/19594 | 0.00054 | 0.013918 | 0.012235 | APLP1/THBS2/FREM2/FREM1/LOXL1/EFNA5/TGFBI/COL5A1/TIMP1/EGFL6/COL8A2/SERPINF1/COL7A1 | 13 |
| CC | GO:0071682 | endocytic vesicle lumen | 6/930 | 23/19594 | 0.000566 | 0.013918 | 0.012235 | SCGB3A2/HPX/MPO/HBA2/HBA1/APOA1 | 6 |
| CC | GO:0031233 | intrinsic component of external side of plasma membrane | 6/930 | 24/19594 | 0.000725 | 0.016927 | 0.01488 | CA4/F3/EFNA5/CEACAM5/RTBDN/PKHD1 | 6 |
| CC | GO:0001533 | cornified envelope | 8/930 | 45/19594 | 0.001127 | 0.025068 | 0.022036 | PI3/CST6/IVL/CNFN/PKP3/RPTN/DSG3/TCHH | 8 |
| CC | GO:0043025 | neuronal cell body | 38/930 | 482/19594 | 0.001588 | 0.033703 | 0.029627 | SYT5/CACNG7/DPYSL5/P2RX6/MAST1/EFNA2/CASR/MAPK8IP2/CPNE6/CACNA1B/UNC5A/CNTN2/ATP1A3/ADCYAP1/PCSK1/SLC8A2/BRS3/DRP2/L1CAM/SRD5A2/AVPR1B/GLRA3/KISS1/CPLX2/CNR2/PTPRN/KCNN4/PYCARD/DNER/STMN2/TPX2/CYP17A1/EPHB2/VGF/OPN4/CNGA3/TACR3/SERPINF1 | 38 |
| CC | GO:0005833 | hemoglobin complex | 4/930 | 12/19594 | 0.001837 | 0.037308 | 0.032797 | AHSP/HBA2/HBQ1/HBA1 | 4 |
| CC | GO:0098936 | intrinsic component of postsynaptic membrane | 14/930 | 123/19594 | 0.002104 | 0.040934 | 0.035984 | CACNG7/P2RX6/CACNG4/LRRTM2/GRIN2D/OPRD1/GABRB2/CNTN2/CHRNA1/GRIK4/SLC6A11/CHRNA9/EPHB2/GABRA3 | 14 |
| CC | GO:0098992 | neuronal dense core vesicle | 4/930 | 13/19594 | 0.002555 | 0.047728 | 0.041957 | SYT5/SCG2/CHGA/OPRD1 | 4 |
| CC | GO:0043679 | axon terminus | 13/930 | 113/19594 | 0.002718 | 0.048813 | 0.04291 | GRIN1/UNC13A/CASR/NMU/OPRD1/PCSK1/SLC8A2/AVPR1B/SLC18A3/SLC18A2/CPLX2/PTPRN/PRKCG | 13 |
| CC | GO:0045095 | keratin filament | 12/930 | 102/19594 | 0.003236 | 0.054585 | 0.047984 | KRT13/KRT5/KRT4/KRT79/KRT6A/KRT25/KRT78/KRT6B/KRTAP16-1/KRTAP2-3/KRT14/KRTAP1-5 | 12 |
| CC | GO:0044306 | neuron projection terminus | 14/930 | 129/19594 | 0.003276 | 0.054585 | 0.047984 | GRIN1/UNC13A/SCRG1/CASR/NMU/OPRD1/PCSK1/SLC8A2/AVPR1B/SLC18A3/SLC18A2/CPLX2/PTPRN/PRKCG | 14 |
| CC | GO:0099060 | integral component of postsynaptic specialization membrane | 10/930 | 77/19594 | 0.00339 | 0.054585 | 0.047984 | CACNG7/P2RX6/CACNG4/LRRTM2/GRIN2D/OPRD1/GABRB2/CHRNA1/CHRNA9/GABRA3 | 10 |
| MF | GO:0003823 | antigen binding | 56/853 | 174/18410 | 3.72E-32 | 2.85E-29 | 2.44E-29 | TRBC2/IGHV4-59/IGLV2-23/IGLC2/LAG3/IGHV5-10-1/IGHV3-33/IGLC3/IGHV3-35/IGKC/IGHV1-69/IGKV1-17/IGHV3-21/IGHV3-73/IGHV6-1/IGHV1-69D/IGHA2/IGLV1-44/IGHA1/IGHV3-64D/IGHG2/IGHG1/IGHV3-49/IGLV7-43/IGHV3-72/IGKV2D-28/IGKV3-15/IGHV2-70/IGHV2-5/IGHV3-13/IGLV3-19/IGLV2-14/IGLL5/IGLC6/IGLV6-57/IGLV1-51/IGLV1-40/IGLV1-47/IGHG4/MFAP4/IGHV4-34/IGLC7/IGHV5-51/IGHV4-39/IGLV2-11/IGKV3D-11/IGLV3-27/IGHV1-18/IGHD/IGHV3-11/IGHV3-30/IGHM/IGKV3-20/NCR3LG1/IGHV4-4/IGKV1D-33 | 56 |
| MF | GO:0034987 | immunoglobulin receptor binding | 36/853 | 80/18410 | 4.74E-27 | 1.82E-24 | 1.55E-24 | TRBC2/IGHV4-59/IGLC2/IGHV5-10-1/IGHV3-33/IGLC3/IGHV3-35/IGKC/IGHV1-69/IGHV3-21/IGHV3-73/IGHV6-1/IGHV1-69D/IGHA2/IGHA1/IGHV3-64D/IGHG2/IGHG1/IGHV3-49/IGHV3-72/IGHV2-70/IGHV2-5/IGHV3-13/IGLL5/IGLC6/IGHG4/IGHV4-34/IGLC7/IGHV5-51/IGHV4-39/IGHV1-18/IGHD/IGHV3-11/IGHV3-30/IGHM/IGHV4-4 | 36 |
| MF | GO:0048018 | receptor ligand activity | 60/853 | 489/18410 | 5.13E-12 | 1.31E-09 | 1.12E-09 | IFNG/MDK/ERFE/SFRP2/WNT7B/CGA/CHGB/WNT2/GDF5/REG1A/HCRT/LTB/CXCL5/IL11/SEMA3A/SCG2/F2/CCL25/SEMA3D/DAND5/IL19/BMP5/GREM1/HAMP/EFNA5/CCL21/LEFTY1/CXCL13/AMH/ADCYAP1/WNT10A/CCL7/INSL3/INHBE/FGF5/TAFA3/EREG/CCL11/TFF1/EDN3/WNT4/FGF10/TIMP1/IFNL1/C10orf99/PF4/CCL19/GRP/EPO/FGF8/CSF2/CCL26/VGF/CCL5/IL21/APOA1/IL17C/UCN2/ARTN/IL17B | 60 |
| MF | GO:0030546 | signaling receptor activator activity | 60/853 | 496/18410 | 9.34E-12 | 1.79E-09 | 1.53E-09 | IFNG/MDK/ERFE/SFRP2/WNT7B/CGA/CHGB/WNT2/GDF5/REG1A/HCRT/LTB/CXCL5/IL11/SEMA3A/SCG2/F2/CCL25/SEMA3D/DAND5/IL19/BMP5/GREM1/HAMP/EFNA5/CCL21/LEFTY1/CXCL13/AMH/ADCYAP1/WNT10A/CCL7/INSL3/INHBE/FGF5/TAFA3/EREG/CCL11/TFF1/EDN3/WNT4/FGF10/TIMP1/IFNL1/C10orf99/PF4/CCL19/GRP/EPO/FGF8/CSF2/CCL26/VGF/CCL5/IL21/APOA1/IL17C/UCN2/ARTN/IL17B | 60 |
| MF | GO:0005201 | extracellular matrix structural constituent | 30/853 | 172/18410 | 3.21E-10 | 4.92E-08 | 4.21E-08 | THBS2/SRPX/EMILIN1/SRPX2/CILP/COL22A1/ECM1/MFAP2/TGFBI/COL25A1/COL10A1/ZPLD1/COL19A1/SPON1/TFPI2/COL5A1/PRG4/LUM/MFAP4/COL1A1/COL11A1/ENAM/COL1A2/COL8A2/COMP/PCOLCE/ZP1/COL6A3/CTHRC1/COL7A1 | 30 |
| MF | GO:0005539 | glycosaminoglycan binding | 32/853 | 234/18410 | 4.06E-08 | 4.92E-06 | 4.21E-06 | MDK/EPYC/APLP1/THBS2/MPO/REG1A/F2/RSPO4/AZU1/ITIH1/COL25A1/CXCL13/LRTM2/CCL7/RSPO2/CEMIP/COL5A1/APOH/FGF10/CCN5/REG3G/PGLYRP2/PF4/COL11A1/PRSS57/NCAN/COMP/PCOLCE/PLA2G2D/IGHM/ELANE/REG1B | 32 |
| MF | GO:0005125 | cytokine activity | 32/853 | 235/18410 | 4.50E-08 | 4.92E-06 | 4.21E-06 | IFNG/WNT7B/WNT2/GDF5/LTB/CXCL5/IL11/SCG2/CCL25/IL19/BMP5/GREM1/CCL21/LEFTY1/CXCL13/WNT10A/CCL7/INHBE/CCL11/WNT4/TIMP1/IFNL1/C10orf99/PF4/CCL19/EPO/CSF2/CCL26/CCL5/IL21/IL17C/IL17B | 32 |
| MF | GO:0030414 | peptidase inhibitor activity | 27/853 | 187/18410 | 1.51E-07 | 1.45E-05 | 1.24E-05 | RARRES1/NLRP7/SERPINE1/PI3/SPINK13/CST6/WFDC10B/PTTG1/ITIH3/ITIH1/TFPI2/WFDC5/PI15/HMSD/WFDC12/TIMP1/AHSG/SPINK2/BIRC5/WFDC3/WFDC13/SERPINB5/VIL1/COL6A3/SLPI/SERPINF1/COL7A1 | 27 |
| MF | GO:0004866 | endopeptidase inhibitor activity | 26/853 | 180/18410 | 2.54E-07 | 2.16E-05 | 1.85E-05 | RARRES1/NLRP7/SERPINE1/PI3/SPINK13/CST6/WFDC10B/PTTG1/ITIH3/ITIH1/TFPI2/WFDC5/HMSD/WFDC12/TIMP1/AHSG/SPINK2/BIRC5/WFDC3/WFDC13/SERPINB5/VIL1/COL6A3/SLPI/SERPINF1/COL7A1 | 26 |
| MF | GO:0061134 | peptidase regulator activity | 30/853 | 230/18410 | 3.07E-07 | 2.25E-05 | 1.92E-05 | SFRP2/RARRES1/NLRP7/SERPINE1/PI3/SPINK13/CST6/WFDC10B/PTTG1/ITIH3/ITIH1/TFPI2/WFDC5/PI15/HMSD/WFDC12/TIMP1/AHSG/SPINK2/BIRC5/WFDC3/PYCARD/WFDC13/SERPINB5/VIL1/PCOLCE/COL6A3/SLPI/SERPINF1/COL7A1 | 30 |
| MF | GO:0061135 | endopeptidase regulator activity | 27/853 | 194/18410 | 3.22E-07 | 2.25E-05 | 1.92E-05 | SFRP2/RARRES1/NLRP7/SERPINE1/PI3/SPINK13/CST6/WFDC10B/PTTG1/ITIH3/ITIH1/TFPI2/WFDC5/HMSD/WFDC12/TIMP1/AHSG/SPINK2/BIRC5/WFDC3/WFDC13/SERPINB5/VIL1/COL6A3/SLPI/SERPINF1/COL7A1 | 27 |
| MF | GO:0017171 | serine hydrolase activity | 27/853 | 195/18410 | 3.58E-07 | 2.29E-05 | 1.96E-05 | PRTN3/DPP6/F3/AADAC/C1R/CELA1/PRSS3/F2/TLL2/PRSS2/PRSS21/TMPRSS11D/AZU1/PCSK1/PLAU/TMPRSS6/KLK11/KLK13/MMP9/HPR/KLK5/PRSS57/KLK10/ELANE/MMP7/MMP12/MMP13 | 27 |
| MF | GO:0004252 | serine-type endopeptidase activity | 25/853 | 174/18410 | 4.76E-07 | 2.76E-05 | 2.36E-05 | PRTN3/F3/C1R/CELA1/PRSS3/F2/TLL2/PRSS2/PRSS21/TMPRSS11D/AZU1/PCSK1/PLAU/TMPRSS6/KLK11/KLK13/MMP9/HPR/KLK5/PRSS57/KLK10/ELANE/MMP7/MMP12/MMP13 | 25 |
| MF | GO:0004867 | serine-type endopeptidase inhibitor activity | 18/853 | 98/18410 | 5.04E-07 | 2.76E-05 | 2.36E-05 | SERPINE1/PI3/SPINK13/WFDC10B/ITIH3/ITIH1/TFPI2/WFDC5/HMSD/WFDC12/SPINK2/WFDC3/WFDC13/SERPINB5/COL6A3/SLPI/SERPINF1/COL7A1 | 18 |
| MF | GO:0008236 | serine-type peptidase activity | 26/853 | 191/18410 | 8.19E-07 | 4.18E-05 | 3.58E-05 | PRTN3/DPP6/F3/C1R/CELA1/PRSS3/F2/TLL2/PRSS2/PRSS21/TMPRSS11D/AZU1/PCSK1/PLAU/TMPRSS6/KLK11/KLK13/MMP9/HPR/KLK5/PRSS57/KLK10/ELANE/MMP7/MMP12/MMP13 | 26 |
| MF | GO:0008201 | heparin binding | 23/853 | 168/18410 | 3.16E-06 | 0.000151 | 0.00013 | MDK/APLP1/THBS2/MPO/F2/RSPO4/AZU1/COL25A1/CXCL13/LRTM2/CCL7/RSPO2/COL5A1/APOH/FGF10/CCN5/PF4/COL11A1/PRSS57/COMP/PCOLCE/PLA2G2D/ELANE | 23 |
| MF | GO:0008009 | chemokine activity | 11/853 | 49/18410 | 1.15E-05 | 0.000503 | 0.000431 | CXCL5/CCL25/CCL21/CXCL13/CCL7/CCL11/C10orf99/PF4/CCL19/CCL26/CCL5 | 11 |
| MF | GO:0001664 | G protein-coupled receptor binding | 31/853 | 288/18410 | 1.18E-05 | 0.000503 | 0.000431 | WNT7B/APLP1/CCKBR/WNT2/HCRT/REEP2/CXCL5/CCL25/NMU/CCL21/TFF2/NPFFR2/CXCL13/ADCYAP1/WNT10A/CCL7/INSL3/CCL11/EDN3/WNT4/RTP3/AGTR1/C10orf99/KISS1/PF4/CCL19/PTGER1/CCL26/CCL5/CTHRC1/UCN2 | 31 |
| MF | GO:0030020 | extracellular matrix structural constituent conferring tensile strength | 10/853 | 41/18410 | 1.31E-05 | 0.000528 | 0.000452 | COL25A1/COL10A1/COL19A1/COL5A1/COL1A1/COL11A1/COL1A2/COL8A2/COL6A3/COL7A1 | 10 |
| MF | GO:0008237 | metallopeptidase activity | 23/853 | 189/18410 | 2.27E-05 | 0.000828 | 0.000709 | ADAMTS14/TRABD2A/ADAM33/CLCA2/TLL2/PRSS2/CPZ/CPA2/ADAM12/TMPRSS6/CPA1/MMP9/MMP23B/TRHDE/ADAMDEC1/CPXM1/CPB2/ADAM7/CPA5/CPA4/MMP7/MMP12/MMP13 | 23 |
| MF | GO:0005200 | structural constituent of cytoskeleton | 16/853 | 104/18410 | 2.27E-05 | 0.000828 | 0.000709 | ACTBL2/TUBA3D/TUBA3C/INA/KRT5/TUBAL3/KRT19/KRT15/TUBB3/KRT6A/ADD2/KRT16/DES/KRT6B/GFAP/KRT14 | 16 |
| MF | GO:0004857 | enzyme inhibitor activity | 37/853 | 390/18410 | 3.06E-05 | 0.001066 | 0.000913 | CDKN2A/RARRES1/NLRP7/SERPINE1/PI3/SPINK13/CST6/WFDC10B/SLN/PTTG1/ITIH3/ITIH1/PPP1R27/SCG5/CAMK2N2/TFPI2/WFDC5/PI15/HMSD/APOC3/APOC1/APOC2/WFDC12/TIMP1/AHSG/PPP1R1A/SPINK2/BIRC5/WFDC3/WFDC13/SERPINB5/VIL1/GCKR/COL6A3/SLPI/SERPINF1/COL7A1 | 37 |
| MF | GO:0017147 | Wnt-protein binding | 8/853 | 30/18410 | 4.84E-05 | 0.001573 | 0.001346 | SFRP2/TRABD2A/WIF1/SFRP5/FZD2/APCDD1L/SFRP4/CTHRC1 | 8 |
| MF | GO:1901681 | sulfur compound binding | 28/853 | 267/18410 | 4.93E-05 | 0.001573 | 0.001346 | FST/MDK/APLP1/THBS2/MPO/PANK1/F2/RSPO4/RYR2/AZU1/COL25A1/CXCL13/LRTM2/CCL7/RSPO2/COL5A1/APOH/CBS/FGF10/CCN5/PF4/COL11A1/PRSS57/COMP/PCOLCE/PLA2G2D/ELANE/ALDH6A1 | 28 |
| MF | GO:0042379 | chemokine receptor binding | 12/853 | 71/18410 | 9.31E-05 | 0.002854 | 0.002443 | CXCL5/CCL25/CCL21/TFF2/CXCL13/CCL7/CCL11/C10orf99/PF4/CCL19/CCL26/CCL5 | 12 |
| MF | GO:0005230 | extracellular ligand-gated ion channel activity | 12/853 | 73/18410 | 0.000123 | 0.00362 | 0.003099 | GRIN1/P2RX6/GRIN2D/GABRG1/GABRB2/CHRNA1/GRIK4/CHRNA9/GRIN2B/GLRA3/GABRA3/P2RX5 | 12 |
| MF | GO:0015276 | ligand-gated ion channel activity | 18/853 | 145/18410 | 0.000133 | 0.003627 | 0.003106 | GRIN1/P2RX6/CLCA2/GRIN2D/GABRG1/RYR2/GABRB2/KCNJ6/CHRNA1/GRIK4/ASIC1/CHRNA9/GRIN2B/GLRA3/TRPM8/GABRA3/CNGA3/P2RX5 | 18 |
| MF | GO:0022834 | ligand-gated channel activity | 18/853 | 145/18410 | 0.000133 | 0.003627 | 0.003106 | GRIN1/P2RX6/CLCA2/GRIN2D/GABRG1/RYR2/GABRB2/KCNJ6/CHRNA1/GRIK4/ASIC1/CHRNA9/GRIN2B/GLRA3/TRPM8/GABRA3/CNGA3/P2RX5 | 18 |
| MF | GO:0004222 | metalloendopeptidase activity | 15/853 | 111/18410 | 0.000184 | 0.004856 | 0.004157 | ADAMTS14/TRABD2A/ADAM33/CLCA2/TLL2/PRSS2/ADAM12/TMPRSS6/MMP9/MMP23B/ADAMDEC1/ADAM7/MMP7/MMP12/MMP13 | 15 |
| MF | GO:0046873 | metal ion transmembrane transporter activity | 37/853 | 428/18410 | 0.000209 | 0.005345 | 0.004576 | GRIN1/TRPV3/CACNG7/SLC30A3/CACNG4/SCN4A/MFSD2A/CACNG6/KCNQ2/GRIN2D/SLC13A2/TRPM3/SLC5A5/CACNA1G/RYR2/CACNA1B/KCNJ6/ATP1A3/SLC10A2/GRIK4/SLC6A11/SLC8A2/FXYD4/ASIC1/SLC12A8/CHRNA9/GRIN2B/KCNS1/SLC18A2/KCNH3/TRPM8/KCNN4/SLC5A1/NIPAL4/SLC5A8/KCNK17/ATP4B | 37 |
| MF | GO:0022836 | gated channel activity | 31/853 | 340/18410 | 0.000267 | 0.006467 | 0.005536 | GRIN1/CACNG7/P2RX6/CACNG4/SCN4A/CLCA2/CACNG6/KCNQ2/GRIN2D/TRPM3/CACNA1G/GABRG1/RYR2/CACNA1B/GABRB2/KCNJ6/CHRNA1/GRIK4/ASIC1/CHRNA9/GRIN2B/GLRA3/KCNS1/KCNH3/TRPM8/KCNN4/GABRA3/KCNK17/CNGA3/P2RX5/CLIC3 | 31 |
| MF | GO:0005518 | collagen binding | 11/853 | 68/18410 | 0.00027 | 0.006467 | 0.005536 | COCH/MRC2/TGFBI/C1QTNF1/LUM/LRRC15/MMP9/COMP/PCOLCE/MMP12/MMP13 | 11 |
| MF | GO:0004181 | metallocarboxypeptidase activity | 7/853 | 29/18410 | 0.000285 | 0.006616 | 0.005664 | CPZ/CPA2/CPA1/CPXM1/CPB2/CPA5/CPA4 | 7 |
| MF | GO:0019825 | oxygen binding | 8/853 | 39/18410 | 0.000352 | 0.00792 | 0.006781 | TDO2/HBA2/CYP19A1/HBQ1/CYP3A4/CBS/CYP17A1/HBA1 | 8 |
| MF | GO:0022824 | transmitter-gated ion channel activity | 10/853 | 60/18410 | 0.000397 | 0.008218 | 0.007036 | GRIN1/GRIN2D/GABRG1/GABRB2/CHRNA1/GRIK4/CHRNA9/GRIN2B/GLRA3/GABRA3 | 10 |
| MF | GO:0022835 | transmitter-gated channel activity | 10/853 | 60/18410 | 0.000397 | 0.008218 | 0.007036 | GRIN1/GRIN2D/GABRG1/GABRB2/CHRNA1/GRIK4/CHRNA9/GRIN2B/GLRA3/GABRA3 | 10 |
| MF | GO:0005126 | cytokine receptor binding | 26/853 | 272/18410 | 0.000397 | 0.008218 | 0.007036 | IFNG/PRLR/LTB/CXCL5/IL11/ECM1/CCL25/GREM1/EFNA5/CCL21/TFF2/LEFTY1/CXCL13/AMH/CCL7/CCL11/IFNL1/C10orf99/PF4/CCL19/PYCARD/EPO/CSF2/CCL26/CCL5/IL21 | 26 |
| MF | GO:0005179 | hormone activity | 15/853 | 122/18410 | 0.00052 | 0.010358 | 0.008867 | ERFE/CGA/CHGB/HCRT/CCL25/HAMP/AMH/ADCYAP1/INSL3/INHBE/EDN3/GRP/EPO/VGF/UCN2 | 15 |
| MF | GO:0008083 | growth factor activity | 18/853 | 162/18410 | 0.000527 | 0.010358 | 0.008867 | MDK/GDF5/REG1A/IL11/F2/BMP5/LEFTY1/AMH/INHBE/FGF5/EREG/TFF1/FGF10/TIMP1/FGF8/CSF2/VGF/ARTN | 18 |
| MF | GO:0019841 | retinol binding | 5/853 | 16/18410 | 0.000601 | 0.011511 | 0.009854 | CRABP1/CRABP2/STRA6/C8G/ADH4 | 5 |
| MF | GO:0030547 | signaling receptor inhibitor activity | 8/853 | 43/18410 | 0.000703 | 0.013143 | 0.011252 | FST/MTRNR2L3/MTRNR2L6/LYPD1/MTRNR2L1/DKKL1/LY6H/CCL5 | 8 |
| MF | GO:0042165 | neurotransmitter binding | 5/853 | 17/18410 | 0.000819 | 0.014941 | 0.012792 | GRIN1/GRIN2D/CHRNA1/CHRM3/SLC6A11 | 5 |
| MF | GO:0042834 | peptidoglycan binding | 5/853 | 18/18410 | 0.001091 | 0.019442 | 0.016644 | REG1A/REG3G/PGLYRP2/IGHM/REG1B | 5 |
| MF | GO:0071855 | neuropeptide receptor binding | 7/853 | 36/18410 | 0.001149 | 0.01979 | 0.016942 | CCKBR/HCRT/NMU/ADCYAP1/EDN3/KISS1/UCN2 | 7 |
| MF | GO:0048407 | platelet-derived growth factor binding | 4/853 | 11/18410 | 0.001163 | 0.01979 | 0.016942 | PDGFRA/COL5A1/COL1A1/COL1A2 | 4 |
| MF | GO:0048020 | CCR chemokine receptor binding | 8/853 | 47/18410 | 0.001296 | 0.021578 | 0.018473 | CCL25/CCL21/CXCL13/CCL7/CCL11/CCL19/CCL26/CCL5 | 8 |
| MF | GO:0030280 | structural constituent of skin epidermis | 7/853 | 37/18410 | 0.001362 | 0.021833 | 0.018692 | PI3/KRT5/KRT4/KRT79/KRT6A/KRT78/KRT6B | 7 |
| MF | GO:0022803 | passive transmembrane transporter activity | 38/853 | 490/18410 | 0.001377 | 0.021833 | 0.018692 | GRIN1/TRPV3/CACNG7/ATP5F1EP2/P2RX6/GJB6/CACNG4/SCN4A/CLCA2/CACNG6/KCNQ2/GRIN2D/TRPM3/APOL1/CACNA1G/GABRG1/RYR2/CACNA1B/GABRB2/KCNJ6/CHRNA1/GRIK4/FXYD4/ASIC1/CHRNA9/GRIN2B/GLRA3/KCNS1/KCNH3/TRPM8/KCNN4/GABRA3/SLC5A8/KCNK17/CNGA3/AQP8/P2RX5/CLIC3 | 38 |
| MF | GO:0004745 | NAD-retinol dehydrogenase activity | 5/853 | 19/18410 | 0.001425 | 0.021833 | 0.018692 | ADH1C/HSD17B6/RDH16/SDR16C5/ADH4 | 5 |
| MF | GO:0016918 | retinal binding | 5/853 | 19/18410 | 0.001425 | 0.021833 | 0.018692 | CRABP1/CRABP2/STRA6/ADH4/OPN4 | 5 |
| MF | GO:0005216 | ion channel activity | 35/853 | 442/18410 | 0.001473 | 0.022119 | 0.018936 | GRIN1/TRPV3/CACNG7/ATP5F1EP2/P2RX6/CACNG4/SCN4A/CLCA2/CACNG6/KCNQ2/GRIN2D/TRPM3/APOL1/CACNA1G/GABRG1/RYR2/CACNA1B/GABRB2/KCNJ6/CHRNA1/GRIK4/FXYD4/ASIC1/CHRNA9/GRIN2B/GLRA3/KCNS1/KCNH3/TRPM8/KCNN4/GABRA3/KCNK17/CNGA3/P2RX5/CLIC3 | 35 |
| MF | GO:0004175 | endopeptidase activity | 34/853 | 432/18410 | 0.001889 | 0.027829 | 0.023825 | PRTN3/ADAMTS14/TRABD2A/ADAM33/F3/C1R/CLCA2/CELA1/PRSS3/F2/TLL2/PRSS2/PRSS21/TMPRSS11D/AZU1/ADAM12/PCSK1/PLAU/TMPRSS6/KLK11/KLK13/MMP9/HPR/MMP23B/KLK5/ADAMDEC1/PRSS57/PYCARD/KLK10/ADAM7/ELANE/MMP7/MMP12/MMP13 | 34 |
| MF | GO:0019955 | cytokine binding | 15/853 | 141/18410 | 0.002296 | 0.033178 | 0.028405 | PRLR/IL1R2/IL31RA/GDF5/IL2RG/NLRP7/IL1RL1/GREM1/IL22RA2/IL20RB/SOSTDC1/CXCR3/COMP/PDPN/ELANE | 15 |
| MF | GO:0015267 | channel activity | 37/853 | 489/18410 | 0.002417 | 0.033839 | 0.02897 | GRIN1/TRPV3/CACNG7/ATP5F1EP2/P2RX6/GJB6/CACNG4/SCN4A/CLCA2/CACNG6/KCNQ2/GRIN2D/TRPM3/APOL1/CACNA1G/GABRG1/RYR2/CACNA1B/GABRB2/KCNJ6/CHRNA1/GRIK4/FXYD4/ASIC1/CHRNA9/GRIN2B/GLRA3/KCNS1/KCNH3/TRPM8/KCNN4/GABRA3/KCNK17/CNGA3/AQP8/P2RX5/CLIC3 | 37 |
| MF | GO:0005178 | integrin binding | 16/853 | 156/18410 | 0.00243 | 0.033839 | 0.02897 | SFRP2/EMILIN1/IBSP/CASR/ITGA2B/PTPRZ1/TGFBI/DMP1/COL5A1/CCN5/FRMD5/CD177/EGFL6/GFAP/COMP/FCER2 | 16 |
| MF | GO:0099094 | ligand-gated cation channel activity | 13/853 | 115/18410 | 0.002574 | 0.03521 | 0.030144 | GRIN1/P2RX6/GRIN2D/RYR2/KCNJ6/CHRNA1/GRIK4/ASIC1/CHRNA9/GRIN2B/TRPM8/CNGA3/P2RX5 | 13 |
| MF | GO:0005261 | cation channel activity | 28/853 | 345/18410 | 0.00297 | 0.039909 | 0.034167 | GRIN1/TRPV3/CACNG7/ATP5F1EP2/P2RX6/CACNG4/SCN4A/CACNG6/KCNQ2/GRIN2D/TRPM3/CACNA1G/RYR2/CACNA1B/KCNJ6/CHRNA1/GRIK4/FXYD4/ASIC1/CHRNA9/GRIN2B/KCNS1/KCNH3/TRPM8/KCNN4/KCNK17/CNGA3/P2RX5 | 28 |
| MF | GO:0048019 | receptor antagonist activity | 6/853 | 32/18410 | 0.003132 | 0.041366 | 0.035414 | FST/MTRNR2L3/MTRNR2L6/MTRNR2L1/DKKL1/CCL5 | 6 |
| MF | GO:0005262 | calcium channel activity | 13/853 | 119/18410 | 0.003476 | 0.045132 | 0.038638 | GRIN1/TRPV3/CACNG7/CACNG4/CACNG6/GRIN2D/TRPM3/CACNA1G/RYR2/CACNA1B/CHRNA9/GRIN2B/TRPM8 | 13 |
| MF | GO:0004180 | carboxypeptidase activity | 7/853 | 44/18410 | 0.003828 | 0.048871 | 0.04184 | CPZ/CPA2/CPA1/CPXM1/CPB2/CPA5/CPA4 | 7 |
| MF | GO:0015085 | calcium ion transmembrane transporter activity | 14/853 | 135/18410 | 0.003991 | 0.049447 | 0.042333 | GRIN1/TRPV3/CACNG7/CACNG4/CACNG6/GRIN2D/TRPM3/CACNA1G/RYR2/CACNA1B/SLC8A2/CHRNA9/GRIN2B/TRPM8 | 14 |
| MF | GO:0046943 | carboxylic acid transmembrane transporter activity | 16/853 | 164/18410 | 0.004002 | 0.049447 | 0.042333 | SLC22A13/SLC38A5/MFSD2A/SLC27A2/SLC13A2/SLC7A4/SLC6A19/SLC7A13/SLC10A2/SLC6A18/SLC25A21/SLC6A11/SLC7A3/SLC38A3/SLC5A8/SLC16A9 | 16 |
| MF | GO:0005237 | inhibitory extracellular ligand-gated ion channel activity | 4/853 | 15/18410 | 0.004146 | 0.050411 | 0.043158 | GABRG1/GABRB2/GLRA3/GABRA3 | 4 |
| MF | GO:0005342 | organic acid transmembrane transporter activity | 16/853 | 165/18410 | 0.004247 | 0.050834 | 0.04352 | SLC22A13/SLC38A5/MFSD2A/SLC27A2/SLC13A2/SLC7A4/SLC6A19/SLC7A13/SLC10A2/SLC6A18/SLC25A21/SLC6A11/SLC7A3/SLC38A3/SLC5A8/SLC16A9 | 16 |
| MF | GO:1904315 | transmitter-gated ion channel activity involved in regulation of postsynaptic membrane potential | 7/853 | 46/18410 | 0.004936 | 0.058174 | 0.049804 | GRIN2D/GABRG1/GABRB2/CHRNA1/GRIK4/CHRNA9/GABRA3 | 7 |
