# Supplementary material for: NUDT1 Could Be a Prognostic Biomarker and Correlated with Immune Infiltration in Clear Cell Renal Cell Carcinoma
Source: Appl Bionics Biomech. 2022 Dec 26;2022:3669296. doi: 10.1155/2022/3669296 (PMC9808898; doi:10.1155/2022/3669296)
Supplement: Supplementary 5 — KEGG enrichment analysis of DEGs. [file 3669296.f5.docx]

KEGG enrichment analysis of DEGs

| ID | Description | GeneRatio | BgRatio | pvalue | p.adjust | qvalue | geneID | Count |
| --- | --- | --- | --- | --- | --- | --- | --- | --- |
| hsa04080 | Neuroactive ligand-receptor interaction | 43/361 | 362/8145 | 2.15E-09 | 4.32E-07 | 3.98E-07 | GRIN1/PRLR/CGA/GALR1/CCKBR/NPBWR1/P2RX6/HCRT/GRIN2D/PRSS3/F2/PRSS2/GABRG1/NMU/OPRD1/GABRB2/NPY4R/CHRNA1/CHRM3/NPFFR2/ADCYAP1/INSL3/GRIK4/EDN3/SSTR3/CHRNA9/ADRA1D/BRS3/GRIN2B/TAC3/AVPR1B/GLRA3/AGTR1/KISS1/CNR2/MTNR1A/GRP/PTGER1/GABRA3/VGF/UCN2/TACR3/P2RX5 | 43 |
| hsa04974 | Protein digestion and absorption | 21/361 | 103/8145 | 3.09E-09 | 4.32E-07 | 3.98E-07 | COL22A1/PRSS3/PRSS2/CPA2/SLC6A19/COL25A1/ATP1A3/COL10A1/COL19A1/SLC8A2/COL26A1/COL5A1/CPA1/COL1A1/COL11A1/KCNN4/COL1A2/COL8A2/CPB2/COL6A3/COL7A1 | 21 |
| hsa04060 | Cytokine-cytokine receptor interaction | 34/361 | 295/8145 | 2.44E-07 | 2.28E-05 | 2.10E-05 | IFNG/PRLR/IL1R2/IL31RA/GDF5/LTB/CXCL5/IL2RG/IL11/IL1RL1/CCL25/TNFRSF17/IL19/BMP5/TNFRSF13B/IL20RB/CCL21/CXCL13/AMH/CCL7/INHBE/CCL11/IFNL1/PF4/CXCR3/CCL19/EPO/CSF2/CCL26/CCL5/IL21/IL17C/TNFRSF18/IL17B | 34 |
| hsa04061 | Viral protein interaction with cytokine and cytokine receptor | 14/361 | 100/8145 | 0.00012 | 0.008424 | 0.007759 | CXCL5/IL2RG/CCL25/IL19/IL20RB/CCL21/CXCL13/CCL7/CCL11/PF4/CXCR3/CCL19/CCL26/CCL5 | 14 |
| hsa04727 | GABAergic synapse | 12/361 | 89/8145 | 0.000519 | 0.024972 | 0.023 | SLC38A5/GABRG1/CACNA1B/GABRB2/KCNJ6/SLC6A11/GNB3/GNG4/SLC38A3/PRKCG/GABRA3/GNG8 | 12 |
| hsa04972 | Pancreatic secretion | 13/361 | 102/8145 | 0.000535 | 0.024972 | 0.023 | CLCA2/PRSS3/PRSS2/CPA2/PLA2G1B/RYR2/ATP1A3/CHRM3/PNLIPRP2/CPA1/PRKCG/CPB2/PLA2G2D | 13 |
| hsa05143 | African trypanosomiasis | 7/361 | 37/8145 | 0.001031 | 0.039716 | 0.036581 | IFNG/HBA2/APOL1/HPR/PRKCG/HBA1/APOA1 | 7 |
| hsa04713 | Circadian entrainment | 12/361 | 97/8145 | 0.001135 | 0.039716 | 0.036581 | GRIN1/GRIN2D/CACNA1G/RYR2/KCNJ6/ADCYAP1/GNB3/GRIN2B/GNG4/MTNR1A/PRKCG/GNG8 | 12 |
| hsa00140 | Steroid hormone biosynthesis | 9/361 | 61/8145 | 0.001343 | 0.041782 | 0.038483 | UGT1A10/AKR1D1/SULT2B1/CYP19A1/CYP3A4/HSD17B6/SRD5A2/CYP17A1/HSD11B1 | 9 |
| hsa04512 | ECM-receptor interaction | 11/361 | 88/8145 | 0.00166 | 0.042396 | 0.039049 | THBS2/IBSP/ITGA2B/FREM2/FREM1/SV2C/DMP1/COL1A1/COL1A2/COMP/COL6A3 | 11 |
| hsa05033 | Nicotine addiction | 7/361 | 40/8145 | 0.001666 | 0.042396 | 0.039049 | GRIN1/GRIN2D/GABRG1/CACNA1B/GABRB2/GRIN2B/GABRA3 | 7 |
| hsa05410 | Hypertrophic cardiomyopathy | 11/361 | 90/8145 | 0.001994 | 0.046517 | 0.042845 | CACNG7/CACNG4/CACNG6/ITGA2B/RYR2/MYL2/MYH7/SLC8A2/DES/TNNI3/MYH6 | 11 |
| hsa04020 | Calcium signaling pathway | 21/361 | 240/8145 | 0.002187 | 0.04711 | 0.043391 | GRIN1/CCKBR/P2RX6/GRIN2D/CACNA1G/RYR2/CACNA1B/CHRM3/PDGFRA/FGF5/SLC8A2/ADRA1D/FGF10/ITPKA/AVPR1B/AGTR1/PTGER1/PRKCG/FGF8/TACR3/P2RX5 | 21 |
