# Supplementary material for: NUDT1 Could Be a Prognostic Biomarker and Correlated with Immune Infiltration in Clear Cell Renal Cell Carcinoma
Source: Appl Bionics Biomech. 2022 Dec 26;2022:3669296. doi: 10.1155/2022/3669296 (PMC9808898; doi:10.1155/2022/3669296)
Supplement: Supplementary 6 — The correlation between 22 immune cell subtypes and NUDT1 expression level. [file 3669296.f6.docx]

The correlation between 22 immune cell subtypes and NUDT1 expression level

| Cell | cor | pvalue |
| --- | --- | --- |
| B cells naive | 0.00248 | 0.963657 |
| B cells memory | 0.00118 | 0.982703 |
| Plasma cells | 0.067605 | 0.213715 |
| T cells CD8 | 0.187353 | 0.000515 |
| T cells CD4 naive | -0.06336 | 0.243946 |
| T cells CD4 memory resting | -0.30883 | 6.02E-09 |
| T cells CD4 memory activated | 0.134877 | 0.012802 |
| T cells follicular helper | 0.150908 | 0.005298 |
| T cells regulatory (Tregs) | 0.429149 | 1.15E-16 |
| T cells gamma delta | 0.037359 | 0.492354 |
| NK cells resting | -0.08484 | 0.118401 |
| NK cells activated | 0.118948 | 0.028307 |
| Monocytes | -0.22638 | 2.51E-05 |
| Macrophages M0 | 0.270992 | 3.90E-07 |
| Macrophages M1 | -0.31235 | 3.95E-09 |
| Macrophages M2 | -0.13848 | 0.010575 |
| Dendritic cells resting | -0.06128 | 0.259842 |
| Dendritic cells activated | -0.04368 | 0.422034 |
| Mast cells resting | -0.22992 | 1.86E-05 |
| Mast cells activated | 0.026488 | 0.626474 |
| Eosinophils | -0.12072 | 0.026022 |
| Neutrophils | -0.03354 | 0.53764 |
