# Supplementary material for: NUDT1 Could Be a Prognostic Biomarker and Correlated with Immune Infiltration in Clear Cell Renal Cell Carcinoma
Source: Appl Bionics Biomech. 2022 Dec 26;2022:3669296. doi: 10.1155/2022/3669296 (PMC9808898; doi:10.1155/2022/3669296)
Supplement: Supplementary 7 — The correlation between immune checkpoints and NUDT1 expression level. [file 3669296.f7.docx]

The correlation between immune checkpoints and NUDT1 expression level

| Query | Gene | cor | pvalue |
| --- | --- | --- | --- |
| NUDT1 | CD70 | 0.267293 | 3.73E-10 |
| NUDT1 | PDCD1 | 0.287023 | 1.51E-11 |
| NUDT1 | CD40 | 0.215403 | 5.29E-07 |
| NUDT1 | LAG3 | 0.323218 | 2.11E-14 |
| NUDT1 | CD276 | 0.345292 | 2.42E-16 |
| NUDT1 | TNFRSF9 | 0.192326 | 7.92E-06 |
| NUDT1 | TNFRSF4 | 0.180781 | 2.73E-05 |
| NUDT1 | TNFSF14 | 0.241878 | 1.61E-08 |
| NUDT1 | CD244 | 0.148018 | 0.000615 |
| NUDT1 | TNFSF4 | 0.149487 | 0.000542 |
| NUDT1 | CD274 | -0.19908 | 3.70E-06 |
| NUDT1 | CD200 | -0.15273 | 0.000408 |
| NUDT1 | NRP1 | -0.30222 | 1.07E-12 |
| NUDT1 | TNFSF9 | 0.255708 | 2.18E-09 |
| NUDT1 | TNFSF15 | -0.17885 | 3.34E-05 |
| NUDT1 | CD48 | 0.143845 | 0.000877 |
| NUDT1 | LGALS9 | 0.445798 | 2.44E-27 |
| NUDT1 | TIGIT | 0.21372 | 6.51E-07 |
| NUDT1 | CD27 | 0.237221 | 3.06E-08 |
| NUDT1 | TMIGD2 | 0.395408 | 2.35E-21 |
| NUDT1 | LAIR1 | 0.23844 | 2.59E-08 |
| NUDT1 | CTLA4 | 0.166979 | 0.000109 |
| NUDT1 | CD44 | 0.254448 | 2.63E-09 |
| NUDT1 | TNFRSF8 | 0.343881 | 3.26E-16 |
| NUDT1 | TNFRSF18 | 0.510433 | 1.23E-36 |
| NUDT1 | HHLA2 | -0.17146 | 7.04E-05 |
